# Asymmetric intramolecular α-cyclopropanation of aldehydes using a donor/acceptor carbene mimetic

**DOI:** 10.1038/ncomms10041

**Published:** 2015-12-08

**Authors:** Chaosheng Luo, Zhen Wang, Yong Huang

**Affiliations:** 1 Key Laboratory of Chemical Genomics, Shenzhen Graduate School, Peking University, Xili University Town, Shenzhen 518055, China

## Abstract

Enantioselective α-alkylation of carbonyl is considered as one of the most important processes for asymmetric synthesis. Common alkylation agents, that is, alkyl halides, are notorious substrates for both Lewis acids and organocatalysts. Recently, olefins emerged as a benign alkylating species via photo/radical mechanisms. However, examples of enantioselective alkylation of aldehydes/ketones are scarce and direct asymmetric dialkylation remains elusive. Here we report an intramolecular α-cyclopropanation reaction of olefinic aldehydes to form chiral cyclopropane aldehydes. We demonstrate that an α-iodo aldehyde can function as a donor/acceptor carbene equivalent, which engages in a formal [2+1] annulation with a tethered double bond. Privileged bicyclo[3.1.0]hexane-type scaffolds are prepared in good optical purity using a chiral amine. The synthetic utility of the products is demonstrated by versatile transformations of the bridgehead formyl functionality. We expect the concept of using α-iodo iminium as a donor/acceptor carbene surrogate will find wide applications in chemical reaction development.

## Abstract (web summary)

The formation of chiral centres adjacent to carbonyl groups is a highly challenging task in asymmetric catalysis. Here, the authors report the asymmetric diakylation of carbonyls via an intramolceular α-cyclopropanation procedure.

## Graphical abstract

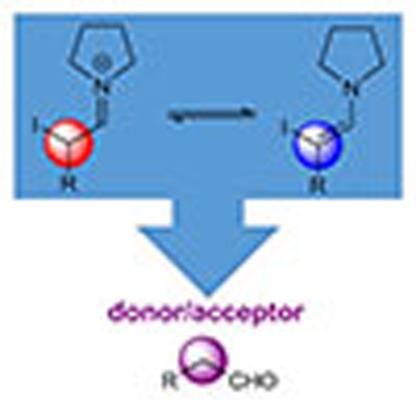

Asymmetric α-alkylation of carbonyl compounds plays a vital role in organic synthesis^1^. It was traditionally accomplished via chiral enolate chemistry^2^,^3^,^4^. Modified carbonyls, such as tin enolates and acidic imino esters, have been successfully alkylated under Lewis acid or phase transfer catalysis^5^,^6^,^7^. However, the more useful direct α-alkylation of aldehyde/ketone remains unsolved. Progress in this area benefited from the recent advance of enamine organocatalysis. Limited success was received using alkyl halides or stabilized carbocation precursors^8^,^9^,^10^,^11^,^12^,^13^,^14^,^15^,^16^,^17^,^18^,^19^. Practically, alkyl halides are not well tolerated by either Lewis acids or nucleophilic organocatalysts because of their strong electrophilicity and interference by forming strong hydrogen halide by-products. Recently, olefins became a viable alkylation agent under mild *redox* catalysis^20^,^21^.

Cyclopropane is a preferred structural motif for medicinal chemistry, owing to its rigidity and metabolic stability^22^. In particular, ring-fused cyclopropanes are prevalent among bioactive natural products and drug molecules. In sharp contrast to their biological stability, fused cyclopropanes carry unique chemical reactivity for fragmentation and rearrangement that is often exploited in natural product synthesis^23^,^24^,^25^. To access these structurally unique scaffolds, a number of synthetic methods have been developed. Typically, these compounds were made via an annulation reaction between an olefin and a tethering one carbon donor/acceptor such as metal carbenoids^26^,^27^,^28^,^29^,^30^,^31^,^32^,^33^,^34^,^35^,^36^,^37^,^38^. Nevertheless, asymmetric synthesis of bicyclic cyclopropanes remains very challenging^27^,^29^,^37^.

Since the renaissance of enamine-iminium catalysis in 2000 (refs 39, 40), enantioselective α-functionalization of carbonyls has taken the centre stage for synthetic innovation. Despite tremendous advance in this field, examples of direct amine catalysed α-alkylation of aldehydes are rare. Among them, two intramolecular α-alkylation strategies are notable: in 2004, Vignola and List reported the first enamine catalysed intramolecular alkylation using halo aldehydes^8^. Using this strategy, a cyclopropane aldehyde product was prepared. In 2013, MacMillan and co-workers accomplished intramolecular α-alkylation of aldehydes having a tethering double bond via enamine-singly occupied molecular orbital catalysis^41^. Alternatively, both MacMillan and Córdova reported methods to generate α-cyclopropane aldehydes from α,β-unsaturated aldehydes using an iminium-enamine cascade strategy^42^,^43^,^44^,^45^. Overviewing recent progress of organocatalytic α-alkylation reactions, efforts have been concentrated on replacing only one hydrogen atom at the α position of aldehydes with alkylating agents. In sharp contrast, double alkylation of the carbonyl α-methylene has been largely neglected^13^.

Herein, we report an orthogonal approach for direct α-cyclopropanation of aldehydes via intramolecular C=C/CH_2_ annulation, enabling rapid access to bicyclo[3.1.0]hexanes and their aza analogues (Fig. 1). This strategy uses an α-iodo iminium species as a key donor/acceptor carbene mimetic.

## Results

### Rationale design of a new donor/acceptor carbene mimetic

**Figure 2:**
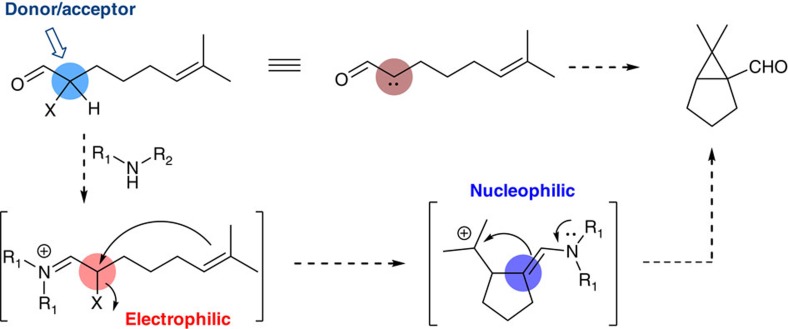
Design of α-halo iminium as a donor/acceptor carbene mimetic. We propose the α-carbon of α-halo aldehyde might serve as a donor/acceptor by the action of a secondary amine. In iminium form, the halide is a good electrophile. In enamine form, the carbon is a good nucleophile.

Recently, we reported an α-allenylation reaction of aldehyde using gold/amine synergistic catalysis, in which the α-methylene of carbonyl was converted to an sp^2^ centre^46^. Along this line, we decided to explore the possibility of forming a cyclopropane through oxidative annulation between the aldehyde α-CH_2_ and a tethering double bond. Conceptually, the net dehydrogenative nature of this transformation is counterintuitive. Normally, cyclopropanation of an olefin requires a donor/acceptor one-carbon counterpart such as a metal carbenoid, whereas the α-carbon of aldehydes is nucleophilic (donor only). We envisioned that an α-halo aldehyde intermediate might serve such a purpose. Upon mixing with a secondary amine, the corresponding α-halo iminium becomes a highly electrophilic alkylation species, whereas its complementary enamine form remains nucleophilic. This donor/acceptor reactivity might be synthetically equivalent to a carbene (Fig. 2).

### Initial attempts on intramolecular α-cyclopropanation

**Figure 3:**
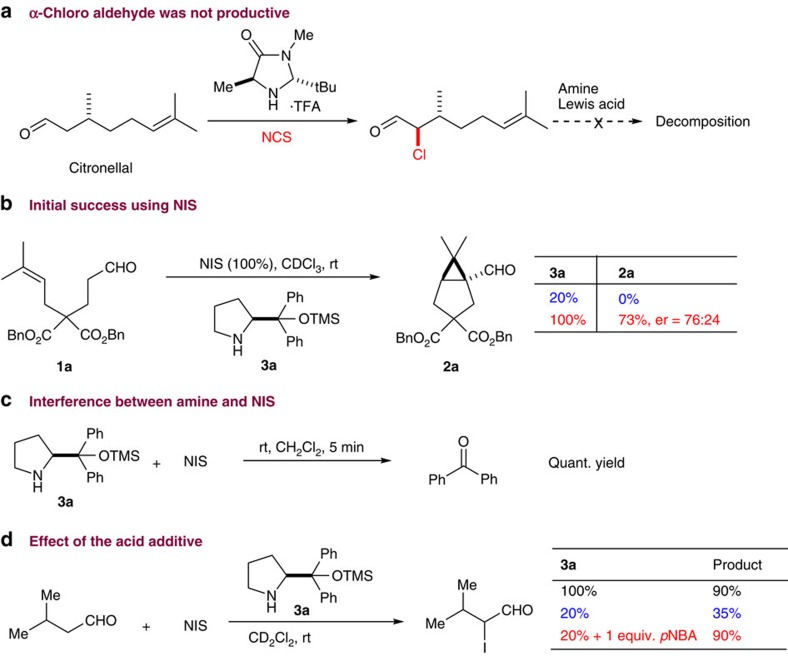
Preliminary exploration of α-halo aldehydes as the key intermediate. (**a**) α-Chloro citronellal failed to undergo α-cyclopropanation using various combinations of amines and Lewis acids. (**b**) The α-cyclopropanation occurred smoothly for substrate **1a** using 1 equiv. NIS and chiral amine **3a**. A catalytic amount of **3a** did not promote this reaction. (**c**) Amine **3a** was found to react with NIS rapidly at room temperature (rt). Benzophenone was found to be the major decomposition product. (**d**) Owing to serious decomposition of **3a** by NIS, α-iodination of isobutyraldehyde requires stoichiometric amine **3a**. However, we found that *para*-nitrobenzoic acid (*p*NBA) stabilizes this amine, allowing for catalytic use. er, enantiomeric ratio; TMS, trimethylsilyl. TFA, trifluoroacetic acid; NCS, N-Chlorosuccinimide; OTMS, trimethylsilyloxyl.

Enantioselective α-halogenation of aldehydes have been reported by several research groups^47^,^48^,^49^,^50^,^51^,^52^,^53^,^54^,^55^,^56^. Among those reported examples, chlorination of citronellal^55^,^56^ seemed suitable as the starting point of our investigation. Unfortunately, attempts to affect the cyclopropanation reaction using α-chloro citronellal were fruitless. In the presence of a secondary amine, no cyclopropane aldehyde was detected. Further activation of the carbon–chloride bond using various Lewis acids resulted in substrate decomposition in most cases (Fig. 3). We speculated that chlorides are not electrophilic enough to react with an olefin, which is a rather poor close shell nucleophile. Next, we focused our attention on an iodination/annulation cascade. To further facilitate ring closure, we decided to install gem-diesters in the middle of the chain to assist conformational folding for intramolecular reaction (the Thorpe-Ingold effect).

We were excited to find out that treating aldehyde **1a** with 1 equiv. of amine **3a** (ref. 57) and N-iodosuccinimide (NIS) yielded the corresponding bicyclo[3.1.0]hexane aldehyde **2a** in moderate yield and enantiomeric ratio (er; Fig. 3). No reaction occurred in the absence of NIS. Interestingly, a catalytic amount of amine **3a** failed to give any product. Careful examination of the crude reaction mixture revealed that prolinol ether **3a** quickly decomposed to benzophenone and pyrrolidin-2-one. Control experiment showed that mixing **3a** with NIS resulted in full conversion to benzophenone within 5 min at room temperature. As a result, α-iodination was undermined. The redox lability of amine **3a** is likely a result of high electron density on the nitrogen atom, which might be attenuated using a weak Brønsted acid. Upon screening various acids, we found that *p*-nitrobenzoic acid (*p*NBA) stabilizes **3a** under the reaction condition. When simple isopentaldehyde was subjected to the α-iodination reaction, the product was obtained in 35% yield using 20 mol% **3a**, compared with a 90% conversion with 1 equiv. of **3a**. Upon adding *p*NBA (1 equiv.) as additive, 90% yield was obtained using 20 mol% **3a** and no decomposition of the amine was observed.

### Optimization of the NIS-mediated α-cyclopropanation

Even though we were able to address the amine decomposition problem, the overall cyclopropanation cascade performed poorly using catalytic amine. Nevertheless, the use of *p*NB*A* continued to improve the efficiency of reactions employing 1 equiv. **3a**. We next examined solvent effect. The reaction proceeds in a number of non-polar solvents (Table 1, entries 1–6). Good enantioselectivity was observed in toluene and ether. Various iodination reagents were also investigated. 1,3-Diiodo-5,5-dimethylhydantoin afforded a very similar result as NIS (Table 1, entry 7). Stronger I^+^ reagents, such as I_2_, ICl, IBr, led to very low conversions (Table 1, entries 8–11). Substrate decomposition was predominant. The amine promoters were studied systematically. The MacMillan's imidazolidinone catalyst^40^ was not productive (Table 1, entry 16). 2,5-Diarylpyrrolidine **3e**^13a^ afforded racemic **2a** in moderate yield (Table 1, entry 15). Varying the size of the silicon group did not improve selectivity (Table 1, entries 12–14). The original catalyst **3a** afforded the highest conversion. Although the 3,5-(CF_3_)_2_-Ph analogue **3g** led to higher er than **3a** (90.5/9.5 versus 84/16; Table 1, entry 17 and entry 5), the yield dropped substantially. We speculated that both the electronic and the steric nature of the aryl group play an important role: electron-neutral aryls lead to higher yields; bulky 3,5-disubstitution improves er. Following this rationale, we prepared the corresponding 3,5-(*t*-Bu)_2_ amine **3l**. Improved yield was obtained at no cost of the selectivity (Table 1, entry 22). With this optimized chiral amine in hand, we quickly re-examined solvent effect. Further improvement of both yield and er was achieved using *i*Pr_2_O as the reaction media (Table 1, entry 24, 75% yield, 93:7 er).

### Substrate scope

The substrate scope was studied next (Table 2). The cyclopropanation occurs smoothly using either carbon or nitrogen as the linker between carbonyl and olefin. Only *cis*-bicyclic isomers were isolated because of ring strain control. However, gem-disubstitution is required for the all-carbon linkers, without which the reaction becomes very slow. Trialkyl substituted double bond affords highest yield and er. Exo-cyclic double bonds were well tolerated, leading to bicyclo[3.1.0]hexanes with an additional ring spiro to the cyclopropane (Table 2, products **2f**–**2h**). The selectivity of these substrates increases with the size of the ring. When a cyclic ketal was used as the linker, both high yield and excellent er were obtained (Table 2, product **2i**). Interestingly, a 3-fuse-5-spiro-4 ring skeleton was obtained using an azetidine-containing substrate **1j**. The alkyl substituents at the double bond terminal is essential. Removing these groups led to low reactivity and poor er (Table 2, products **2k** and **2l**). When a tetrasubstituted olefin was used, a fully substituted cyclopropane product with three contiguous all-carbon quaternary centres was obtained as the single *cis*-diastereomer in 71% yield and 91:9 er (Table 2, product **2m**). We also attempted intermolecular α-cyclopropanation reactions using simple aldehydes and various electron-rich olefins. Unfortunately, no desired cyclopropane product was obtained under the standard reaction condition. The α-iodo aldehyde intermediates were the major products. This result suggests a large entropy penalty is required for the key carbon–carbon bond formation.

### Mechanistic experiments

**Figure 4:**
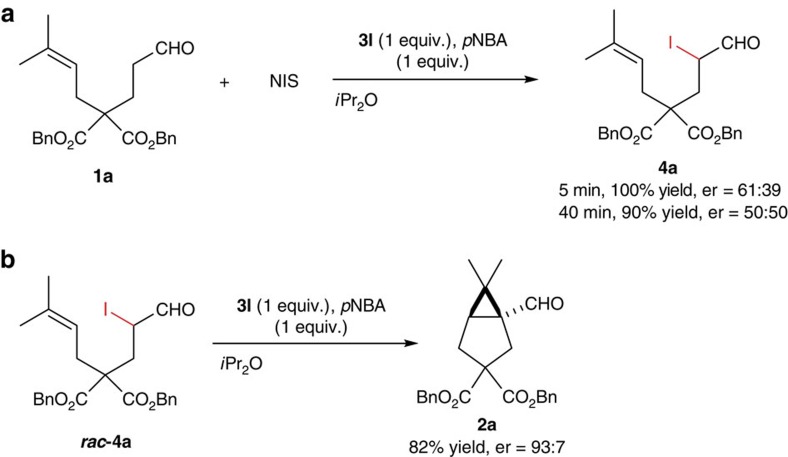
Evidence of α-iodo aldehyde as the key intermediate. (**a**) Under the standard condition for the α-cyclopropanation, α-iodo aldehyde **4a** was isolated after 5 and 40 min. The product was found in low optical purity and quickly racemized during the reaction. (**b**) When racemic **4a** was subjected to amine **3l** in the absence of NIS, product **2a** was obtained in good yield and er, indicating an efficient dynamic kinetic resolution process.

To further understand the mechanism of this unconventional cascade cyclopropanation. We carried out real-time ^1^H NMR experiments using deuterated solvent. The starting material was consumed within 10 min upon addition of NIS. The corresponding α-iodo aldehyde **4a** was obtained in quantitative yield, which was slowly converted to product **2a**. In fact, α-iodo aldehyde **4a** could be isolated by quick flash column chromatography. Subjecting **4a** to amine alone led to the cyclopropane product in good yield without the need of NIS. Therefore, α-iodo aldehyde is very likely the intermediate for the overall cyclopropanation. It is noteworthy that the selectivity of the first step is poor. Er of **4a** was 61:39 upon isolation after 5 min. However, when **4a** was isolated after 40 min, it became racemic (Fig. 4a). This rapid decrease of optical purity for **4a** indicated a facile racemization via enamine formation. Therefore, the stereoselectivity of the overall reaction is solely controlled in the annulation step. Er of the α-iodo intermediate ought to be inconsequential for the ultimate selectivity of the bicyclic product. Indeed, when racemic **4a** was subjected to catalyst **3l** in *i*Pr_2_O, **2a** was isolated in 82% yield and 93:7 er (Fig. 4b).

**Figure 5:**
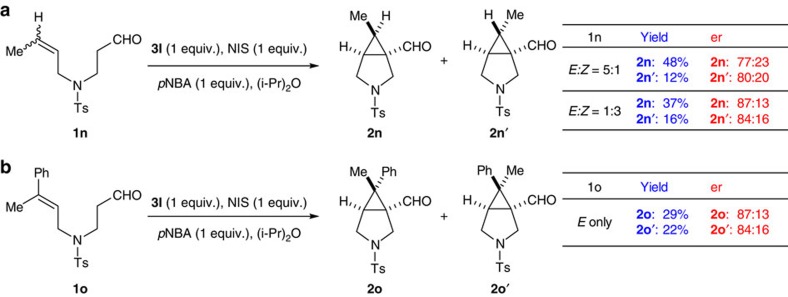
Evidence for the stepwise annulation mechanism. (**a**) When a 5:1 *E*/*Z* mixture of **1n** was subjected to the α-cyclopropanation, products **2n** and **2n**' were obtained as a pair of diastereomers favouring **2n**. Similar diastereomeric ratio and er were observed for a mixture of 1:3 *E*/*Z* isomers. (**b**) Substrate **1o** in pure *E*-form led to a diastereomeric mixture of **2o** and **2o**' in nearly 1:1 ratio. The results in **a** and **b** indicate the formation of the two new carbon–carbon bonds is stepwise.

The annulation step was also studied. We prepared *E*/*Z* isomer pairs of disubstituted olefin **1n**. The *trans*-product **2n** was obtained as the major isomer regardless of the *E*/*Z* ratio of the starting material. Furthermore, a mixture (1.2:1) of diastereomers was obtained when the pure *E*-form of **1o** was used (Fig. 5). Hence, the olefin/iodide annulation is a stepwise process, during which bond rotation occurs. Based on this observation, a free singlet carbene intermediate is highly unlikely. The cyclopropanation could occur through several pathways: (i) halogen extraction by the electron-rich olefin to form an iodiranium species, followed by enamine promoted cyclization and elimination of hydrogen iodide; (ii) double electrophilic alkylation pathway (first, Friedel–Crafts alkylation between olefin and iodide; second, alkylation of enamine by the resulting carbocation, Fig. 2); (iii) radical mechanism by homo-cleavage of the C–I bond. We obtained the X-ray structure of α-iodo aldehyde **4c**. In solid phase, the double bond is oriented 180° away from the iodine atom, an ideal alignment for SN_2_ displacement. Although we could not rule out the possibility of a radical cyclization, attempts to intercept the tertiary radical were unsuccessful. The reaction proceeded with equal efficiency in dark and in the absence of any oxidant, suggesting a radical intermediate was unlikely. At this moment, we believe the reaction mechanism is in line with our original design (Fig. 2).

### Rationale for the stereochemical control

**Figure 6:**
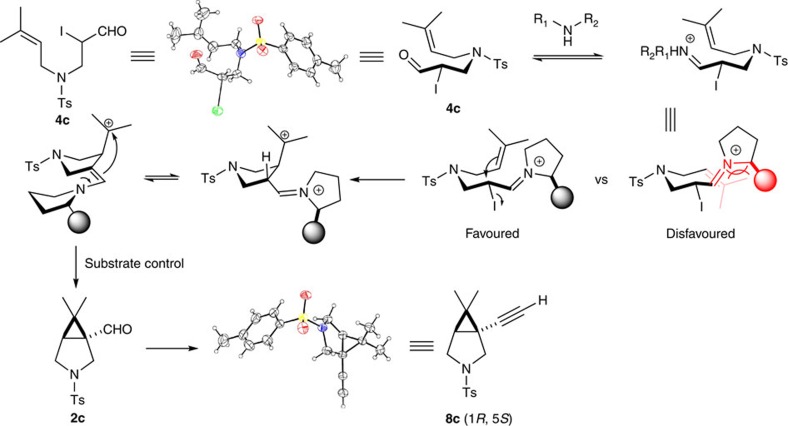
Rationale for the stereoselectivity. There are four possible chair-like conformations for the first C–C bond formation. Owing to the chiral centre on the amine, only the two with the olefin moiety folded away from the bulky substituent are shown. The second C–C bond formation is substrate controlled as the [3.1.0]-bicyclic scaffold is much more stable in *cis*-form.

The electrophilic alkylation is the stereoselectivity determining step, during which the α-iodo iminium intermediate folds into a chair-like conformation. The double bond approaches the α-carbon away from the bulky substituent of the amine and the olefin adopts a pseudo axial orientation in order to avoid steric crowding into the adjacent iminium moiety. This transition state model predicts the absolute stereochemistry of product to be (1*R*, 5*S*), which was confirmed by X-ray crystallography of derivative **8c** (Fig. 6, *vide infra*).

### Synthetic manipulations of the products

**Figure 7:**
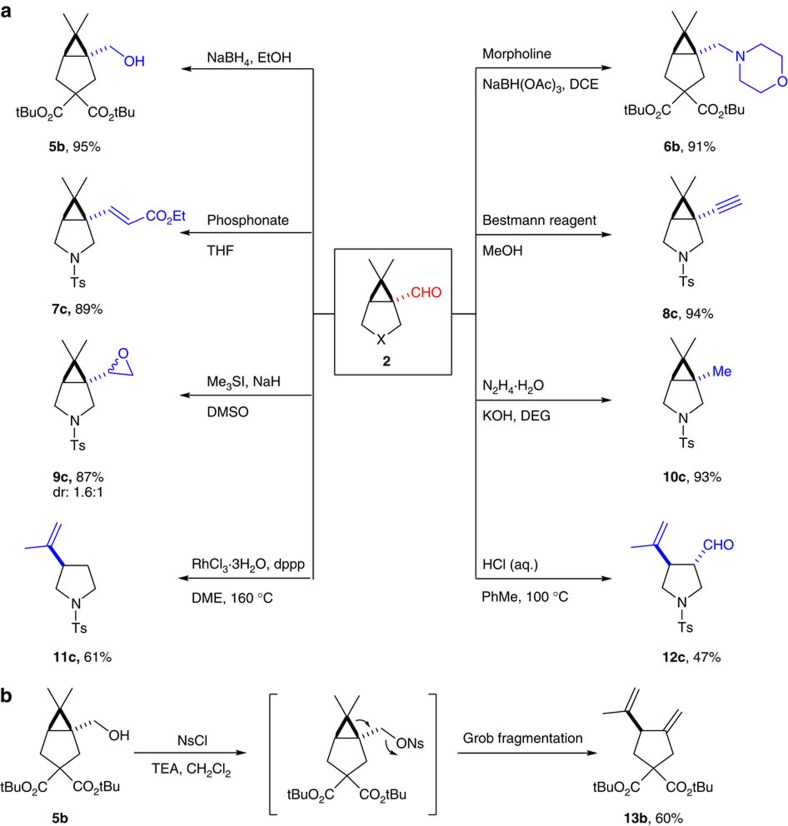
Chemical manipulation of the [3.1.0]-bicyclic aldehyde products. (**a**) The bridgehead formal group can be converted into a number of functionalities. (**b**) Transforming the free OH of **5b** into a good leaving group prompts Grob fragmentation to form a skipped diene. Bestmann reagent, dimethyl (acetyldiazomethyl)phosphonate; DCE, dichloroethane; DEG, diethylene glycol; DME, diglyme; DMSO, dimethyl sulphoxide; dppp, 1,3-bis(diphenylphosphino)propane; NsCl, 4-nitrobenzenesulfonyl chloride; THF, tetrahydrofuran.

The unique bridgehead aldehyde functionality enabled us to perform various chemical manipulations to access a number of chiral scaffolds (Fig. 7). NaBH_4_ reduction of **2b** gave the corresponding alcohol **5b** in 95% yield. Reductive amination using morpholine afforded amine **6b** in 91% yield^58^. Horner–Wadsworth–Emmons reaction of **2c** installed an α,β-unsaturated ester at the bridgehead^59^. Seyferth–Gilbert homolygation using the modified Bestmann's condition generated an alkyne functionality at the [3.1.0] bridgehead^60^. Epoxidation using trimethyl sulfonium iodide afforded **9c** as a mixture of diastereomers^61^. The formyl group of **2c** was smoothly converted to methyl using Huang's modification of Wolff–Kishner reduction^62^. Deformylation using RhCl_3_ resulted concurrent cyclopropane opening to give 3-vinylpyrrolidine **11c** in 61% yield. The aldehyde group was retained in an acid promoted cyclopropane fragmentation to afford 3-formyl-4-vinylpyrrolidine **12c**. Interestingly, skipped diene **13b** was formed via Grob fragmentation^63^ when we carried out sulfonylation on alcohol **5b**. It is noteworthy that most of these derivatives would be very difficult to prepare otherwise.

### Preliminary result for amine catalysis

**Figure 8:**
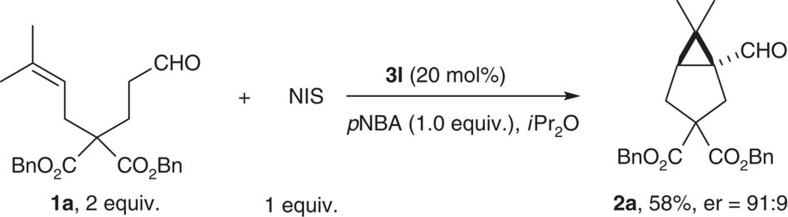
Catalytic turnover of the chiral amine. When **1a** was used in excess, the reaction reached 58% isolated yield using 20 mol% amine **3l**, reflecting a turnover number of 3.

It was intriguing that this cyclopropanation required 100 mol% of the chiral amine to achieve high conversion. Our earlier results showed that the α-iodo aldehyde could be generated in quantitative yield using 20 mol% amine. The lack of amine turnover in the annulation step was likely due to the generation of HI that interfered with the enamine/iminium equilibrium. Various quenching agents were used in an attempt to speed up catalyst regeneration. However, no improvement was observed using bases and silver salts. On the other hand, moderate turnover can be achieved using *p*NBA as buffer and substrate **1a** in excess (Fig. 8). In-depth investigation on further improving catalyst turnover is underway and will be reported in due course.

## Discussion

In summary, we developed the first example of asymmetric intramolecular α-cyclopropanation of aldehydes using a chiral amine promoter. We discovered that the α-iodo iminium functionality could serve as a donor/acceptor carbene mimetic to engage in an intramolecular annulation reaction with an olefin. This strategy enables direct access to privileged chiral bicyclo[3.1.0]hexane scaffolds. The formation of the three-member ring is believed to occur through a stepwise double electrophilic alkylation cascade. This reaction represents the first asymmetric double α-alkylation of aldehydes. The synthetic utility of the ring-fused cyclopropane products is demonstrated by diversified chemical derivatization. We expect the use of α-iodoaldehyde as a versatile donor/acceptor warhead will become a general approach for asymmetric di-functionalization of carbonyl compounds.

## Methods

### General methods and materials

Solvents for reactions were distilled according to general practice before use. All reagents were purchased and used without further purification unless specified otherwise. Amine catalyst was prepared based on the literature report^64^,^65^. Solvents for chromatography were technical grade and distilled before use. Flash chromatography was performed using 200–300 mesh silica gel with the indicated solvent system according to the standard techniques. Analytical thin-layer chromatography was performed using Huanghai silica gel plates with HSGF 254. Qingdao Haiyang Chemical HG/T2354-92 silica gel was used for silica gel flash chromatography. Visualization of the developed chromatogram was performed by ultraviolet absorbance (254 nm) or appropriate stains. ^1^H NMR data were recorded on Bruker nuclear resonance spectrometers (300, 400 or 500 MHz) unless specified otherwise. Chemical shifts (*δ*) in p.p.m. are reported as quoted relative to the residual signals of chloroform (^1^H 7.26 p.p.m. or ^13^C 77.16 p.p.m.). Multiplicities are described as: s (singlet), bs (broad singlet), d (doublet), t (triplet), q (quartet), m (multiplet); and coupling constants (*J*) are reported in Hertz (Hz). ^13^C NMR spectra were recorded on Bruker spectrometers (75, 101 or 126 MHz) with total proton decoupling. High-resolution mass spectrometry (HRMS) (electrospray ionization (ESI)) analysis was performed by the Analytical Instrumentation Center at the Peking University, Shenzhen Graduate School, and HRMS data were reported with ion mass/charge (*m*/*z*) ratios as values in atomic mass units. Chiral HPLC was recorded on a Shimadzu LC-20A spectrometer using Daicel Chiralcel columns. ^1^H NMR, ^13^C NMR and HPLC spectra are provided for all products; see Supplementary Figs 1–106. For The Oak Ridge Thermal Ellipsoid Plot (ORTEP) structures of **2b**, ***rac*****-4c** and **8c**, see Supplementary Figs 107–109. See Supplementary Methods for the characterization data for all compounds. See Supplementary Data 1–3 for X-ray CIF files of compounds **2b**, ***rac*****-4c** and **8c** (CCDC 1405229, 1405230, 1405231).

### Synthesis of substrates

*Synthetic method A for substrates 1a-1b, 1e*. Dibenzyl malonate was converted to dibenzyl 2-allyl-2-(3-methylbut-2-en-1-yl)malonate by double alkylation according to a similar reported procedure^66^. To a solution of this diene (5 mmol, 1 equiv.) in tetrahydrofuran (THF) (50 ml) at 0 °C, 9-borabicyclo[3.3.1]nonane (1.5 equiv., 0.5 M in THF) was slowly added by syringe over 10 min. The reaction was allowed to warm to room temperature and stirred for 3 h. Then 30 ml of water and sodium perborate tetrahydrate (4.5 g) was added slowly (exothermic). The white suspension was stirred overnight. The mixture was filtered to remove the white solid. The filtrate was poured into a separatory funnel and the layers were separated. The aqueous phase was back-extracted three times with 50 ml portions of ether. The combined organic layers were washed with 50 ml of brine. The resulting solution was dried over sodium sulfate, filtered and concentrated to yield a clear oil, which was further purified by flash column chromatography to provide dibenzyl 2-(3-hydroxypropyl)-2-(3-methylbut-2-en-1-yl)malonate. To a flask containing the solution of this primary alcohol in dichloromethane was added pyridinium chlorochromate (1.5 equiv.) and the reaction stirred at room temperature until all the starting material was consumed. Filter to remove the inorganic impurity and the product was isolated by flash column chromatography (ethyl acetate and petrol ether as eluent).

### Synthetic method B for substrates 1c-1d, 1f-1h, 1k-1o

To a solution of N-(3-hydroxypropyl)-4-methylbenzenesulfonamide (5 mmol) in dimethylformamide (DMF) (40 ml), sodium hydride (60% dispersion in mineral oil, 1.2 equiv.) was slowly added. The bubbling solution was stirred for 60 min before 3,3-dimethylallyl bromide (1.3 equiv.) was added (in some cases, heating is required). The reaction was stirred for 16 h before being partitioned between H_2_O and diethyl ether. The organic layer was washed with H_2_O (2 × 40 ml) and brine (40 ml), dried over sodium sulfate, filtered, concentrated and column chromatography can afford the precursor. The final product was obtained by pyridinium chlorochromate (PCC) oxidation.

### Synthetic method C for substrate 1i

Compound dibenzyl 2-allyl-2-(3-methylbut-2-en-1-yl)malonate was reduced to 2-allyl-2-(3-methylbut-2-en-1-yl)propane-1,3-diol with lithium aluminium hydride. To the solution of this diol (5 mmol, 1 equiv.) in THF (100 ml), 2,2-dimethoxypropane (6 mmol, 1.2 equiv.) and PTSA monohydrate (50 mg, 0.25 mmol) were added, the resulting solution was stirred overnight at room temperature. The solvent was removed and the residue was purified by silica gel chromatography to yield 5-allyl-2,2-dimethyl-5-(3-methylbut-2-en-1-yl)-1,3-dioxane. This compound subsequently underwent hydroboration/oxidation and subsequent PCC oxidation to afford the desired product (similar procedure as described above).

### Synthetic method D for substrate **1j**

To a cooled (−20 °C) solution of 2-allyl-2-(3-methylbut-2-en-1-yl)propane-1,3-diol (5 mmol, 1 equiv.) in dichloromethane (50 ml), triethylamine (20 mmol, 4 equiv.) and methanesulfonyl chloride (10 mmol, 2 equiv.) were sequentially added. The mixture was stirred for 30 min, then warmed to ambient temperature and partitioned between ethyl acetate and 1 N HCl. The organic layer was washed with brine, dried over MgSO_4_ and concentrated *in vacuo* to provide 2-allyl-2-(3-methylbut-2-en-1-yl)propane-1,3-diyl dimethanesulfonate. The solution of the resulting dimesylate, potassium carbonate (15 mmol, 3 equiv.) and tosyl amide (7.5 mmol, 1.5 equiv.) in dimethylsulphoxide (50 ml) was heated at 90 °C for 30 h. When cooled to room temperature, water (50 ml) was added, and the product was extracted with dichloromethane (3 × 50 ml). The combined organic layer was washed with brine (3 × 30 ml). Dried over sodium sulfate, filtered, concentrated and purified by column chromatography to deliver 3-allyl-3-(3-methylbut-2-en-1-yl)-1-tosylazetidine. The final product **1j** was obtained by sequential hydroboration/oxidation and subsequent PCC oxidation (similar procedure as described above).

### General procedure for α-cyclopropanation of aldehydes

A 2-dram vial was charged with a solution of substrate **1** (0.1 mmol, 1.0 equiv.) in diisopropyl ether (1 ml). Amine **3l** (0.1 mmol, 1.0 equiv.) and *p*NBA (0.1 mmol, 1.0 equiv.) were added sequentially. The reaction mixture was stirred for 5 min before NIS (0.1 mmol, 1.0 equiv.) was added. The reaction vessel was capped with a screw cap and stirred for 24 h at 25 °C. The reaction mixture was directly subjected to silica gel flash column chromatography (ethyl acetate/petrol ether) to afford product **2**. A racemic sample was prepared similarly using racemic amine.

## Additional information

**Accession codes**: The X-ray crystallographic coordinates for structures reported in this study have been deposited at the Cambridge Crystallographic Data Centre (CCDC), under deposition numbers CCDC 1405229, 1405230, 1405231. These data can be obtained free of charge from the Cambridge Crystallographic Data Centre via www.ccdc.cam.ac.uk/data_request/cif.

**How to cite this article:** Luo, C. *et al.* Asymmetric intramolecular α-cyclopropanation of aldehydes using a donor/acceptor carbene mimetic. *Nat. Commun.* 6:10041 doi: 10.1038/ncomms10041 (2015).

## Supplementary Material

Supplementary Information Supplementary Figures 1-109 and Supplementary Methods

Supplementary Data 1 X-ray CIF file for compound 2b (CCDC1405229)

Supplementary Data 2 X-ray CIF file for compound 4c (CCDC1405230)

Supplementary Data 3 X-ray CIF file for compound 8c (CCDC1405231)

## Figures and Tables

**Figure 1:**
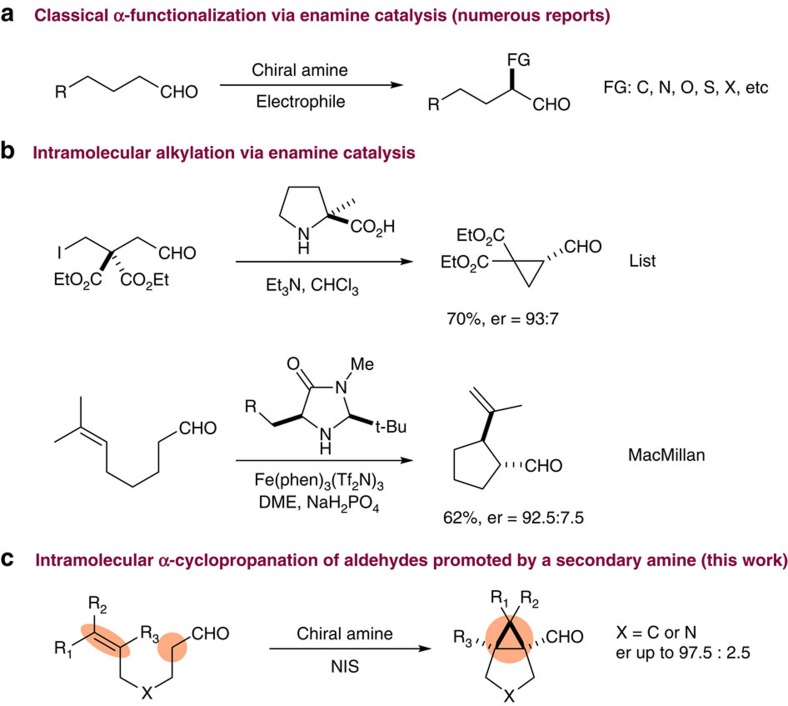
α-Functionalization of aldehydes via enamine organocatalysis. (**a**) A number of reactions dealing with enantioselective α-functionalization of aldehydes have been reported using the classical enamine organocatalysis. (**b**) Two representative reports of intramolecular α-alkylation of aldehydes are shown. (**c**) Our strategy for direct α-cyclopropanation of aldehydes. DME, dimethoxyethane; FG, functional group; NIS, N-Iodosuccinimide; phen, 1, 10-phenanthroline; Tf_2_N, (CF_3_SO_2_)_2_N.

**Table 1:**
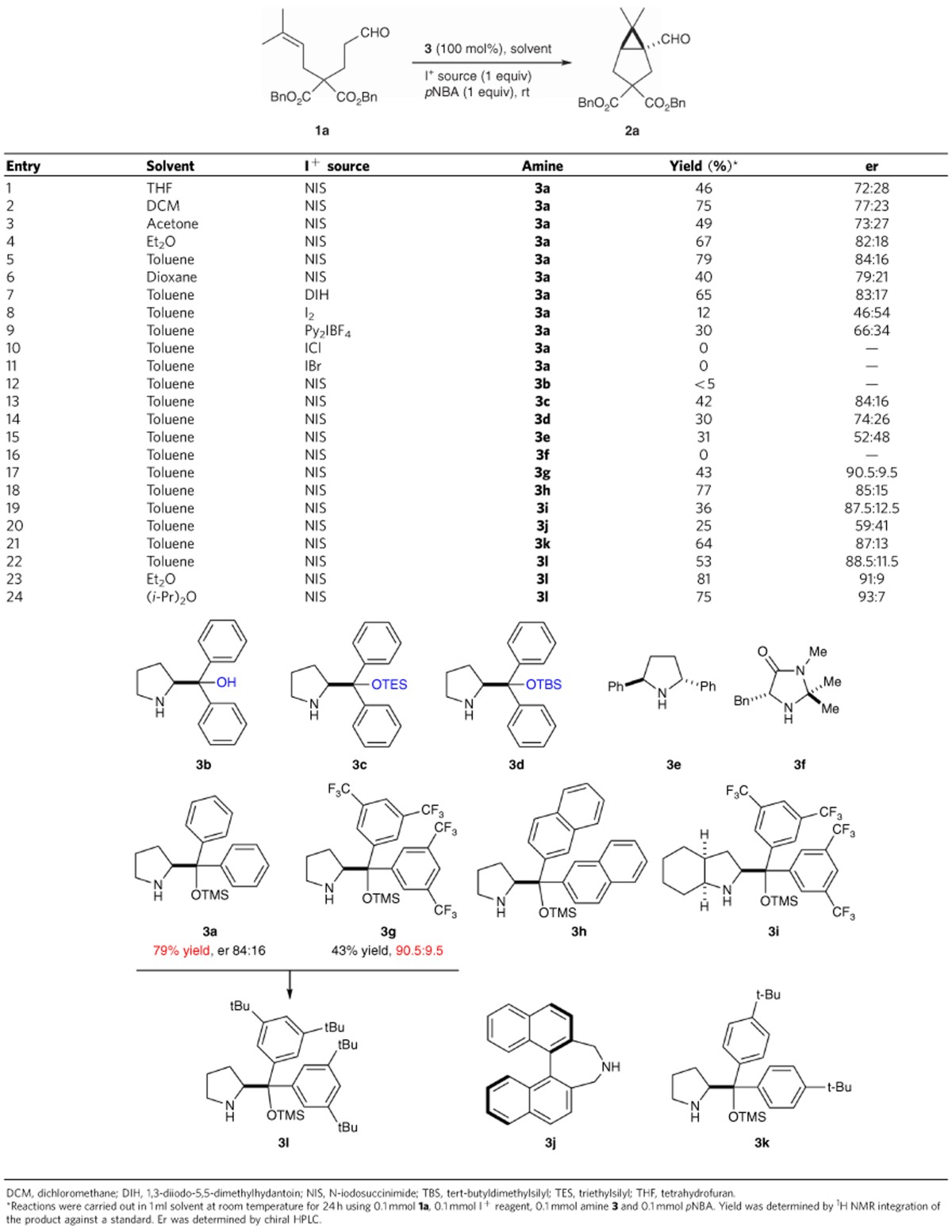
Condition optimization.

**Table 2:**
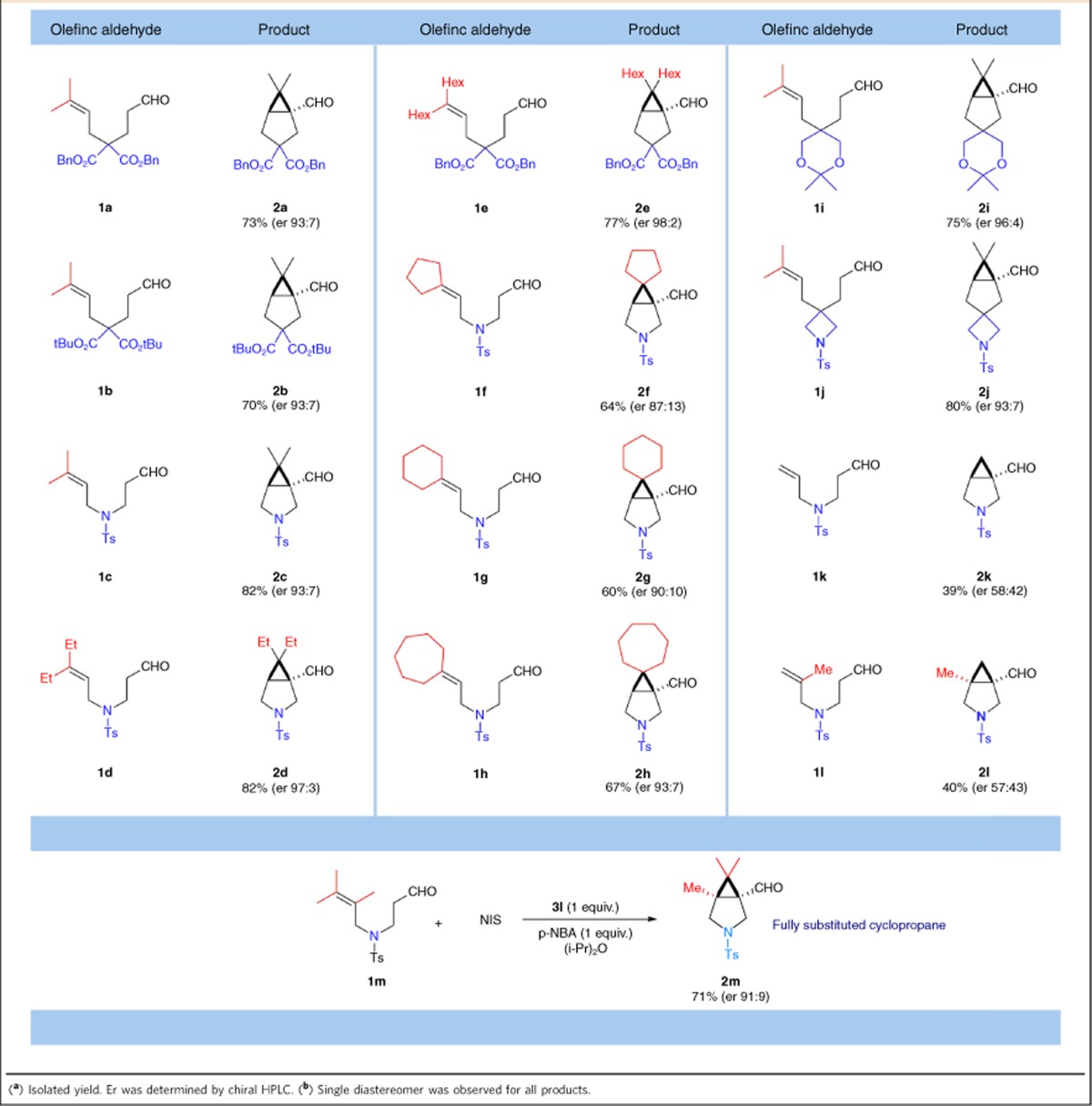
Substrate scope for the **α**-cyclopropanation reaction^a,b^.
