# Supplementary material for: Asymmetric intramolecular α-cyclopropanation of aldehydes using a donor/acceptor carbene mimetic
Source: Nat Commun. 2015 Dec 8;6:10041. doi: 10.1038/ncomms10041 (PMC4686670; doi:10.1038/ncomms10041)
Supplement: Supplementary Information — Supplementary Figures 1-109 and Supplementary Methods [file ncomms10041-s1.pdf]

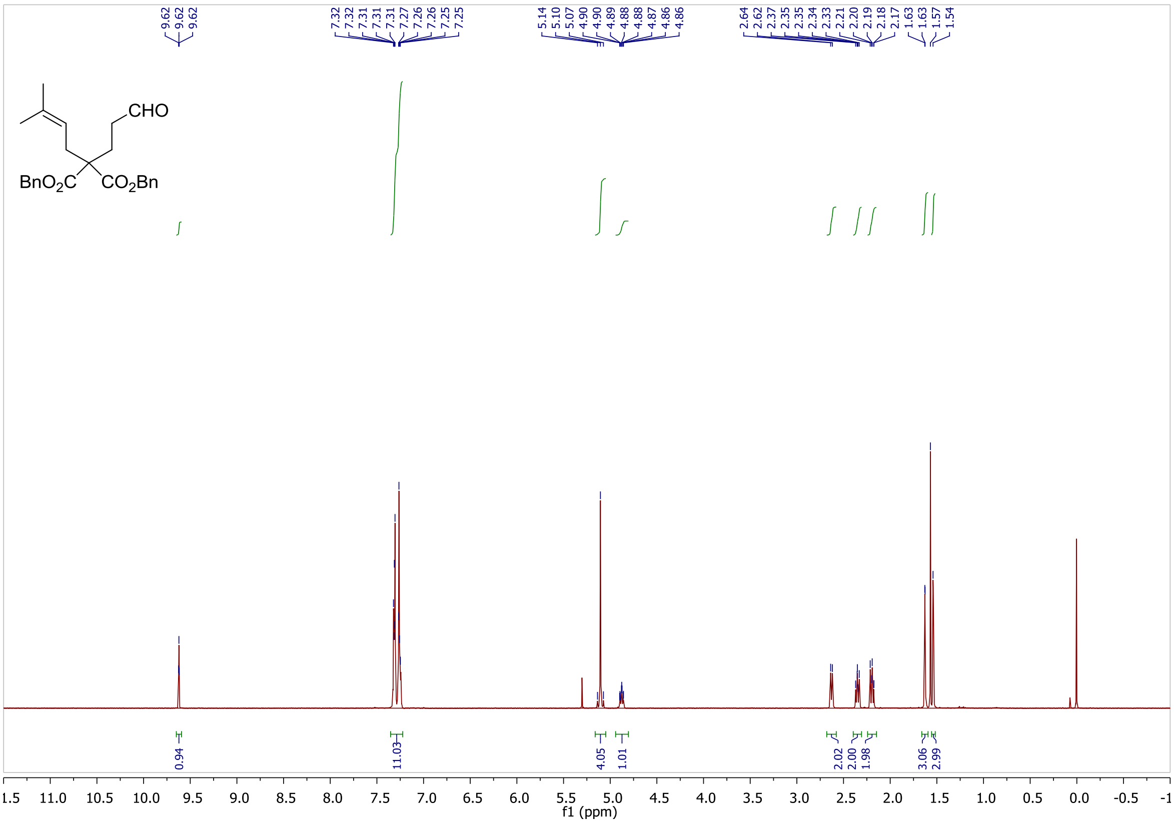

Supplementary Figure 1. ^{1}H NMR of the **1a** (400 MHz, $CDCl_3$)

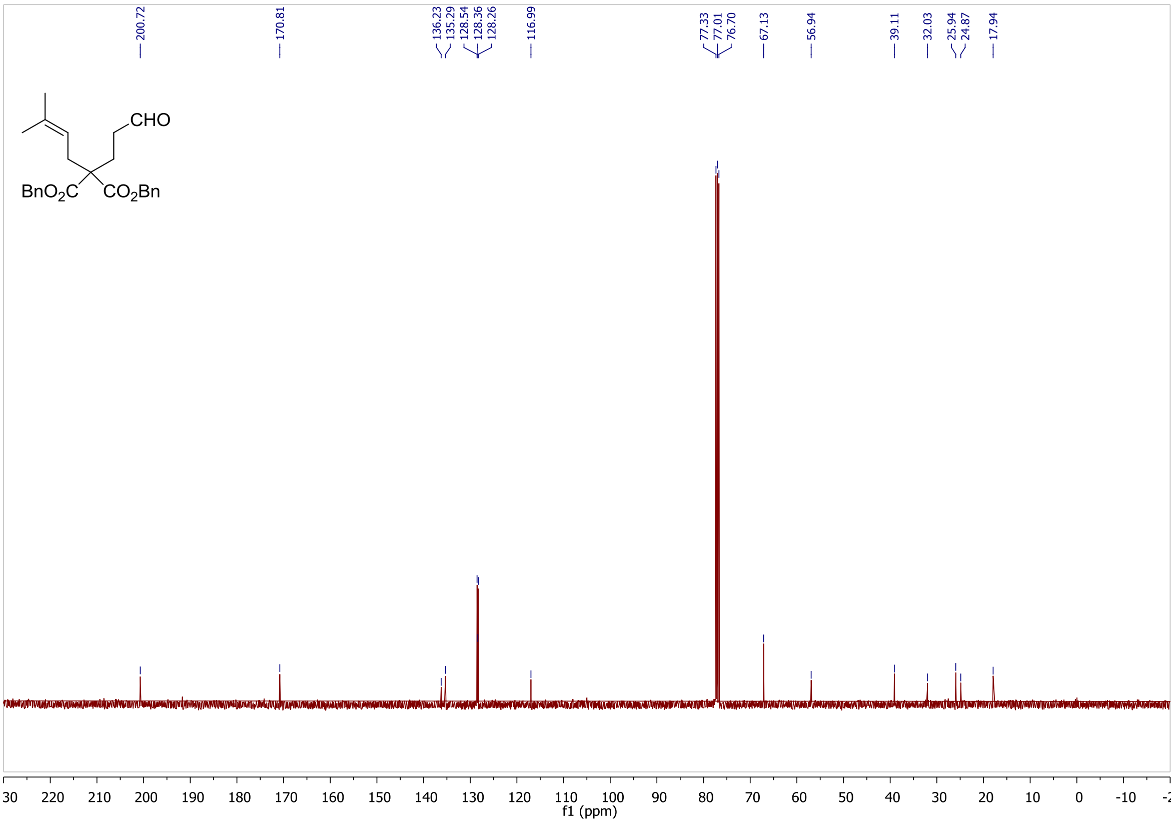

Supplementary Figure 2. ^{13}C NMR of the **1a** (101 MHz, $CDCl_3$)

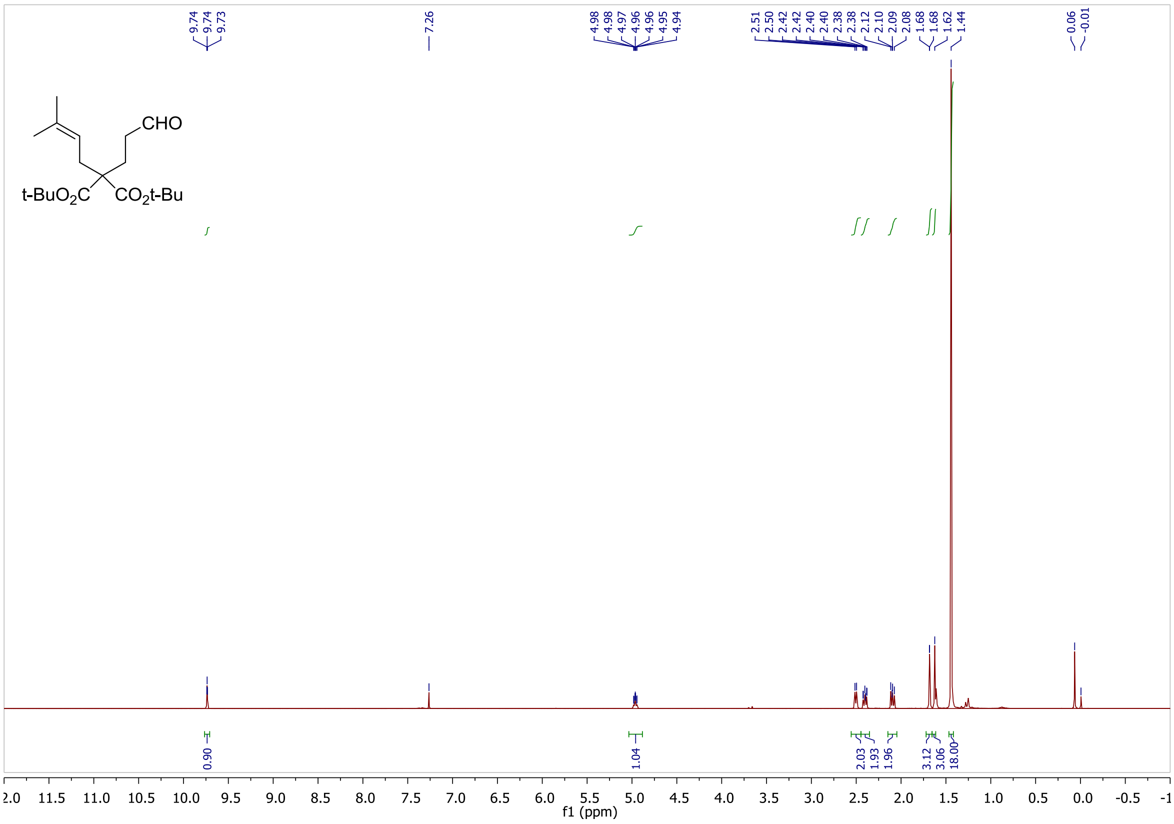

Supplementary Figure 3. ^{1}H NMR of the **1b** (400 MHz, $CDCl_3$)

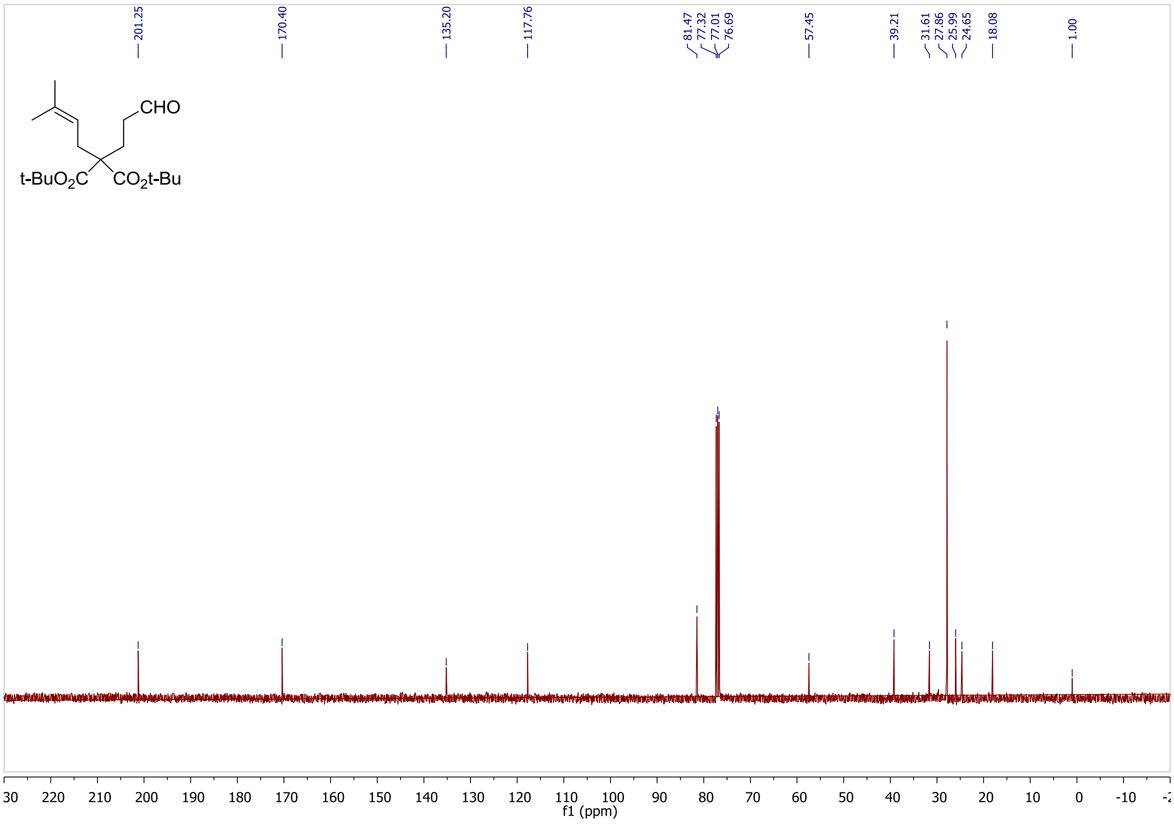

Supplementary Figure 4. ^{13}C NMR of the **1b** (101 MHz, $CDCl_3$)

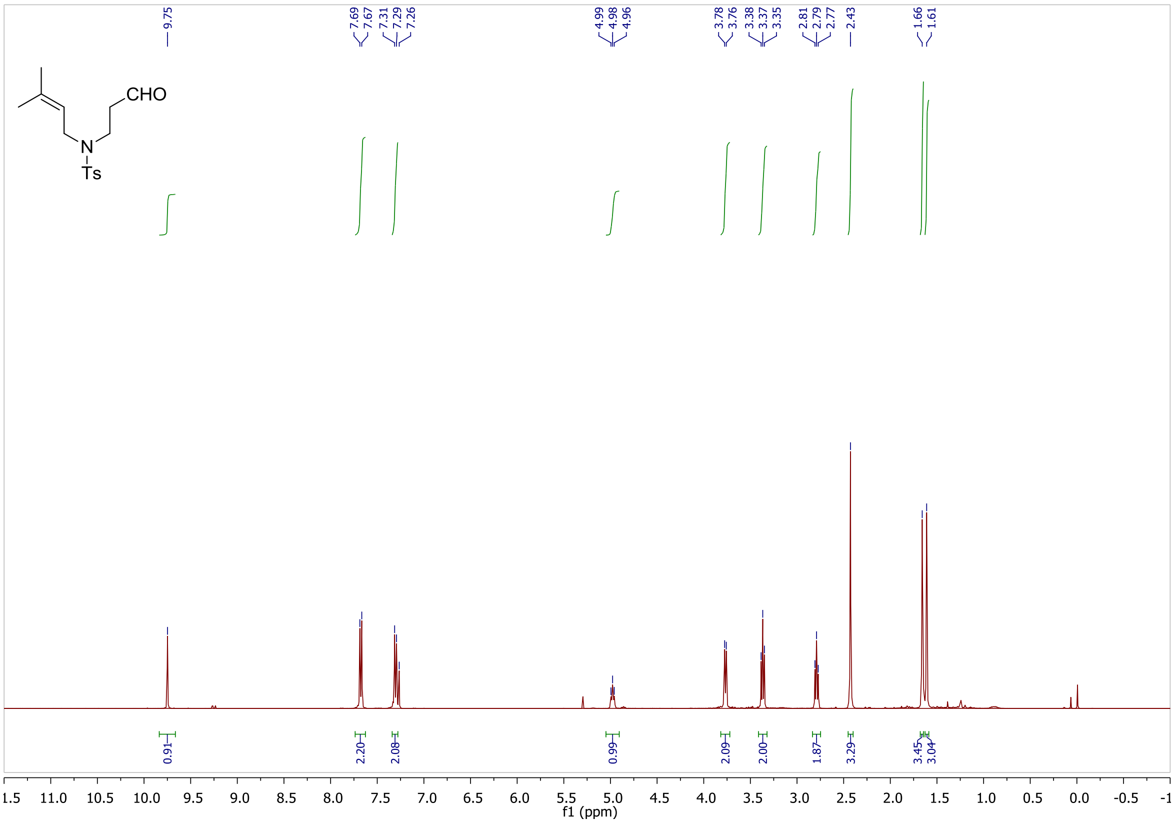

Supplementary Figure 5. ^{1}H NMR of the **1c** (400 MHz, $CDCl_3$)

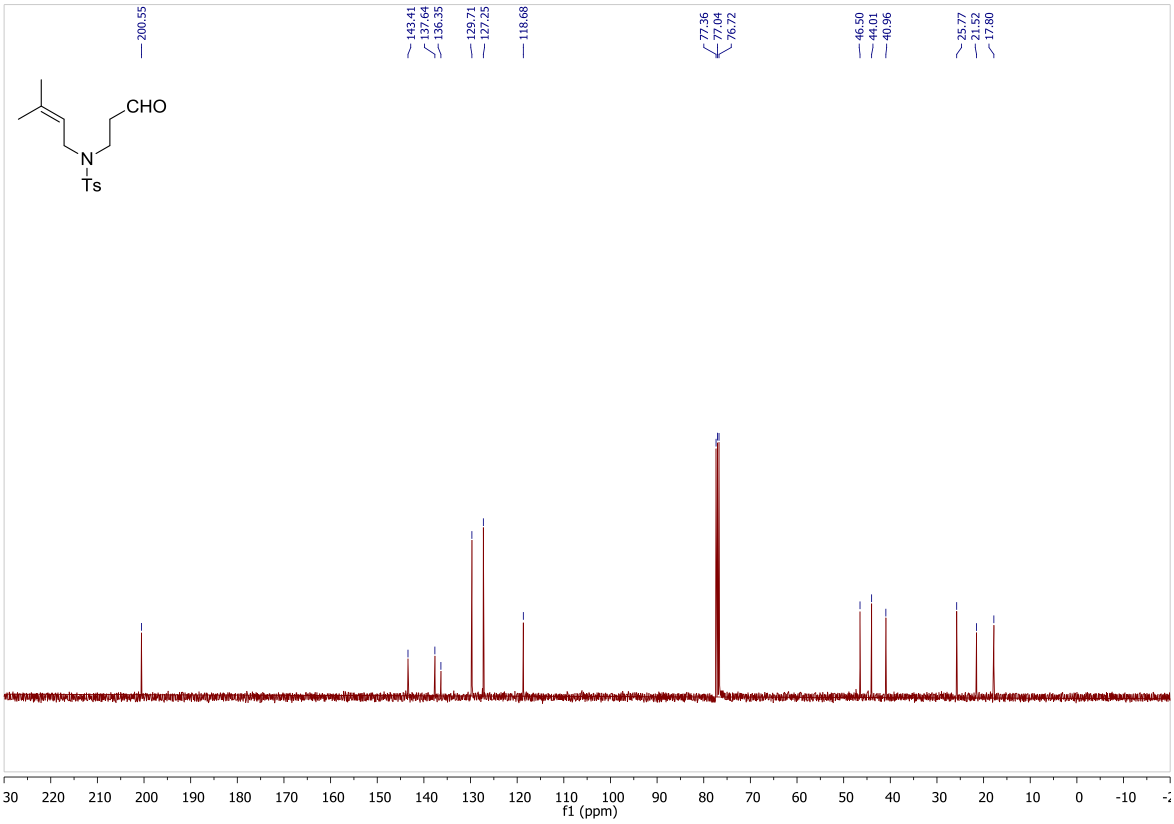

Supplementary Figure 6. ^{13}C NMR of the **1c** (101 MHz, $CDCl_3$)

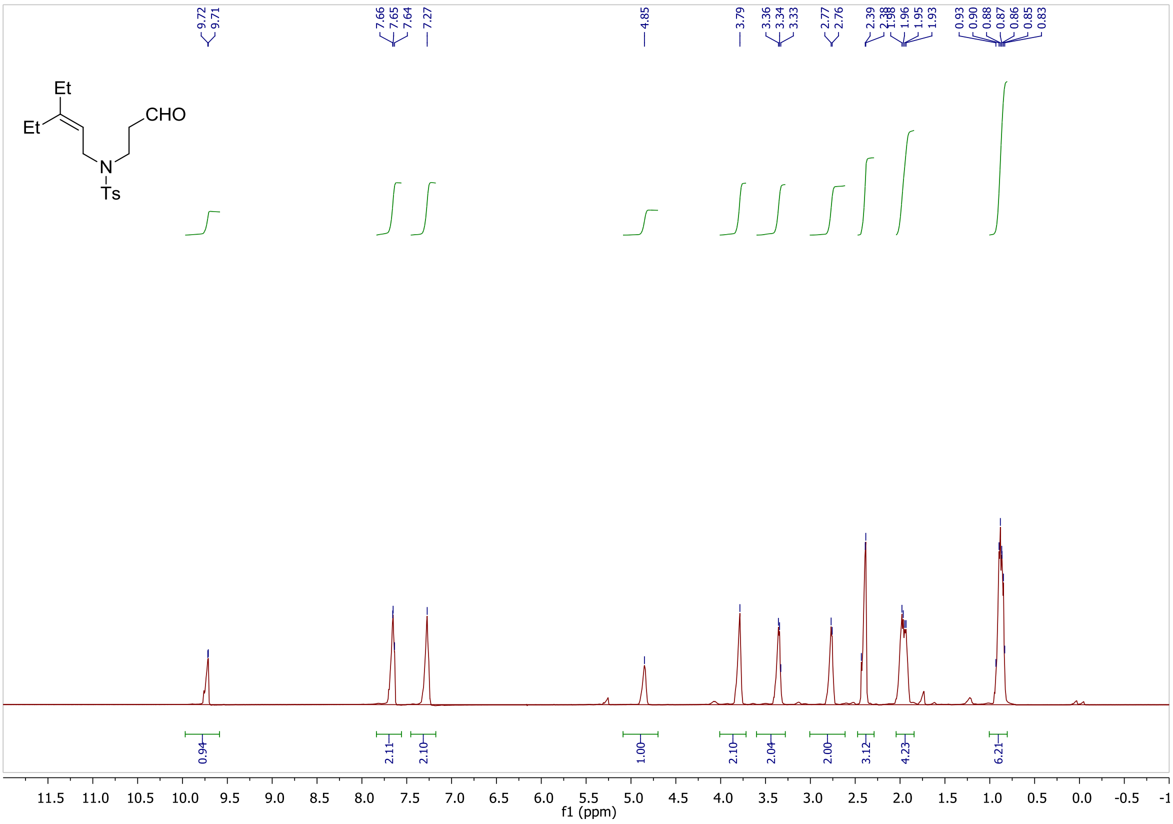

Supplementary Figure 7. ^{1}H NMR of the **1d** (500 MHz, $CDCl_3$)

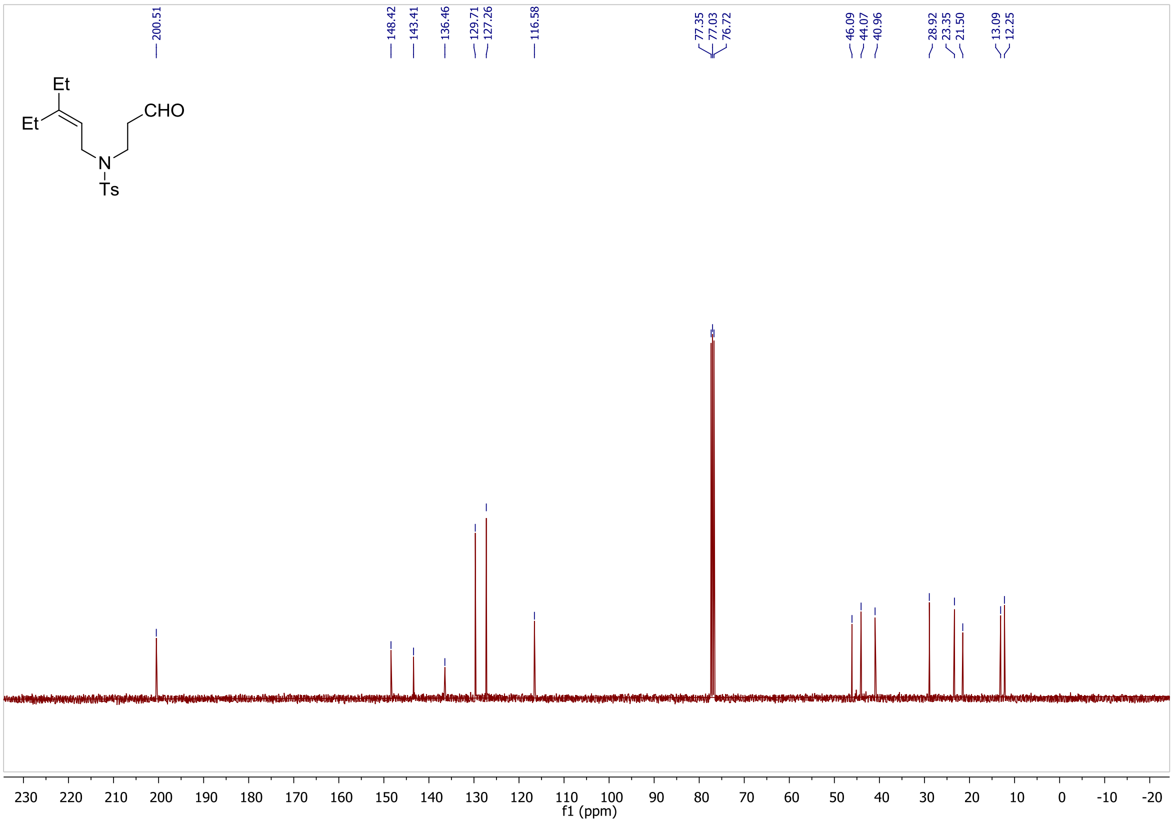

Supplementary Figure 8. ^{13}C NMR of the **1d** (126 MHz, $CDCl_3$)

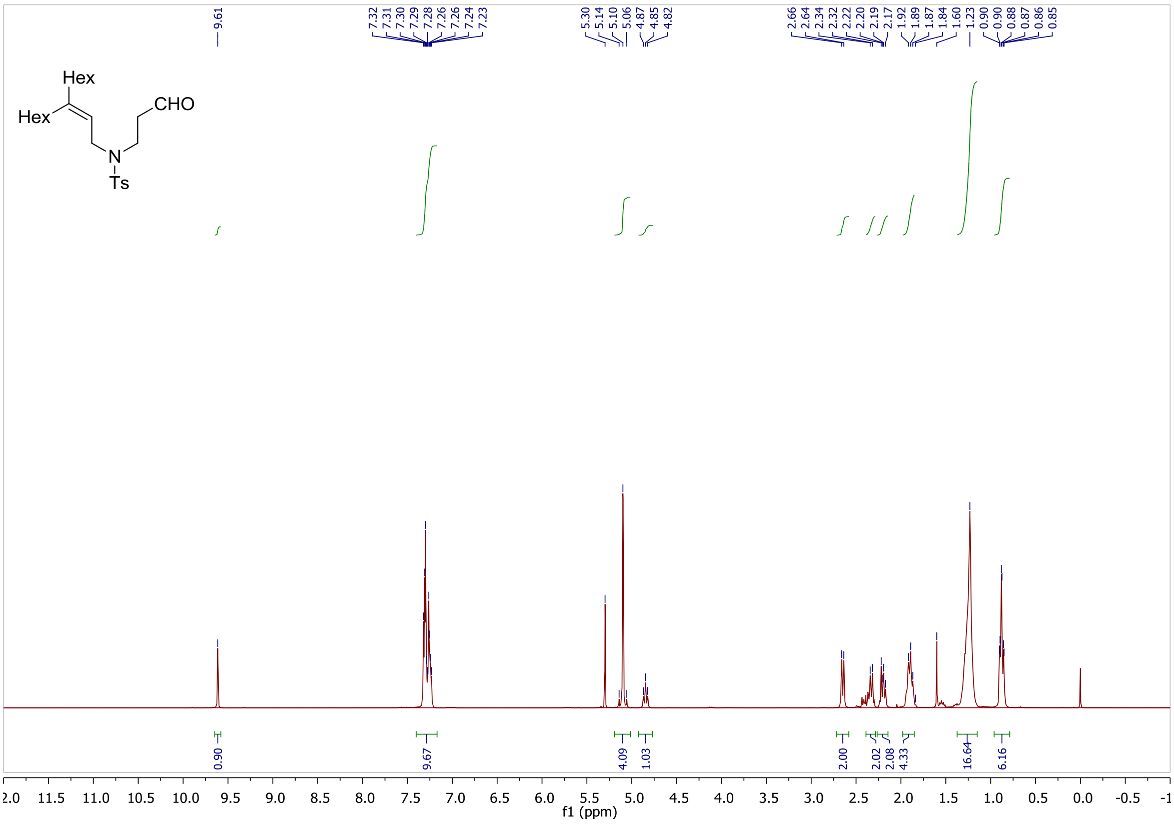

Supplementary Figure 9. ^{1}H NMR of the **1e** (300 MHz, $CDCl_3$)

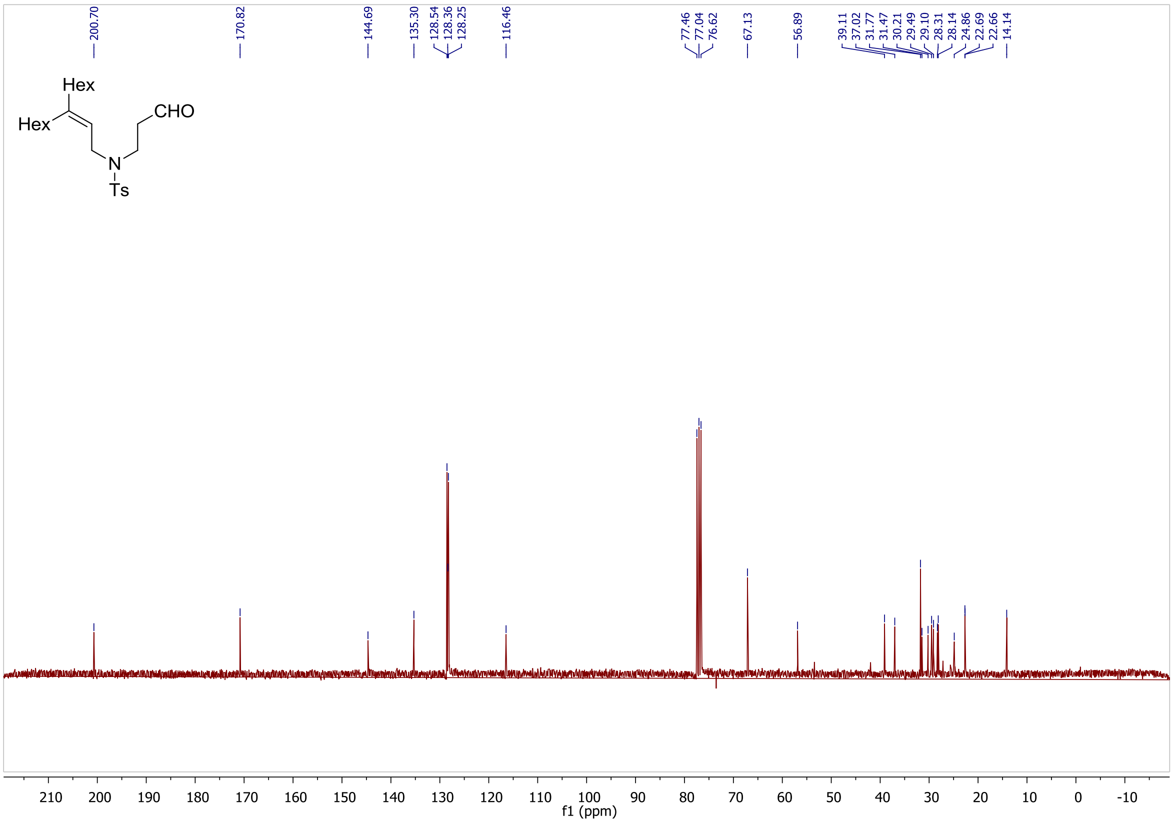

Supplementary Figure 10. ^{13}C NMR of the **1e** (75 MHz, $CDCl_3$)

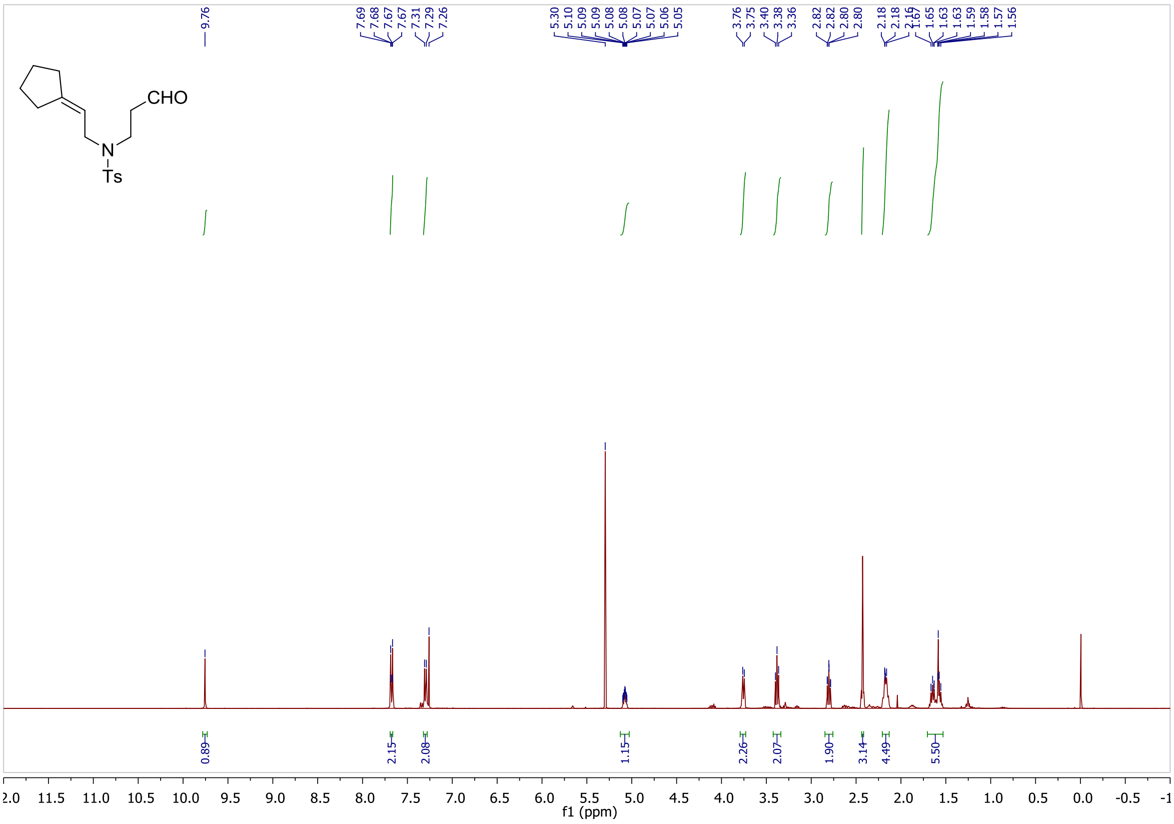

Supplementary Figure 11. ^{1}H NMR of the **1f** (400 MHz, $CDCl_3$)

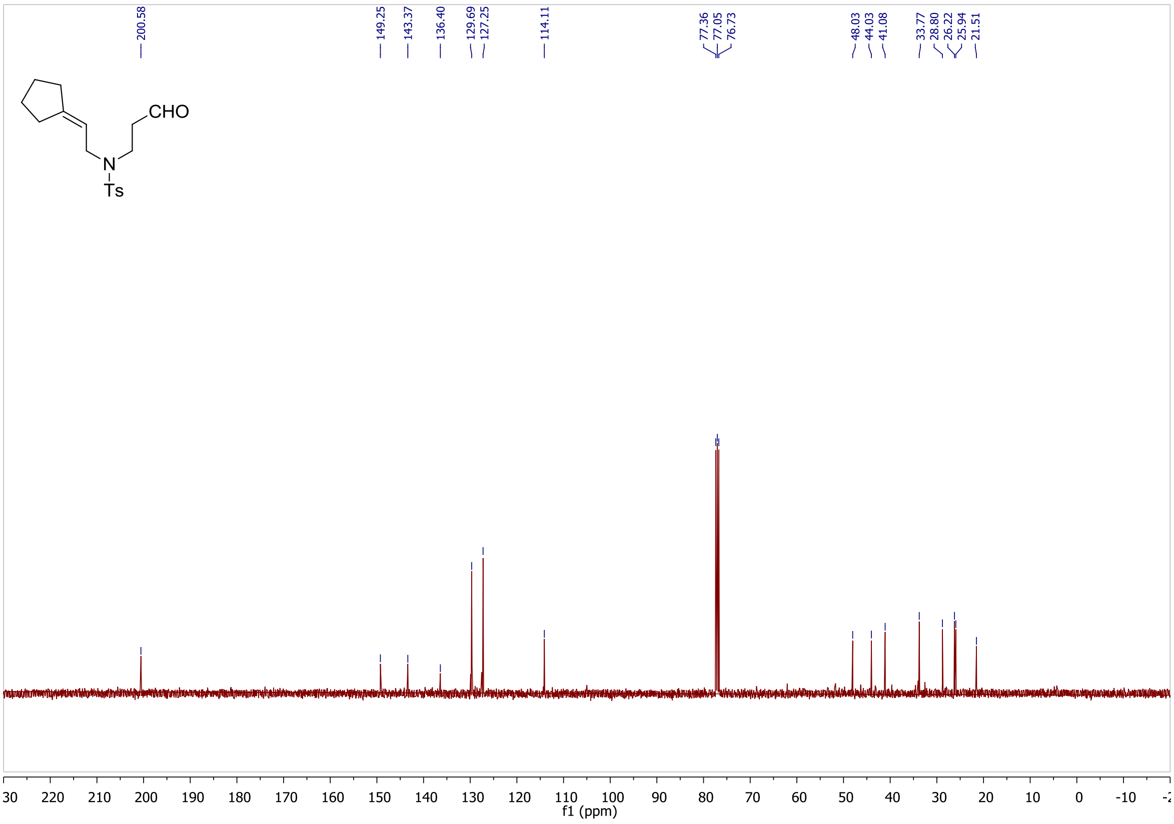

Supplementary Figure 12. ^{13}C NMR of the **1f** (101 MHz, $CDCl_3$)

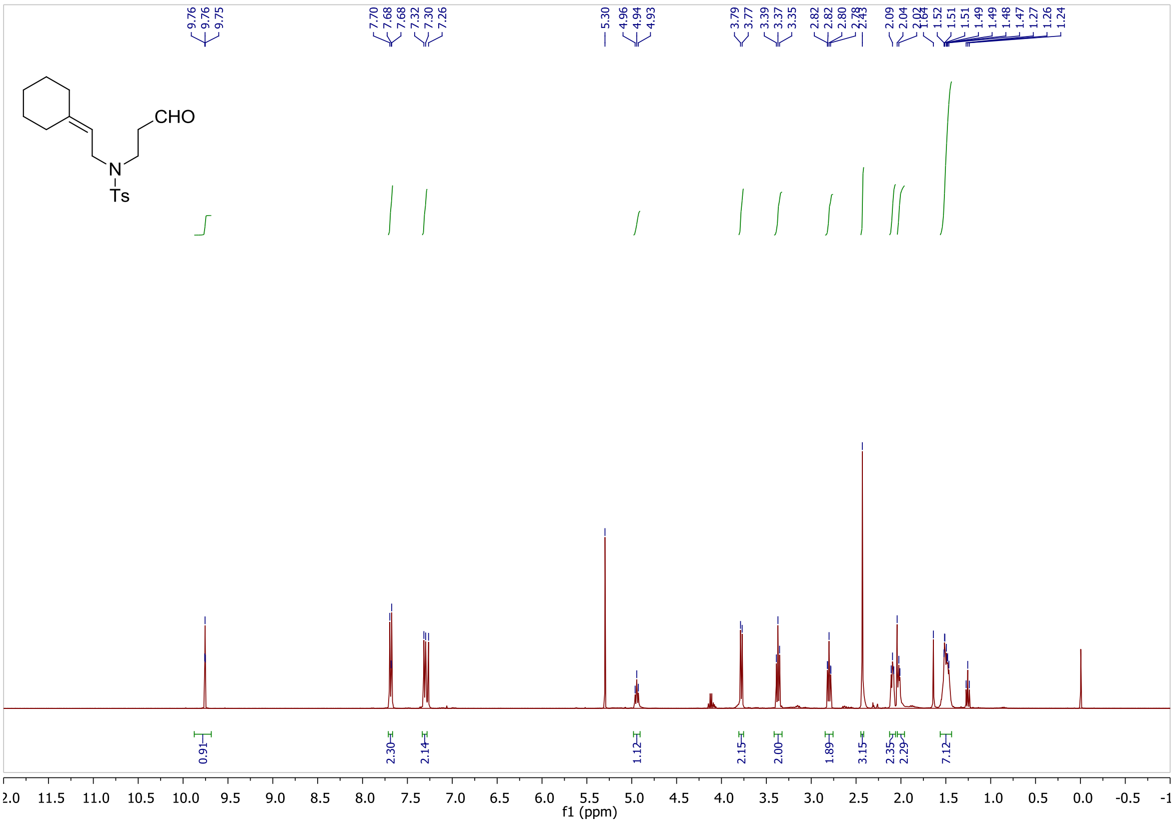

Supplementary Figure 13. ^{1}H NMR of the **1g** (400 MHz, $CDCl_3$)

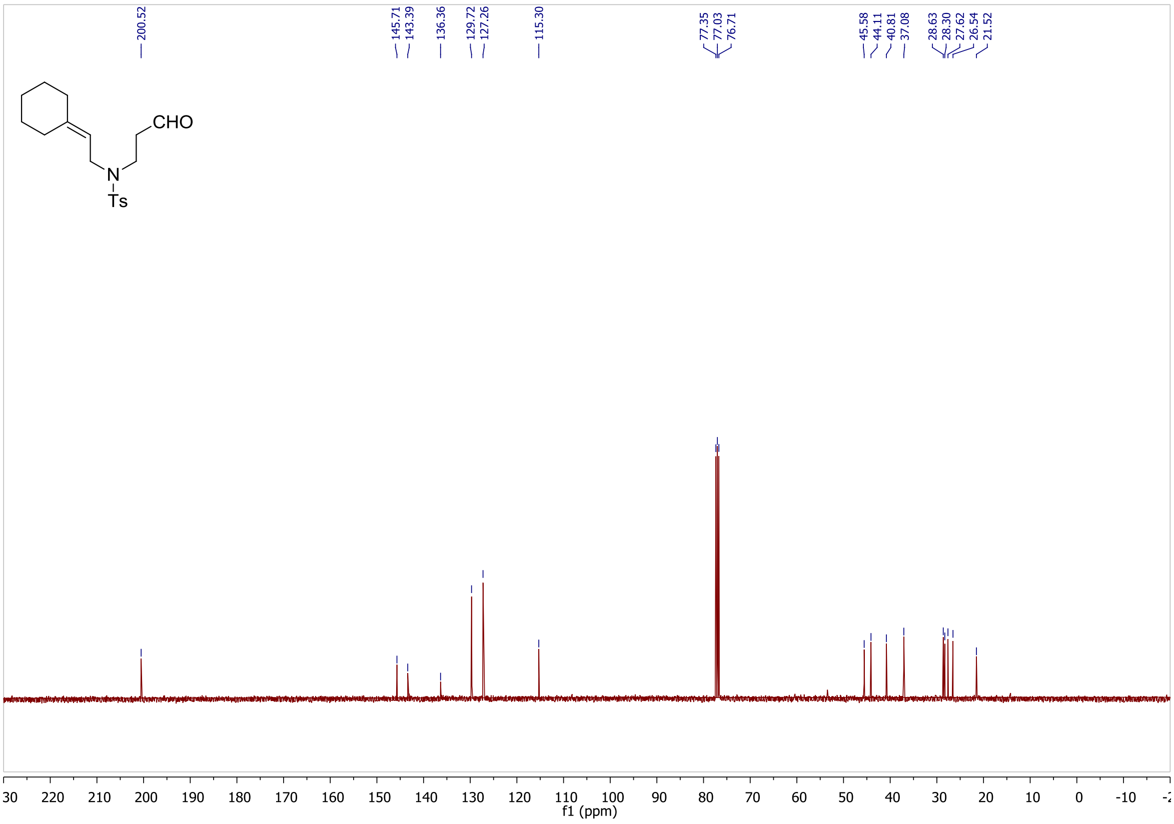

Supplementary Figure 14. ^{13}C NMR of the **1g** (101 MHz, $CDCl_3$)

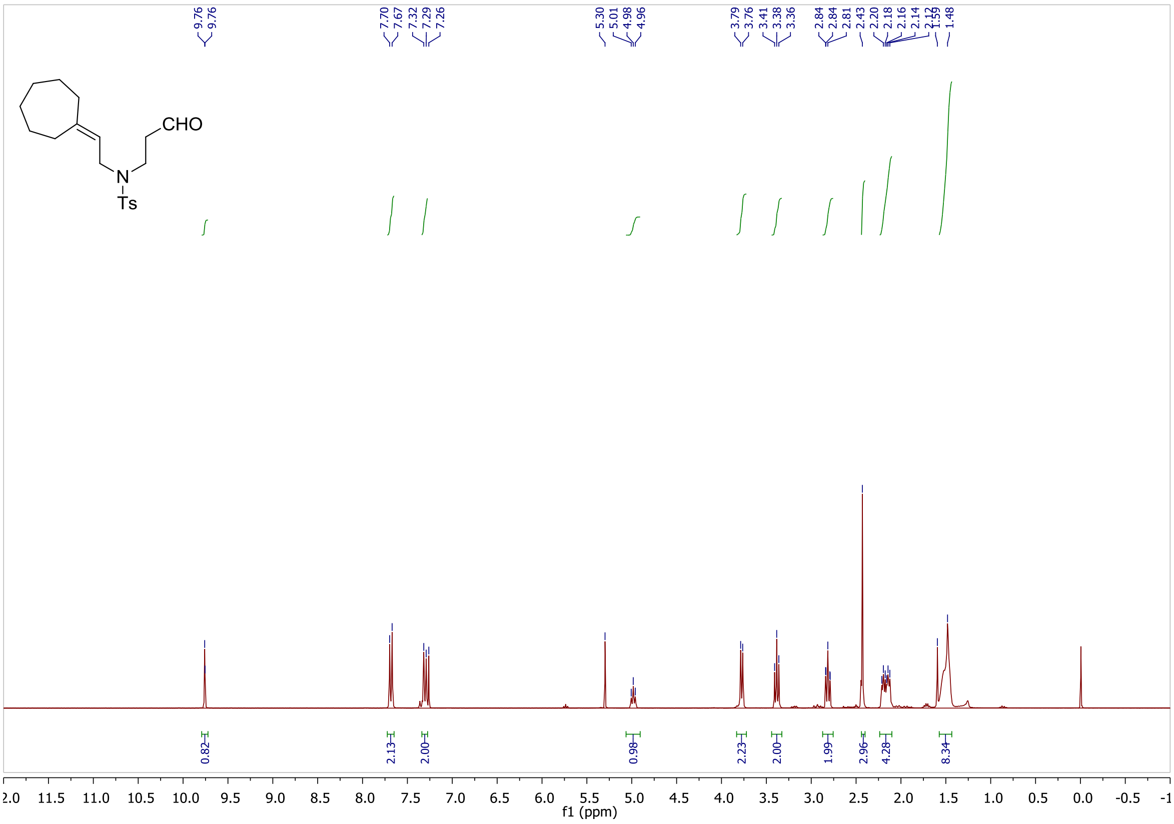

Supplementary Figure 15. ^{1}H NMR of the **1h** (400 MHz, $CDCl_3$)

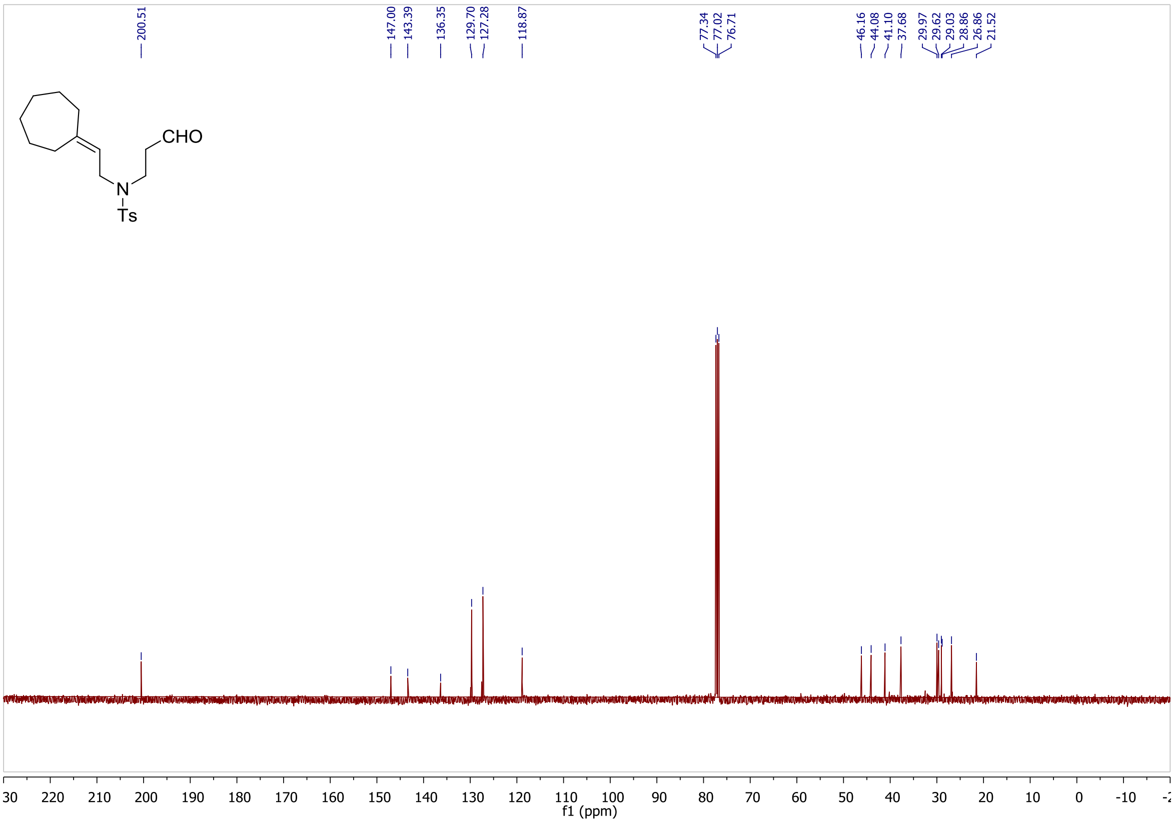

Supplementary Figure 16. ^{13}C NMR of the **1h** (101 MHz, $CDCl_3$)

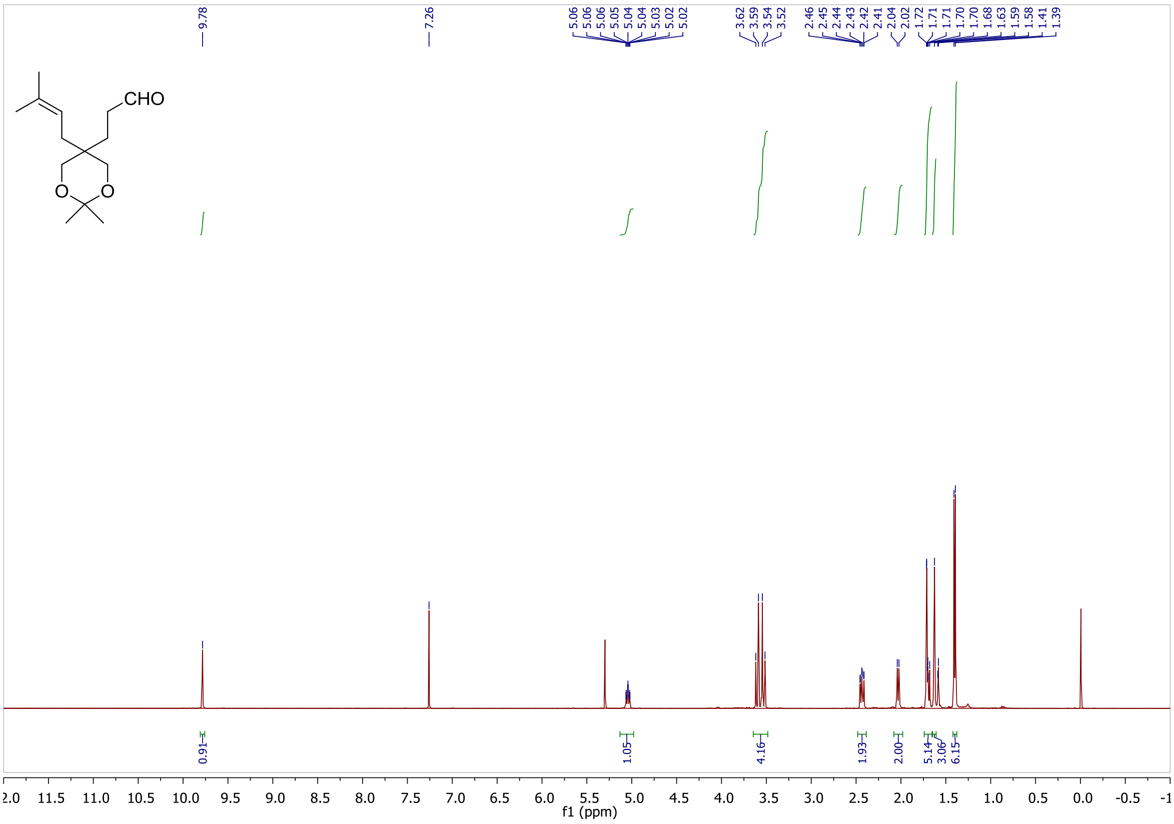

Supplementary Figure 17. ^{1}H NMR of the **1i** (400 MHz, $CDCl_3$)

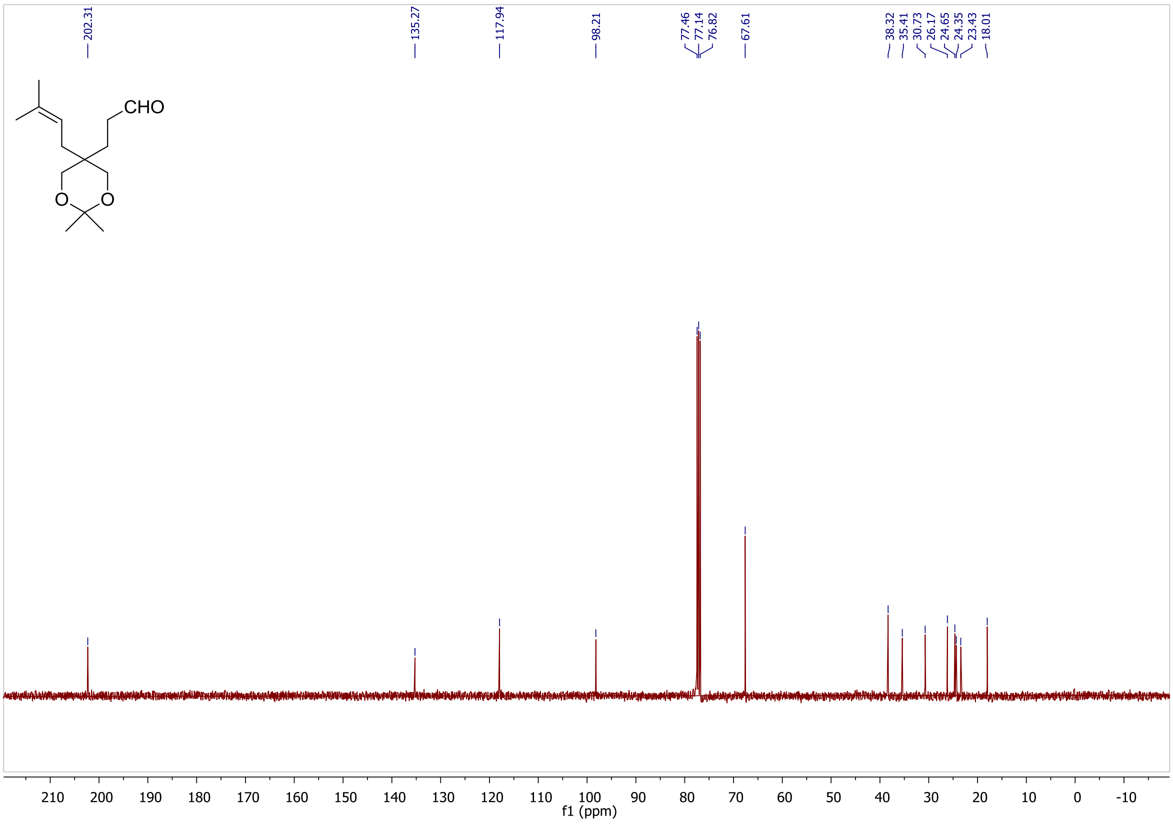

Supplementary Figure 18. ^{13}C NMR of the **1i** (101 MHz, $CDCl_3$)

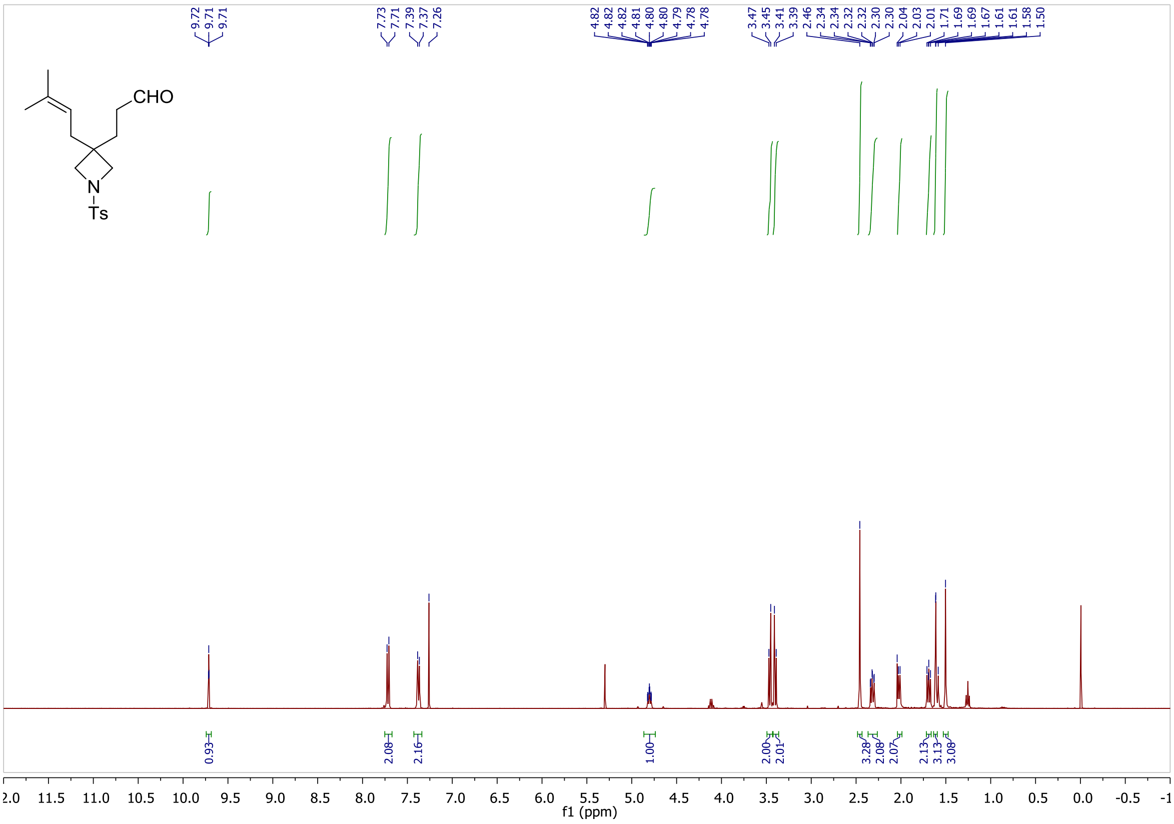

Supplementary Figure 19. ^{1}H NMR of the **1j** (400 MHz, $CDCl_3$)

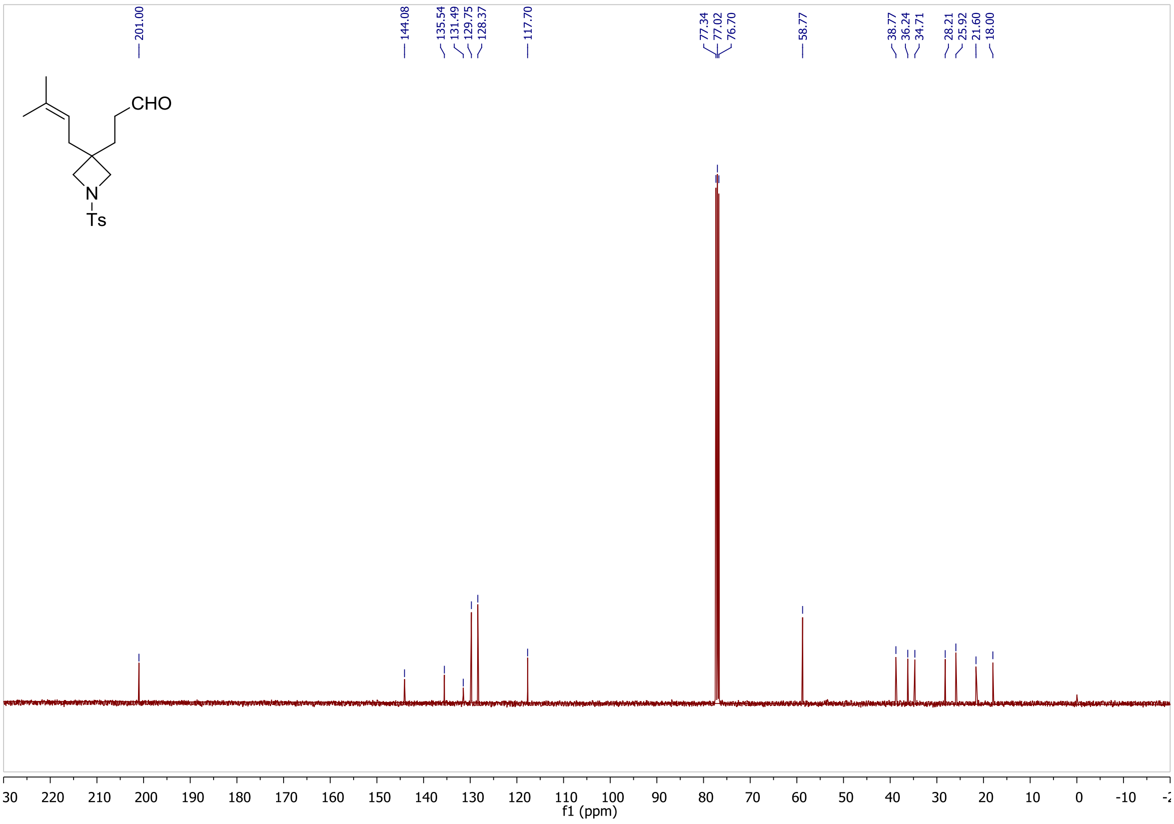

Supplementary Figure 20. ^{13}C NMR of the **1j** (101 MHz, $CDCl_3$)

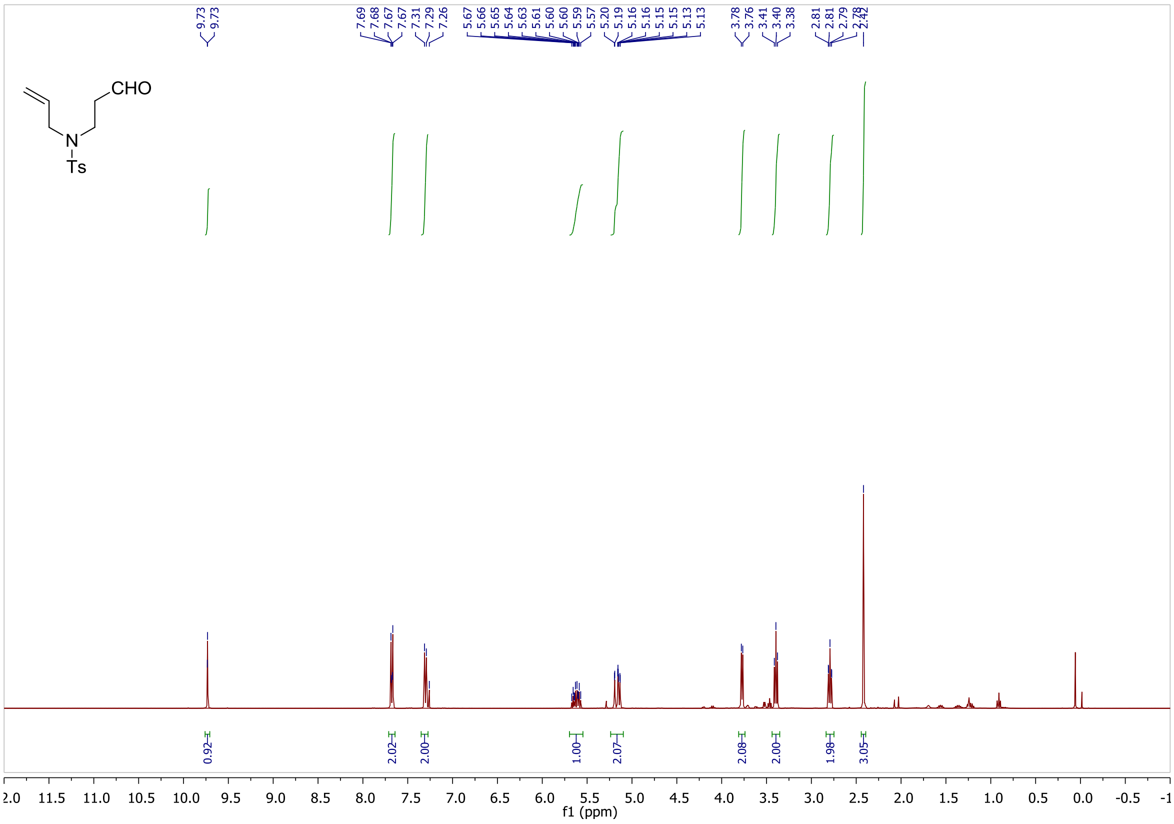

Supplementary Figure 21. ^{1}H NMR of the **1k** (400 MHz, $CDCl_3$)

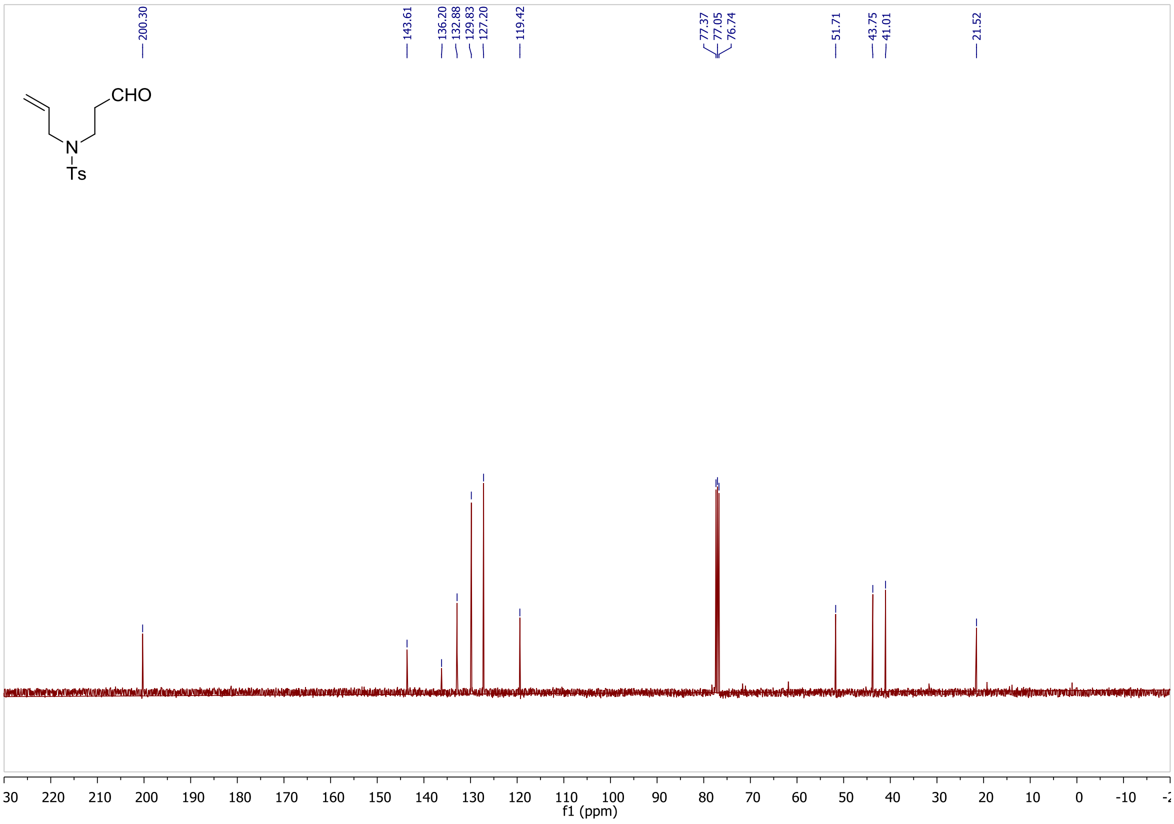

Supplementary Figure 22. ^{13}C NMR of the **1k** (101 MHz, $CDCl_3$)

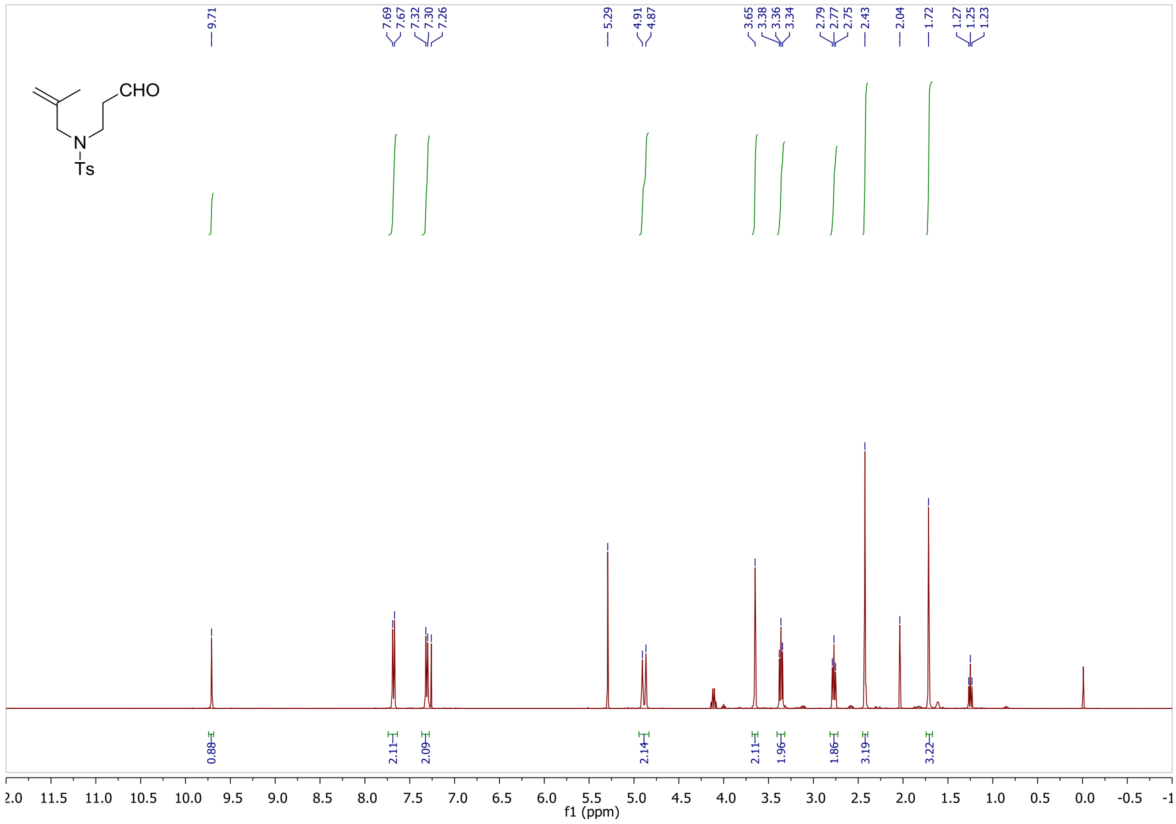

Supplementary Figure 23. ^{1}H NMR of the **1l** (400 MHz, $CDCl_3$)

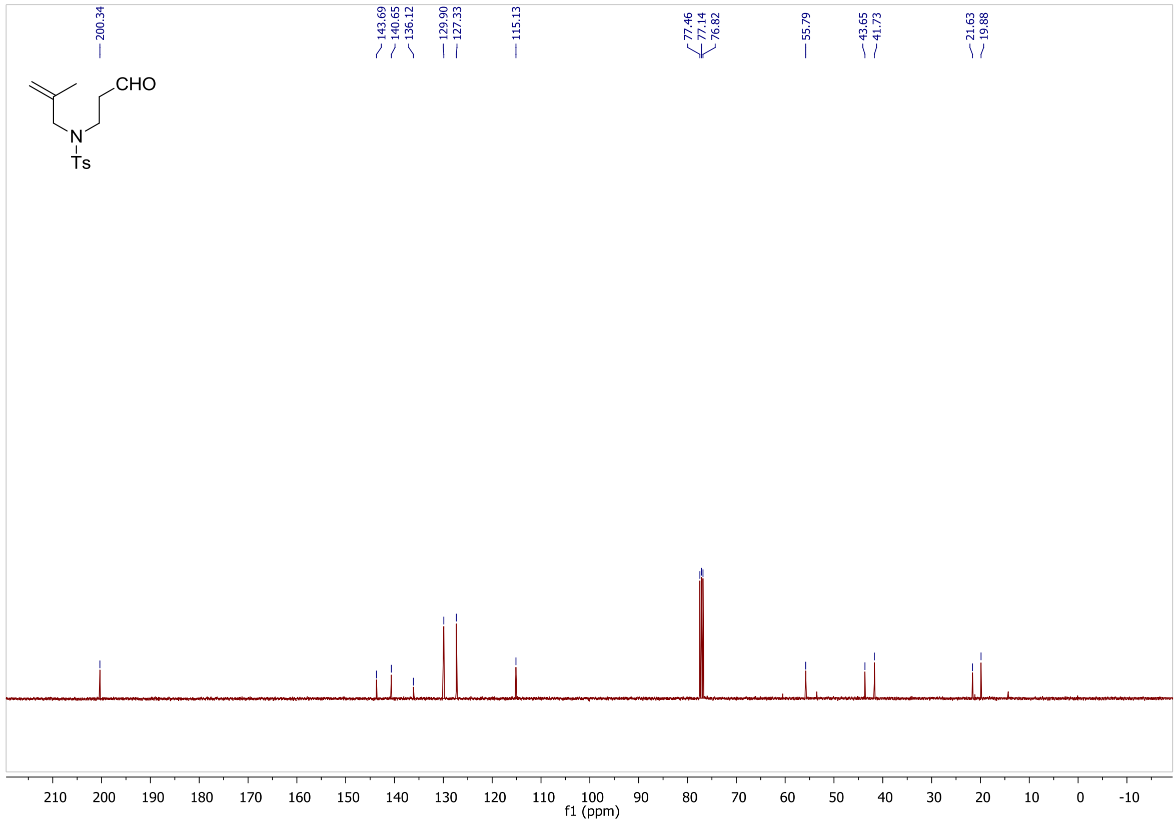

Supplementary Figure 24. ^{13}C NMR of the **1l** (101 MHz, $CDCl_3$)

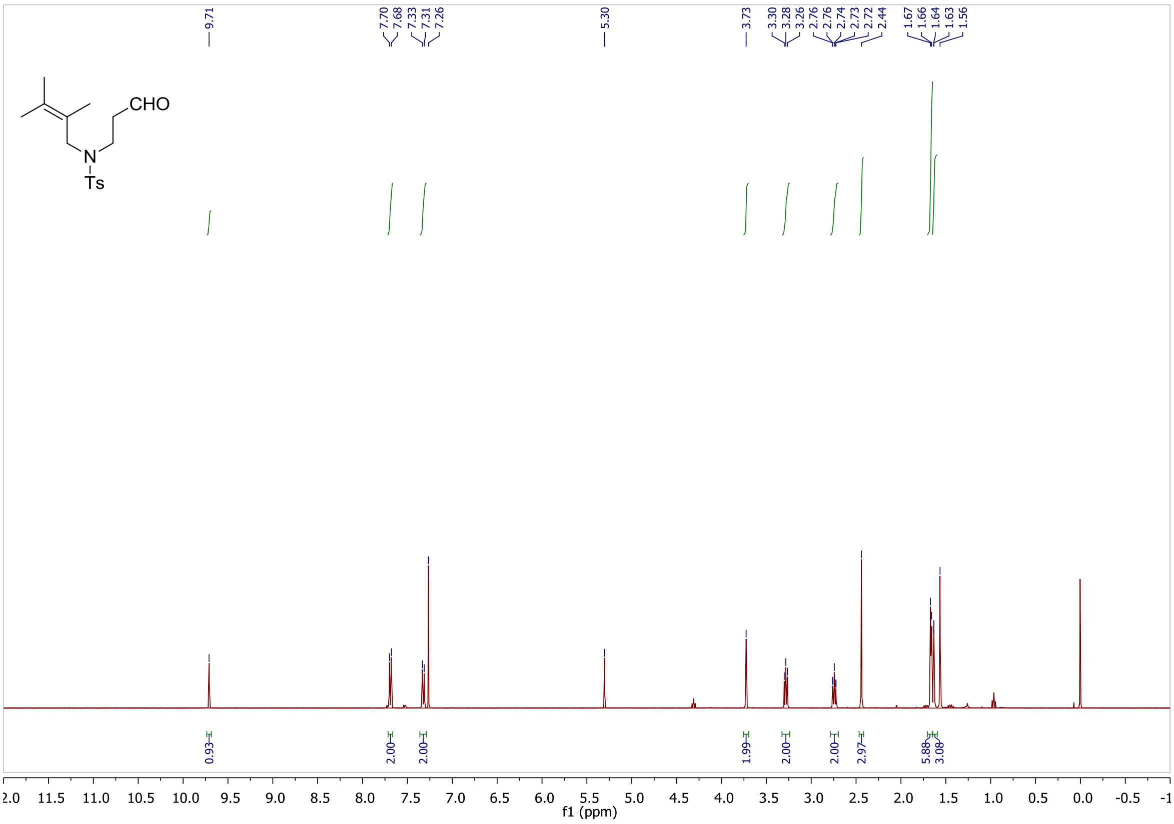

Supplementary Figure 25. ^{1}H NMR of the **1m** (400 MHz, $CDCl_3$)

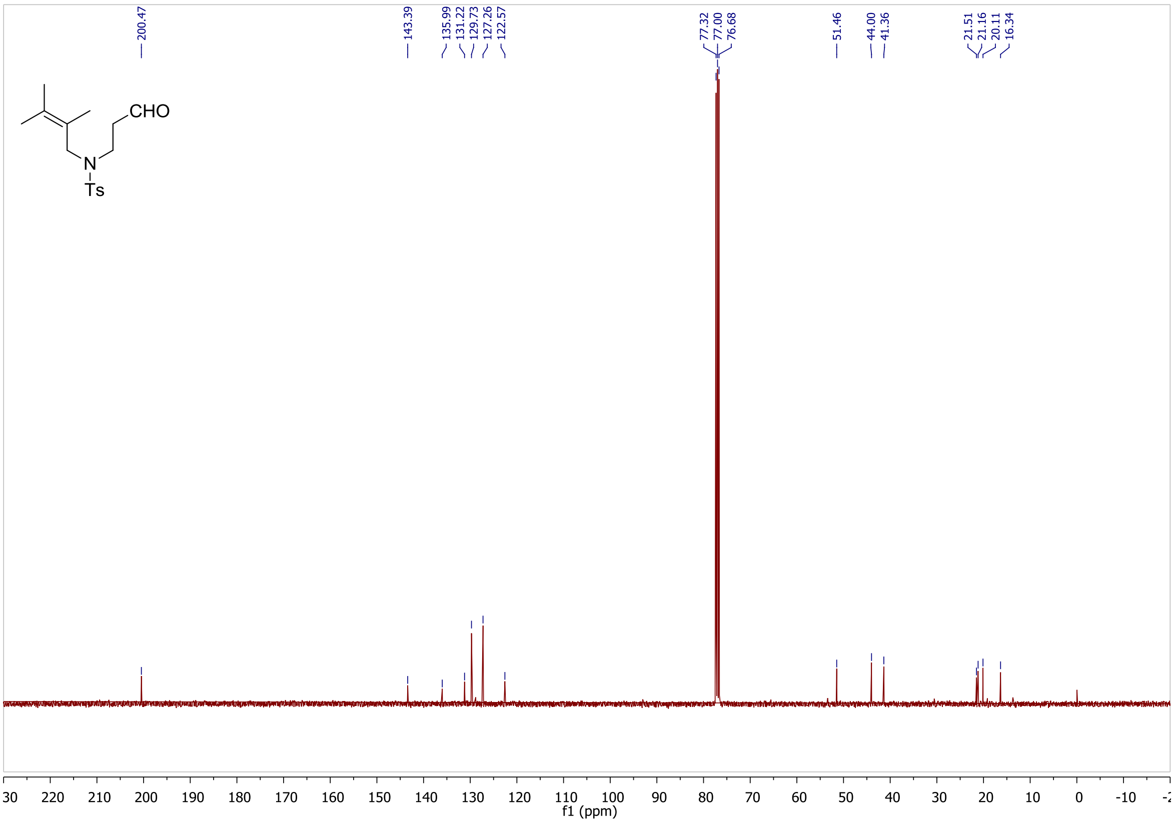

Supplementary Figure 26. ^{13}C NMR of the **1m** (101 MHz, $CDCl_3$)

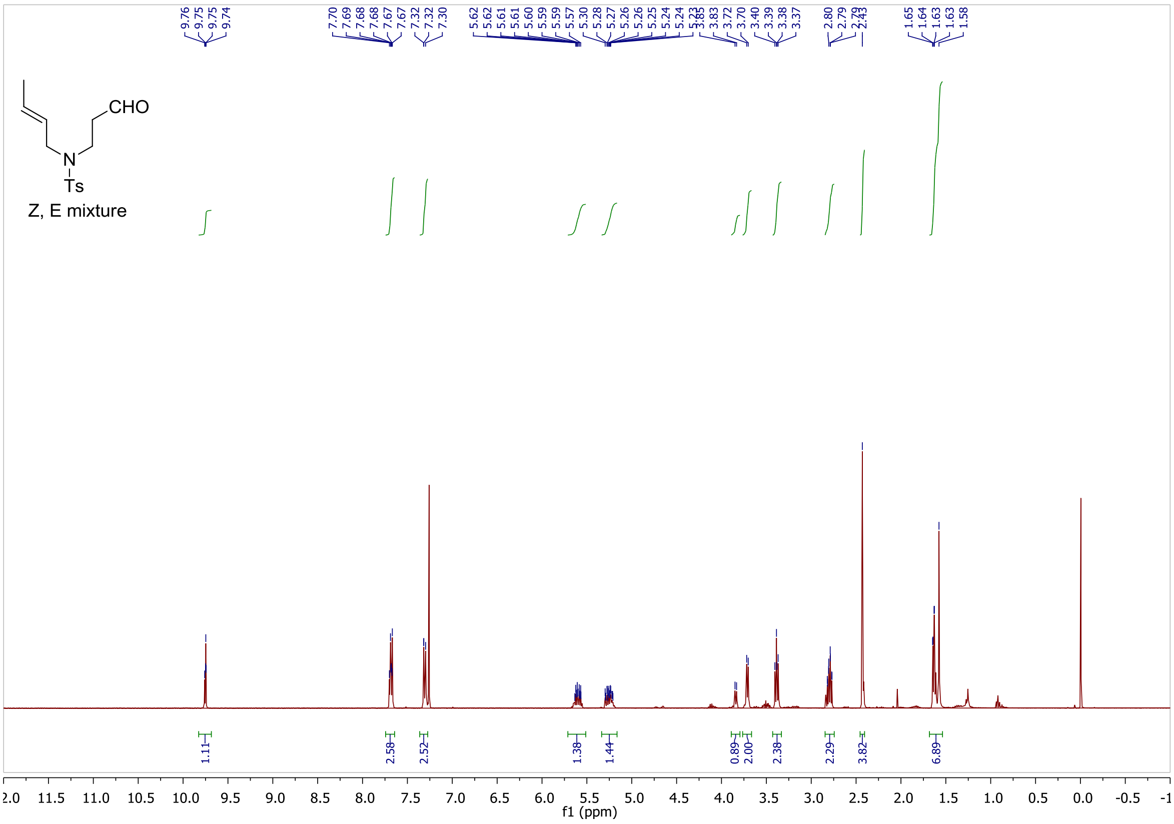

Supplementary Figure 27. ^{1}H NMR of the **1n** (400 MHz, $CDCl_3$)

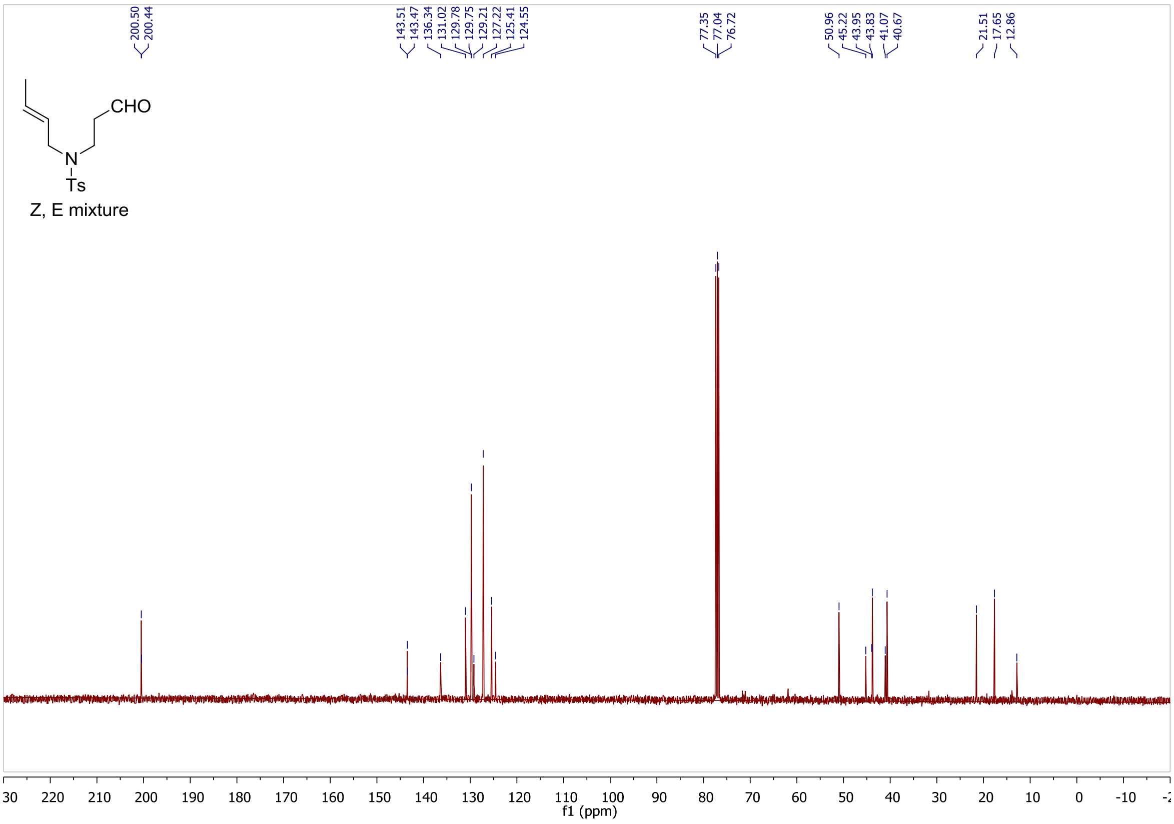

Supplementary Figure 28. ^{13}C NMR of the **1n** (101 MHz, $CDCl_3$)

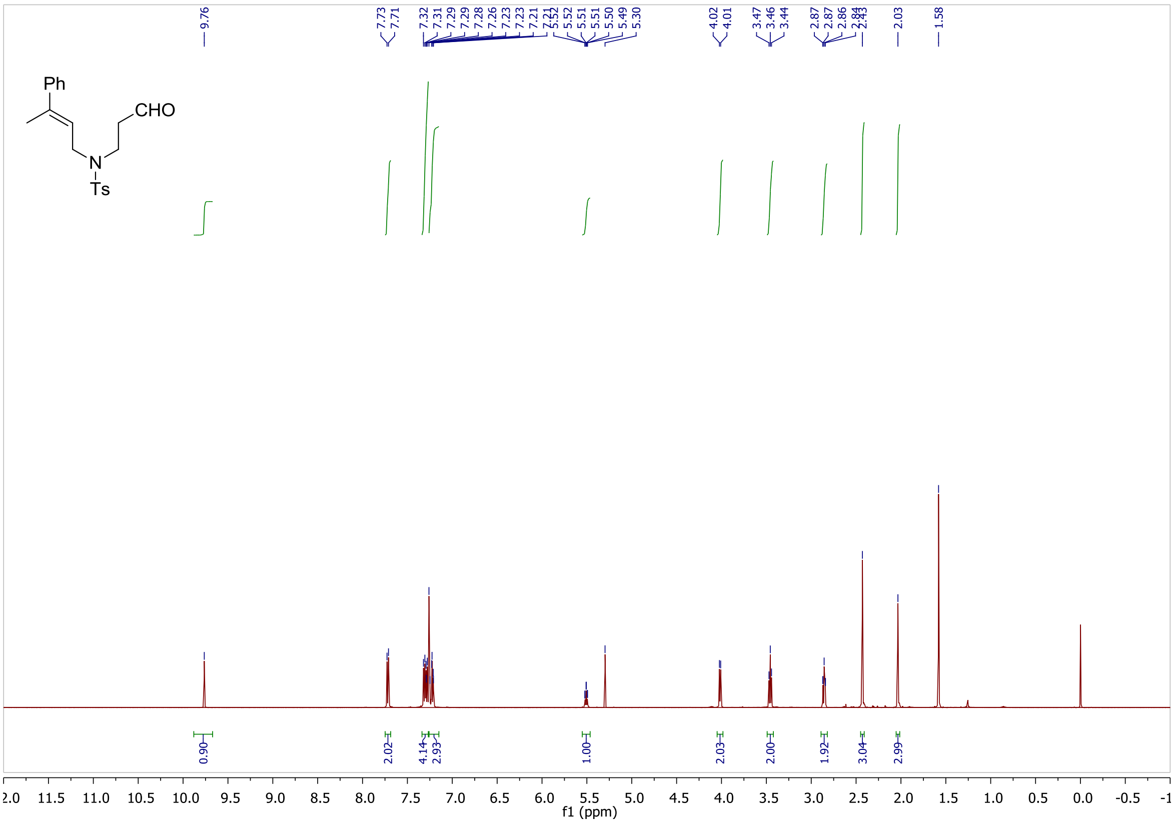

Supplementary Figure 29. ^{1}H NMR of the **1o** (500 MHz, $CDCl_3$)

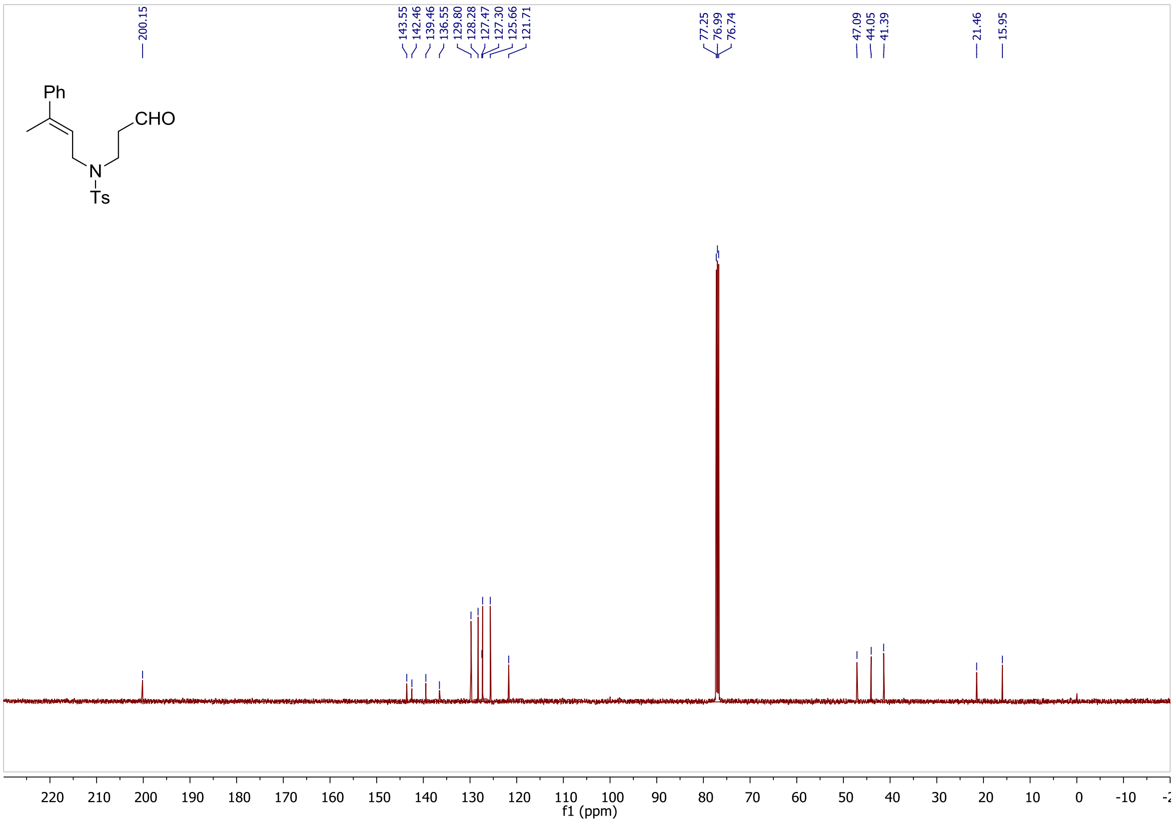

Supplementary Figure 30. ^{13}C NMR of the **1o** (126 MHz, $CDCl_3$)

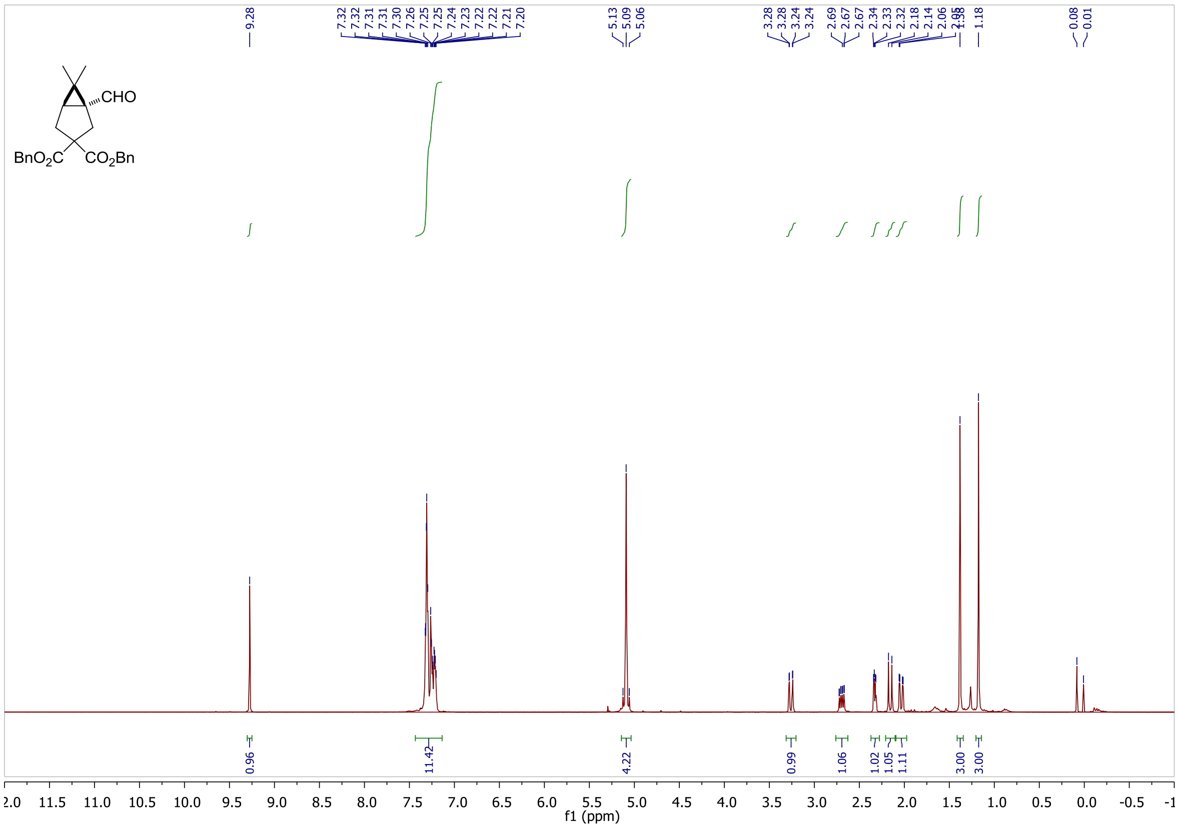

Supplementary Figure 31. ^{1}H NMR of the **2a** (400 MHz, $CDCl_3$)

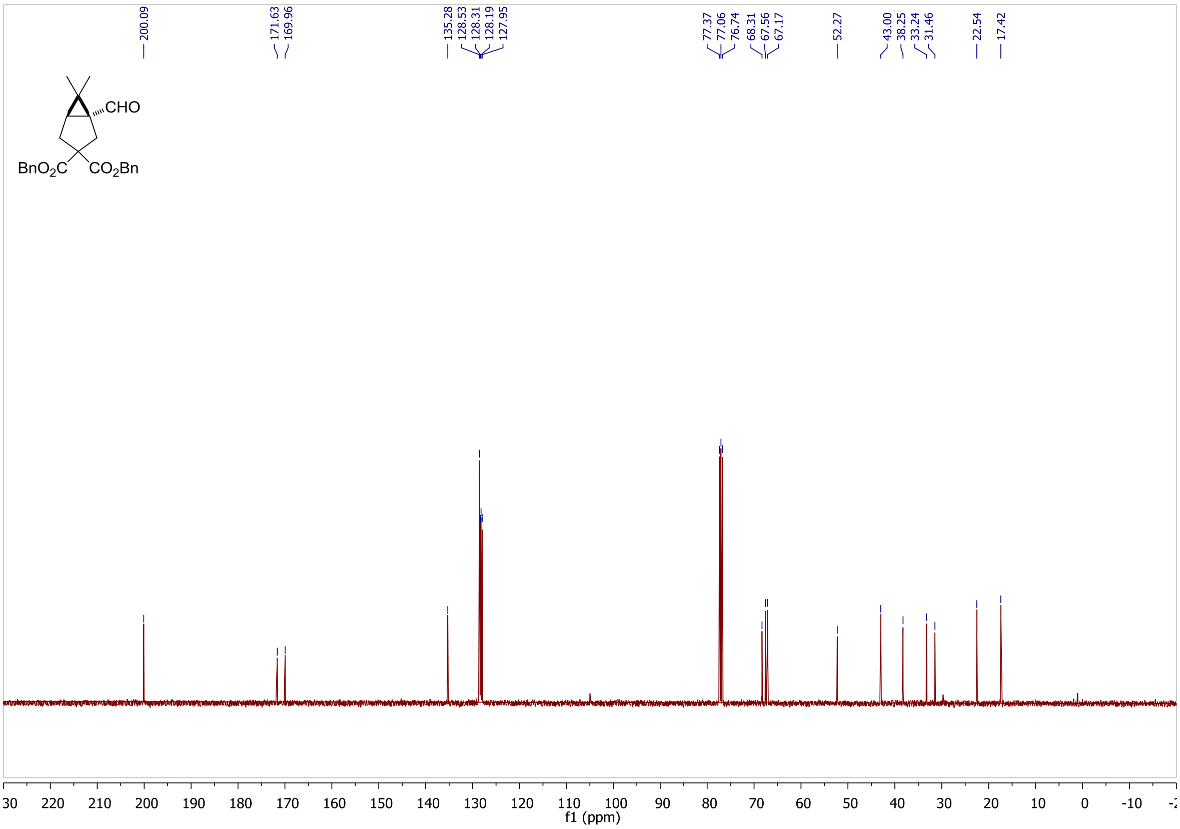

Supplementary Figure 32. ^{13}C NMR of the **2a** (101 MHz, $CDCl_3$)

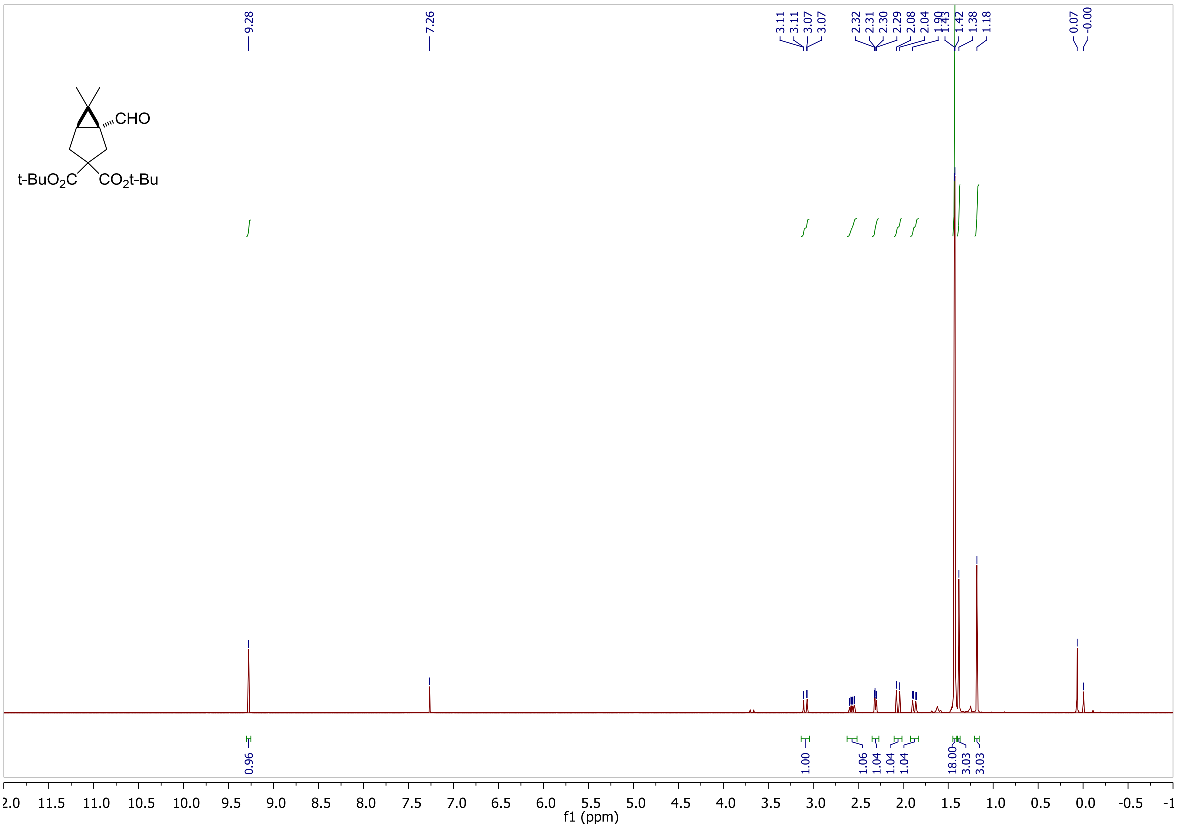

Supplementary Figure 33. ^{1}H NMR of the **2b** (400 MHz, $CDCl_3$)

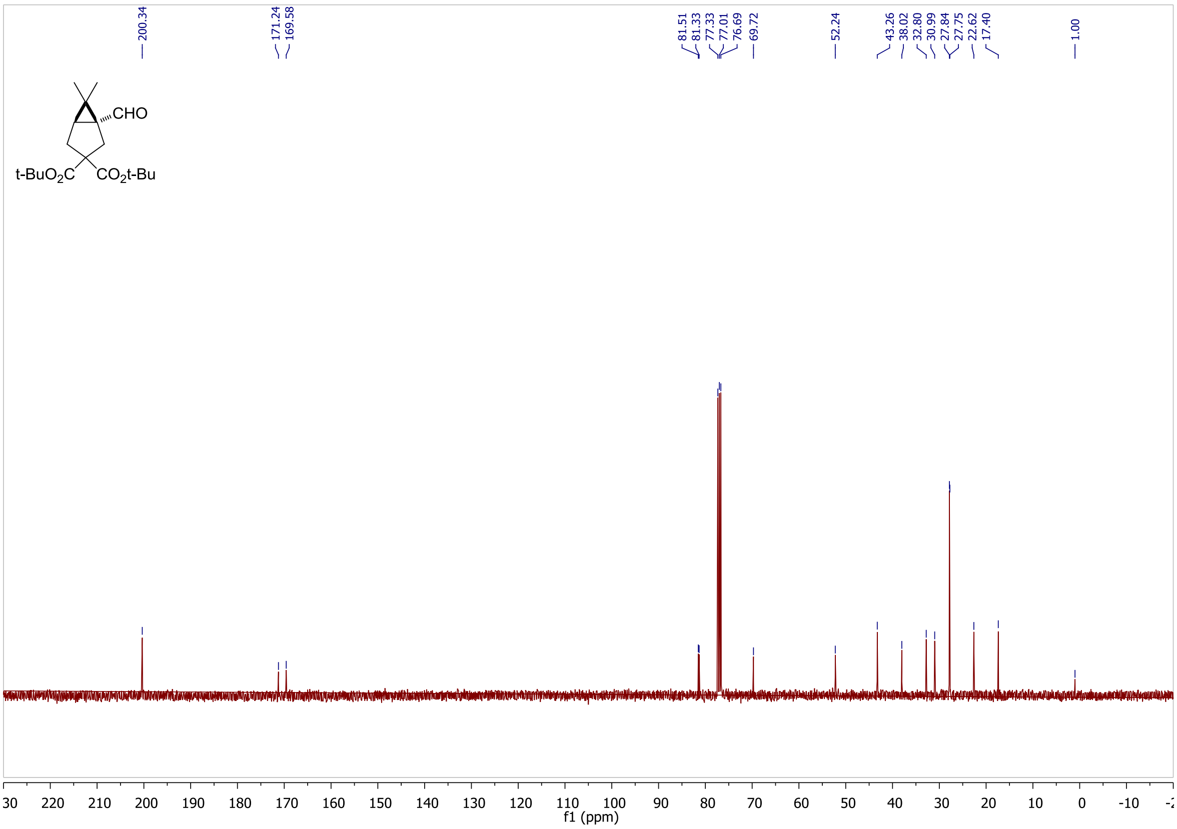

Supplementary Figure 34. ^{13}C NMR of the **2b** (101 MHz, $CDCl_3$)

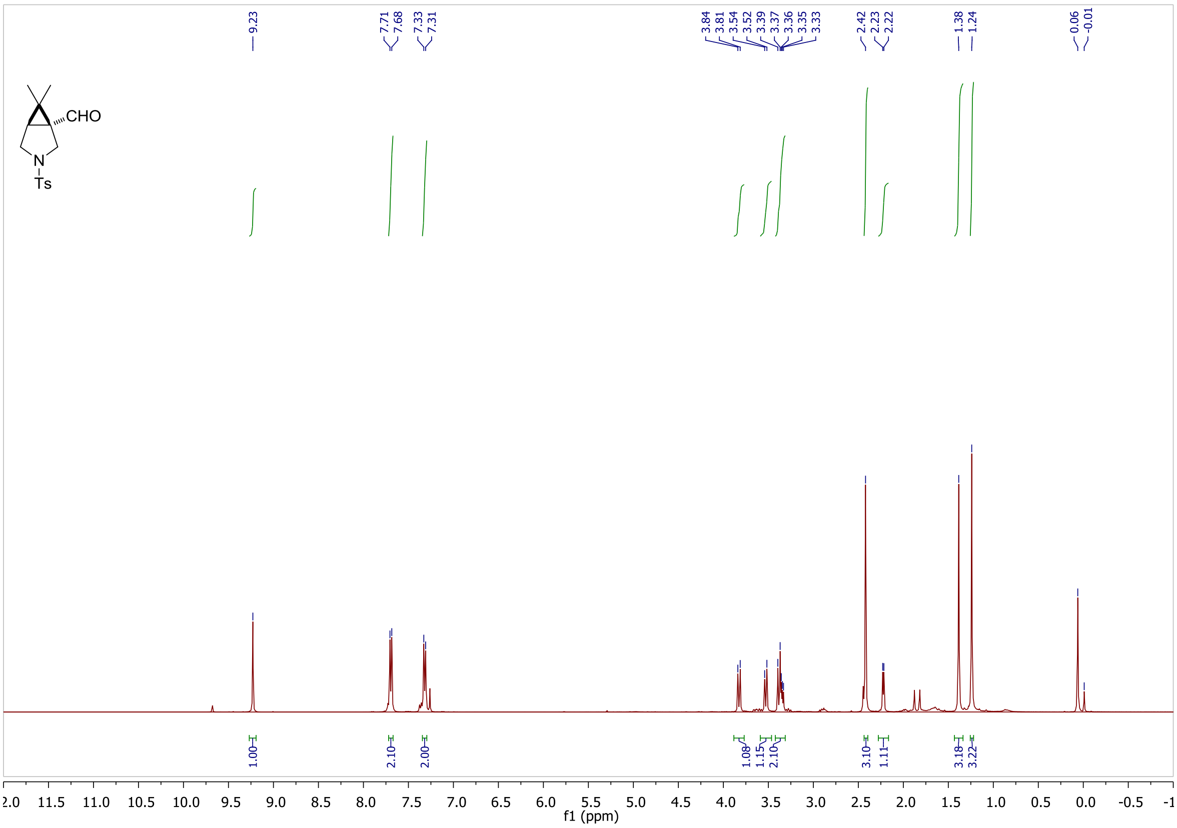

Supplementary Figure 35. ^{1}H NMR of the **2c** (400 MHz, $CDCl_3$)

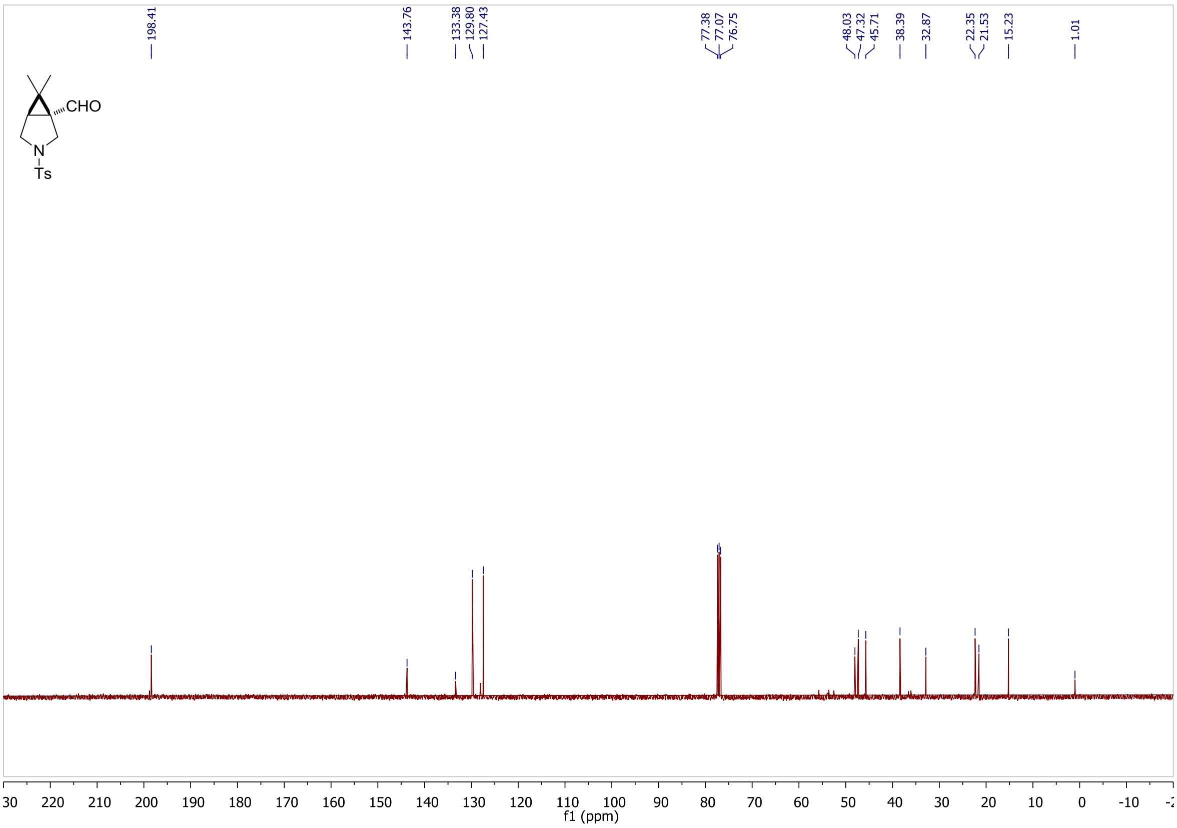

Supplementary Figure 36. ^{13}C NMR of the **2c** (101 MHz, $CDCl_3$)

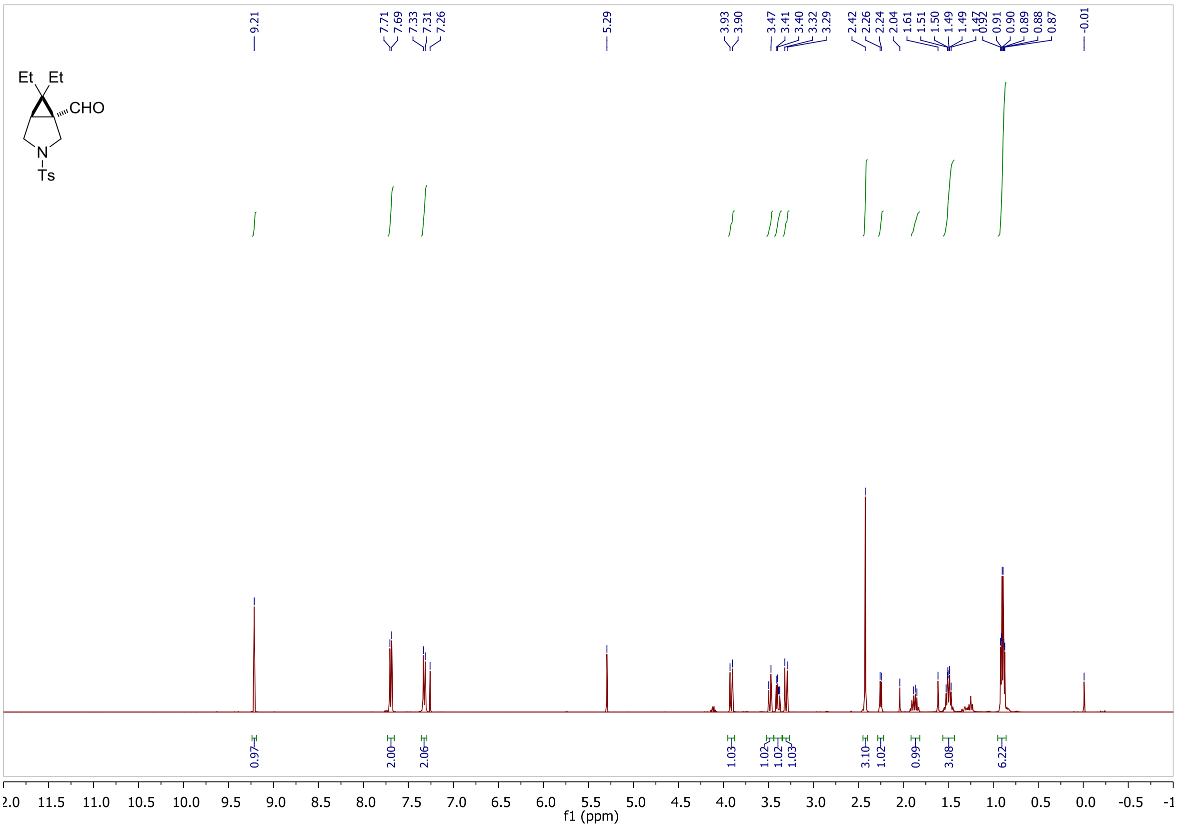

Supplementary Figure 37. ^{1}H NMR of the **2d** (400 MHz, $CDCl_3$)

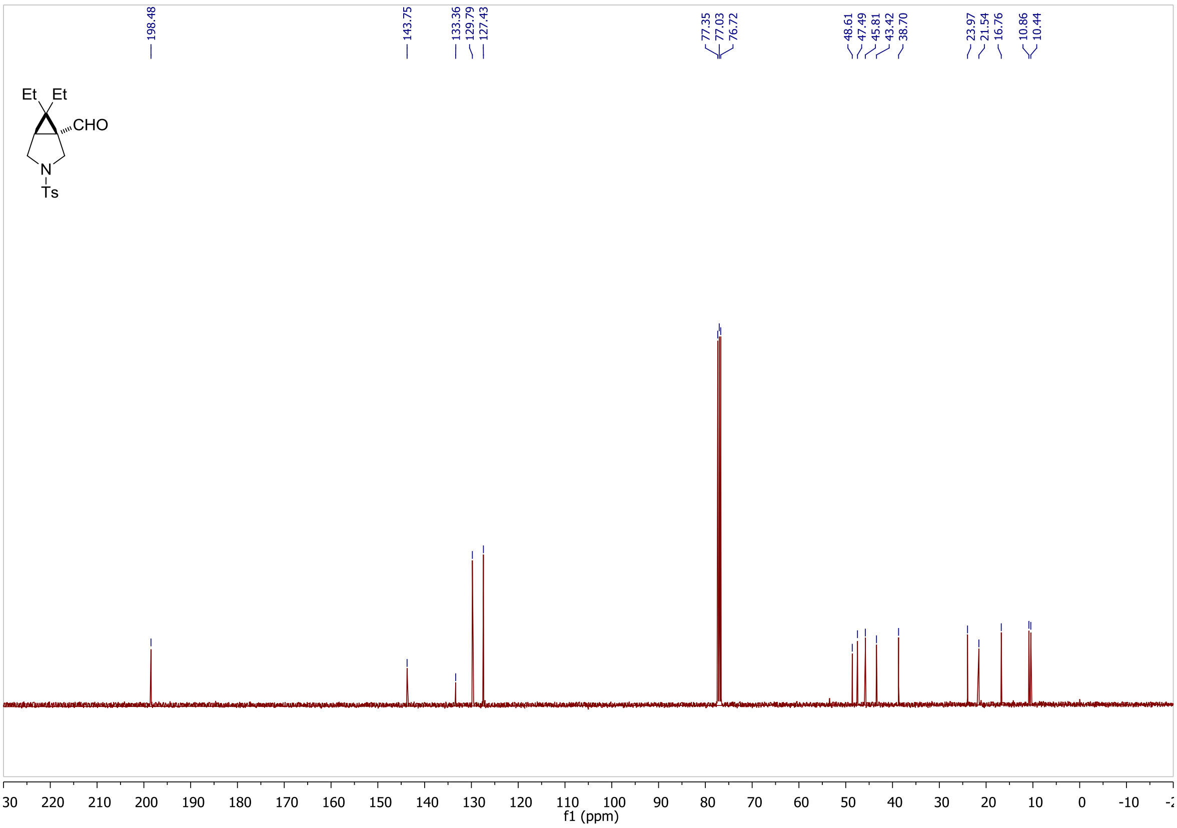

Supplementary Figure 38. ^{13}C NMR of the **2d** (101 MHz, $CDCl_3$)

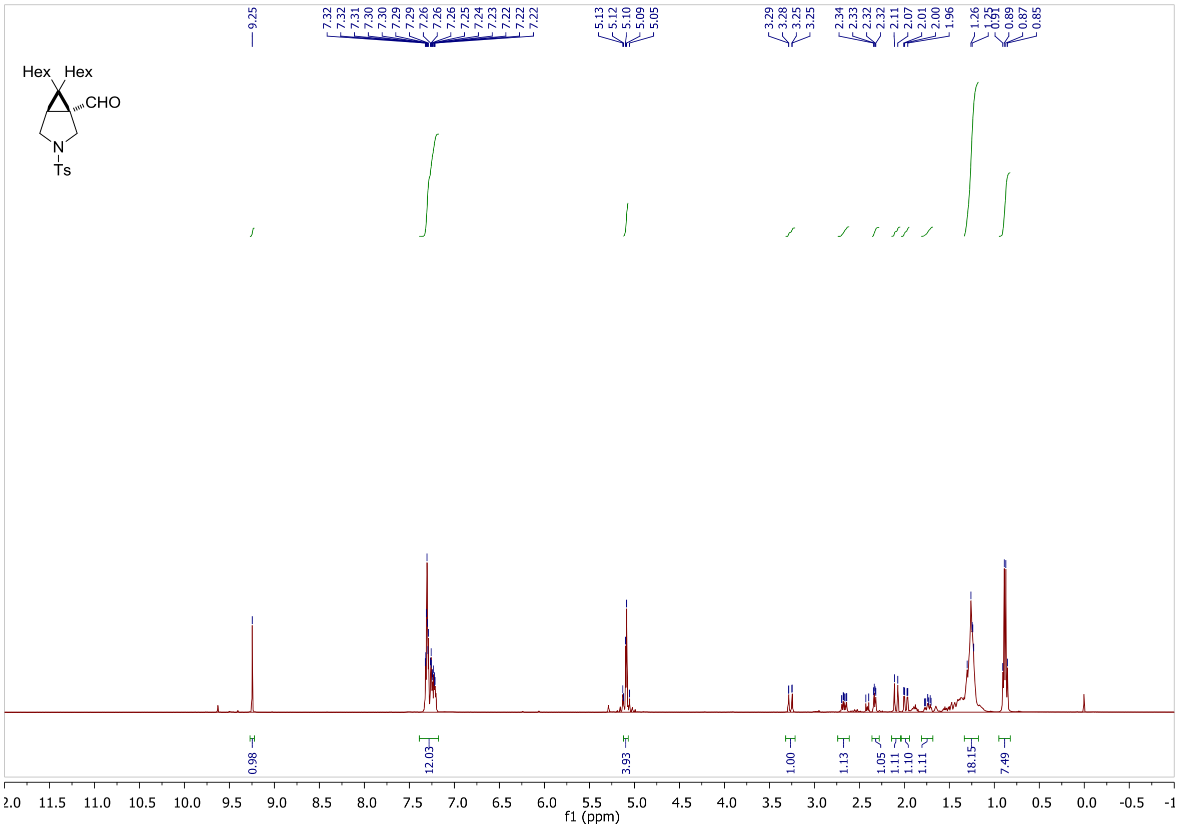

Supplementary Figure 39. ^{1}H NMR of the **2e** (400 MHz, $CDCl_3$)

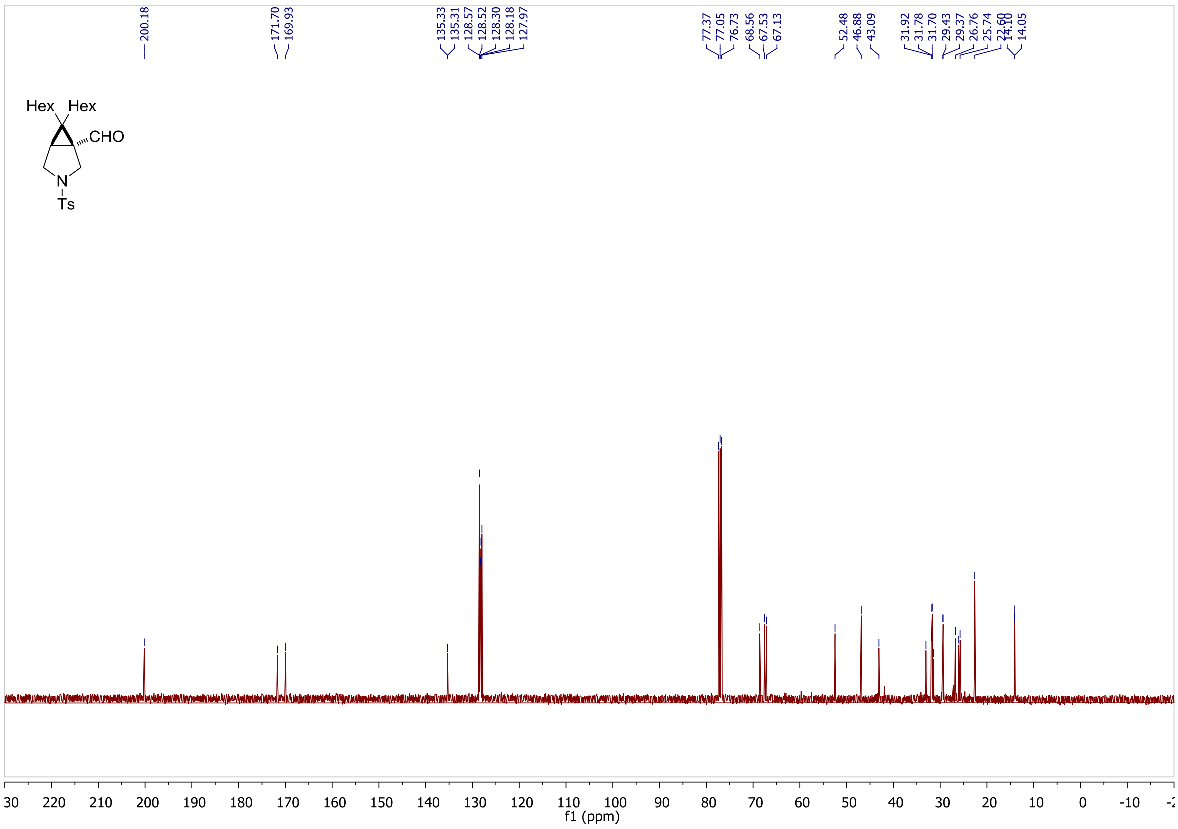

Supplementary Figure 40. ^{13}C NMR of the **2e** (101 MHz, $CDCl_3$)

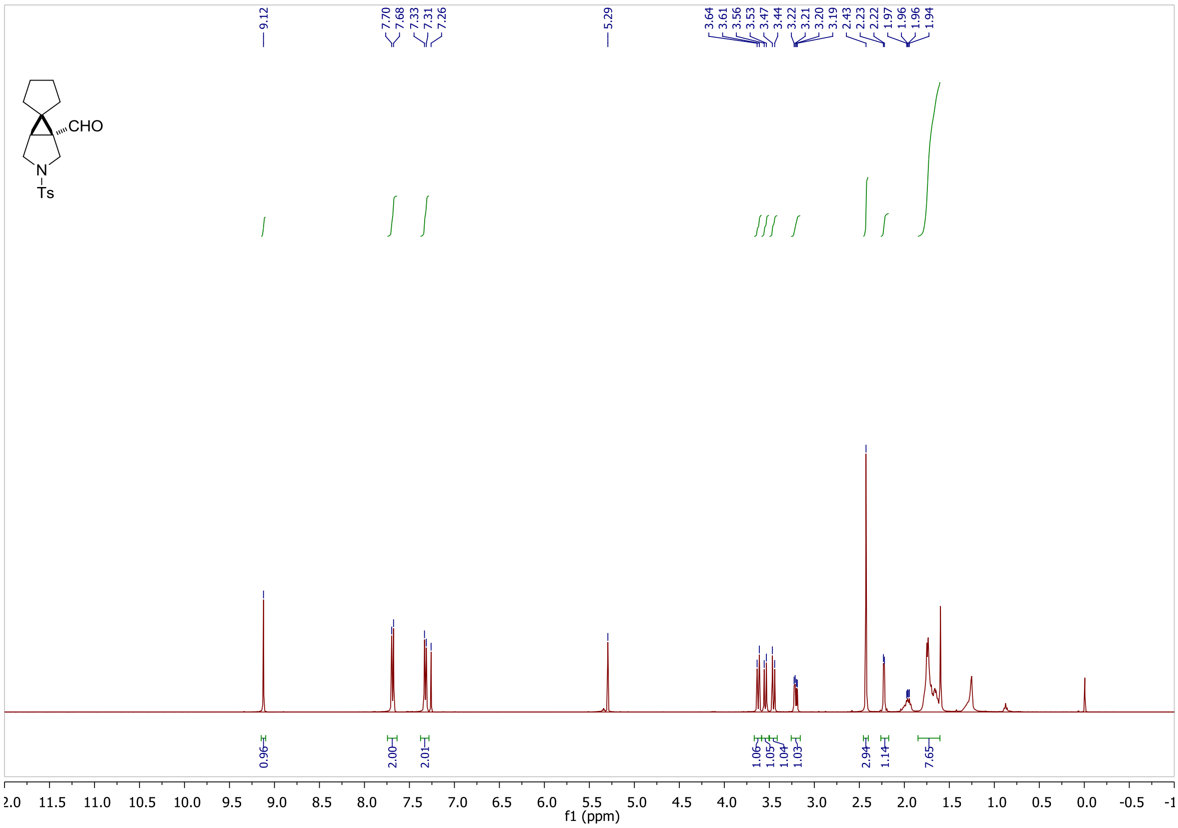

Supplementary Figure 41. ^{1}H NMR of the **2f** (400 MHz, $CDCl_3$)

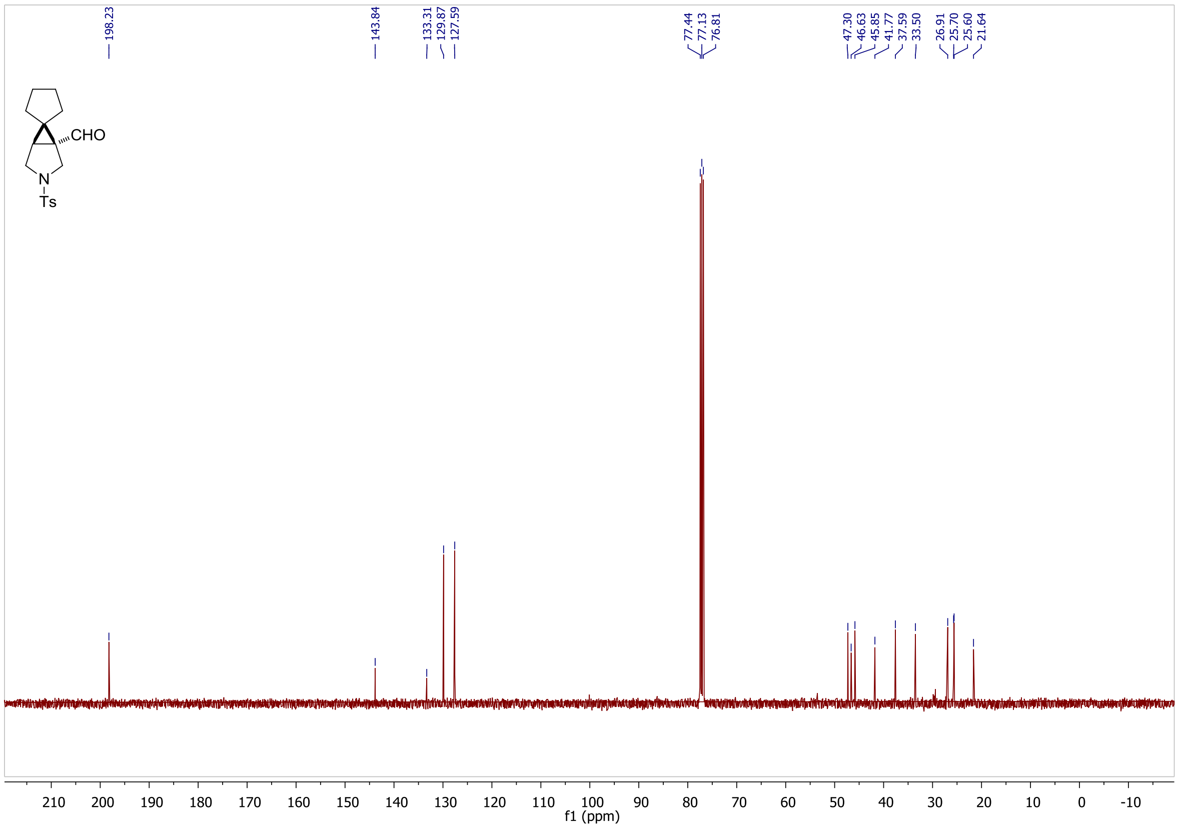

Supplementary Figure 42. ^{13}C NMR of the **16f** (101 MHz, $CDCl_3$)

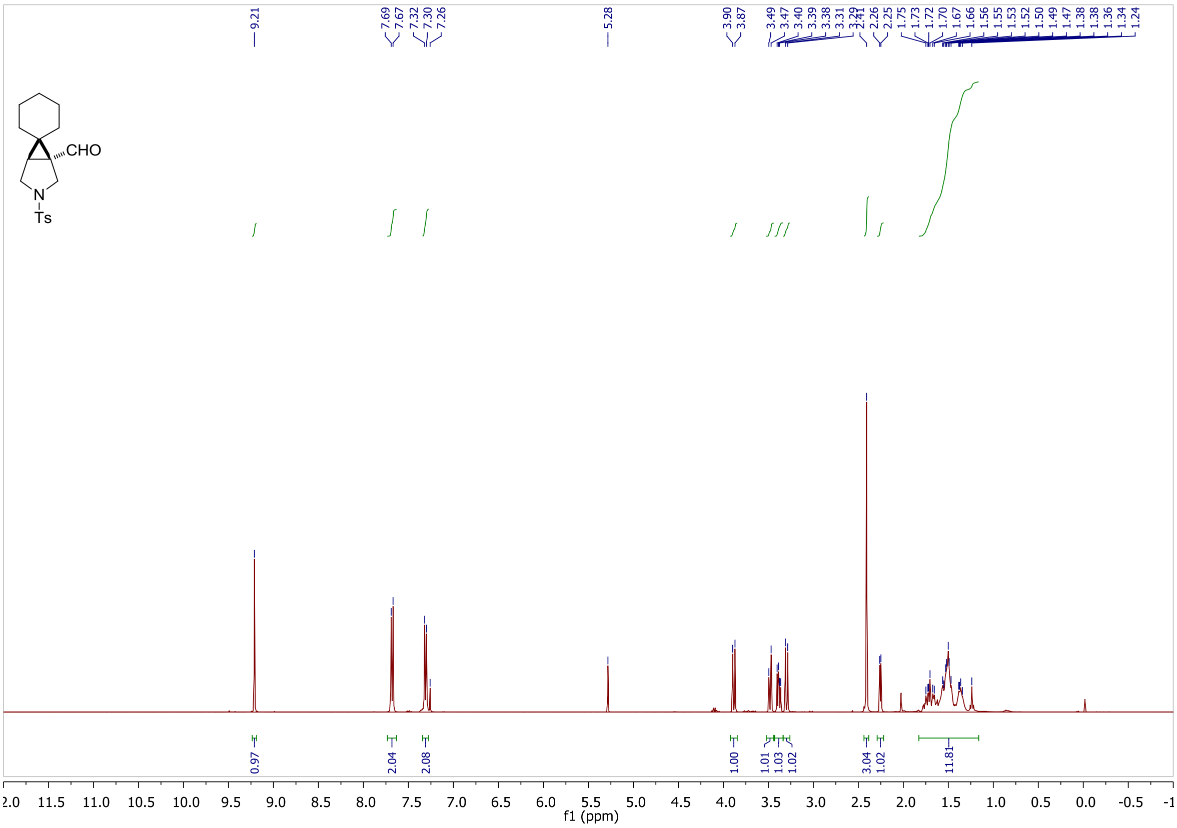

Supplementary Figure 43. ^{1}H NMR of the **2g** (400 MHz, $CDCl_3$)

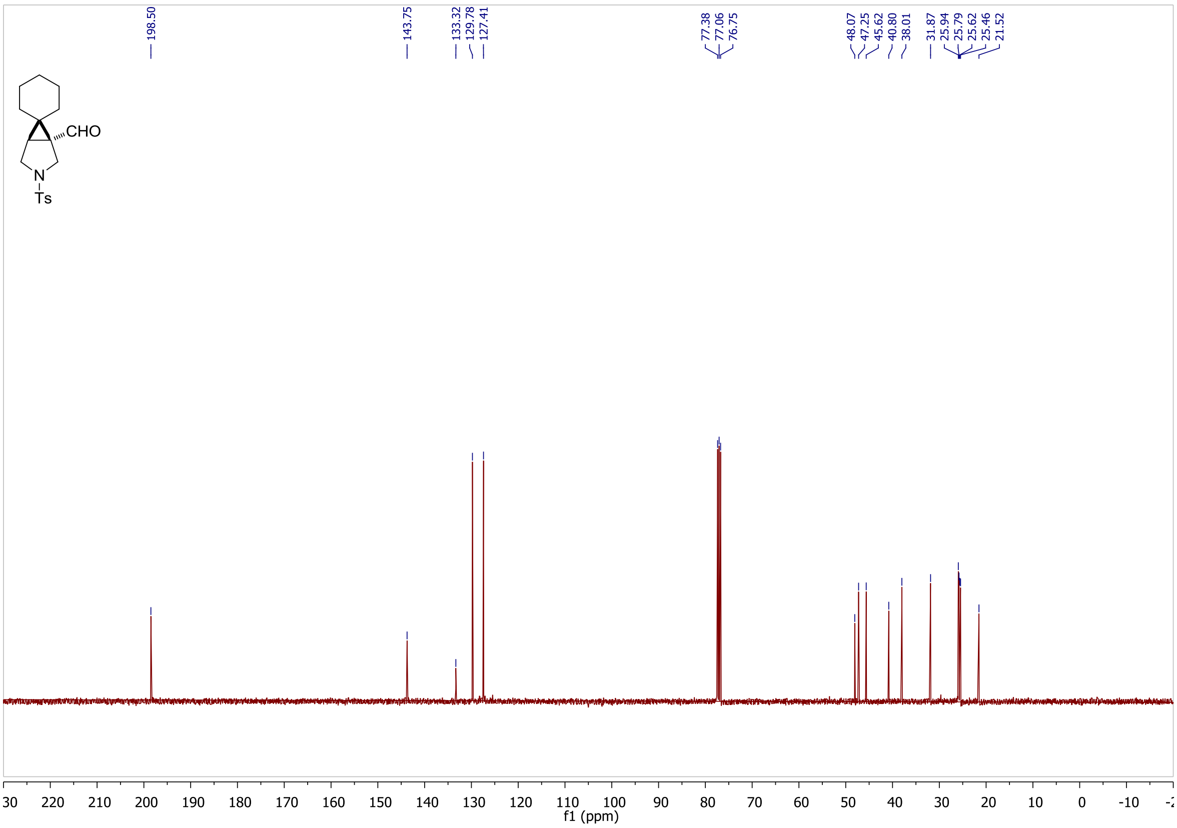

Supplementary Figure 44. ^{13}C NMR of the **2g** (101 MHz, $CDCl_3$)

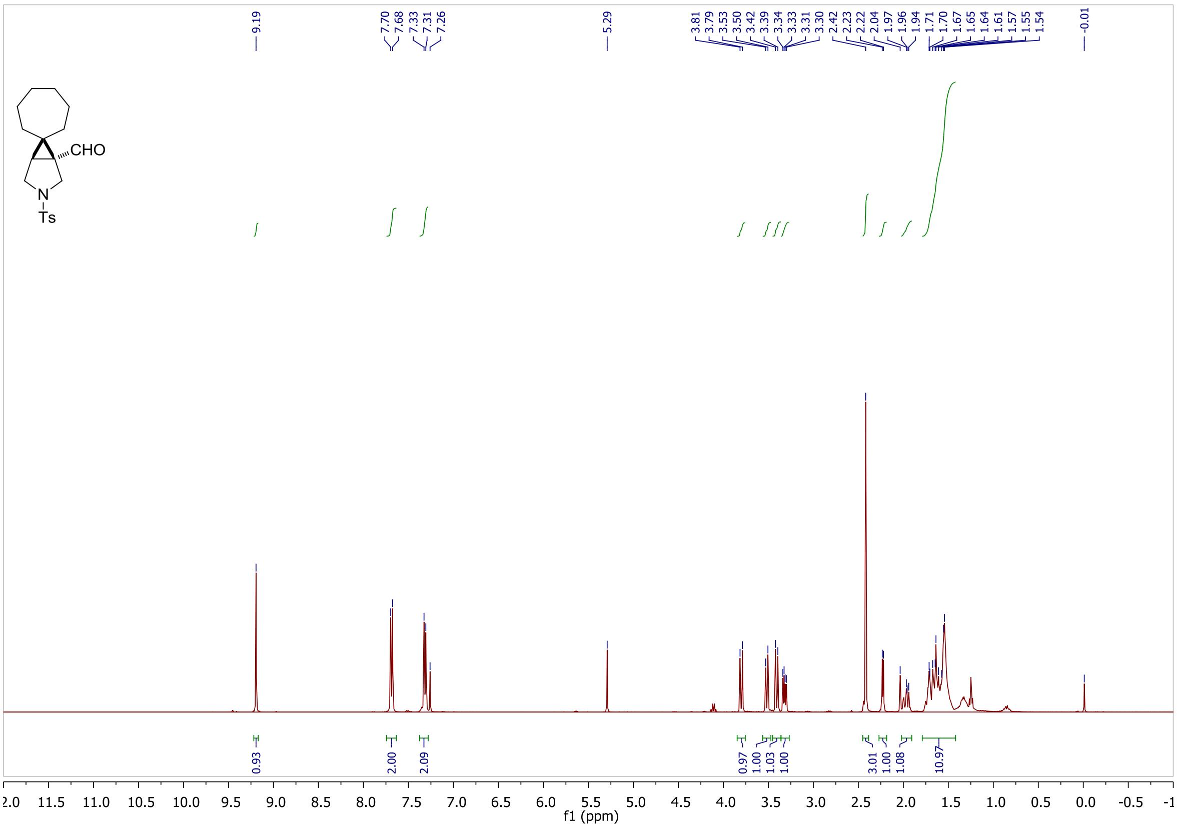

Supplementary Figure 45. ^{1}H NMR of the **2h** (400 MHz, $CDCl_3$)

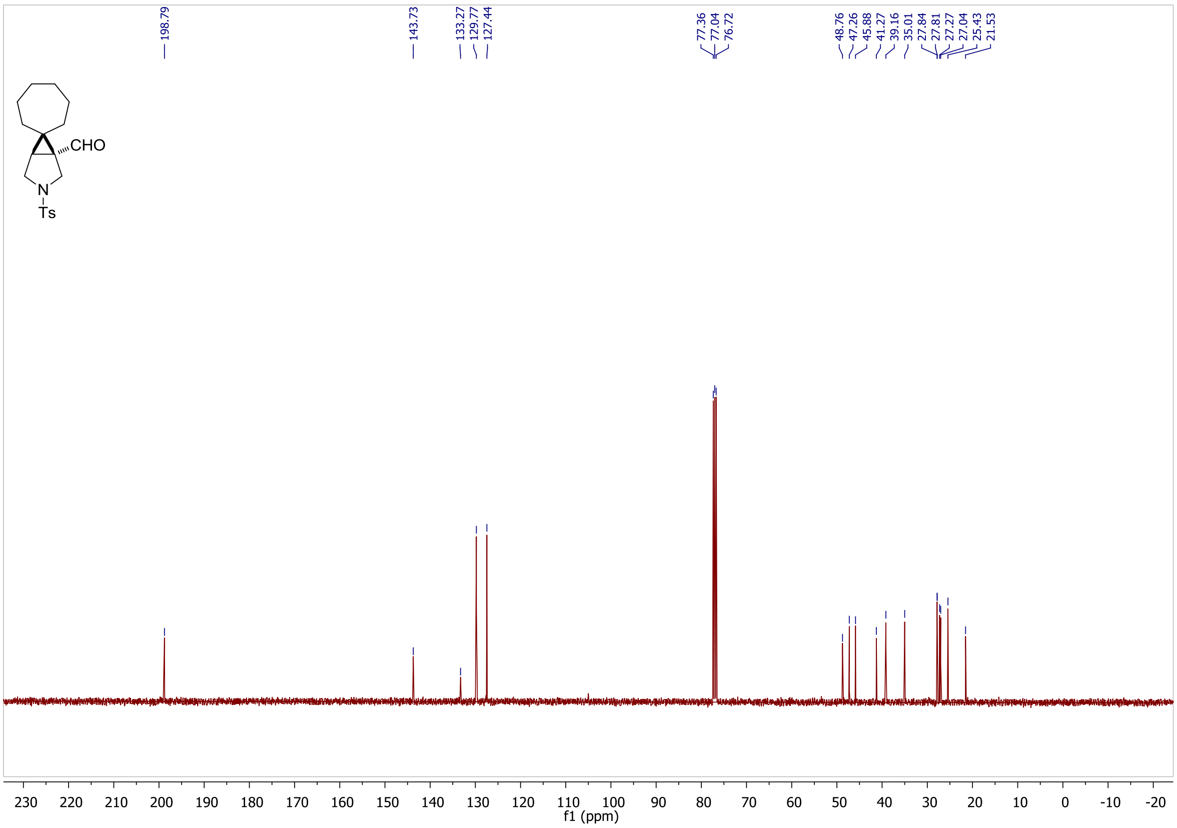

Supplementary Figure 46. ^{13}C NMR of the **2h** (101 MHz, $CDCl_3$)

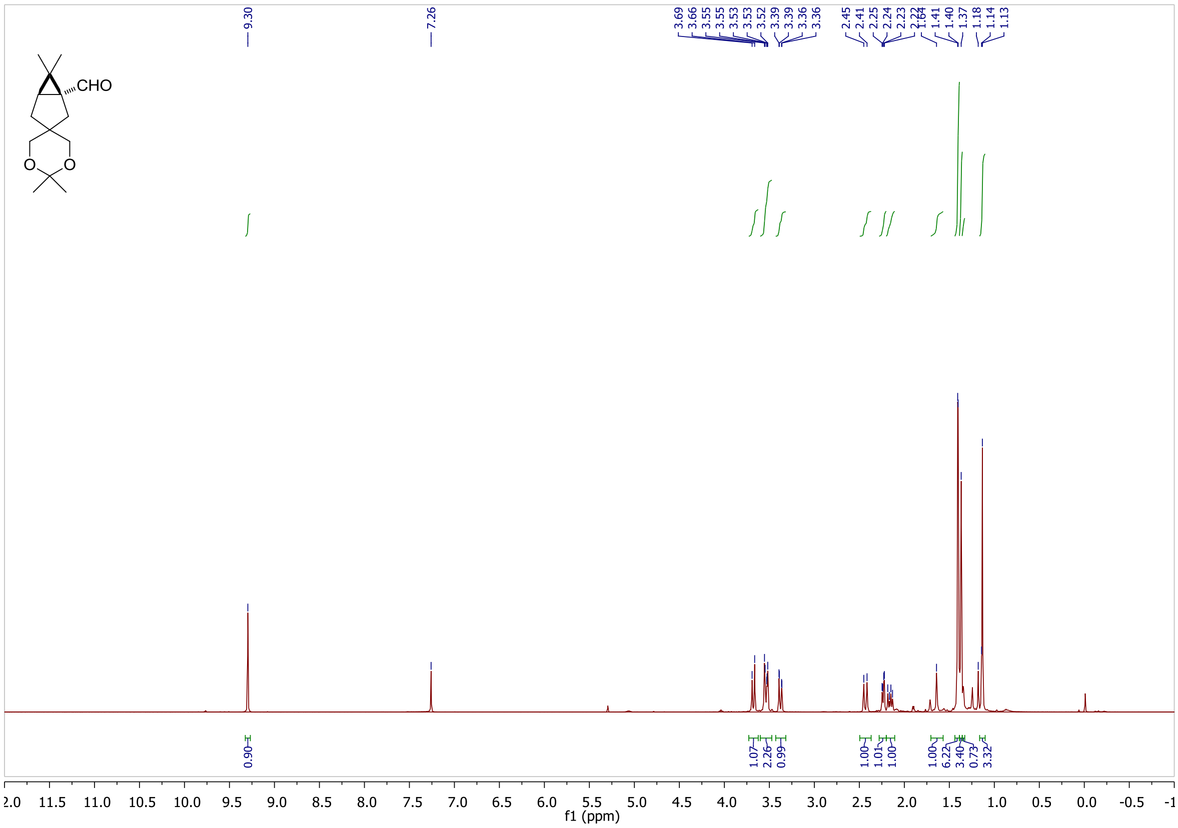

Supplementary Figure 47. ^{1}H NMR of the **2i** (400 MHz, $CDCl_3$)

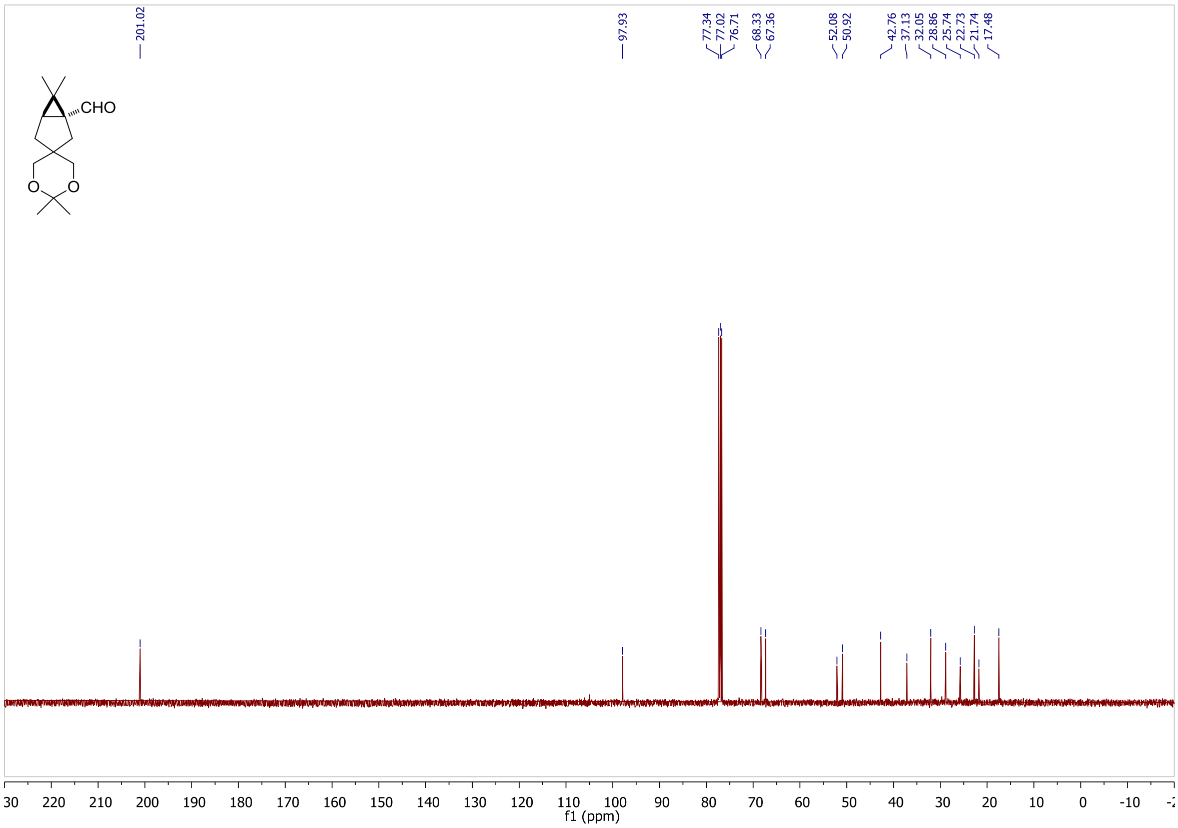

Supplementary Figure 48. ^{13}C NMR of the **2i** (101 MHz, $CDCl_3$)

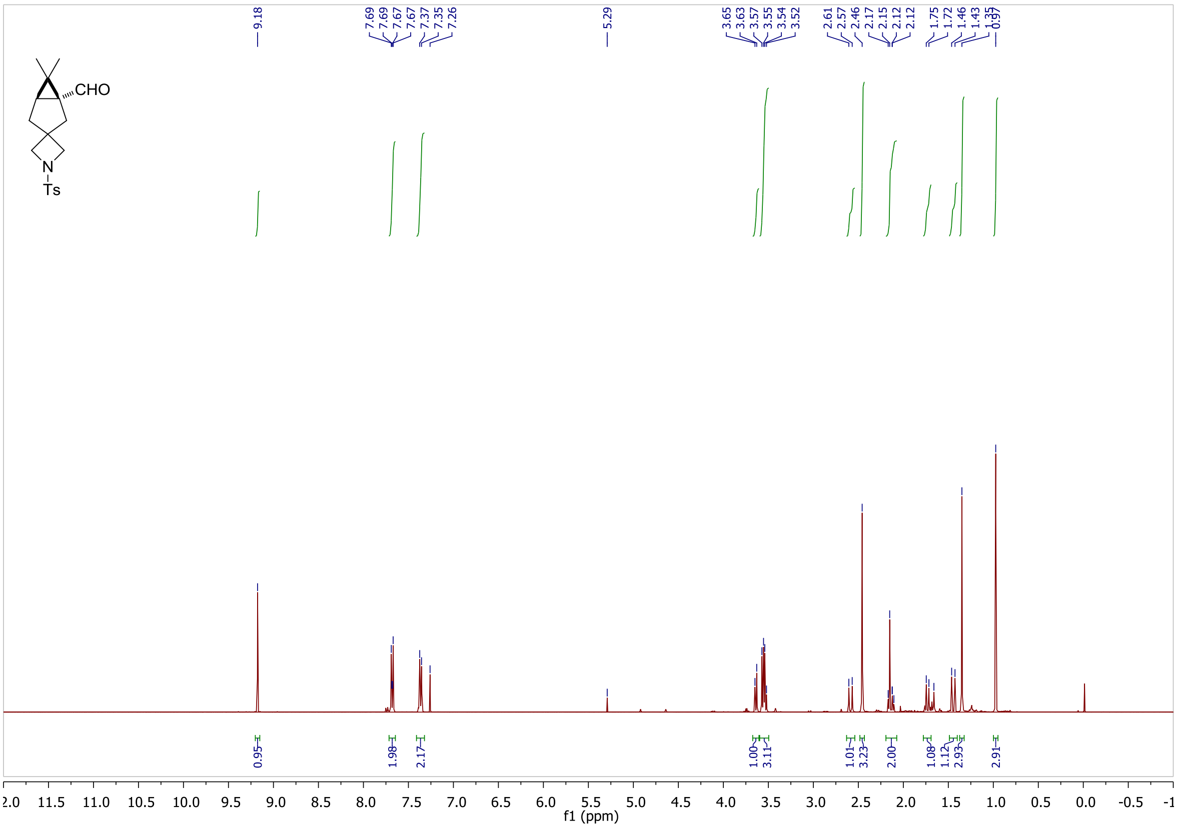

Supplementary Figure 49. ^{1}H NMR of the **2j** (400 MHz, $CDCl_3$)

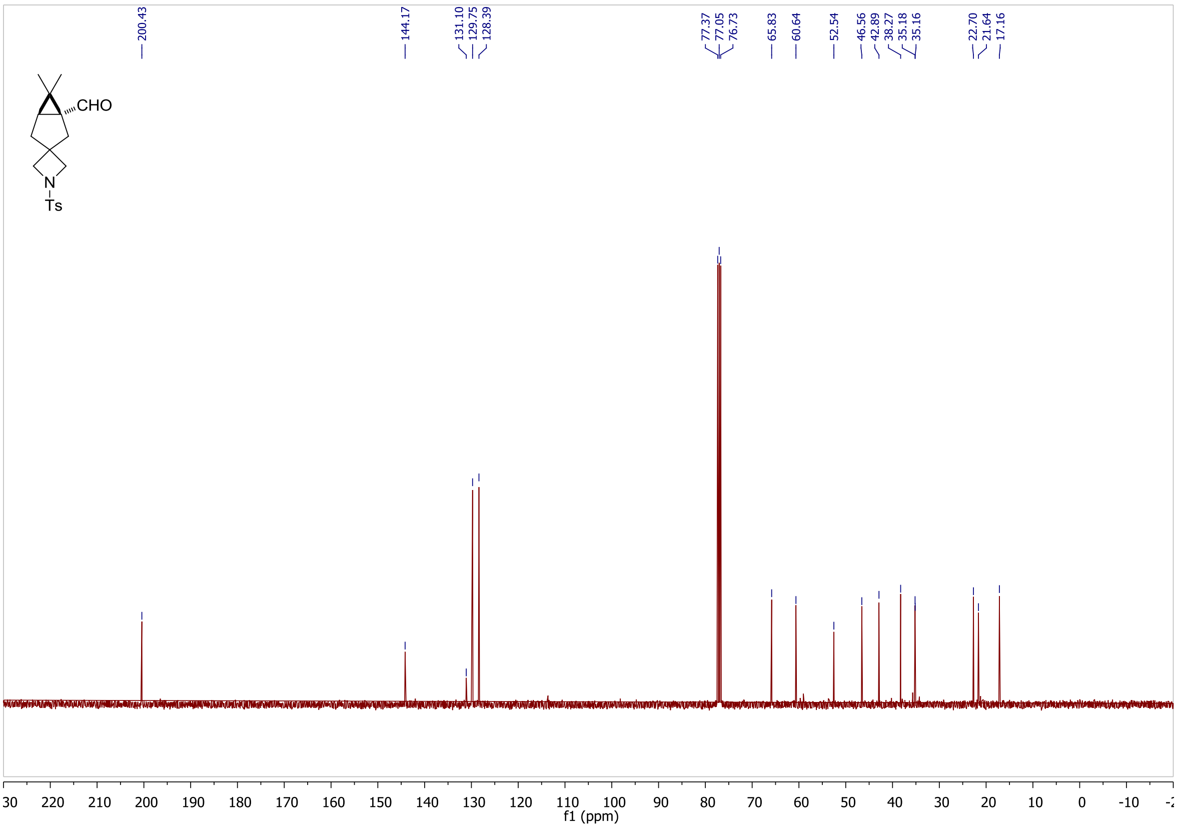

Supplementary Figure 50. ^{13}C NMR of the **2j** (101 MHz, $CDCl_3$)

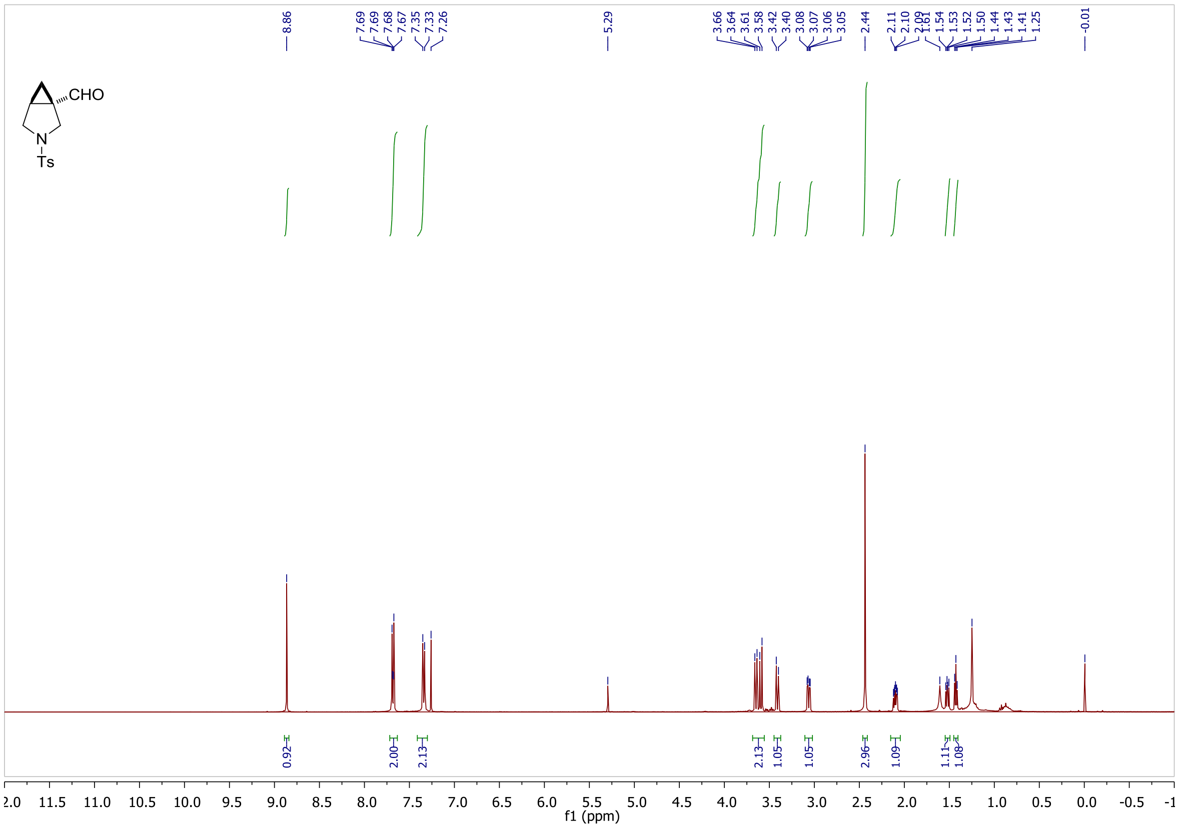

Supplementary Figure 51. ^{1}H NMR of the **2k** (400 MHz, $CDCl_3$)

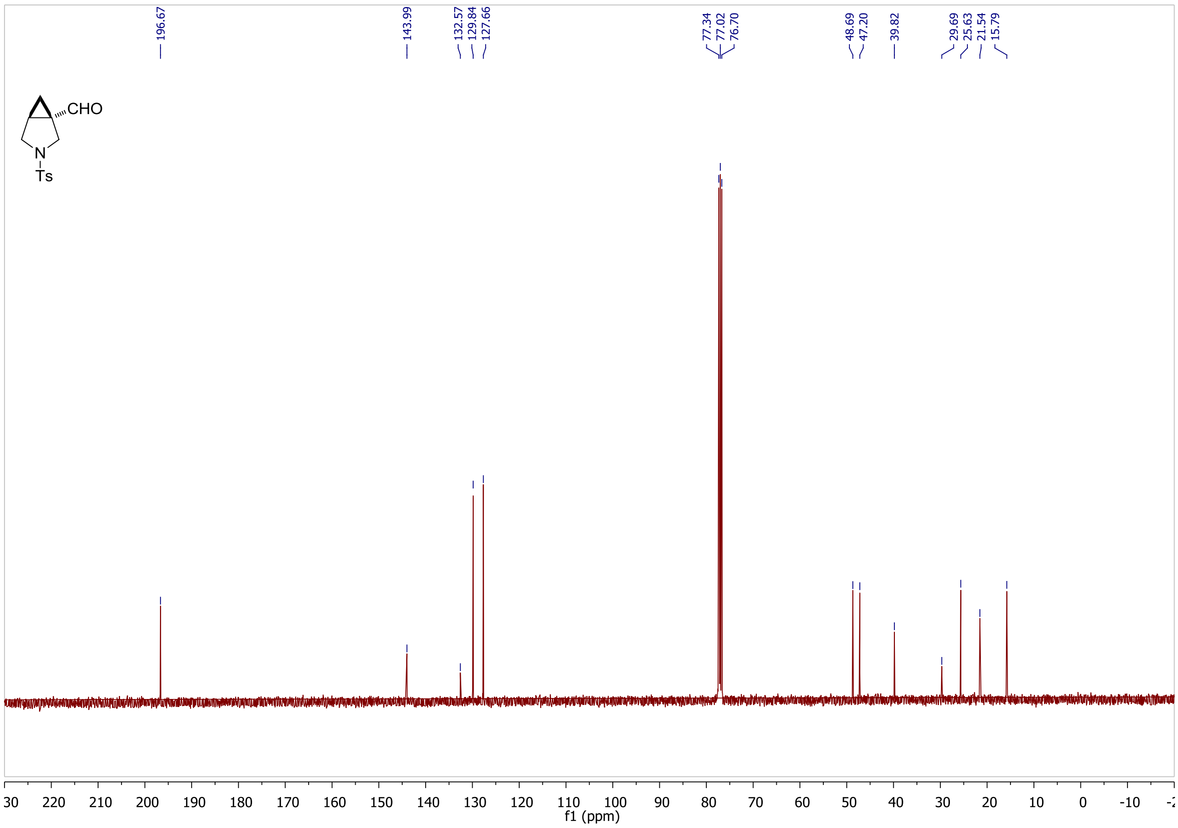

Supplementary Figure 52. ^{13}C NMR of the **2k** (101 MHz, $CDCl_3$)

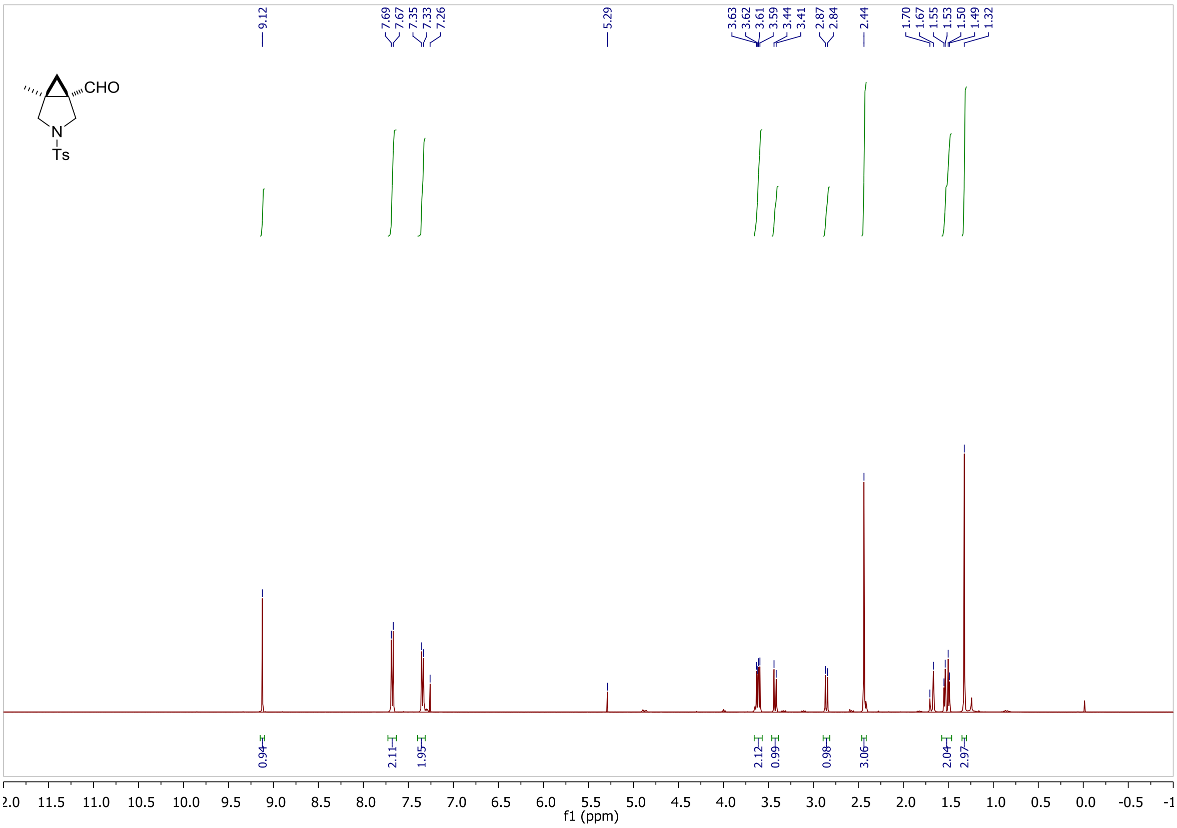

Supplementary Figure 53. ^{1}H NMR of the **2l** (400 MHz, $CDCl_3$)

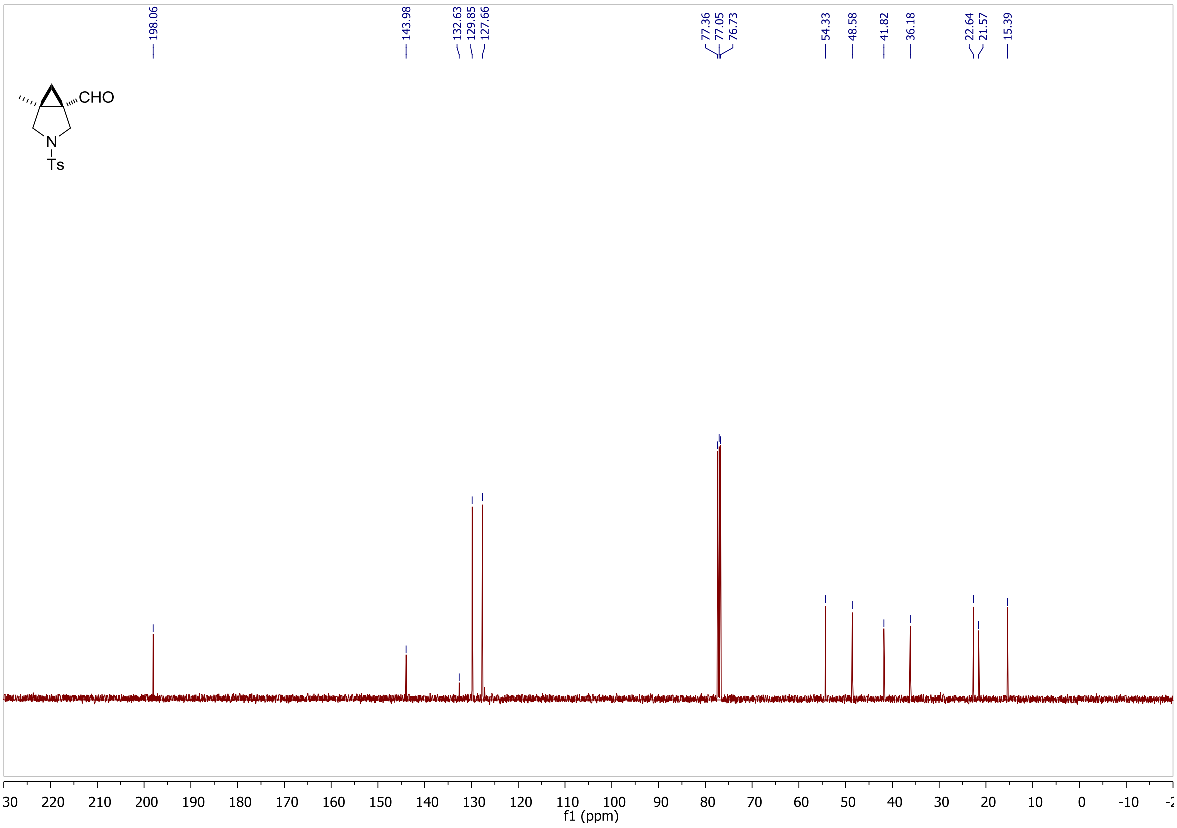

Supplementary Figure 54. ^{13}C NMR of the **2l** (101 MHz, $CDCl_3$)

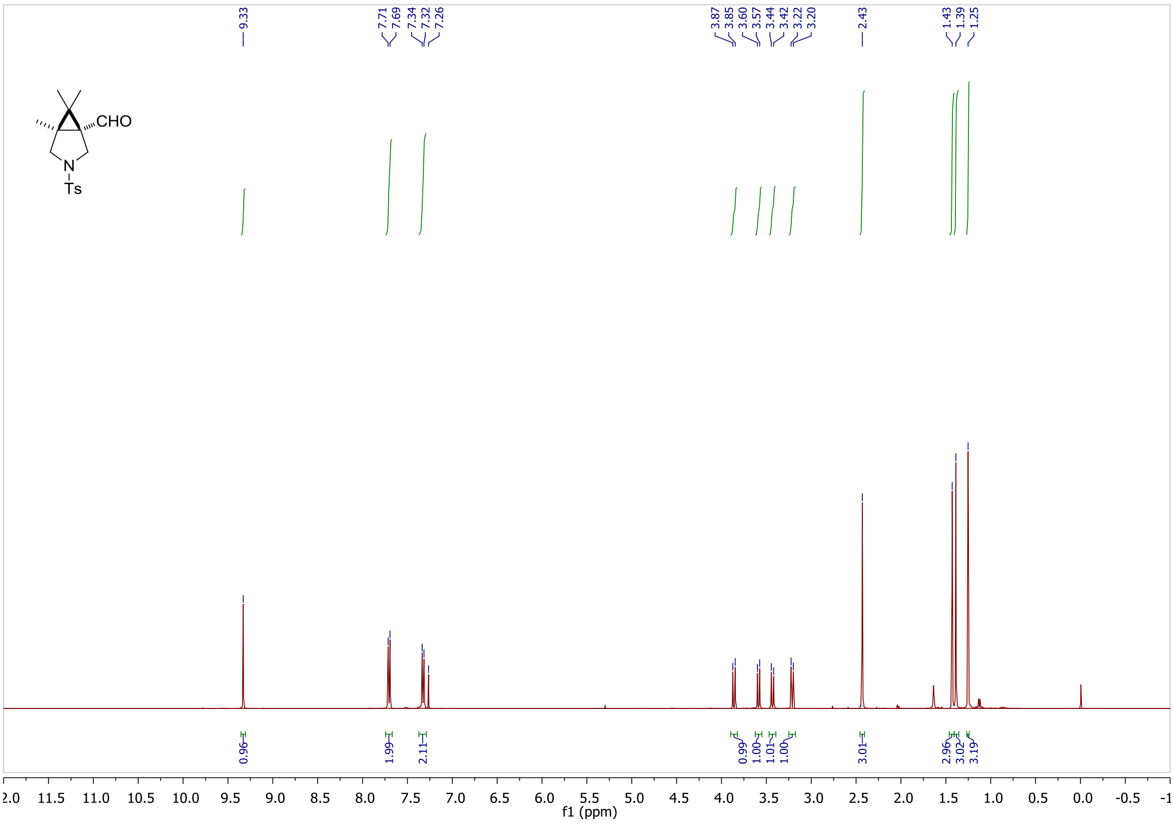

Supplementary Figure 55. ^{1}H NMR of the **2m** (400 MHz, $CDCl_3$)

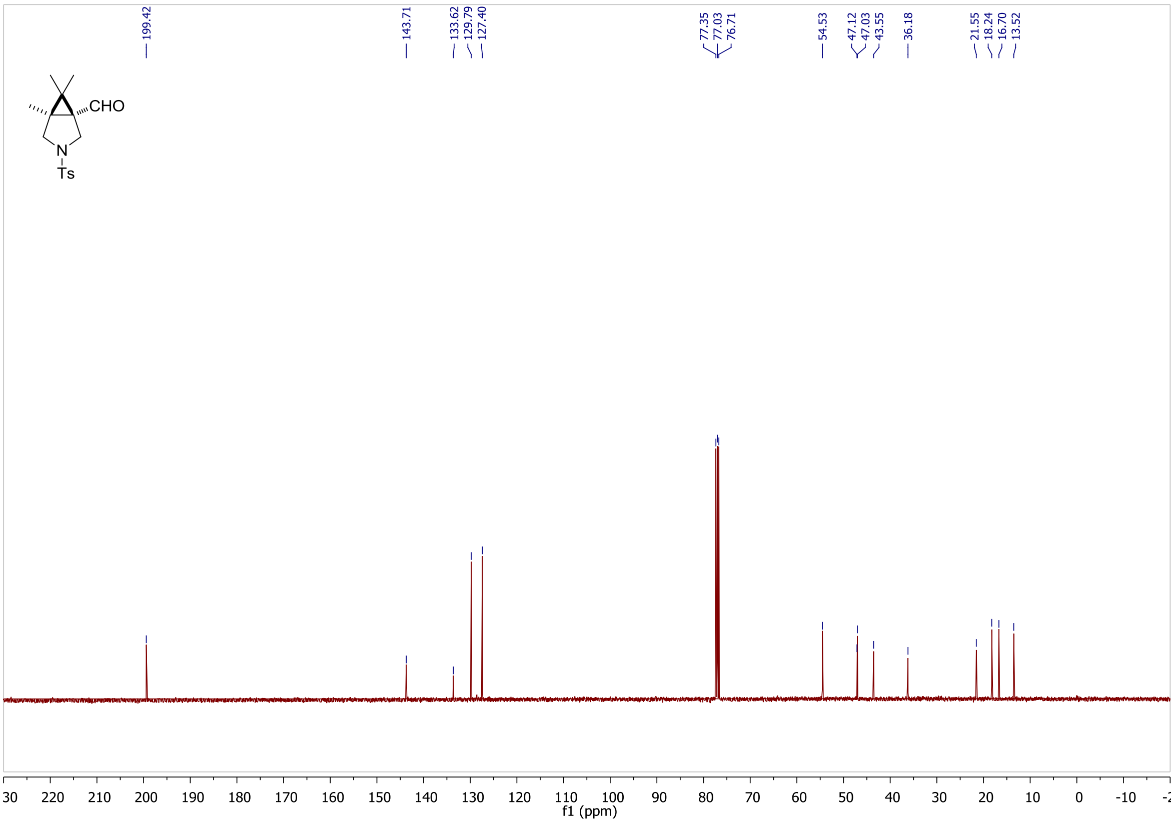

Supplementary Figure 56. ^{13}C NMR of the **2m** (101 MHz, $CDCl_3$)

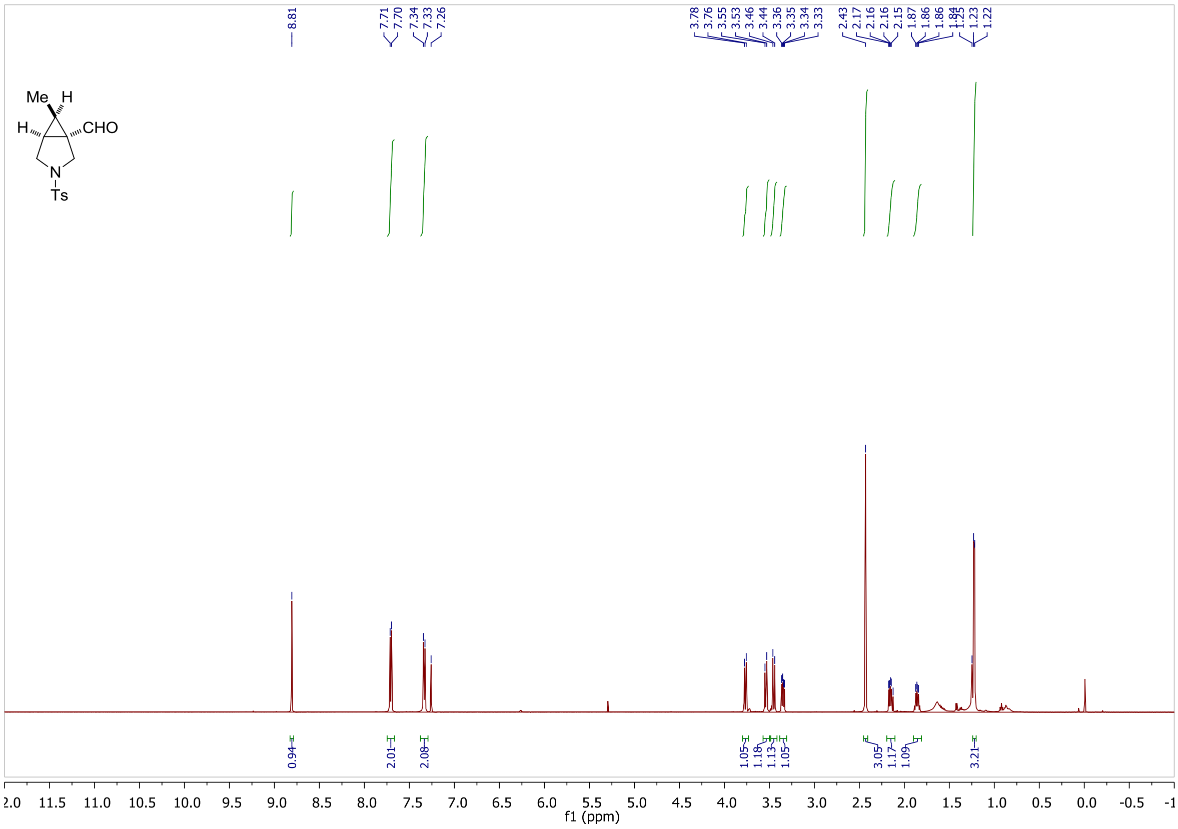

Supplementary Figure 57. ^{1}H NMR of the trans-**2n** (500 MHz, $CDCl_3$)

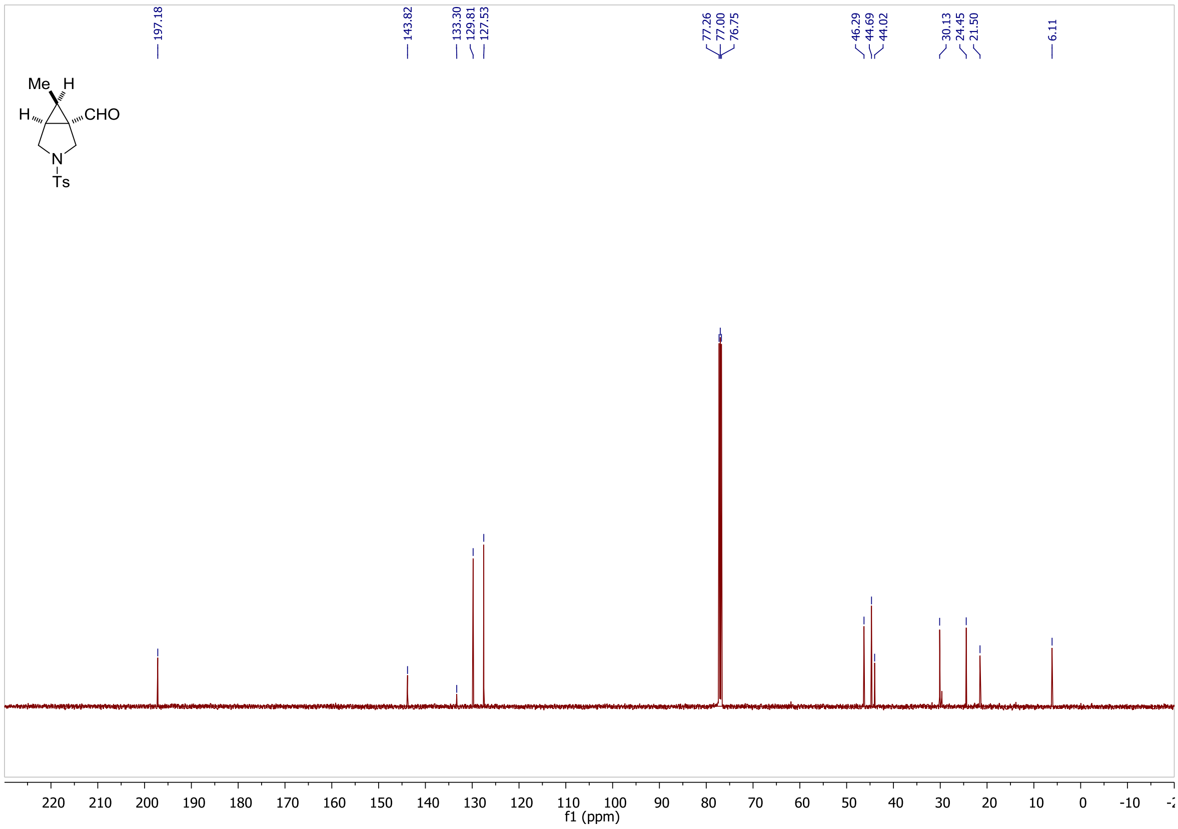

Supplementary Figure 58. ^{13}C NMR of the trans-**2n** (126 MHz, $CDCl_3$)

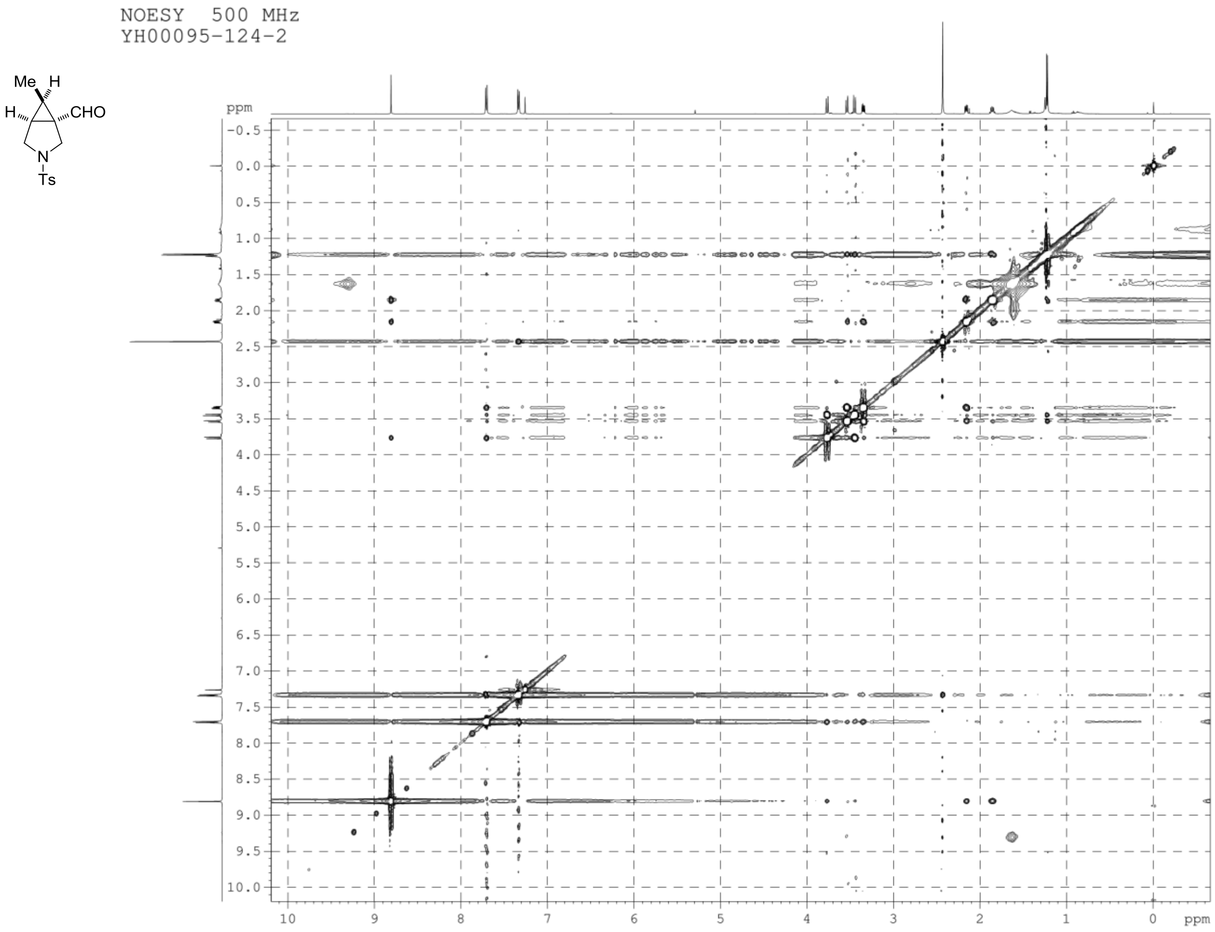

Supplementary Figure 59. NOESY of the trans-**2n** (126 MHz, $CDCl_3$)

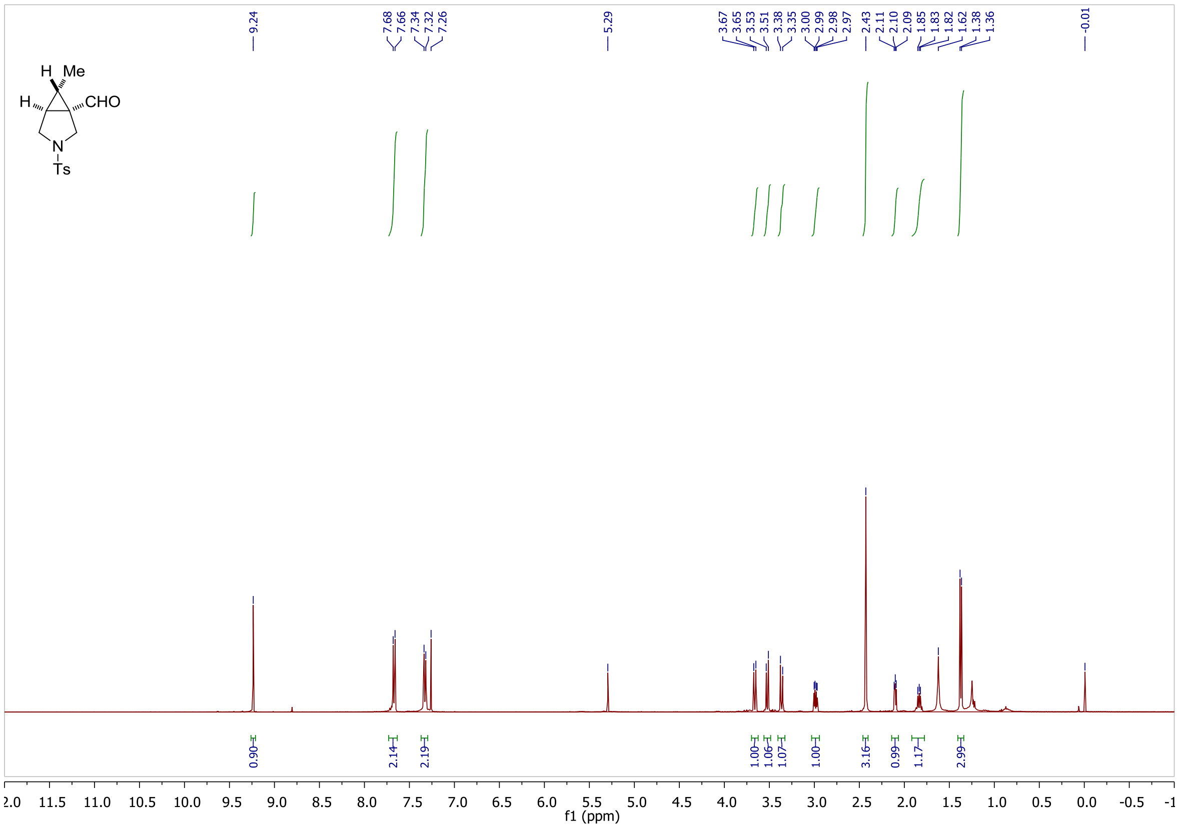

Supplementary Figure 60. ^{1}H NMR of the cis-**2n** (400 MHz, $CDCl_3$)

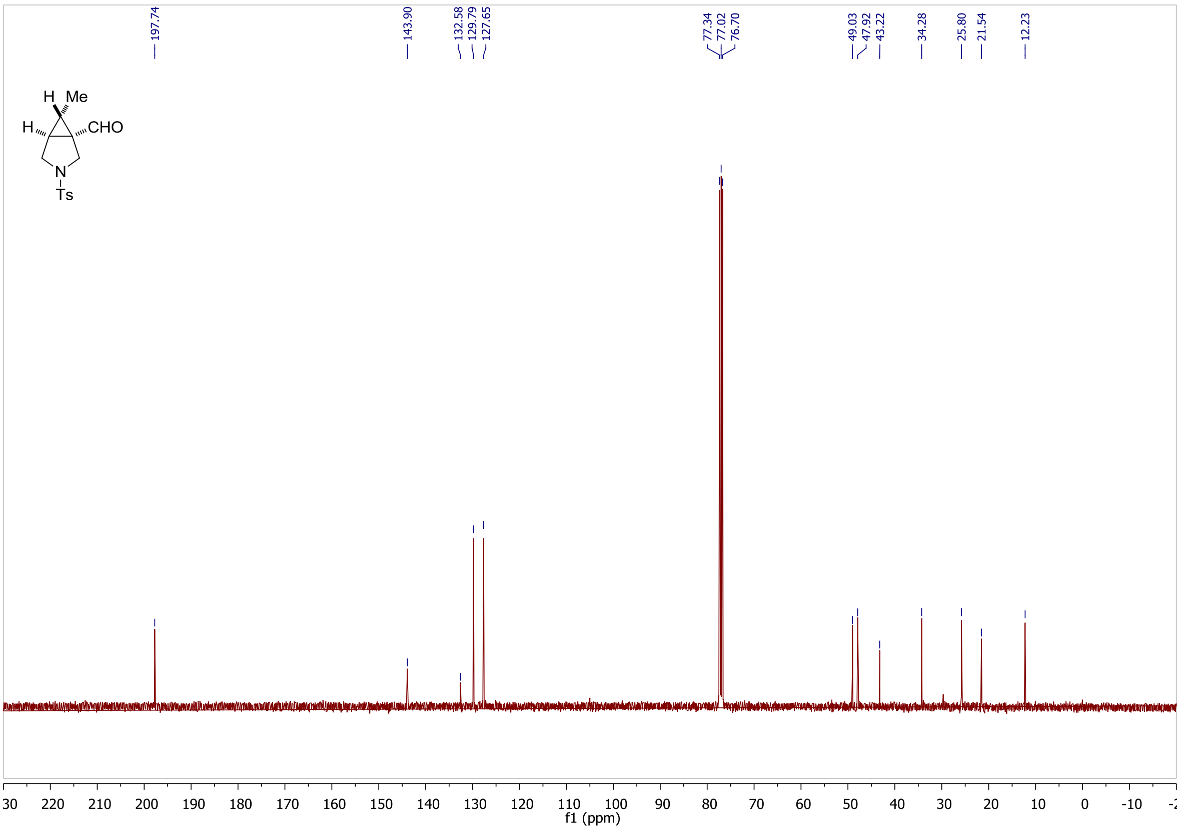

Supplementary Figure 61. ^{13}C NMR of the cis-**2n** (101 MHz, $CDCl_3$)

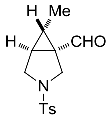

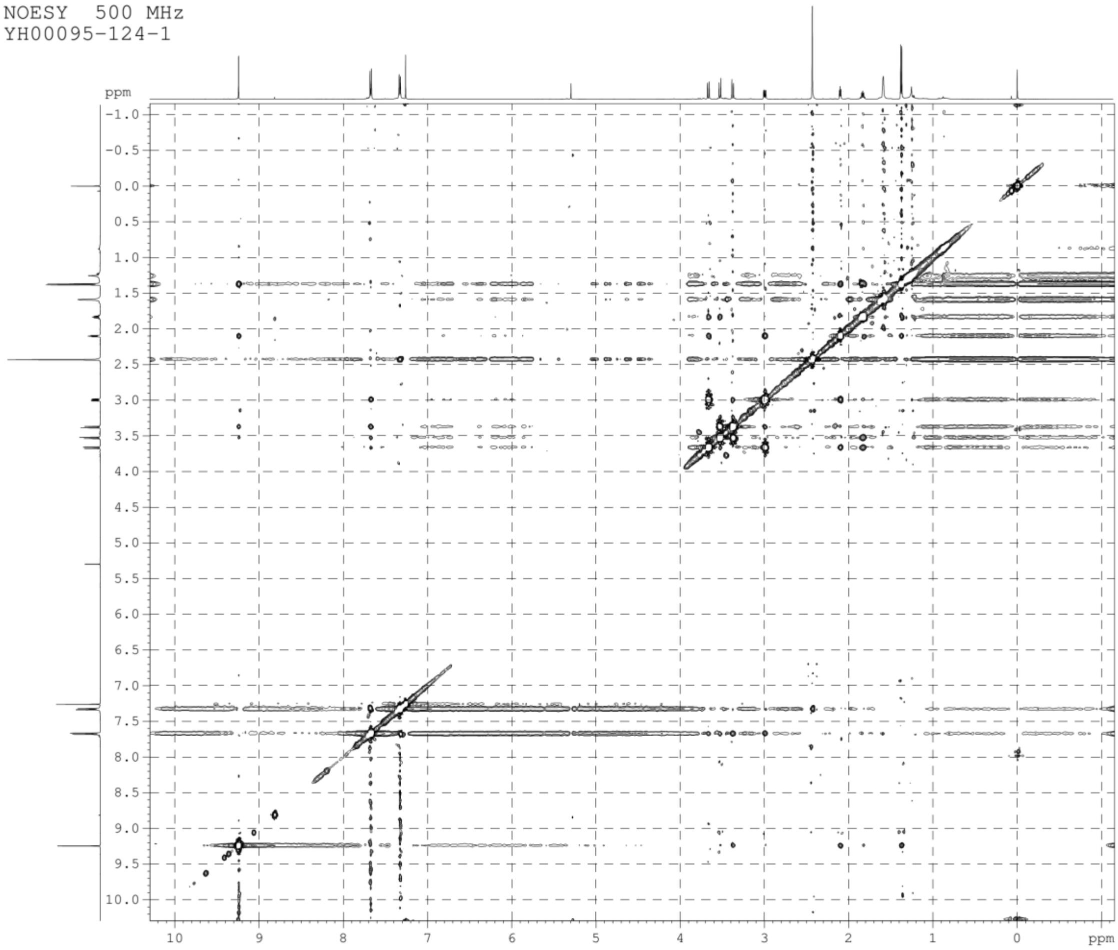

Supplementary Figure 62. NOESY of the cis-**2n** (126 MHz, $CDCl_3$)

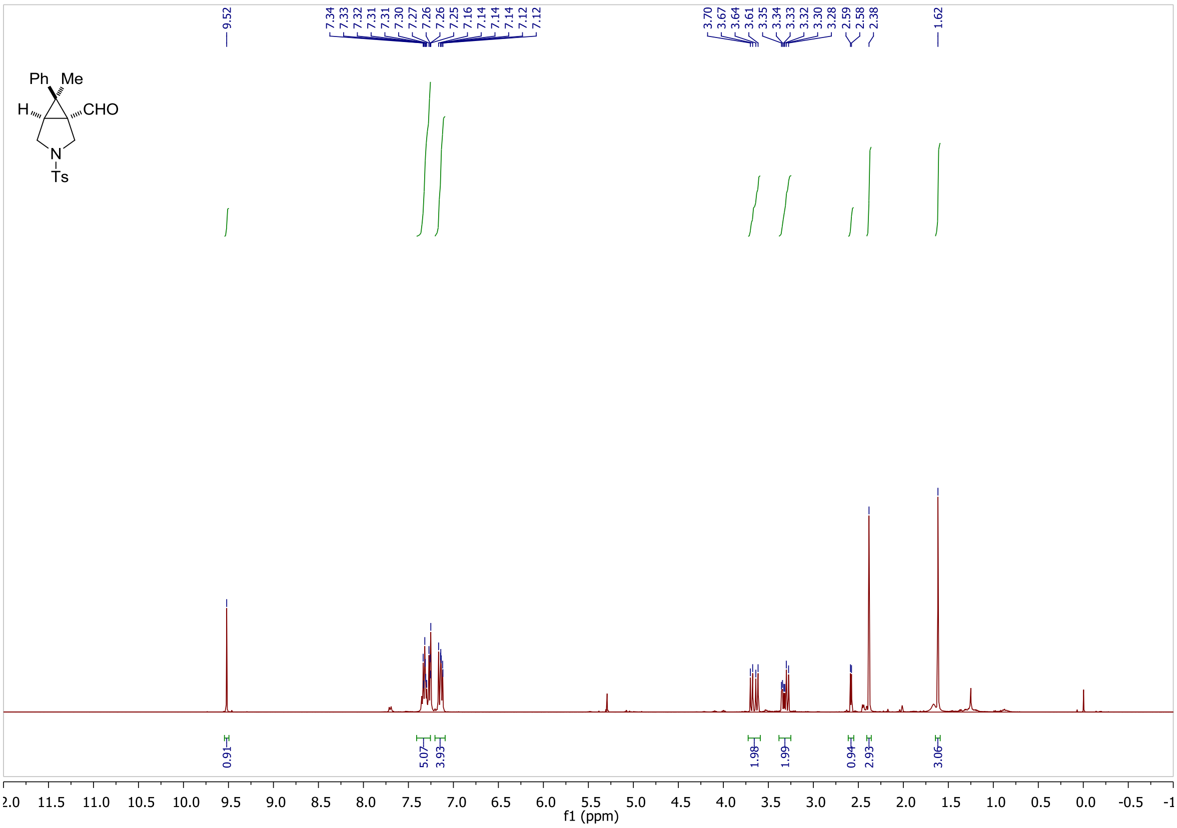

Supplementary Figure 63. ^{1}H NMR of the trans-**2o** (400 MHz, $CDCl_3$)

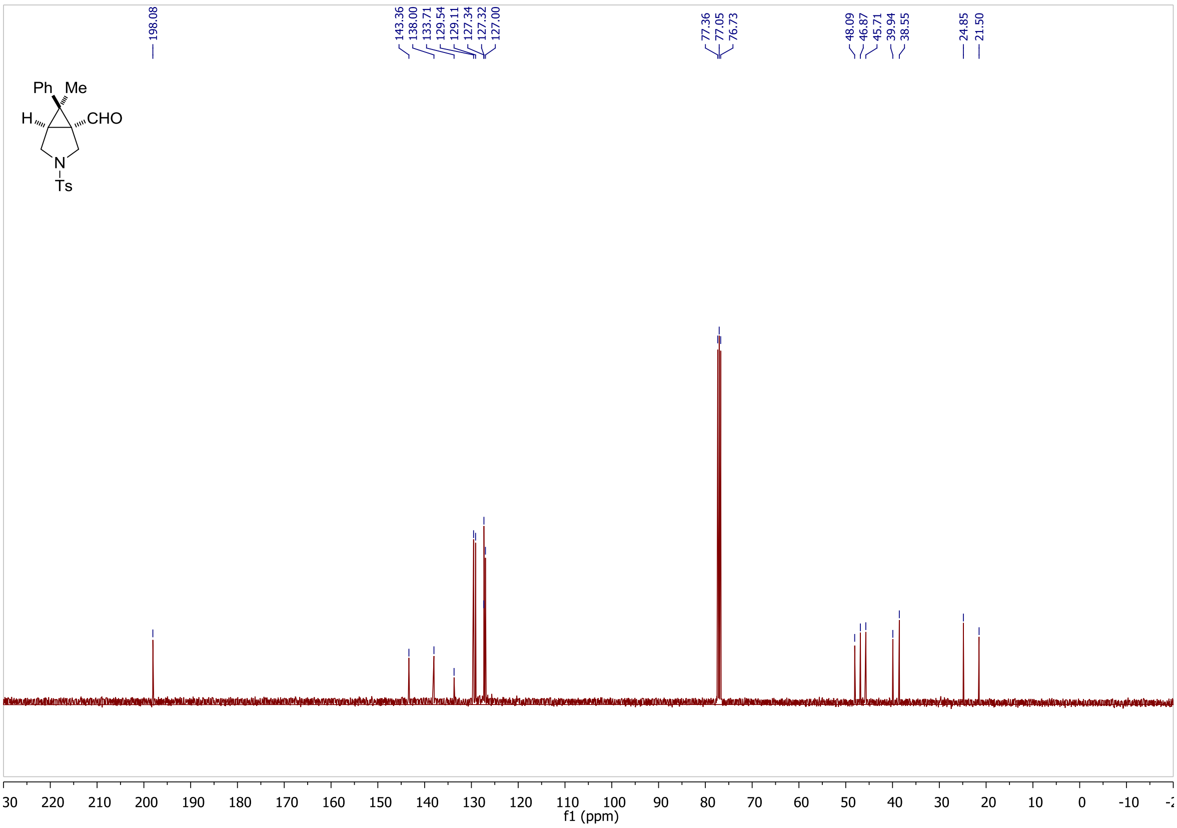

Supplementary Figure 64. ^{13}C NMR of the trans-**2o** (101 MHz, $CDCl_3$)

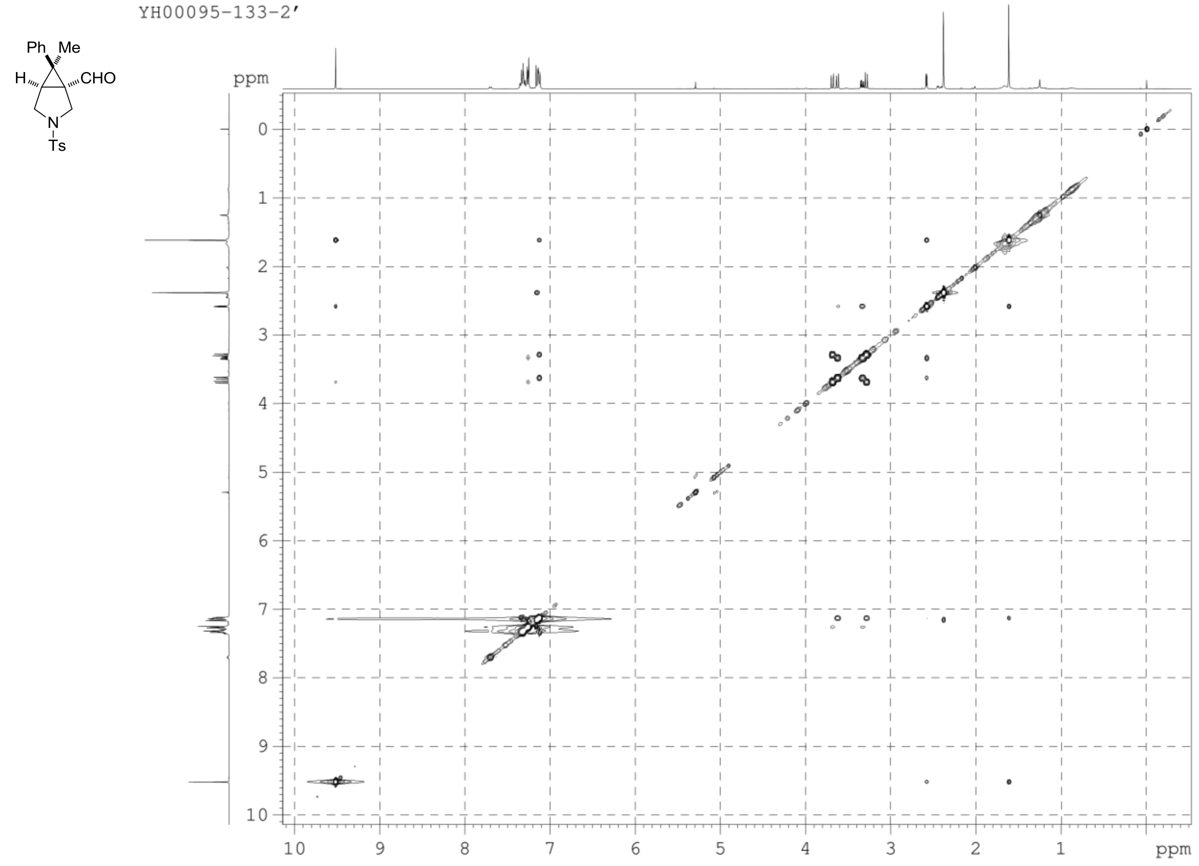

Supplementary Figure 65. NOESY of the trans-**2o** (101 MHz, $CDCl_3$)

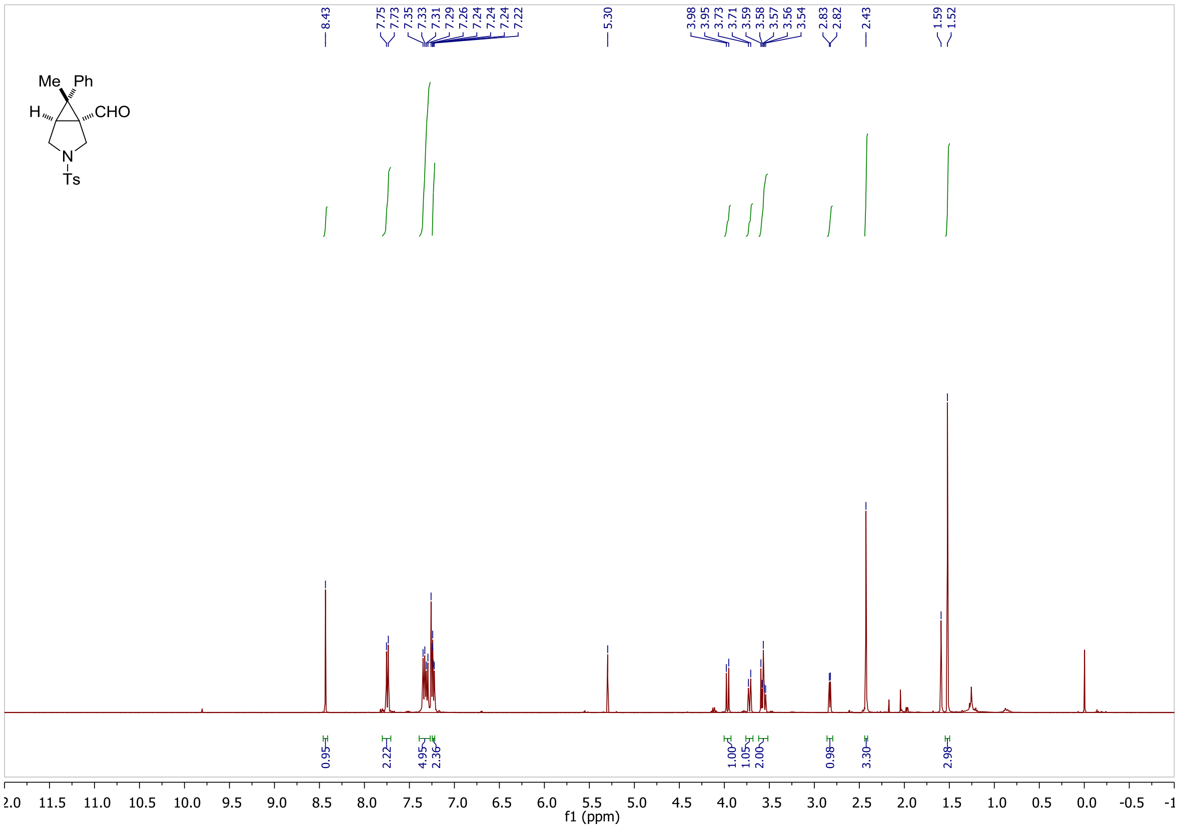

Supplementary Figure 66. ^{1}H NMR of the cis-**2o** (400 MHz, $CDCl_3$)

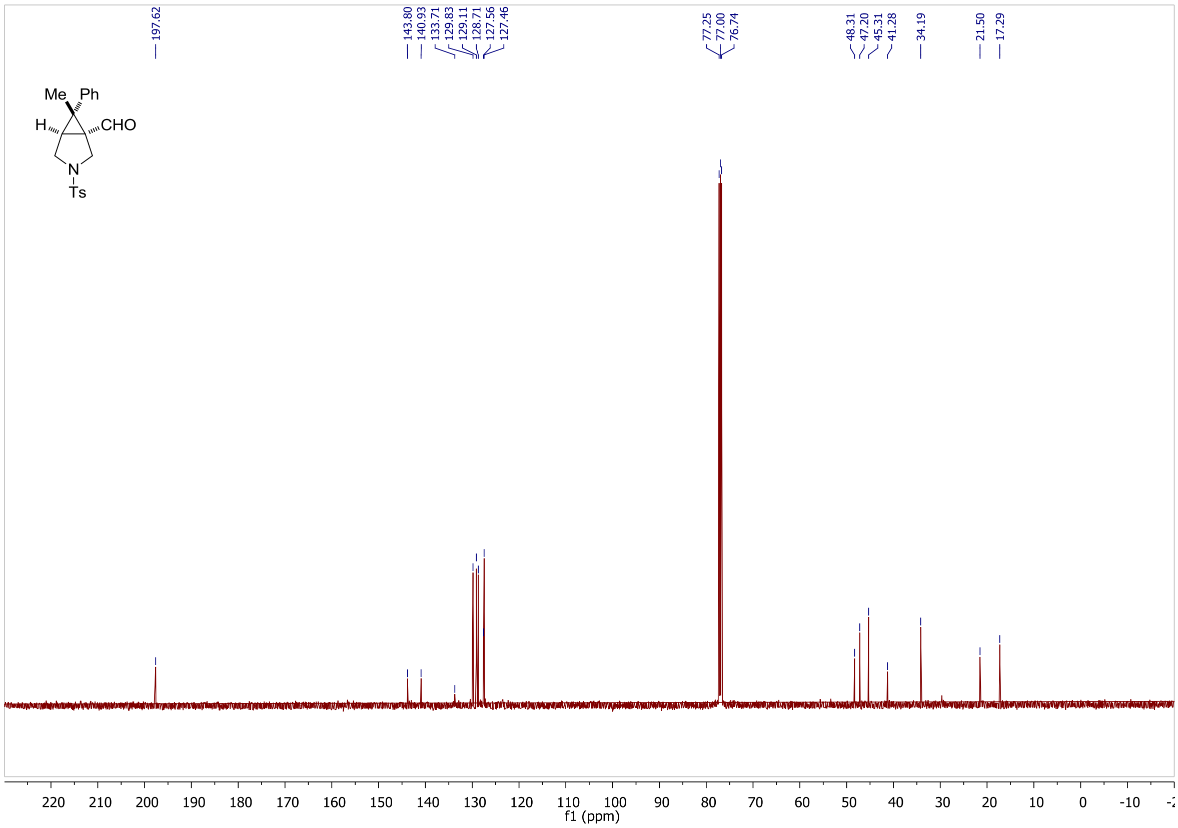

Supplementary Figure 67. ^{13}C NMR of the cis-**2o** (126 MHz, $CDCl_3$)

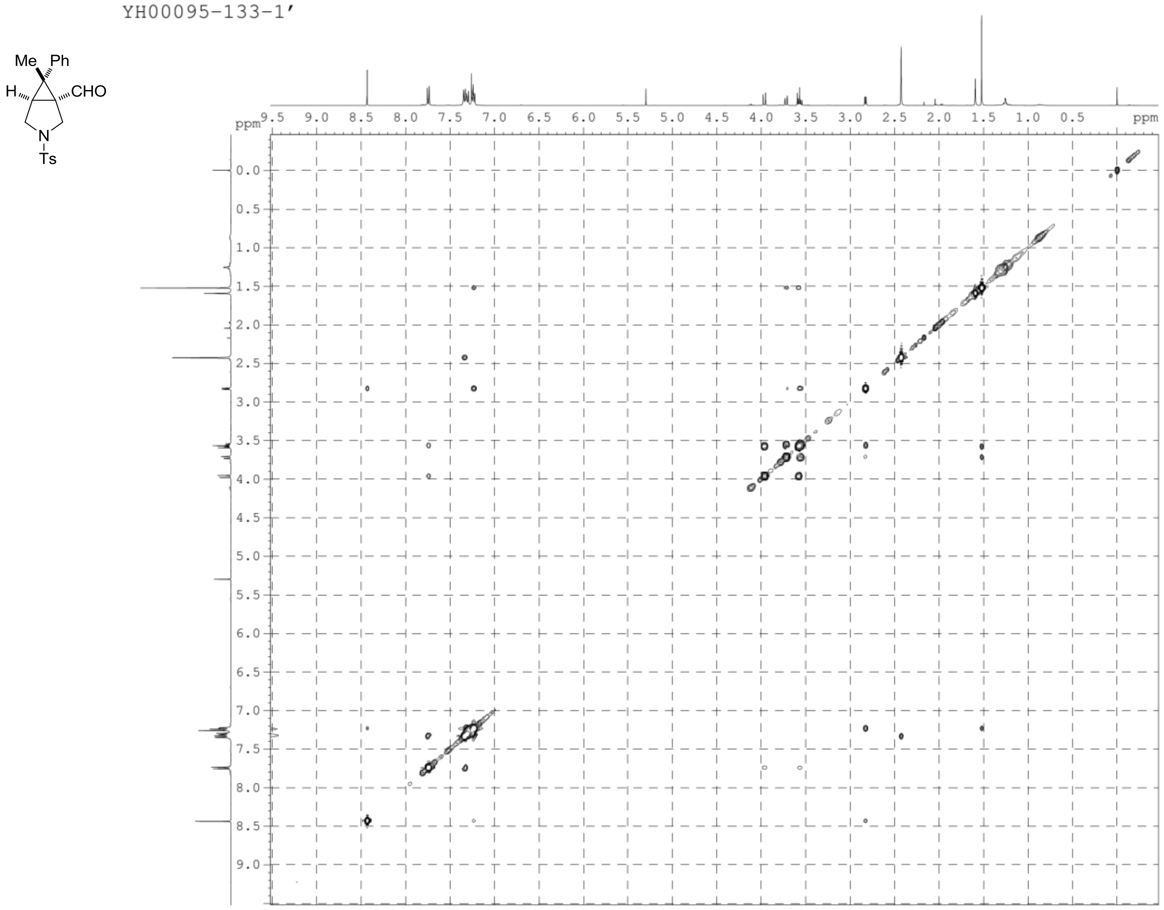

Supplementary Figure 68. NOESY of the cis-**2o** (101 MHz, $CDCl_3$)

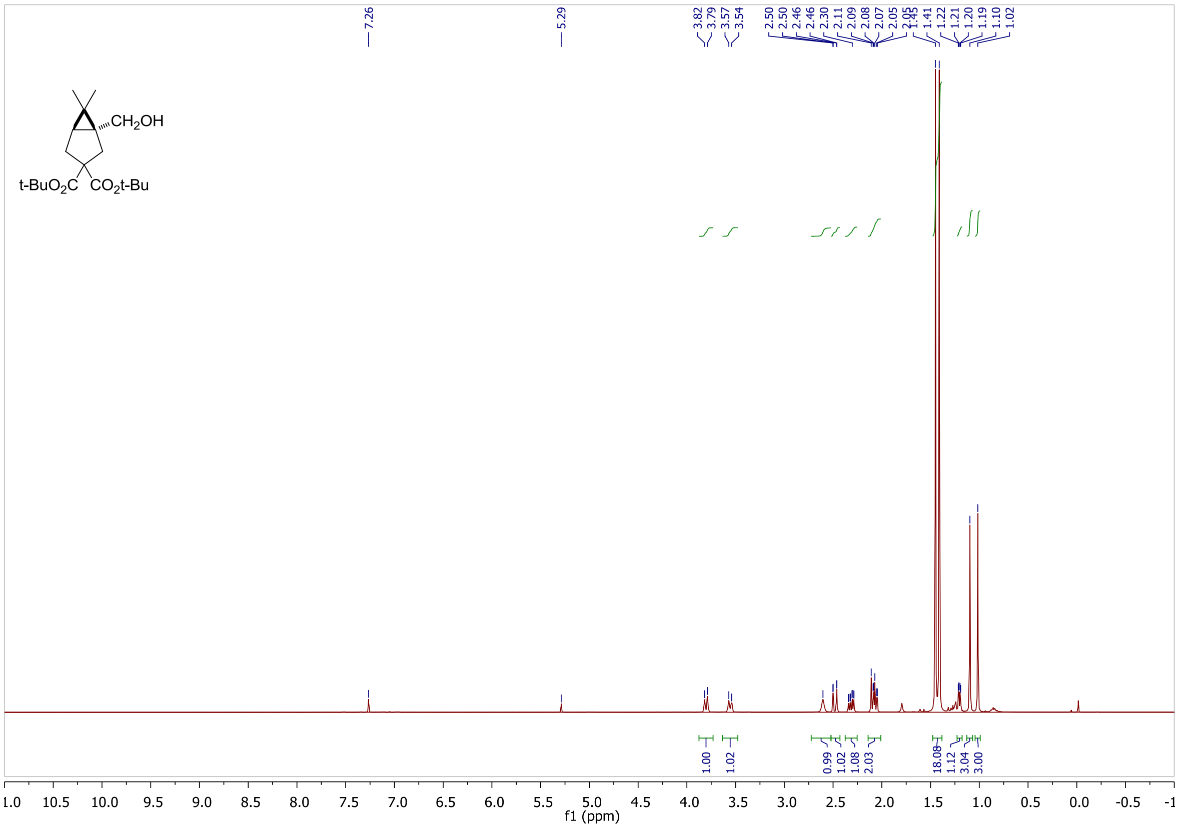

Supplementary Figure 69. ^{1}H NMR of the **5b** (400 MHz, $CDCl_3$)

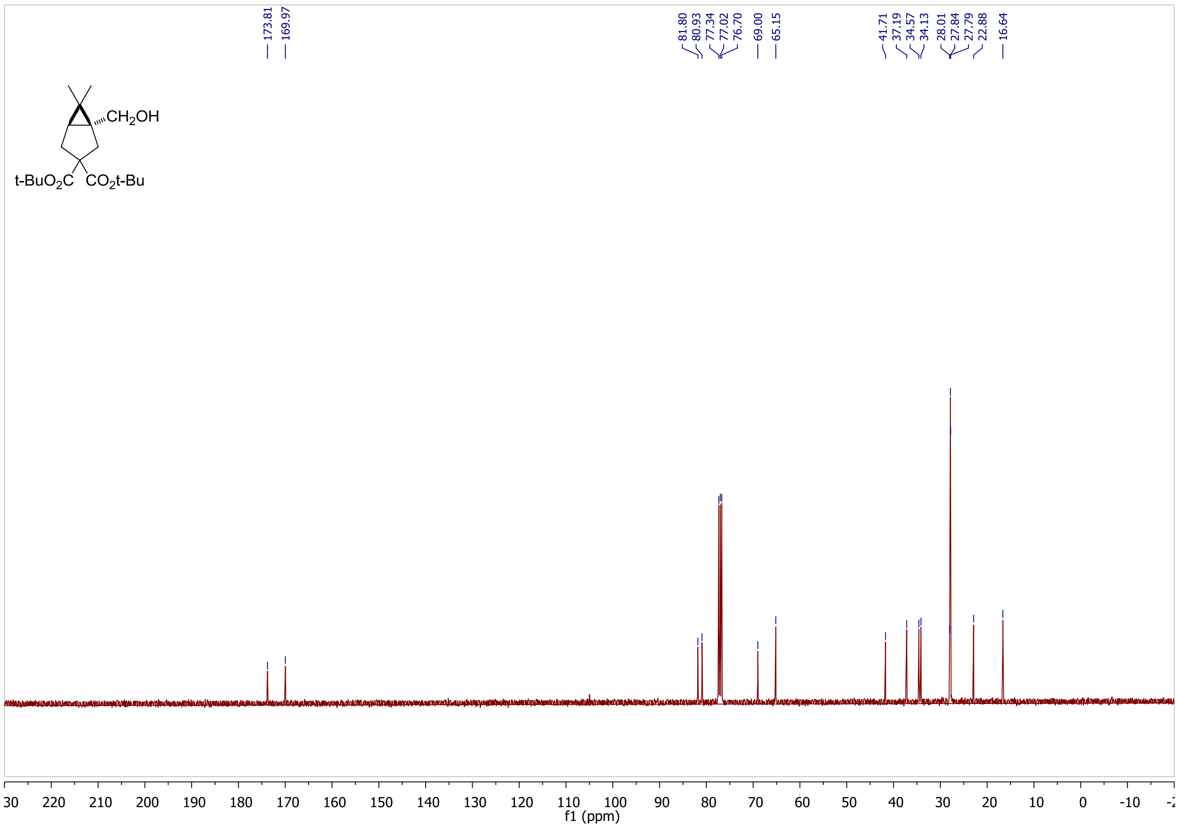

Supplementary Figure 70. ^{13}C NMR of the **5b** (101 MHz, $CDCl_3$)

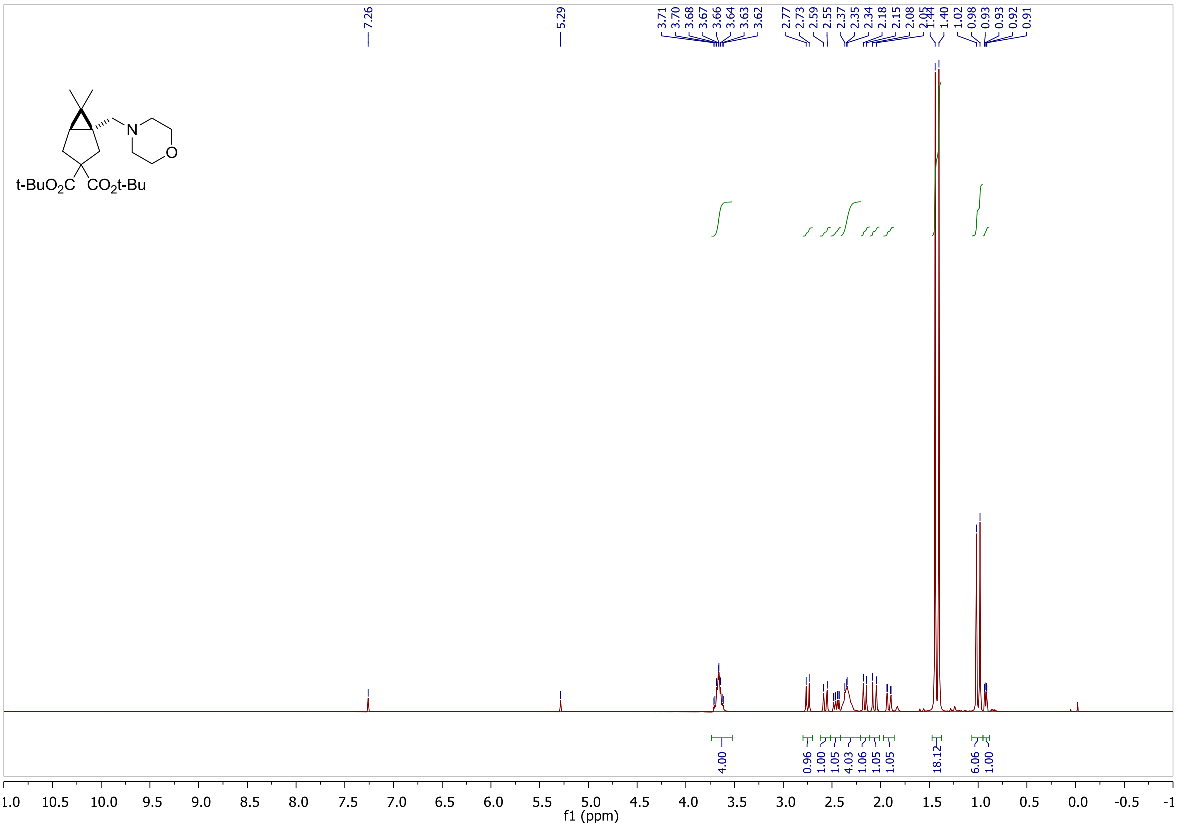

Supplementary Figure 71. ^{1}H NMR of the **6b** (400 MHz, $CDCl_3$)

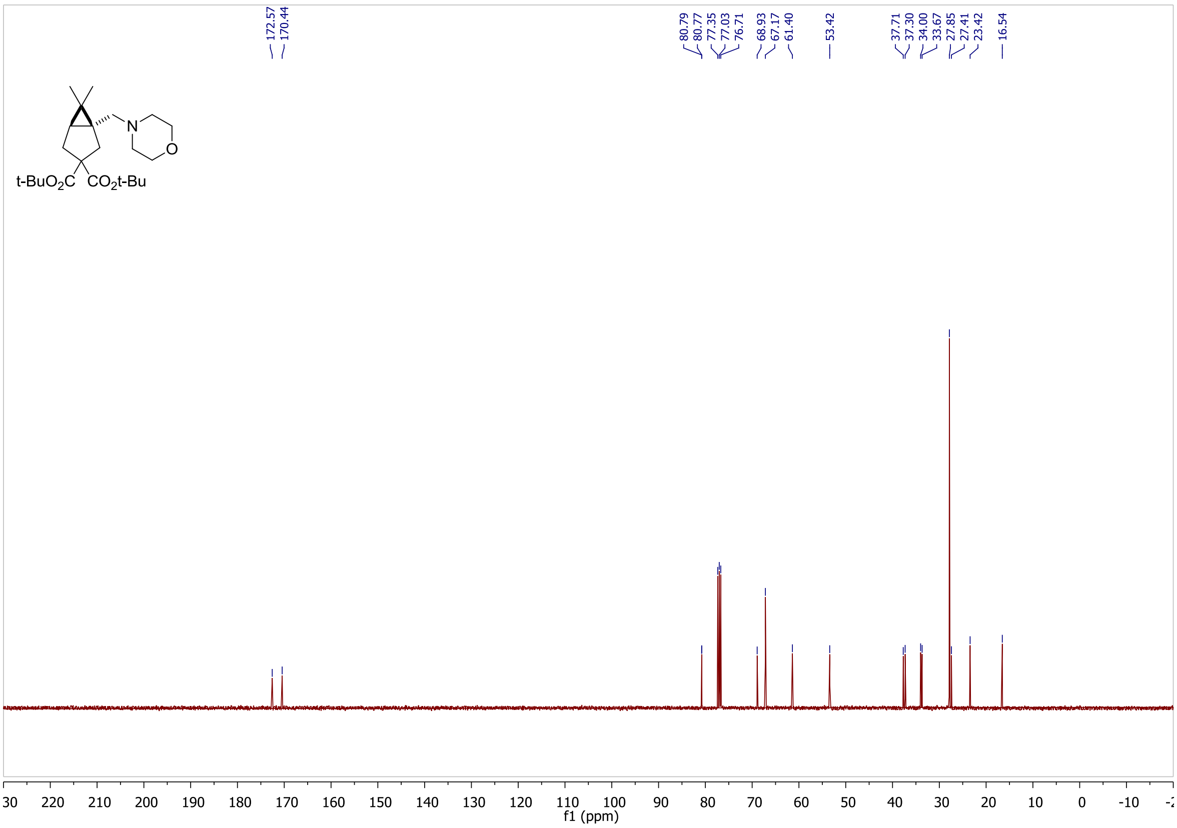

Supplementary Figure 72. ^{13}C NMR of the **6b** (101 MHz, $CDCl_3$)

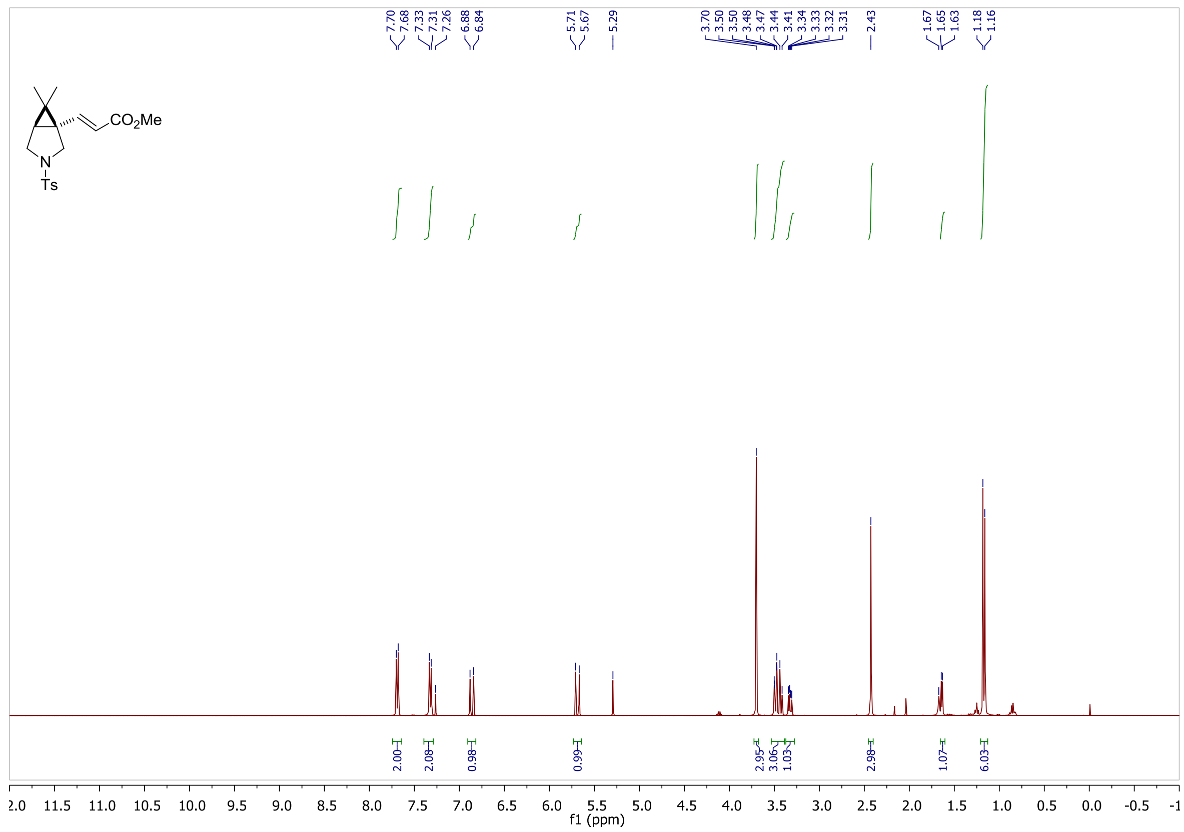

Supplementary Figure 73. ^{1}H NMR of the **7c** (400 MHz, $CDCl_3$)

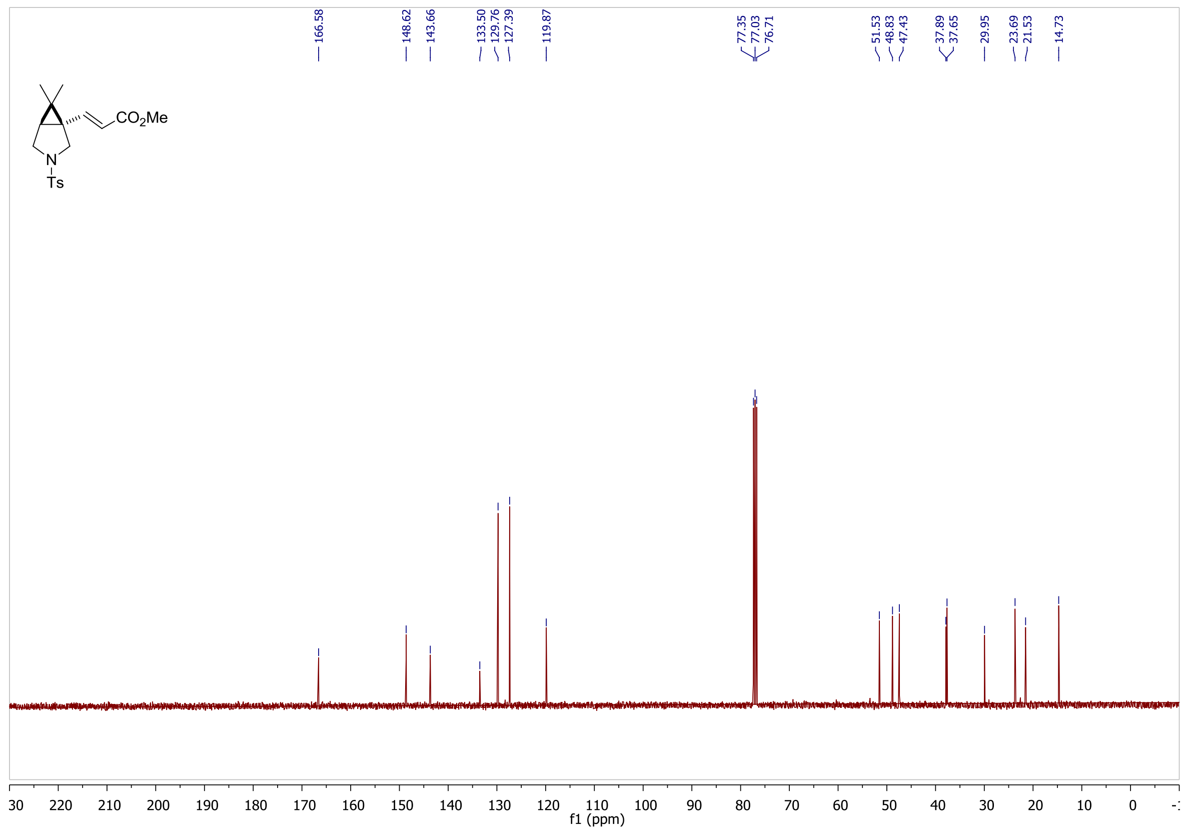

Supplementary Figure 74. ^{13}C NMR of the **7c** (101 MHz, $CDCl_3$)

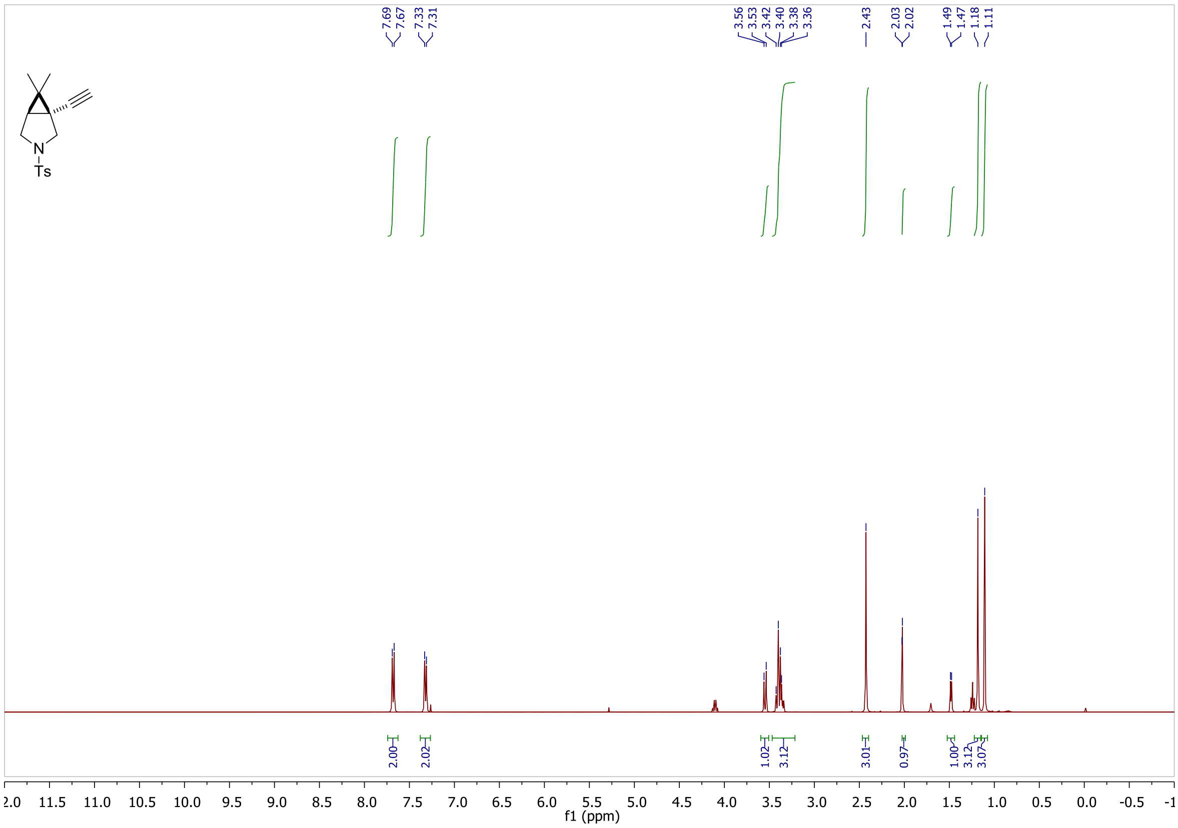

Supplementary Figure 75. ^{1}H NMR of the **8c** (400 MHz, $CDCl_3$)

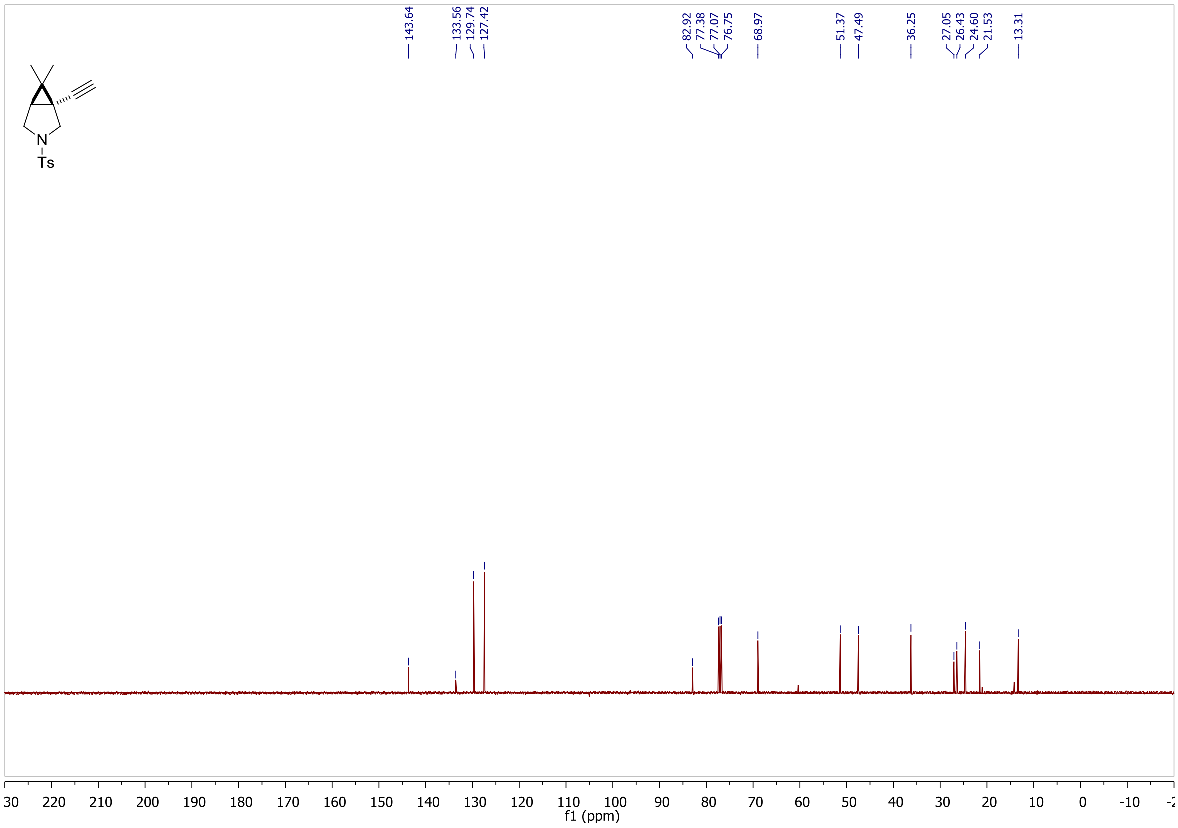

Supplementary Figure 76. ^{13}C NMR of the **8c** (101 MHz, $CDCl_3$)

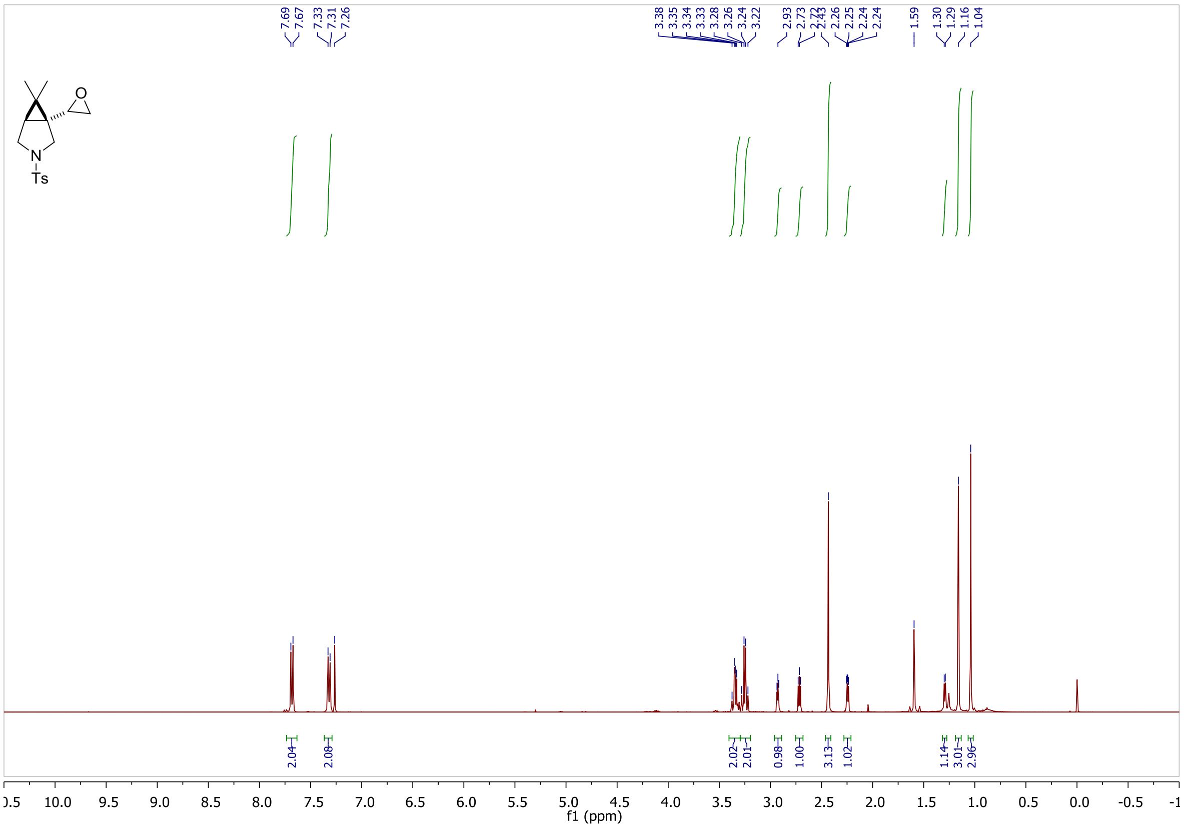

Supplementary Figure 77. ^{1}H NMR of the **9c** (400 MHz, $CDCl_3$)

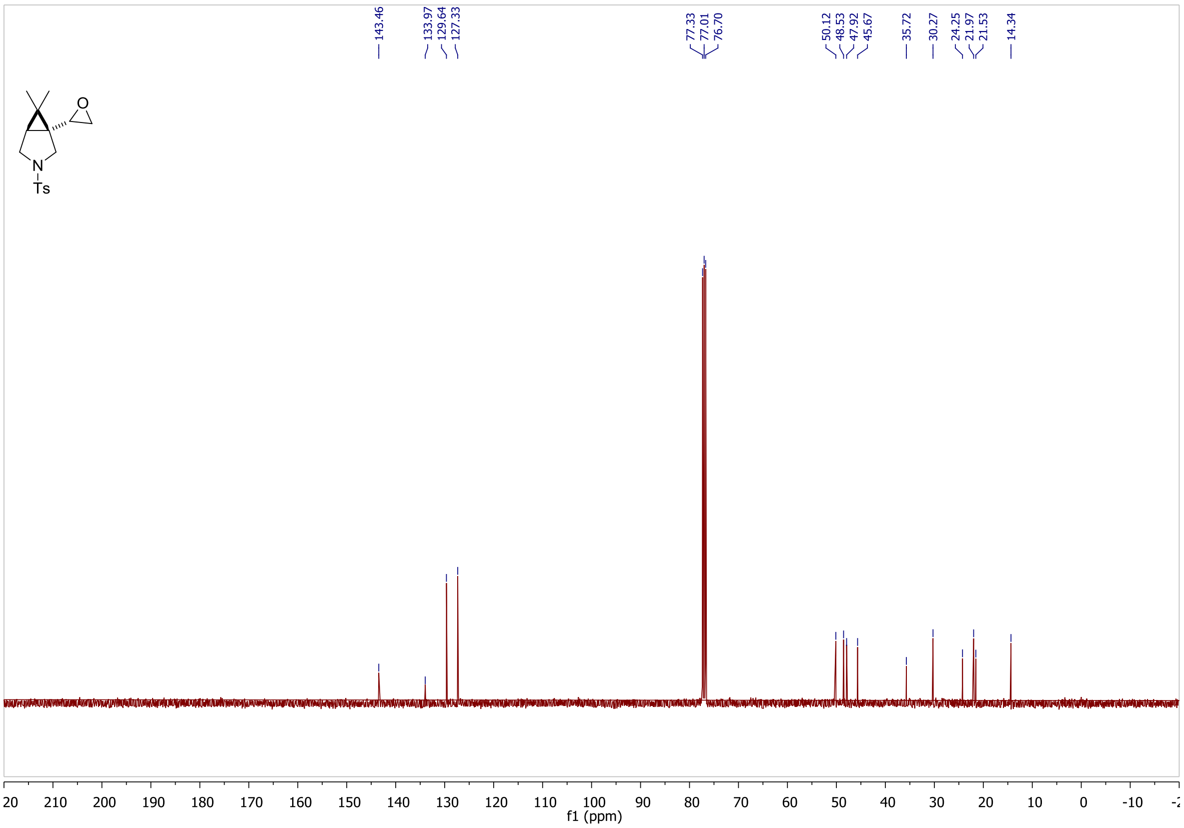

Supplementary Figure 78. ^{13}C NMR of the **9c** (101 MHz, $CDCl_3$)

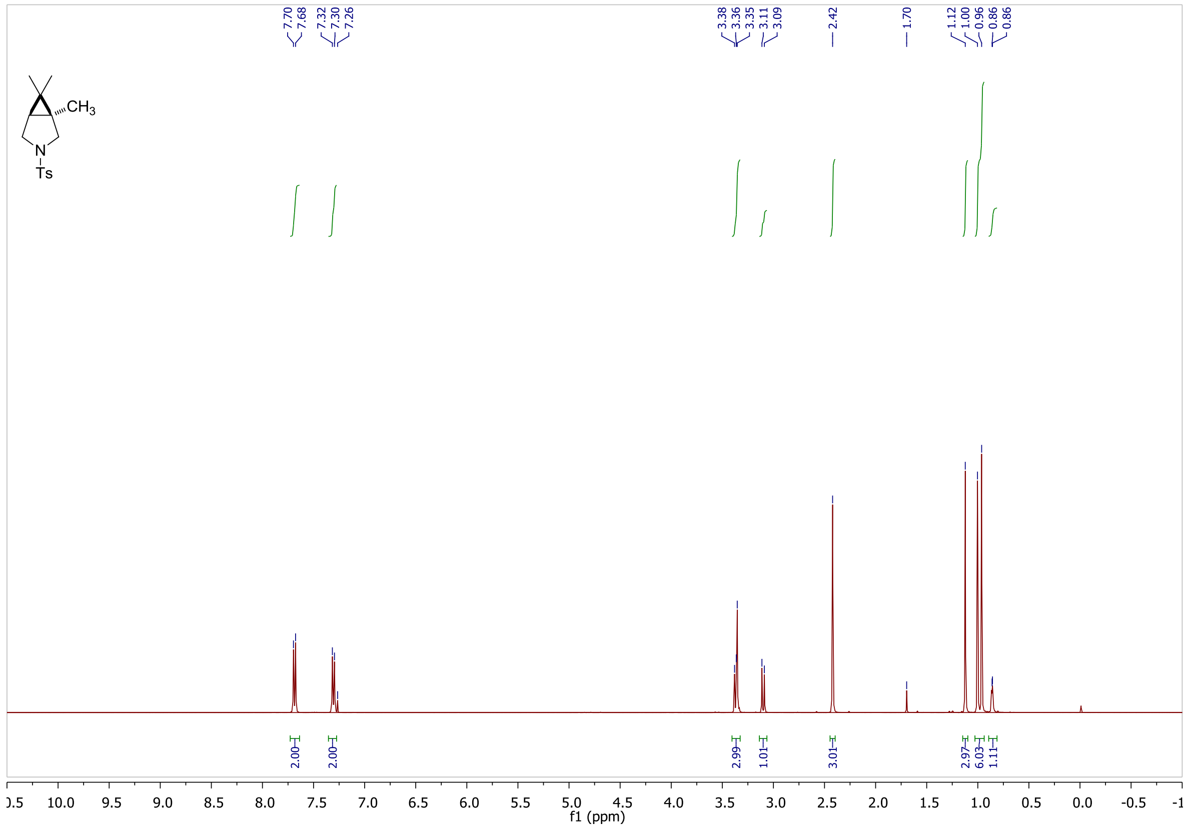

Supplementary Figure 79. ^{1}H NMR of the **10c** (400 MHz, $CDCl_3$)

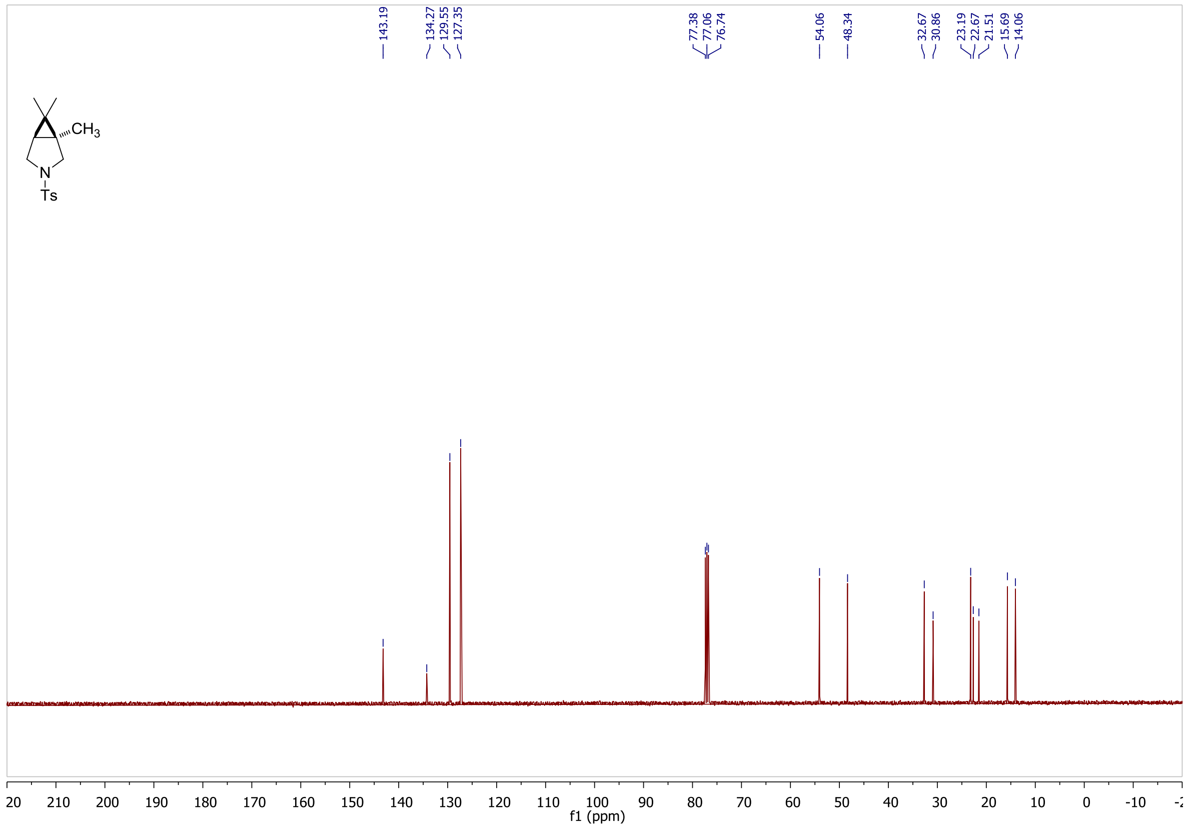

Supplementary Figure 80. ^{13}C NMR of the **10c** (101 MHz, $CDCl_3$)

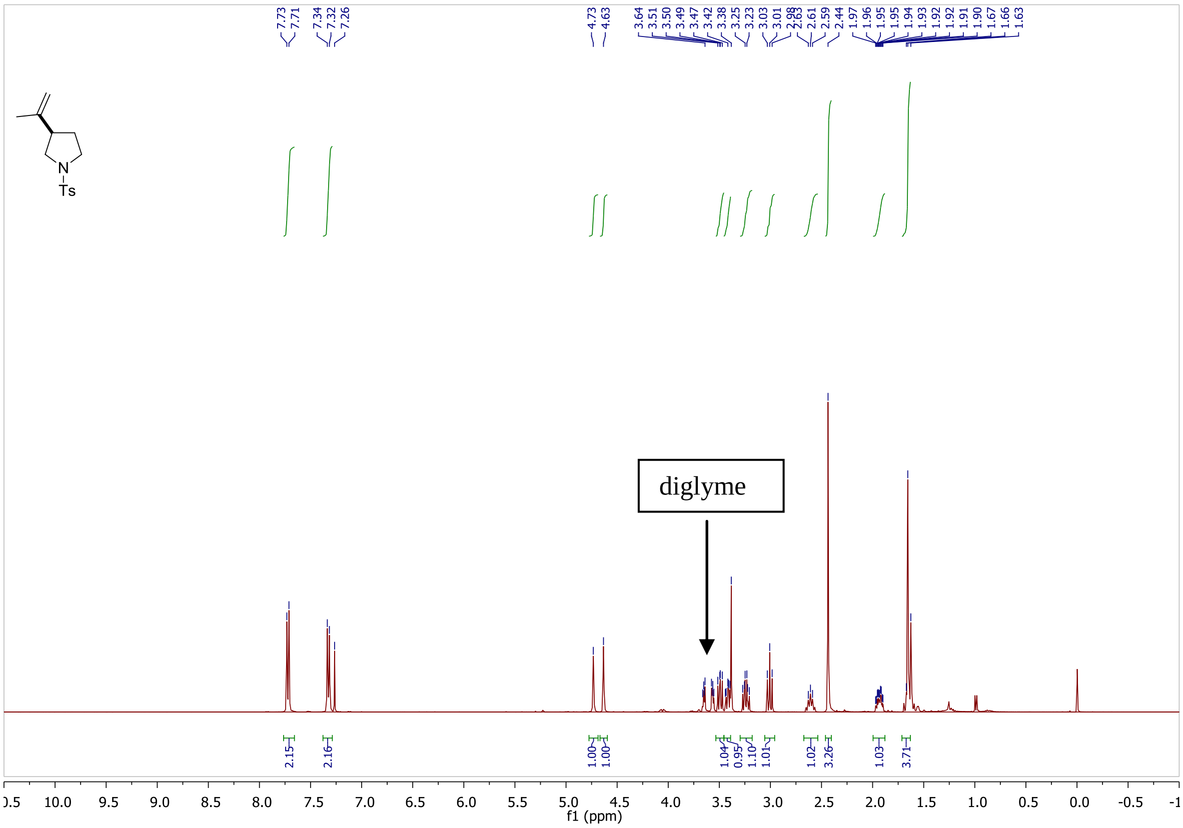

Supplementary Figure 81. ^{1}H NMR of the **11c** (400 MHz, $CDCl_3$)

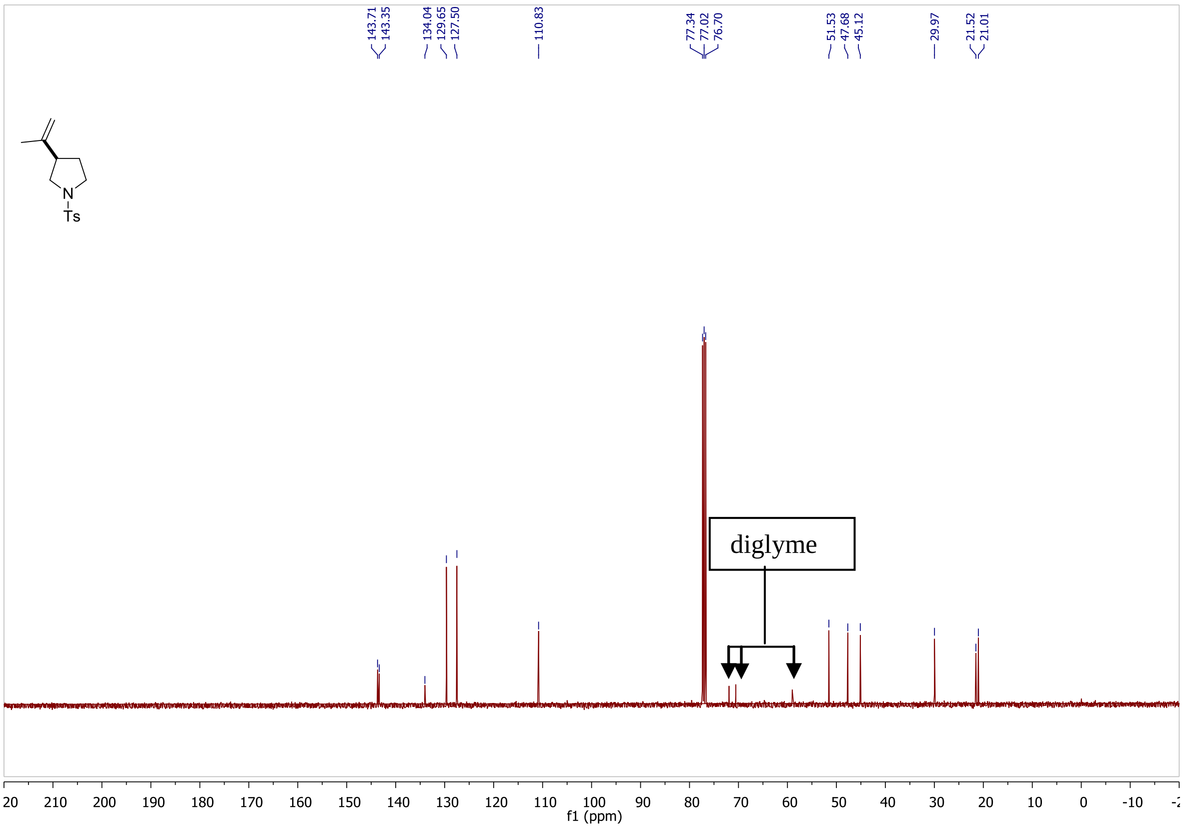

Supplementary Figure 82. ^{13}C NMR of the **11c** (101 MHz, $CDCl_3$)

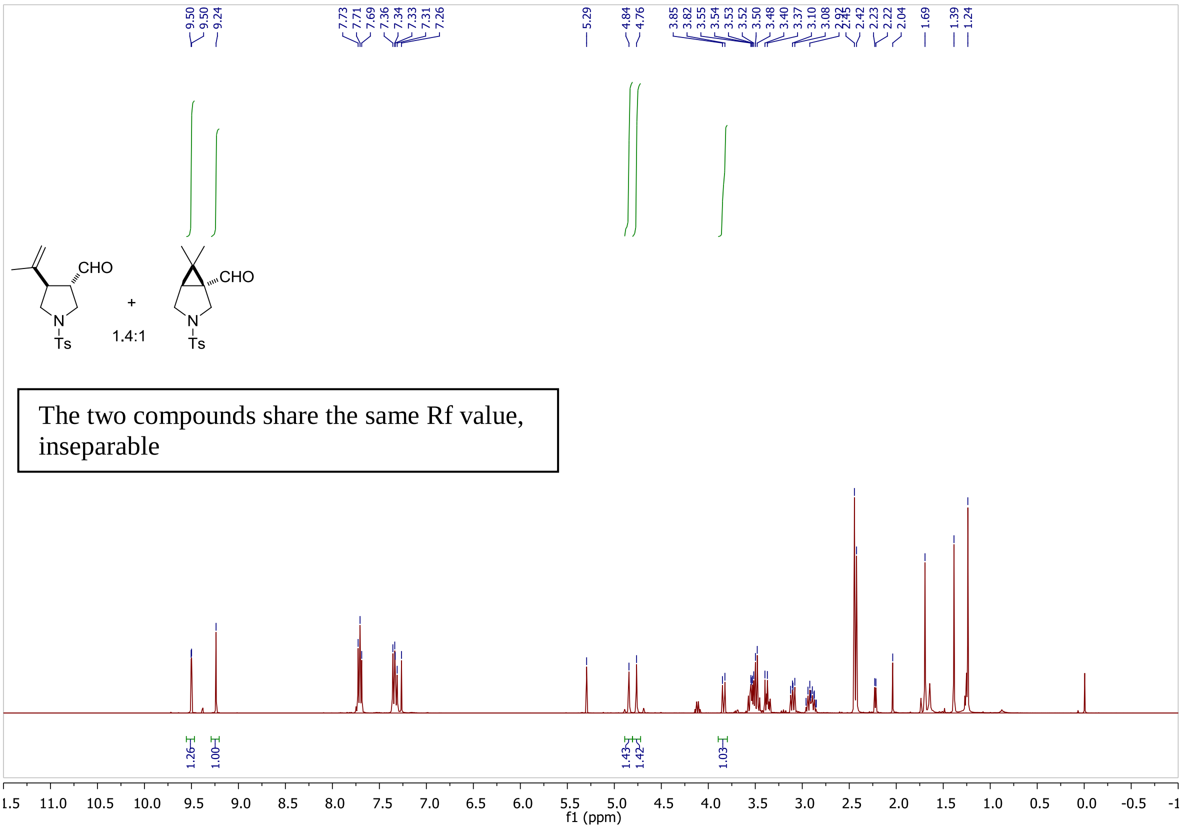

Supplementary Figure 83. ^{1}H NMR of the **12c** (400 MHz, $CDCl_3$)

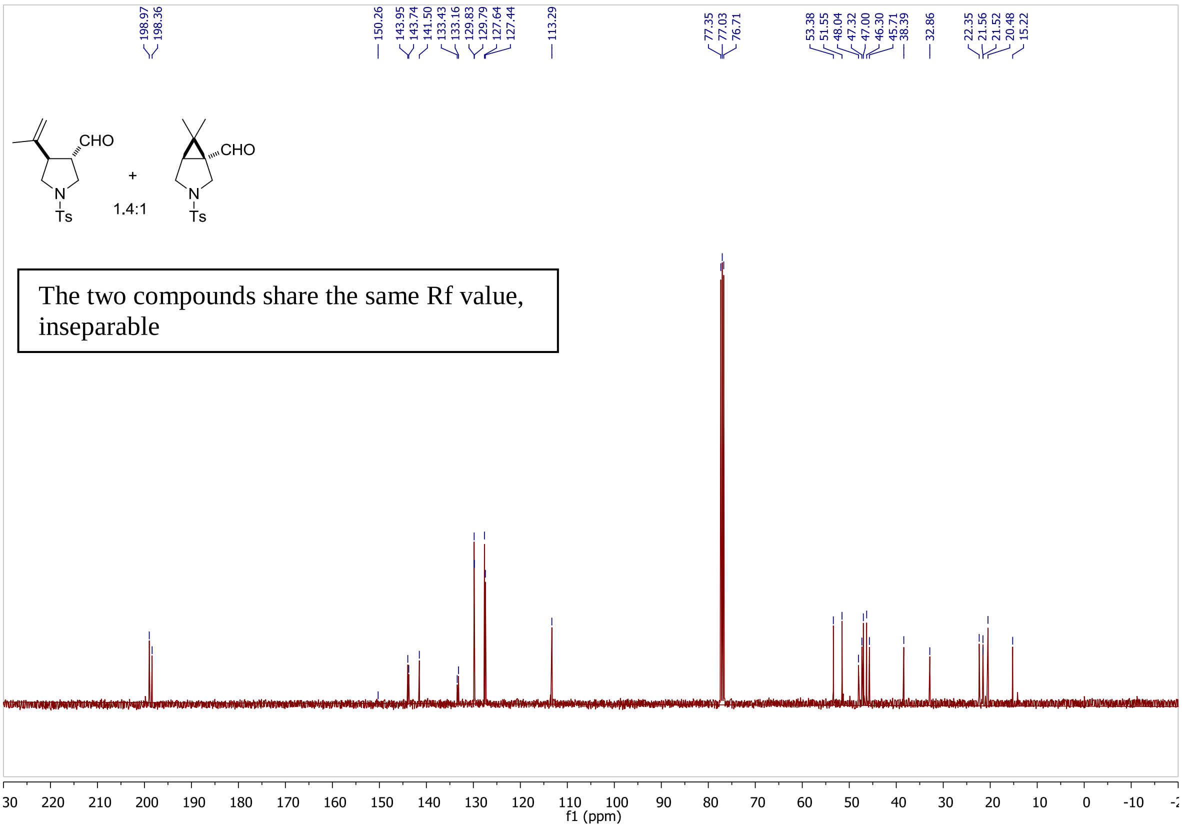

Supplementary Figure 84. ^{13}C NMR of the **12c** (101 MHz, $CDCl_3$)

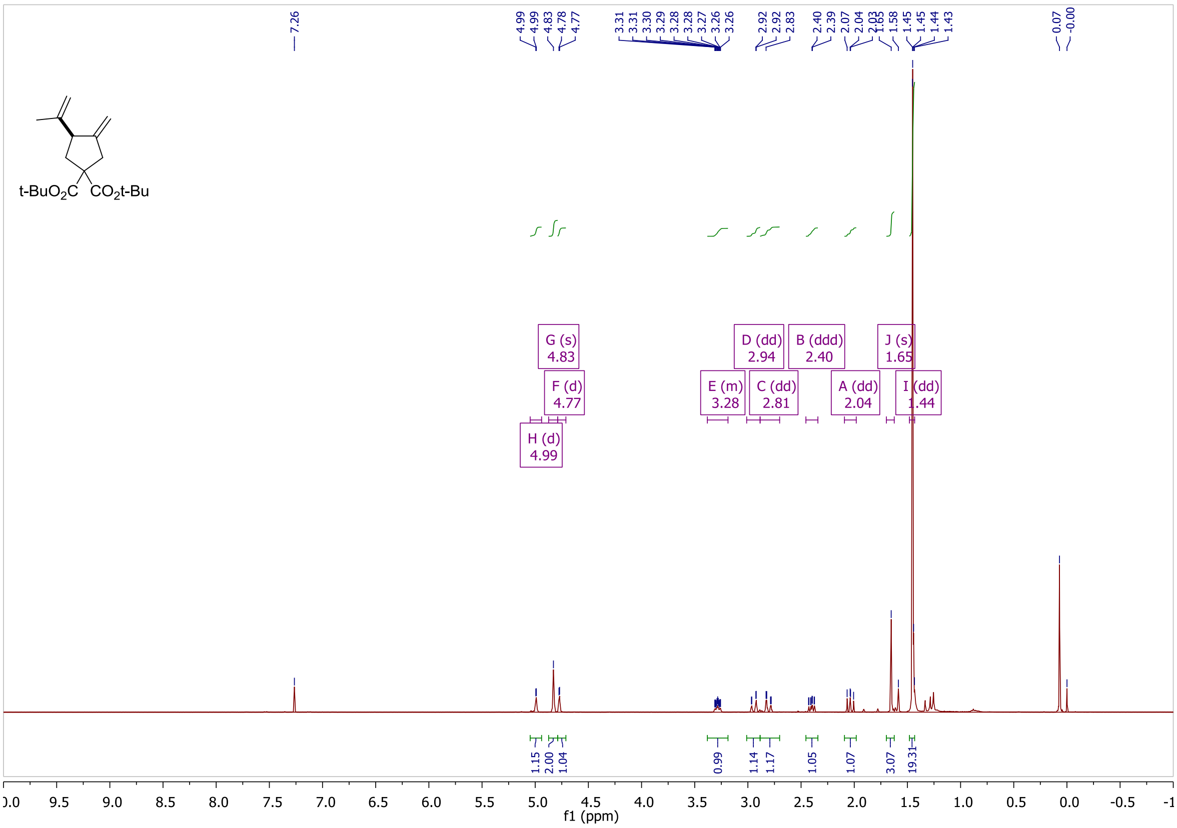

Supplementary Figure 85. ^{1}H NMR of the **13b** (400 MHz, $CDCl_3$)

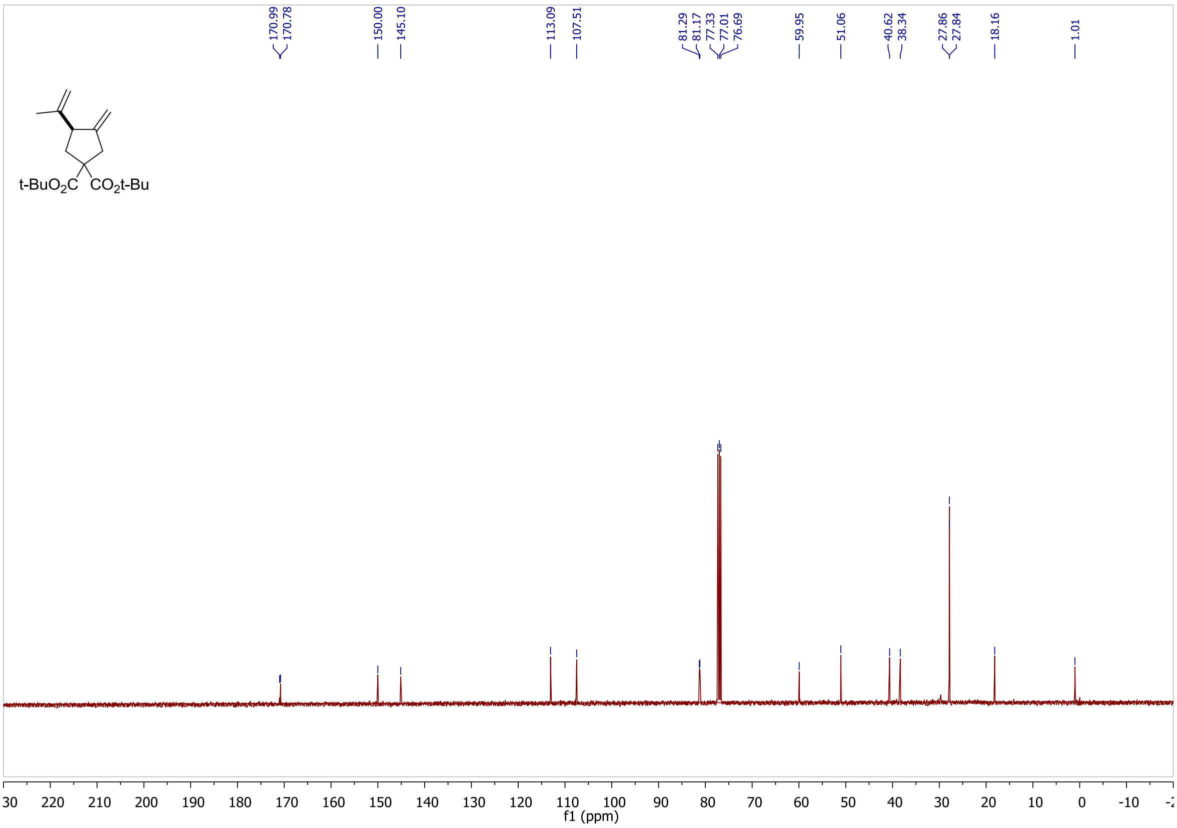

Supplementary Figure 86. ^{13}C NMR of the **13b** (101 MHz, $CDCl_3$)

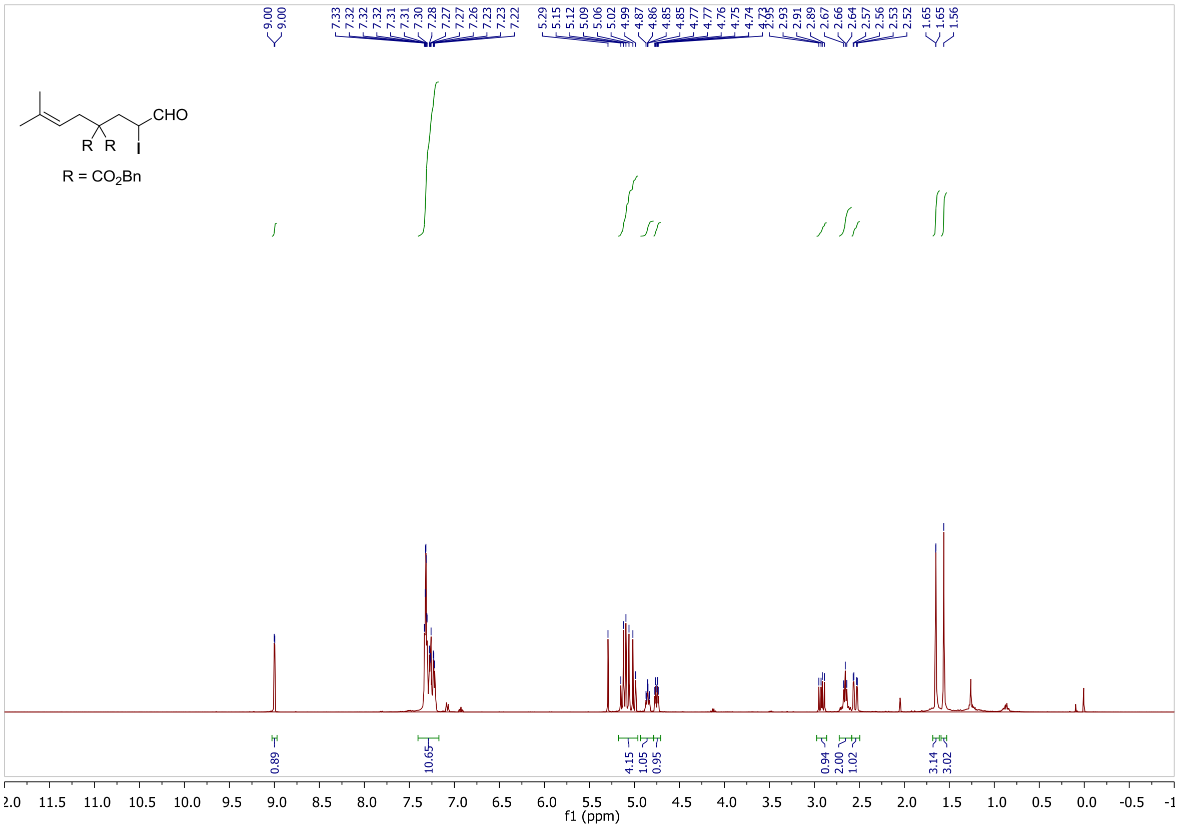

Supplementary Figure 87. ^{1}H NMR of the **4a** (400 MHz, $CDCl_3$)

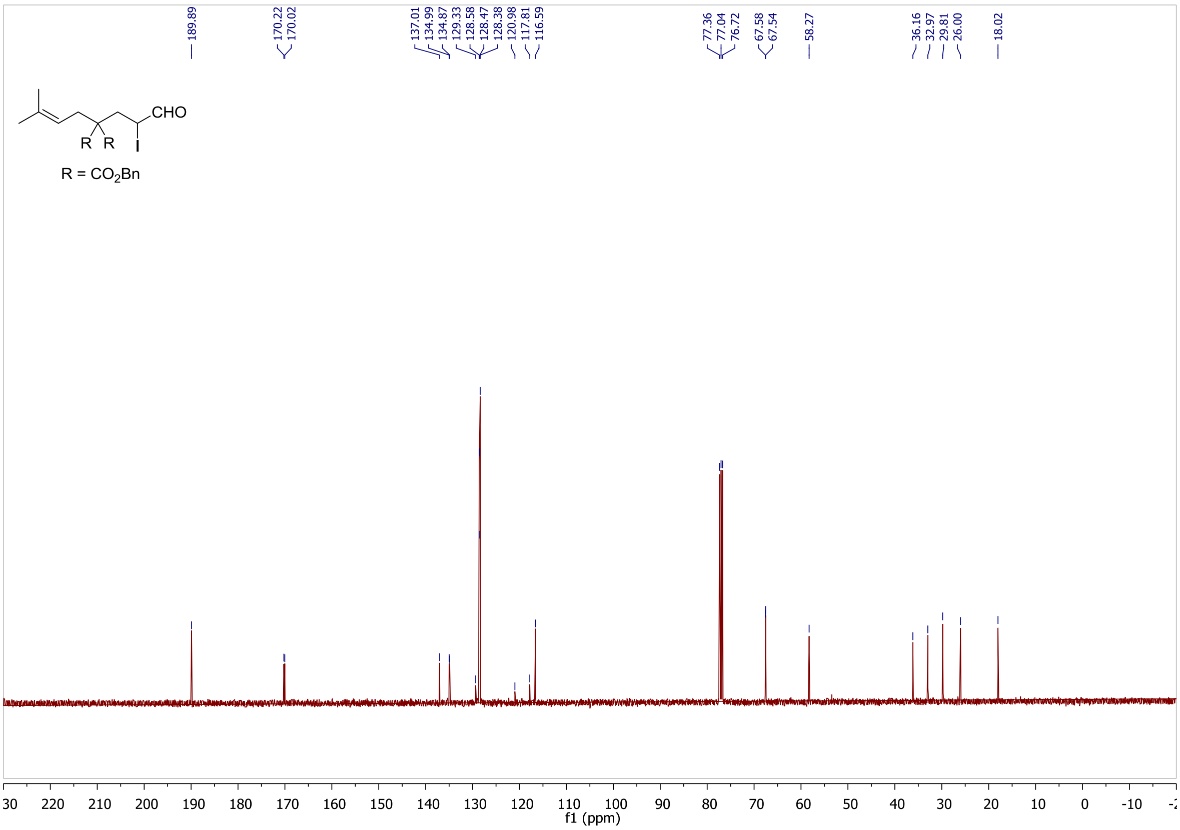

Supplementary Figure 88. ^{13}C NMR of the **4a** (101 MHz, $CDCl_3$)

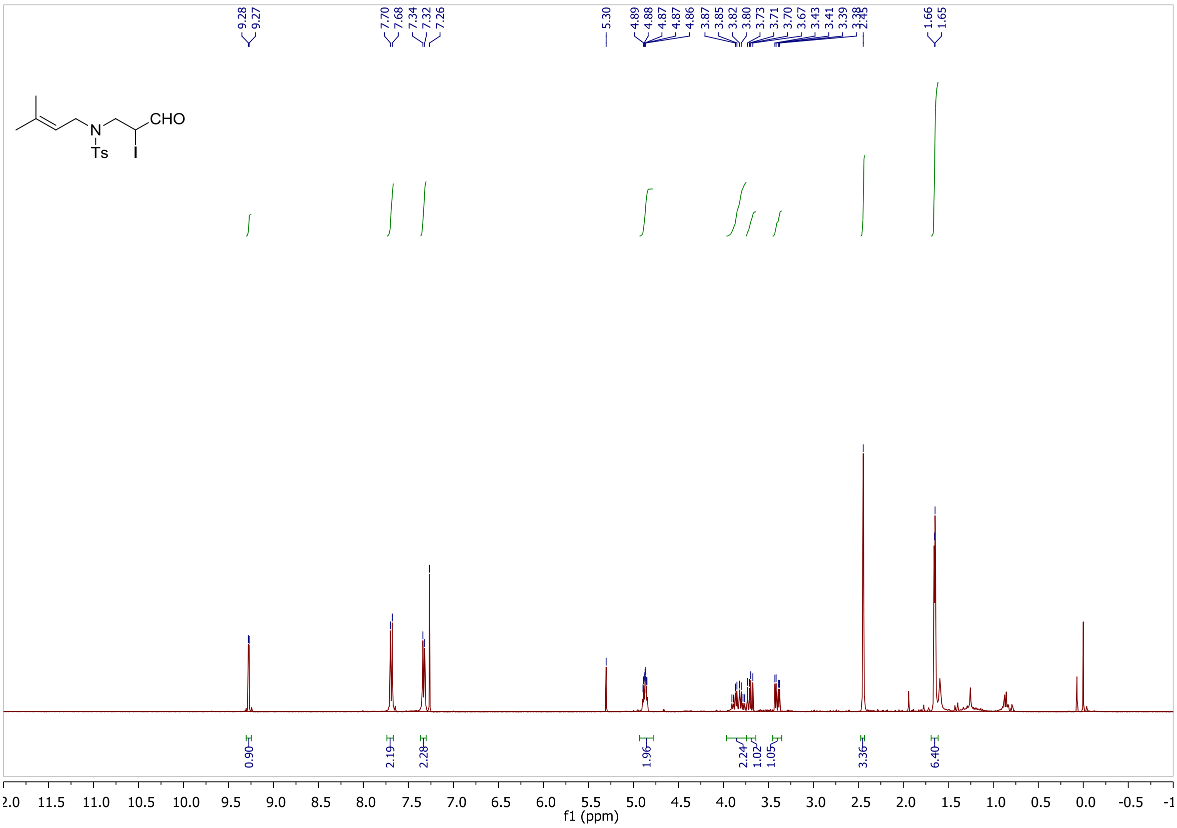

Supplementary Figure 89. ^{1}H NMR of the **4c** (400 MHz, $CDCl_3$)

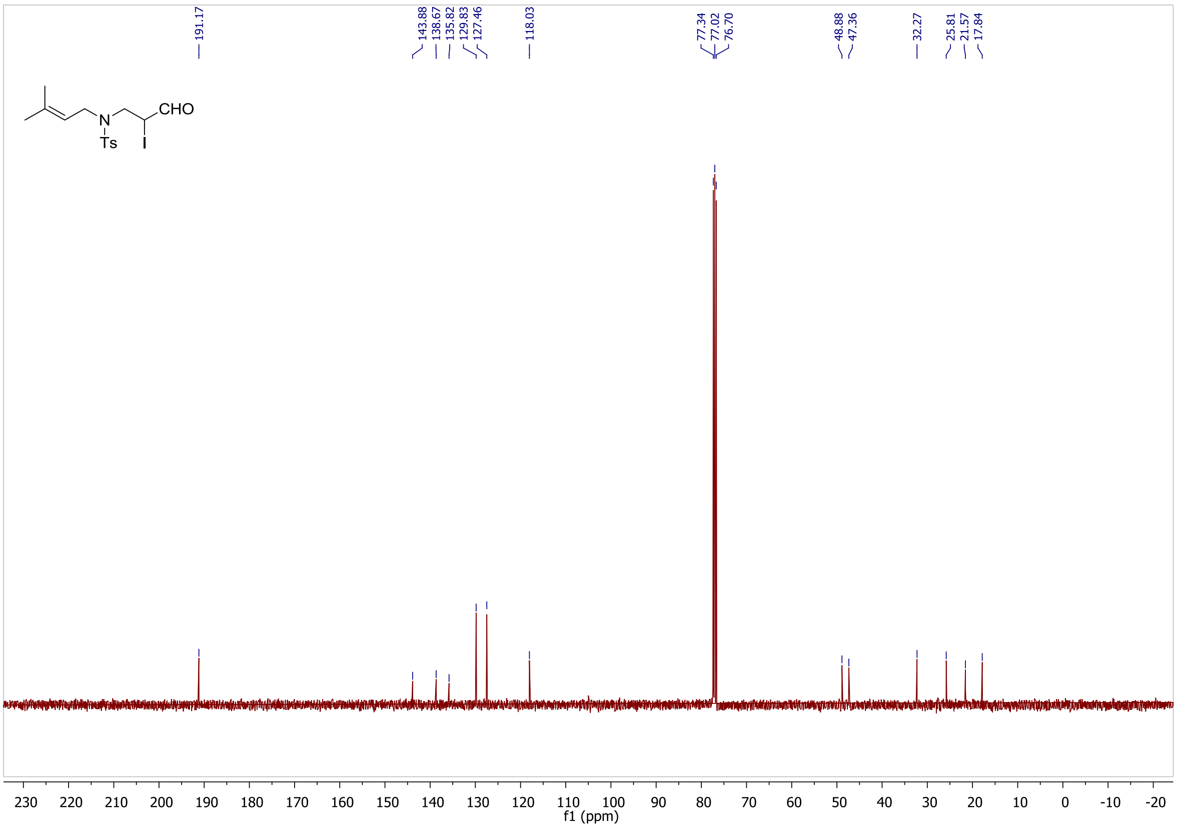

Supplementary Figure 90. ^{13}C NMR of the **4c** (101 MHz, $CDCl_3$)

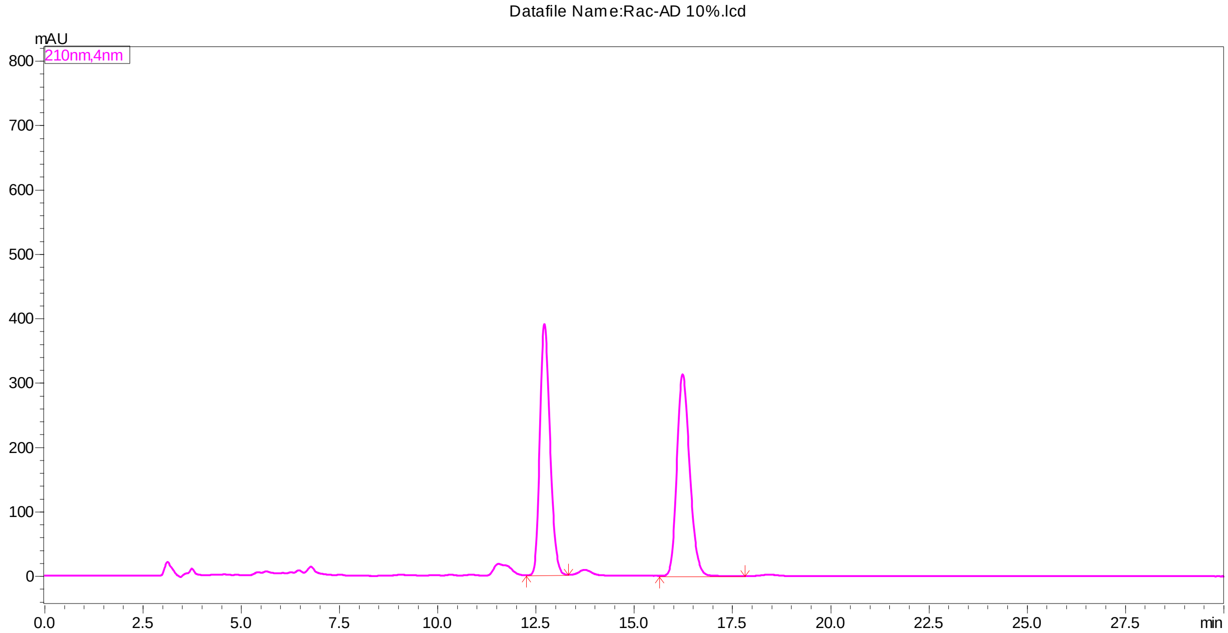

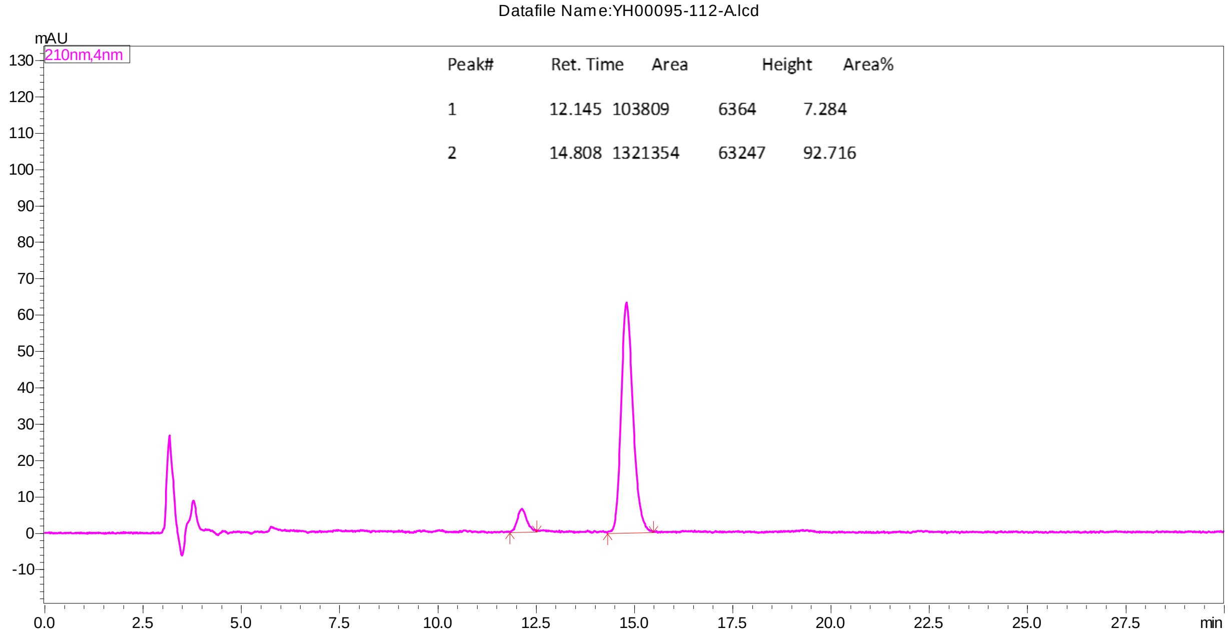

Peak#	Ret. Time	Area	Height	Area%
1	12.145	103809	6364	7.284
2	14.808	1321354	63247	92.716

Supplementary Figure 91. HPLC traces for racemic and chiral product **2a**

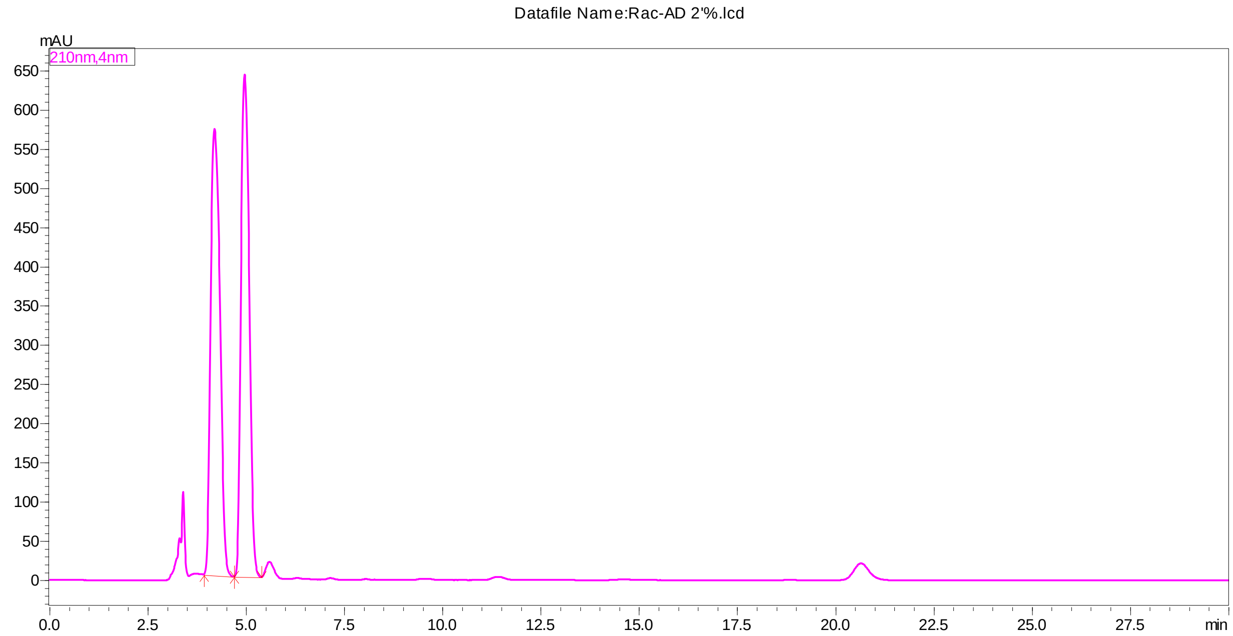

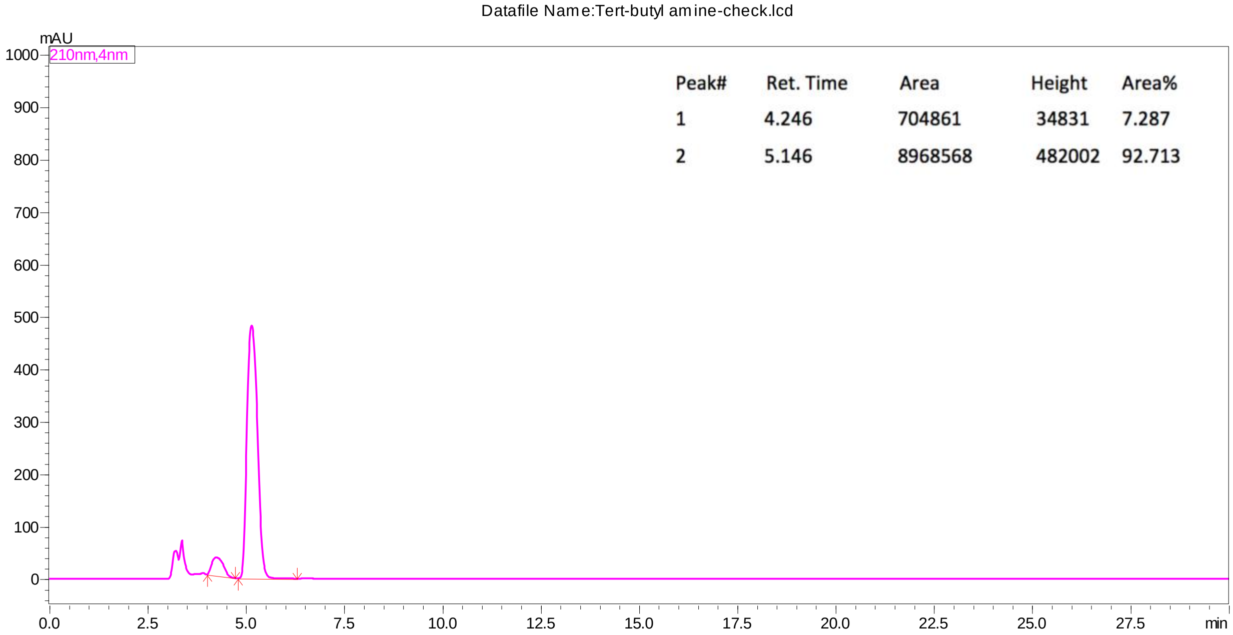

Supplementary Figure 92. HPLC traces for racemic and chiral product **2b**

Datafile Name:Rac-AD-80%.lcd

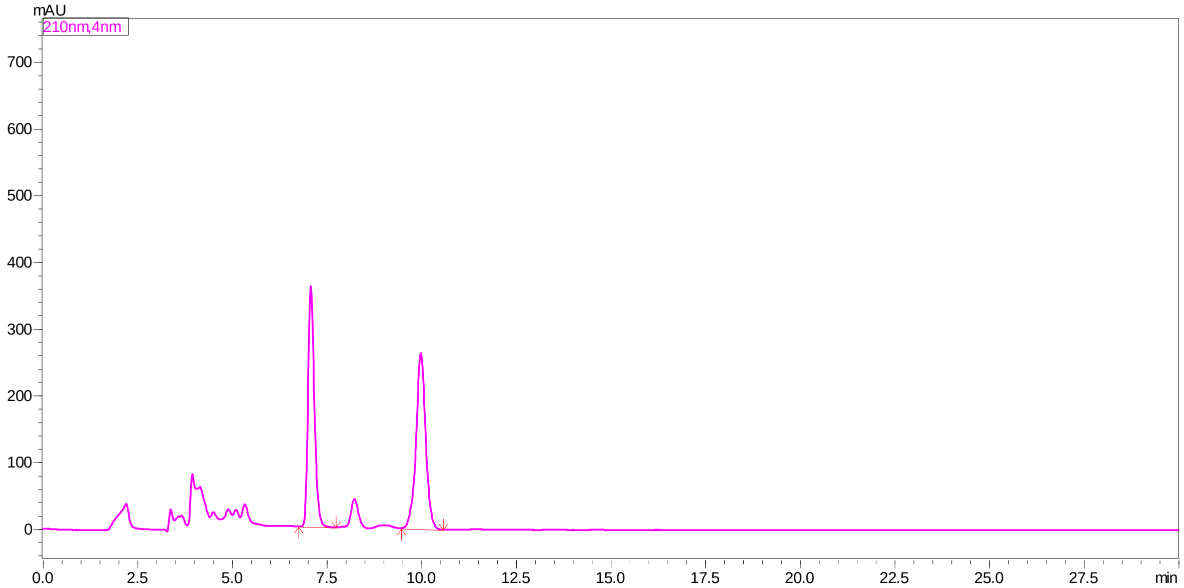

Datafile Name:Amine-tert-butyl-NIH'.lcd

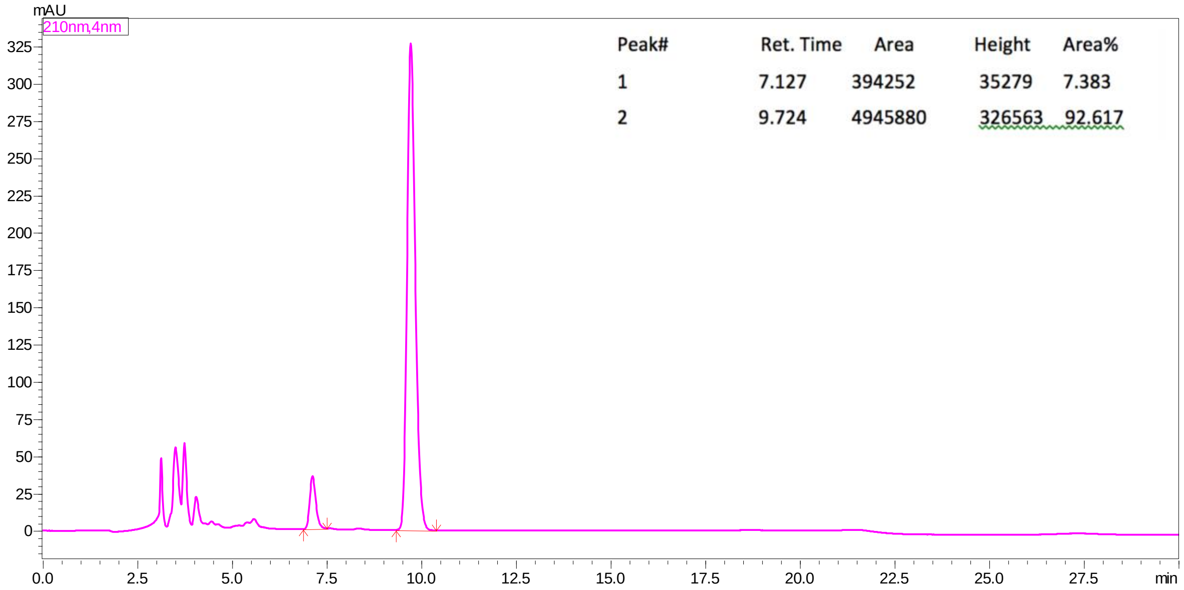

Peak#	Ret. Time	Area	Height	Area%
1	7.127	394252	35279	7.383
2	9.724	4945880	326563	92.617

Supplementary Figure 93. HPLC traces for racemic and chiral product **2c**

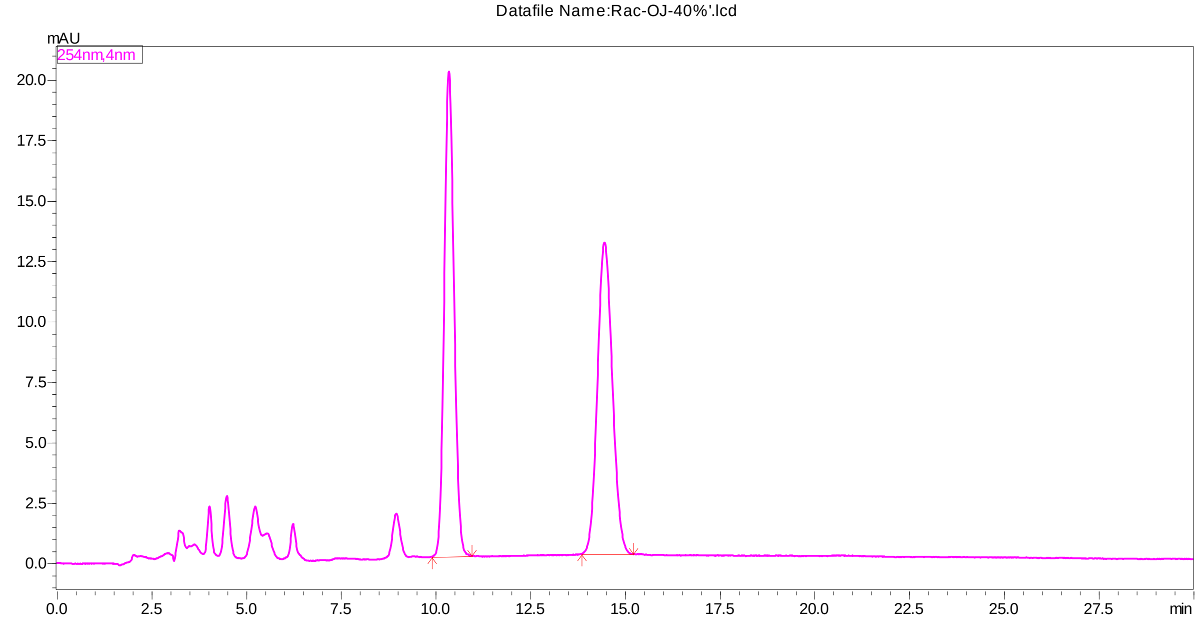

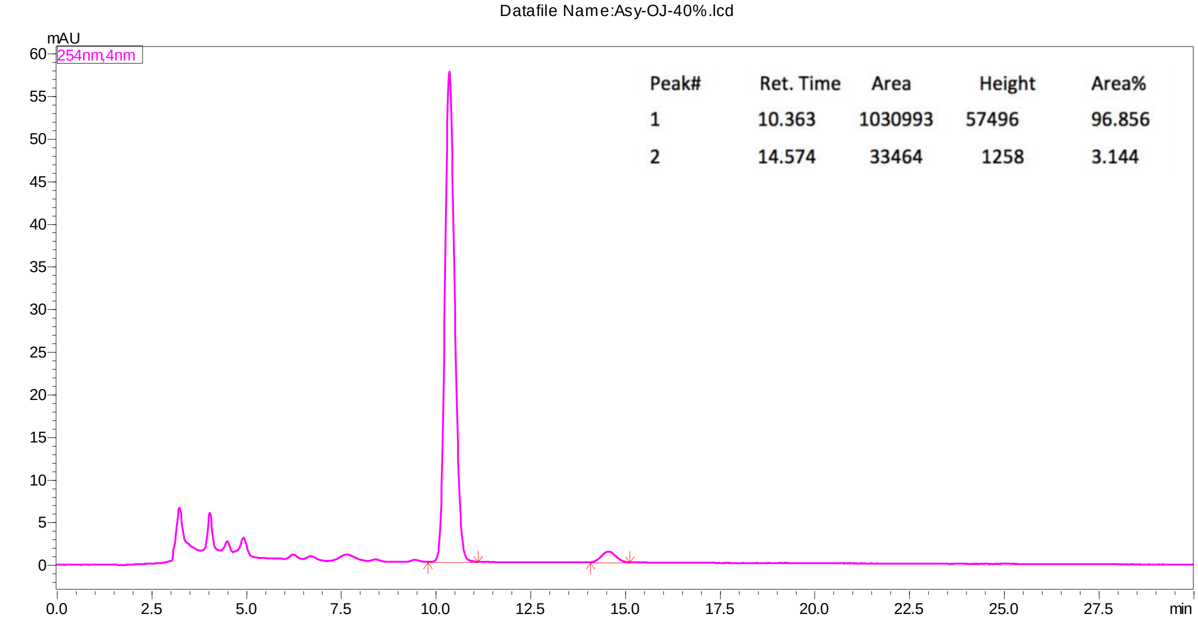

Peak#	Ret. Time	Area	Height	Area%
1	10.363	1030993	57496	96.856
2	14.574	33464	1258	3.144

Supplementary Figure 94. HPLC traces for racemic and chiral product **2d**

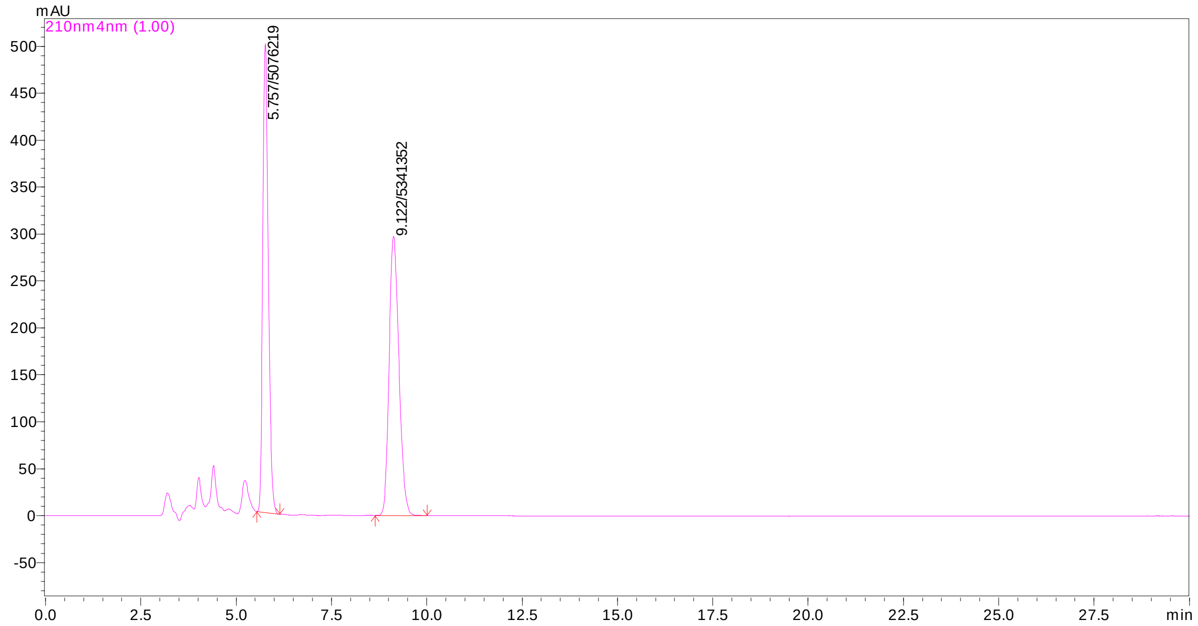

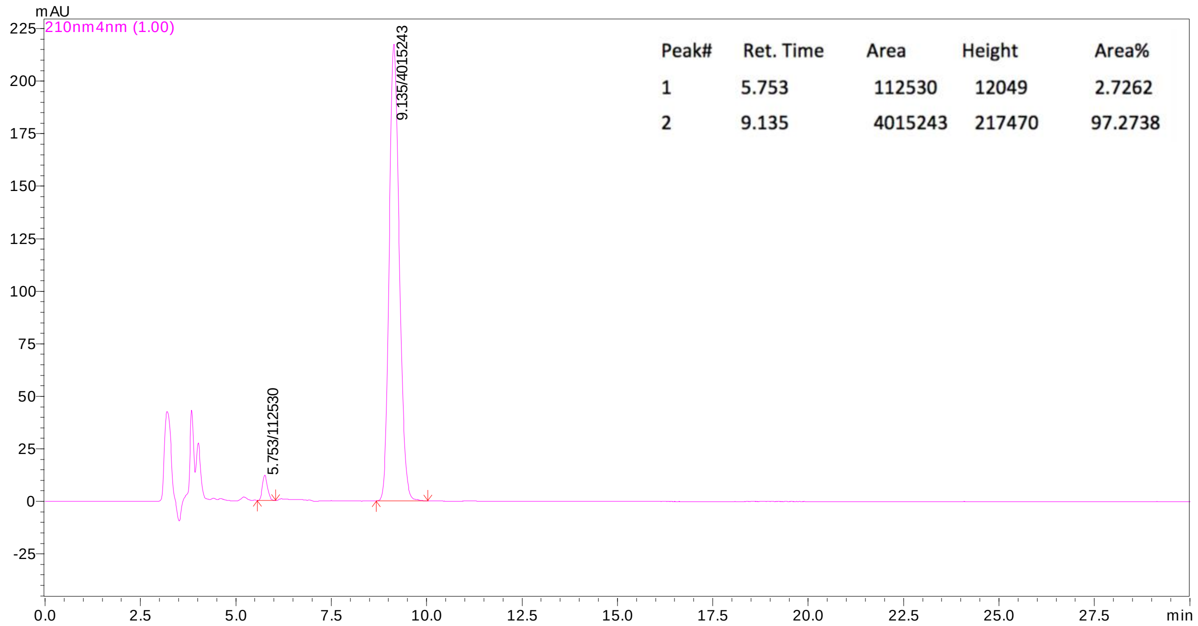

Peak#	Ret. Time	Area	Height	Area%
1	5.753	112530	12049	2.7262
2	9.135	4015243	217470	97.2738

Supplementary Figure 95. HPLC traces for racemic and chiral product **2e**

Datafile Name:YH00095-129-0.lcd

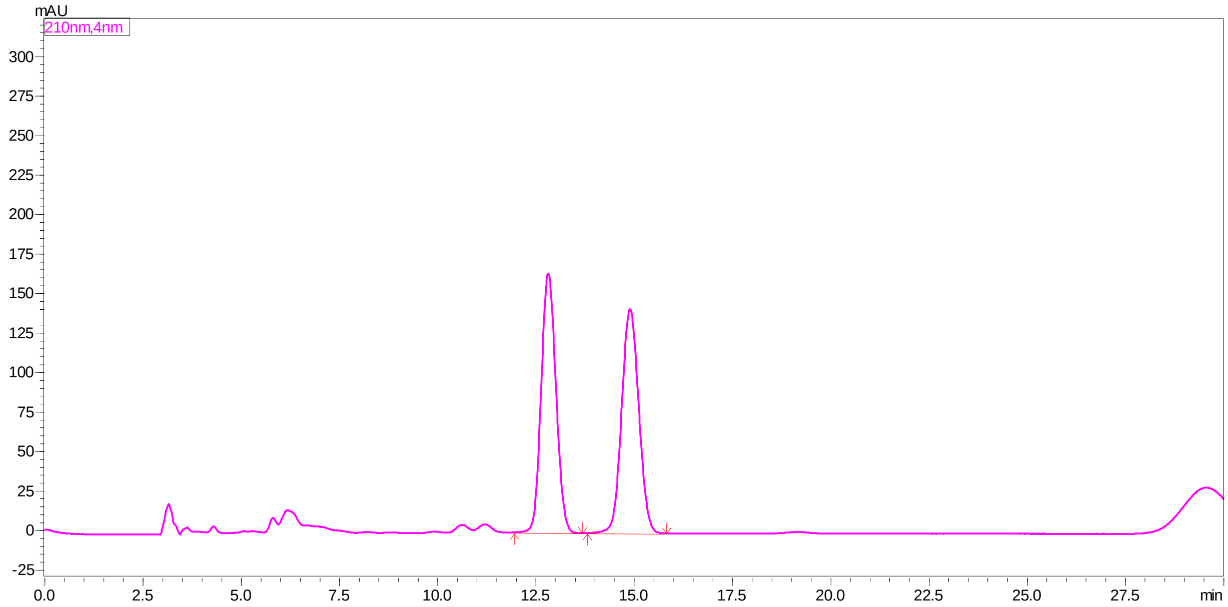

Datafile Name:YH00095-129-1.lcd

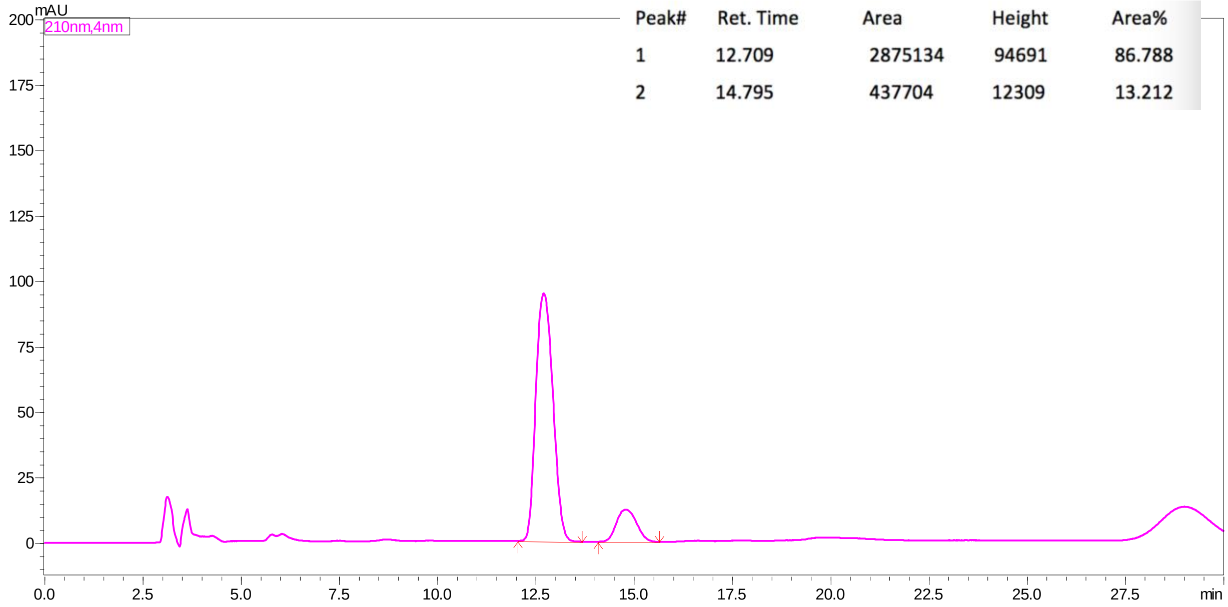

Peak#	Ret. Time	Area	Height	Area%
1	12.709	2875134	94691	86.788
2	14.795	437704	12309	13.212

Supplementary Figure 96. HPLC traces for racemic and chiral product **2f**

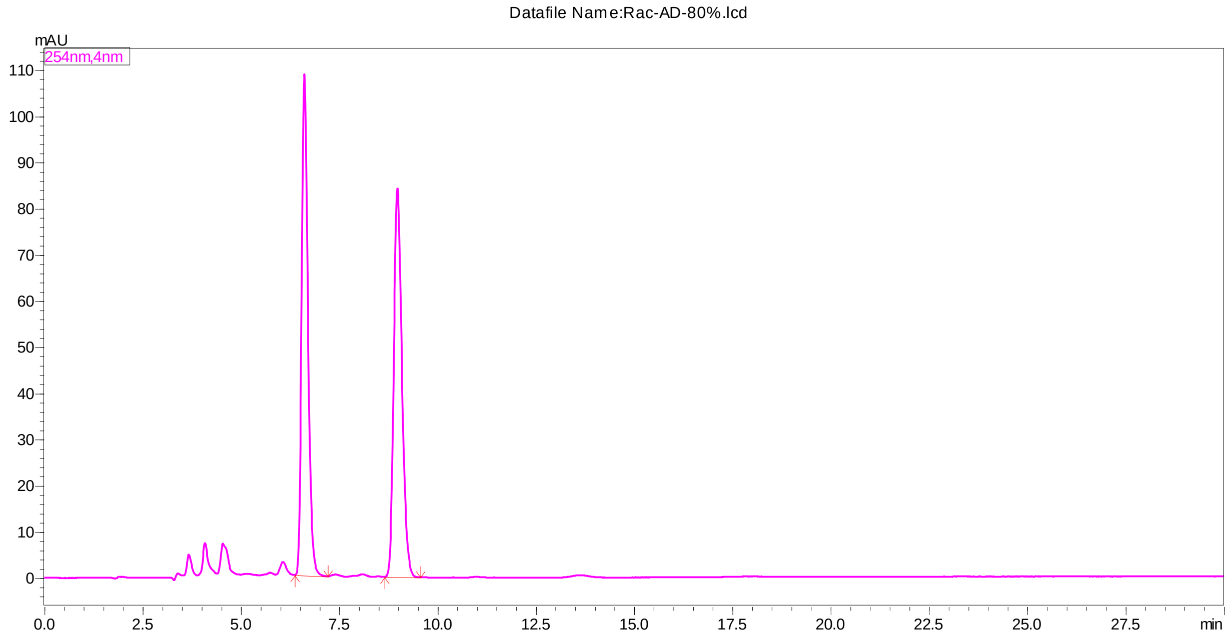

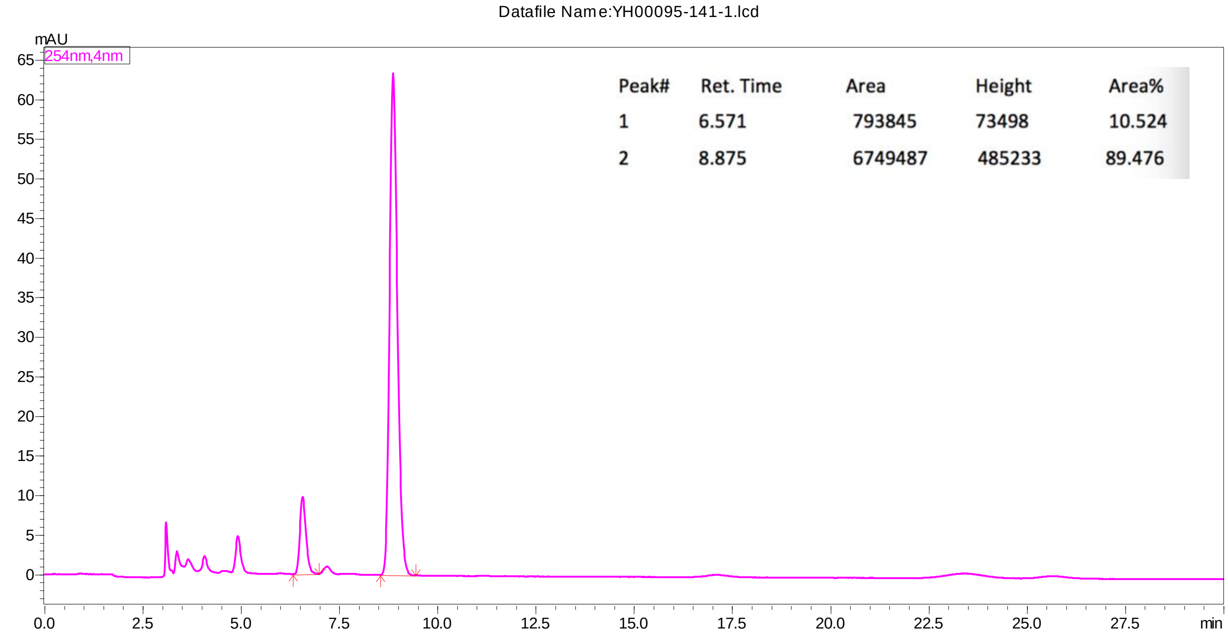

Peak#	Ret. Time	Area	Height	Area%
1	6.571	793845	73498	10.524
2	8.875	6749487	485233	89.476

Supplementary Figure 97. HPLC traces for racemic and chiral product **2g**

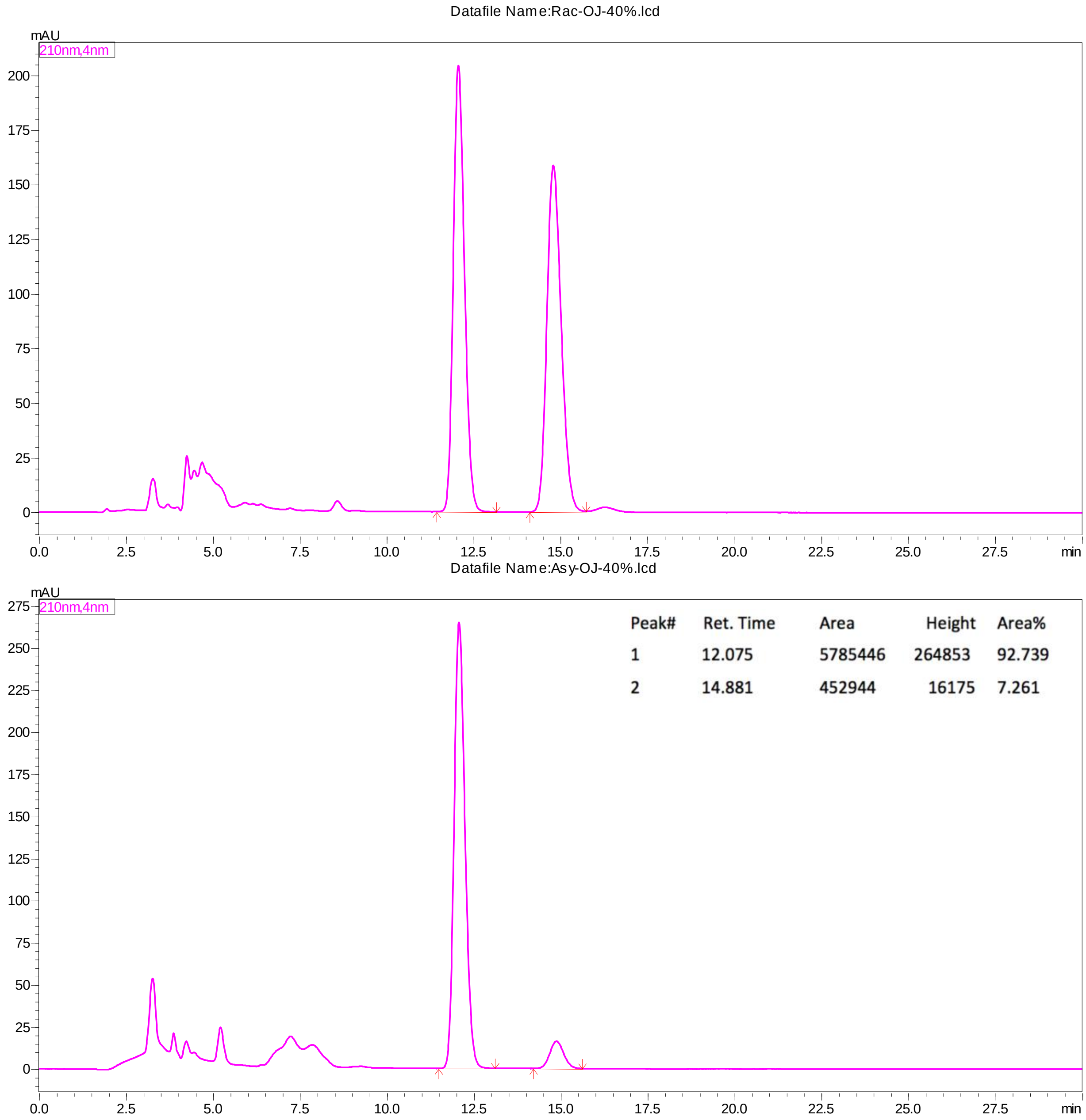

Supplementary Figure 98. HPLC traces for racemic and chiral product **2h**

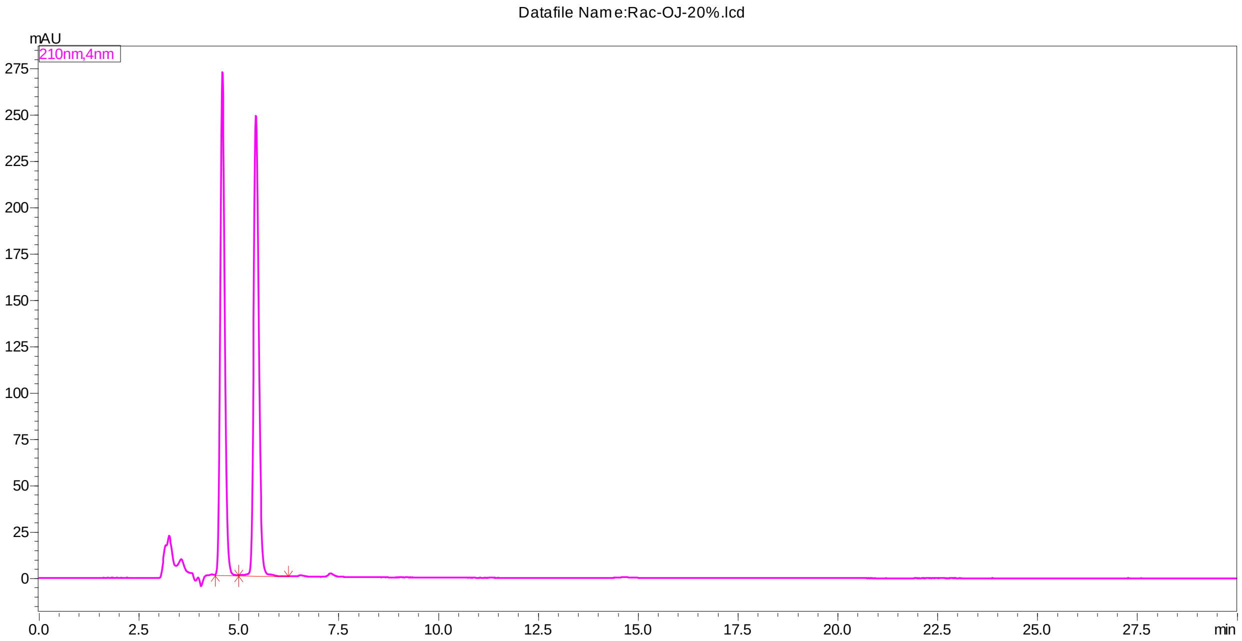

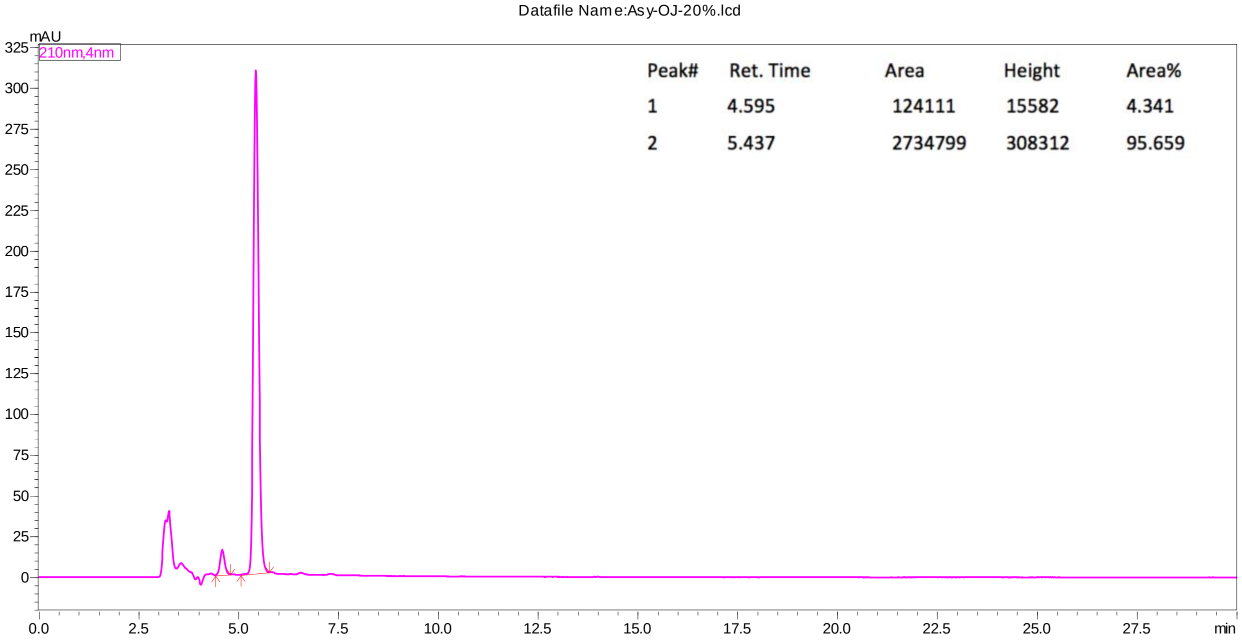

Peak#	Ret. Time	Area	Height	Area%
1	4.595	124111	15582	4.341
2	5.437	2734799	308312	95.659

Supplementary Figure 99. HPLC traces for racemic and chiral product **2i**

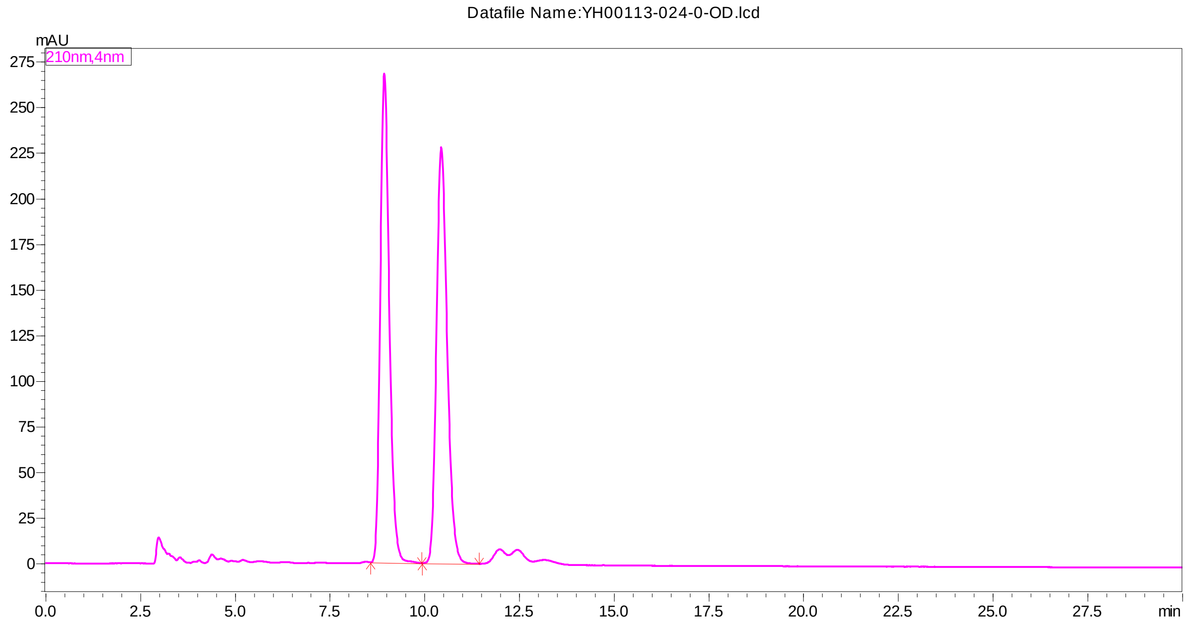

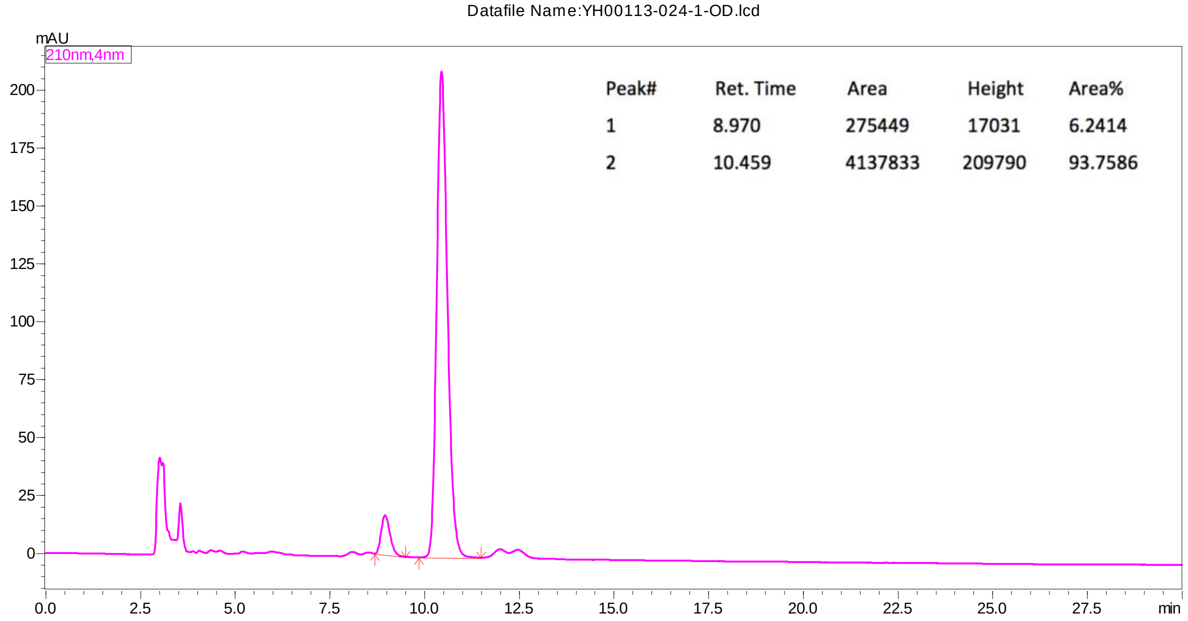

Peak#	Ret. Time	Area	Height	Area%
1	8.970	275449	17031	6.2414
2	10.459	4137833	209790	93.7586

Supplementary Figure 100. HPLC traces for racemic and chiral product **2j**

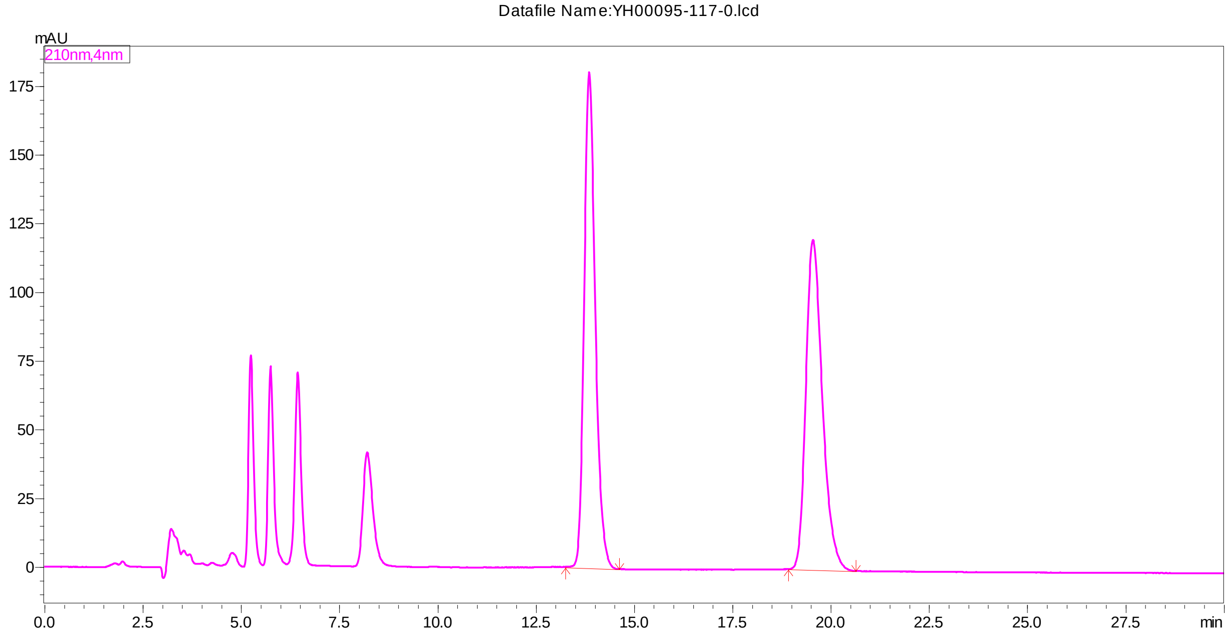

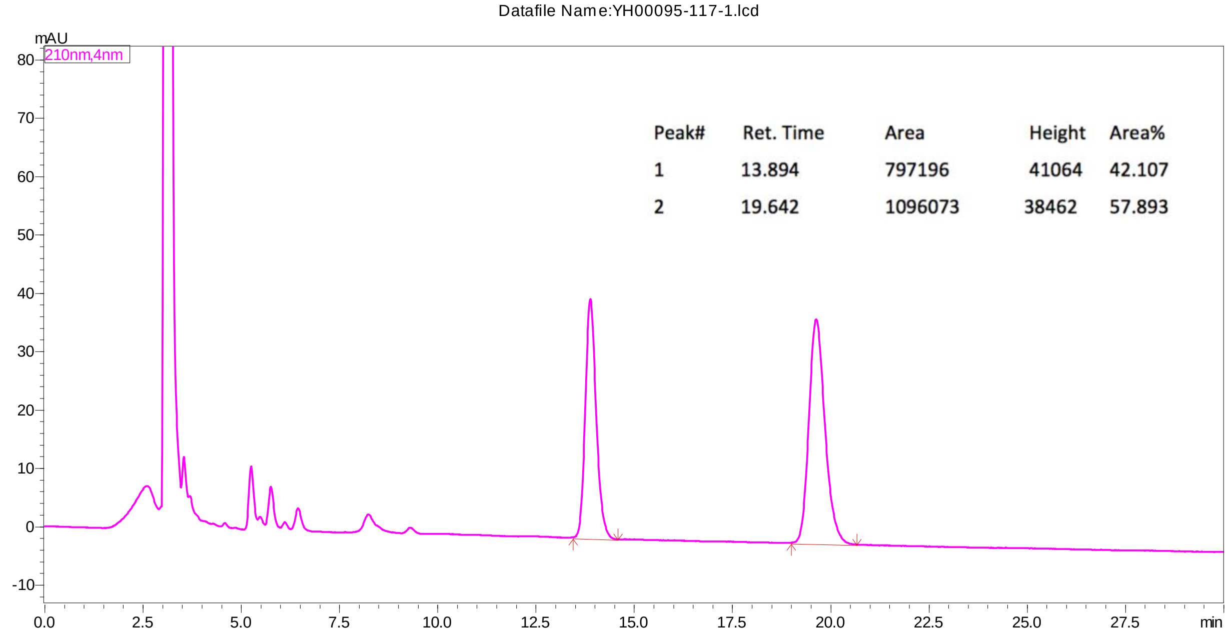

Peak#	Ret. Time	Area	Height	Area%
1	13.894	797196	41064	42.107
2	19.642	1096073	38462	57.893

Supplementary Figure 100. HPLC traces for racemic and chiral product **2k**

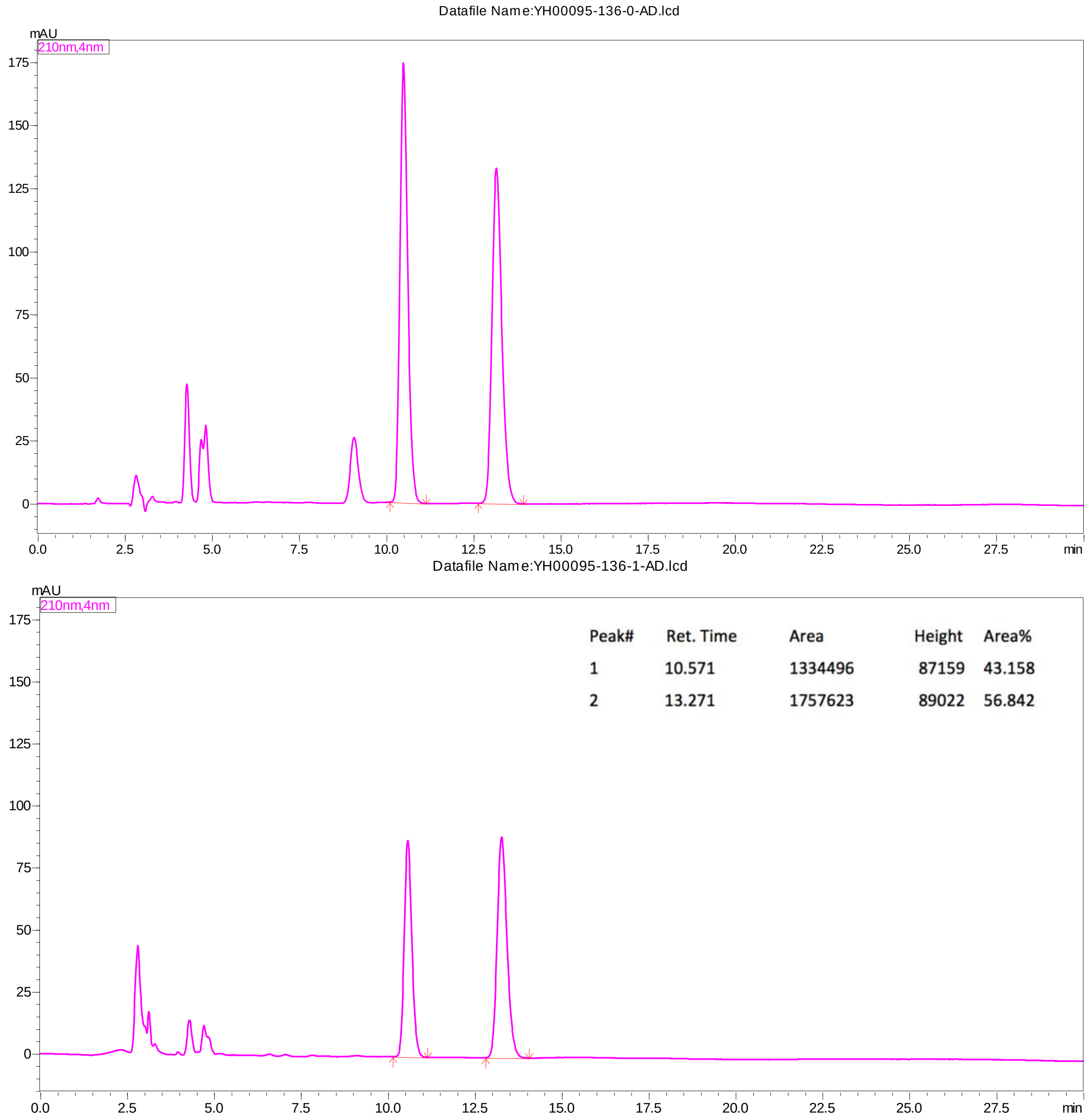

Peak#	Ret. Time	Area	Height	Area%
1	10.571	1334496	87159	43.158
2	13.271	1757623	89022	56.842

Supplementary Figure 101. HPLC traces for racemic and chiral product **2l**

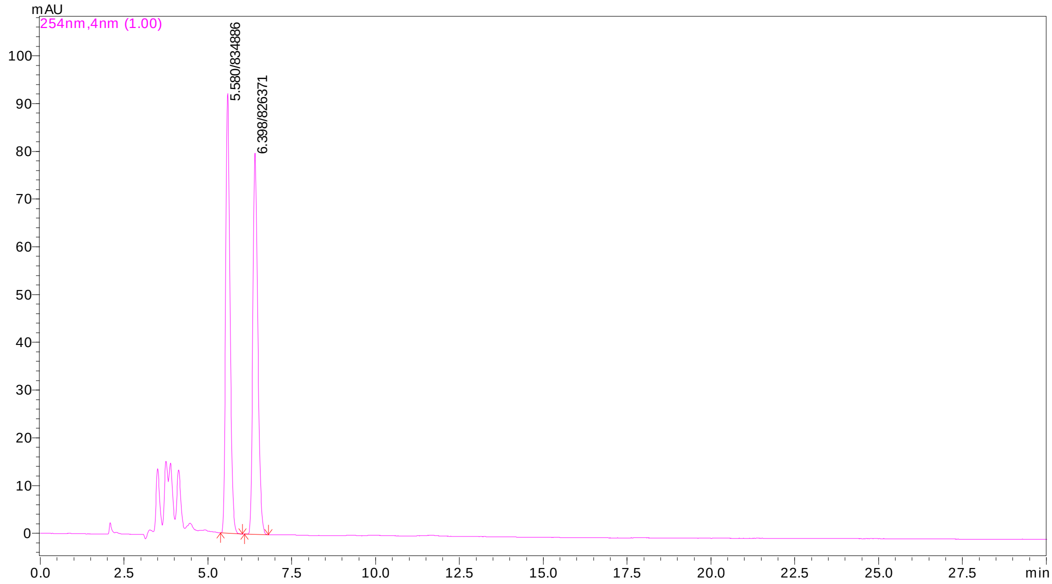

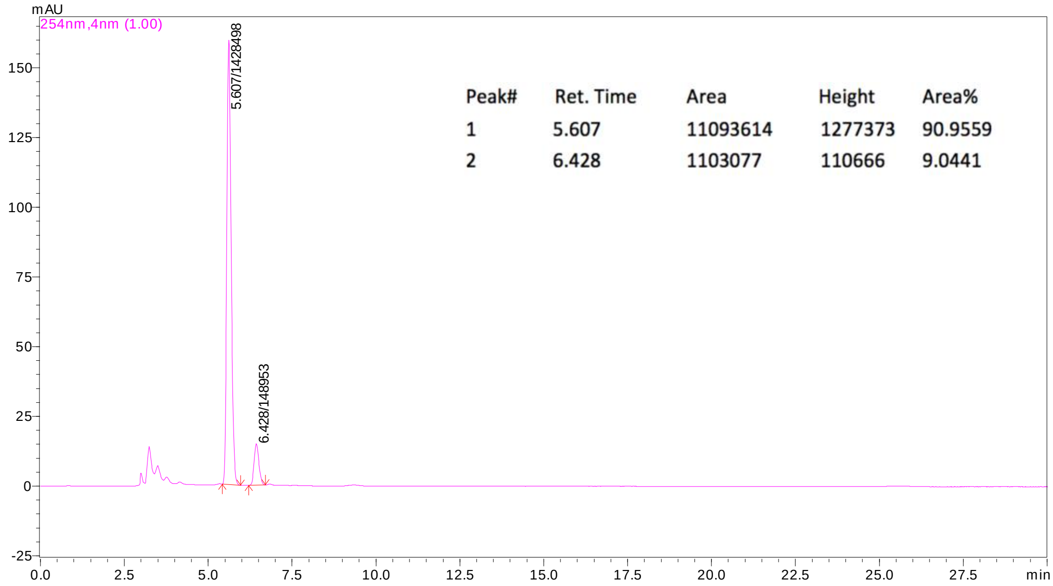

Peak#	Ret. Time	Area	Height	Area%
1	5.607	11093614	1277373	90.9559
2	6.428	1103077	110666	9.0441

Supplementary Figure 102. HPLC traces for racemic and chiral product **2m**

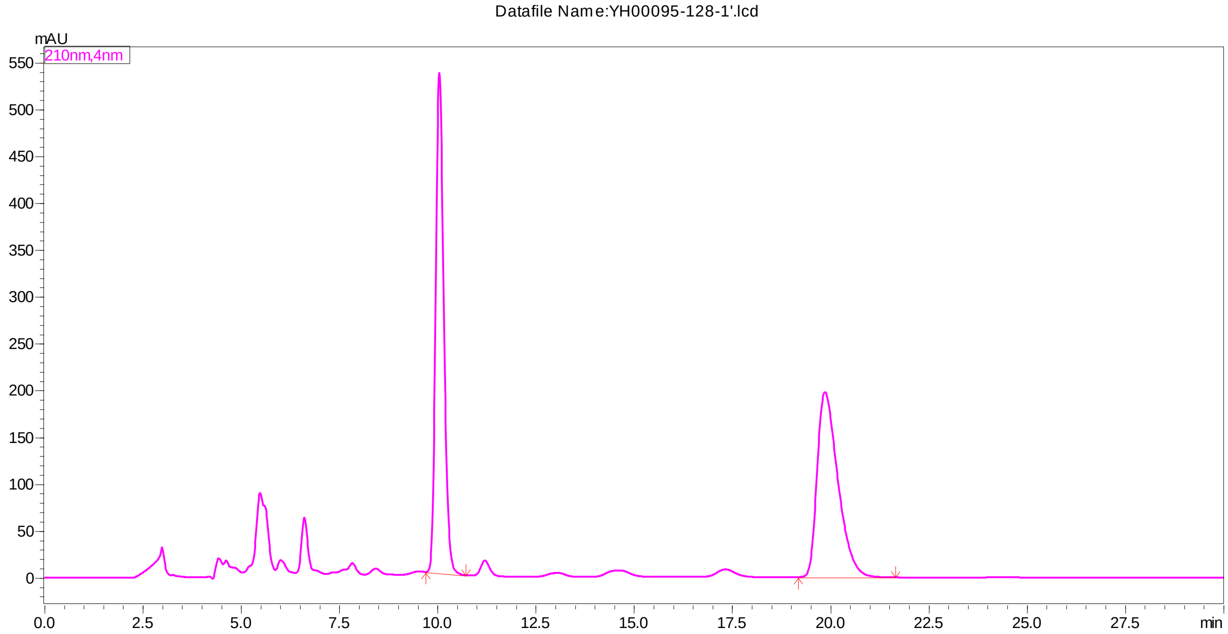

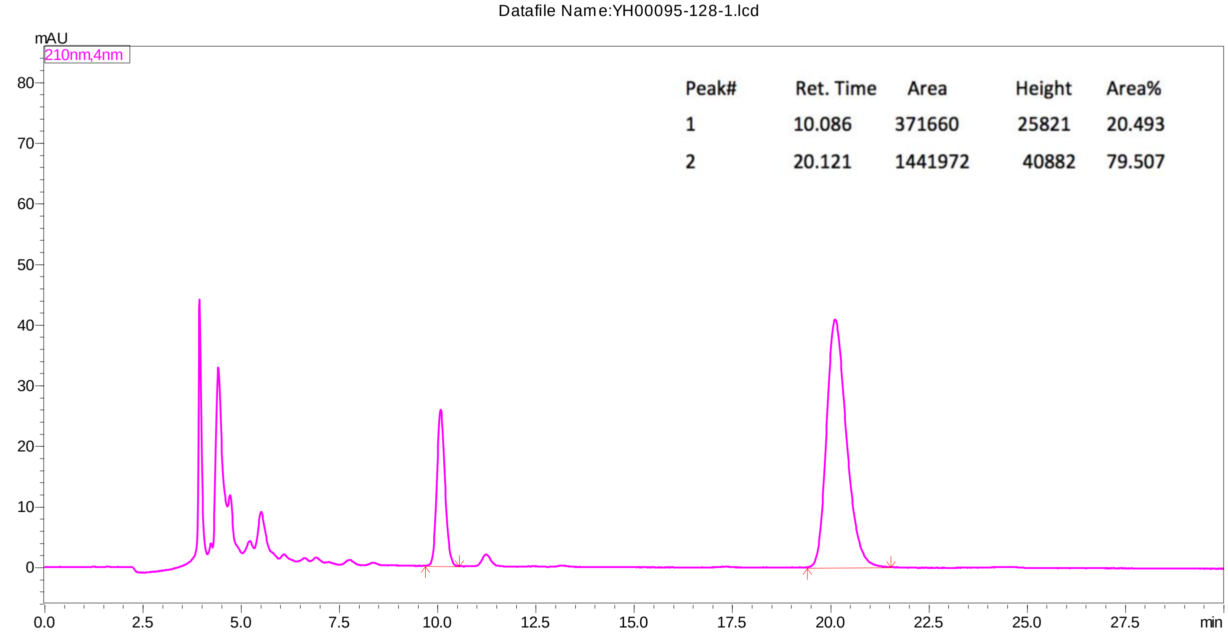

Peak#	Ret. Time	Area	Height	Area%
1	10.086	371660	25821	20.493
2	20.121	1441972	40882	79.507

Supplementary Figure 103. HPLC traces for racemic and chiral product **2n**

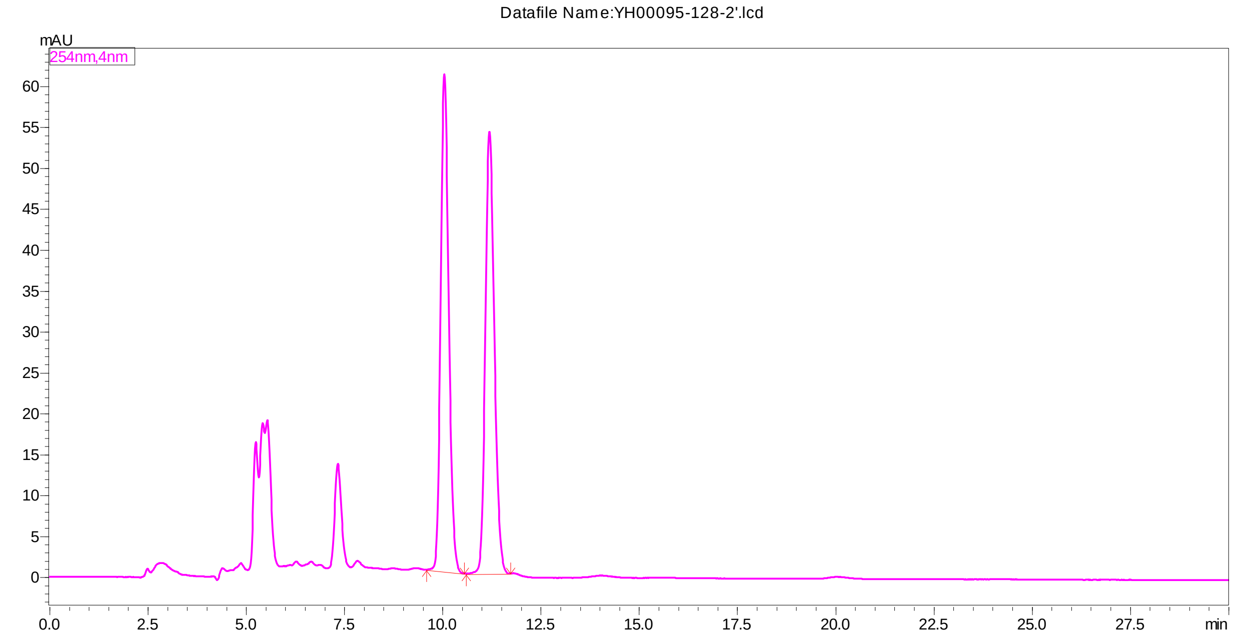

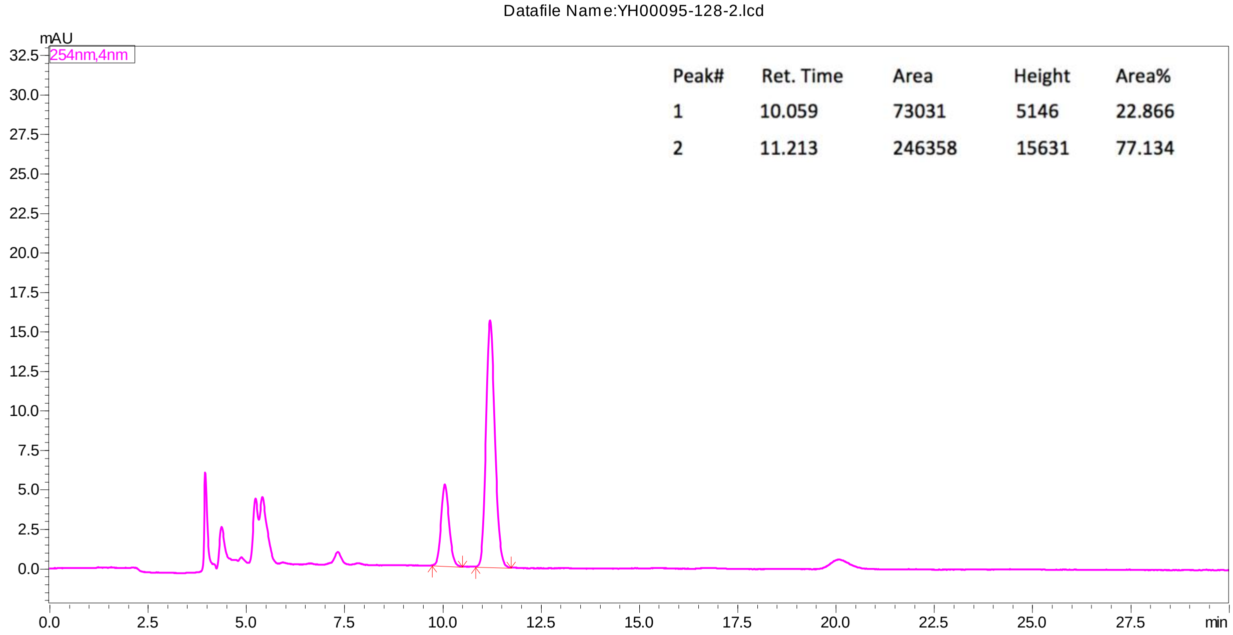

Peak#	Ret. Time	Area	Height	Area%
1	10.059	73031	5146	22.866
2	11.213	246358	15631	77.134

Supplementary Figure 104. HPLC traces for racemic and chiral product **2n'**

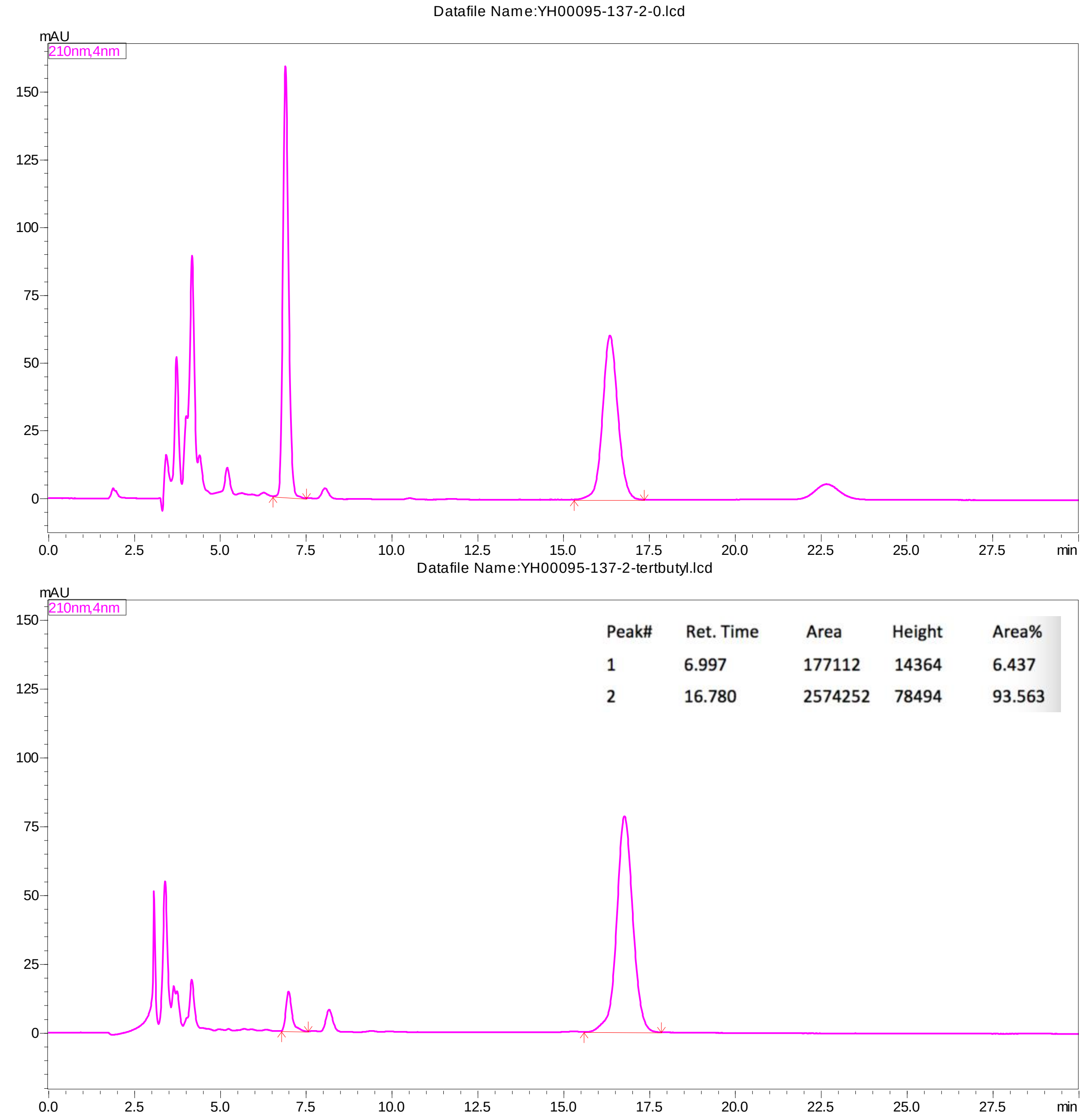

Peak#	Ret. Time	Area	Height	Area%
1	6.997	177112	14364	6.437
2	16.780	2574252	78494	93.563

Supplementary Figure 105. HPLC traces for racemic and chiral product **2o**

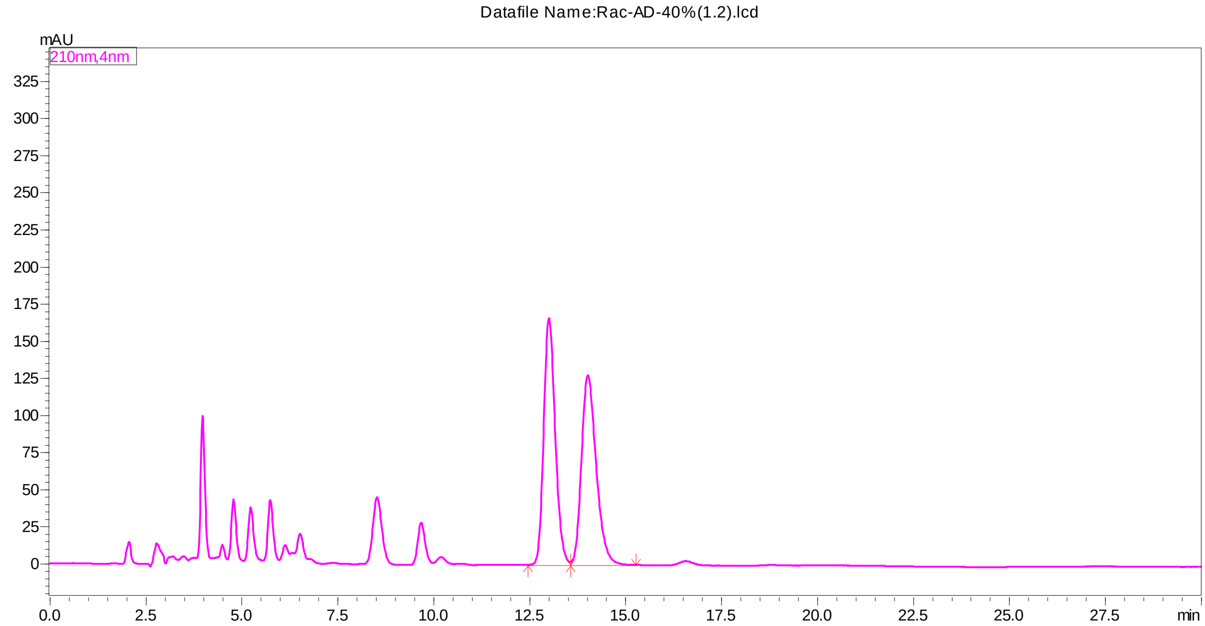

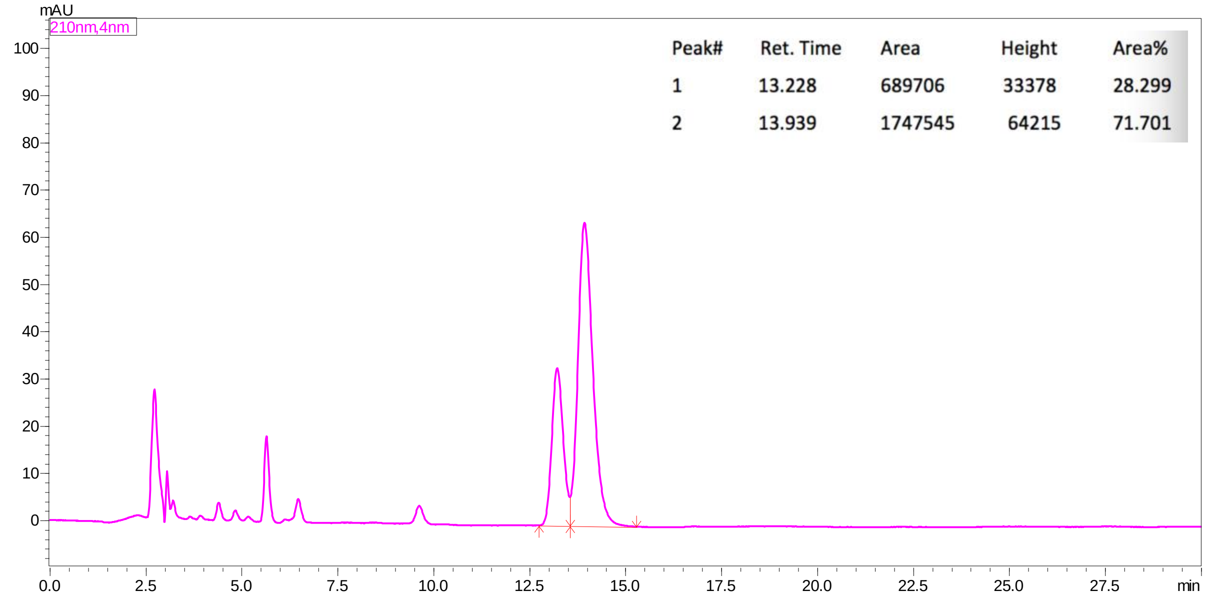

Peak#	Ret. Time	Area	Height	Area%
1	13.228	689706	33378	28.299
2	13.939	1747545	64215	71.701

Supplementary Figure 106. HPLC traces for racemic and chiral product **2o'**

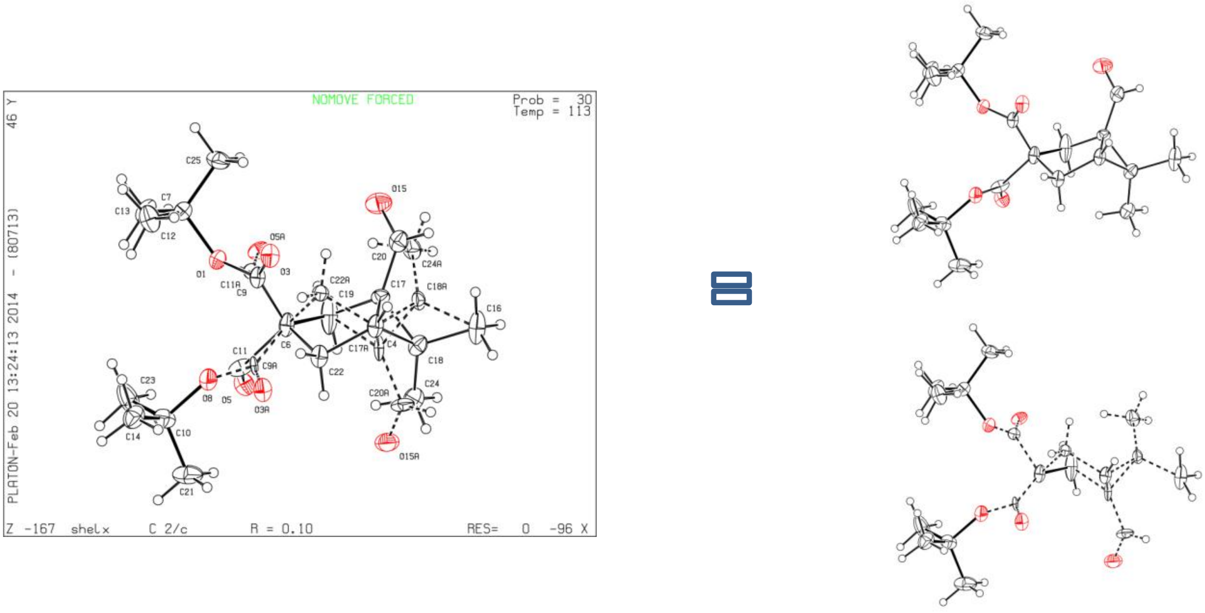

Supplementary Figure 107. ORTEP presentation of **2b**

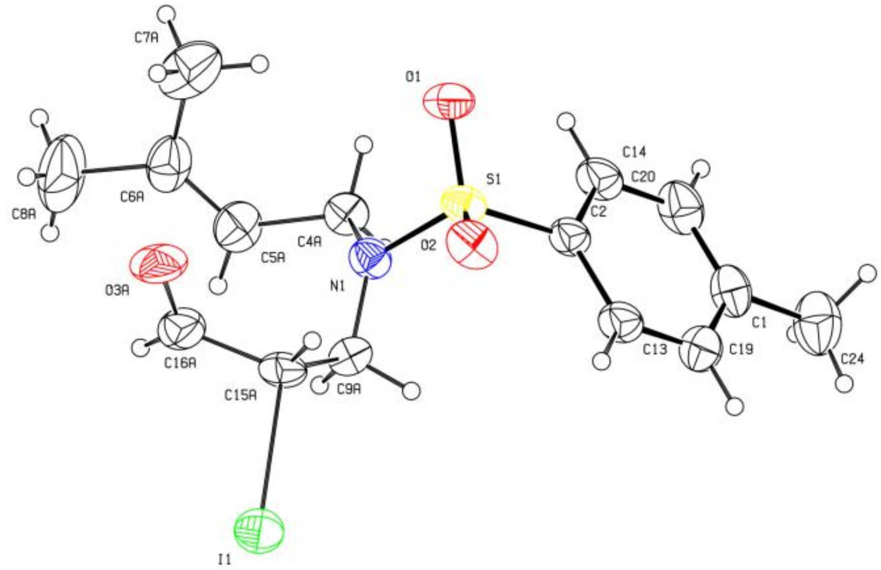

Supplementary Figure 108. ORTEP presentation of ***rac*-4c**

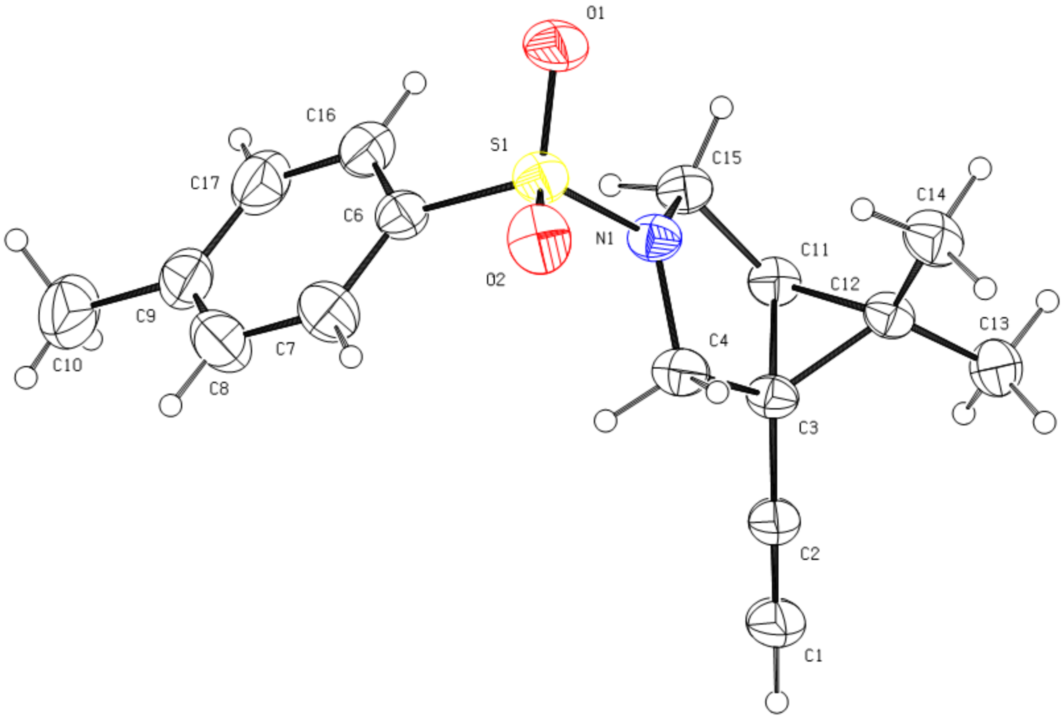

Supplementary Figure 109. ORTEP presentation of **8c**

Supplementary methods

Analytical data of the substrates:

(1) **dibenzyl 2-(3-methylbut-2-en-1-yl)-2-(3-oxopropyl)malonate 1a** (SM01)

The title compound was prepared according to method A. **^{1}H NMR** (400 MHz, $CDCl_3$) δ 9.62 (t, *J* = 1.2 Hz, 1H), 7.35 – 7.22 (m, 10H), 5.16 – 5.05 (m, 4H), 4.88 (ddd, *J* = 7.4, 4.4, 1.4 Hz, 1H), 2.63 (d, *J* = 7.4 Hz, 2H), 2.40 – 2.31 (m, 2H), 2.24 – 2.15 (m, 2H), 1.63 (d, *J* = 1.0 Hz, 3H), 1.54 (s, 3H). **^{13}C NMR** (101 MHz, $CDCl_3$) δ 200.72, 170.81, 136.23, 135.29, 128.54, 128.36, 128.26, 116.99, 67.13, 56.94, 39.11, 32.03, 25.94, 24.87, 17.94. **HRMS** (ESI-TOF) [M+Na] calculated for $[C_{25}H_{28}NaO_5]^+$ 431.1834, observed 431.1823.

(2) **di-*tert*-butyl 2-(3-methylbut-2-en-1-yl)-2-(3-oxopropyl)malonate 1b** (SM02)

The title comound was prepared according to method A. **^{1}H NMR** (400 MHz, $CDCl_3$) δ 9.74 (t, *J* = 1.4 Hz, 1H), 4.96 (ddd, *J* = 7.3, 6.0, 1.3 Hz, 1H), 2.51 (d, *J* = 7.3 Hz, 2H), 2.45 – 2.35 (m, 2H), 2.10 (dd, *J* = 9.2, 6.9 Hz, 2H), 1.68 (d, *J* = 0.7 Hz, 3H), 1.62 (s, 3H), 1.44 (s, 18H). **^{13}C NMR** (101 MHz, $CDCl_3$) δ 201.25, 170.40, 135.20, 117.76, 81.47, 57.45, 39.21, 31.61, 27.86, 25.99, 24.65, 18.08. **HRMS** (ESI-TOF) [M+H] calculated for $[C_{19}H_{33}O_5]^+$ 341.2328, observed 341.2336.

(3) **4-methyl-*N*-(3-methylbut-2-en-1-yl)-*N*-(3-oxopropyl)benzenesulfonamide 1c** (SM03)

The title compound was prepared according to method B. **^{1}H NMR** (400 MHz, $CDCl_3$) δ 9.75 (s, 1H), 7.68 (d, J = 8.3 Hz, 2H), 7.30 (d, J = 8.1 Hz, 2H), 4.98 (t, J = 7.1 Hz, 1H), 3.77 (d, J = 7.0 Hz, 2H), 3.37 (t, J = 7.1 Hz, 2H), 2.79 (t, J = 7.1 Hz, 2H), 2.43 (s, 3H), 1.66 (s, 3H), 1.61 (s, 3H). **^{13}C NMR** (101 MHz, $CDCl_3$) δ 200.55, 143.41, 137.64, 136.35, 129.71, 127.25, 118.68, 46.50, 44.01, 40.96, 25.77, 21.52, 17.80. **HRMS** (ESI-TOF) [M+H] calculated for $[C_{15}H_{22}NO_3S]^+$ 296.1320, observed 296.1304.

(4) ***N*-(3-ethylpent-2-en-1-yl)-4-methyl-N-(3-oxopropyl)benzenesulfonamide 1d** (SM04)

The title compound was prepared according to method B. **^{1}H NMR** (500 MHz, $CDCl_3$) δ 9.71 (d, J = 2.7 Hz, 1H), 7.84 – 7.56 (m, 2H), 7.20 – 7.28 (m, 2H), 4.85 (s, 1H), 3.79 (s, 2H), 3.60 – 3.28 (m, 2H), 2.76 (d, J = 6.0 Hz, 2H), 2.29 (s, 3H), 1.96 (dd, J = 15.7, 7.5 Hz, 4H), 1.01 – 0.80 (m, 6H). **^{13}C NMR** (101 MHz, $CDCl_3$) δ 200.51, 148.42, 143.41, 136.46, 129.71, 127.26, 116.58, 46.09, 44.07, 40.96, 28.92, 23.35, 21.50, 13.09, 12.25. **HRMS** (ESI-TOF) [M+H] calculated for $[C_{17}H_{26}NO_3S]^+$ 324.1633, observed 324.1639.

(5) **dibenzyl 2-(3-hexylnon-2-en-1-yl)-2-(3-oxopropyl)malonate 1e** (SM05)

The title compound was prepared according to method A. **^{1}H NMR** (300 MHz, $CDCl_3$) δ 9.61 (s, 1H), 7.40 – 7.17 (m, 10H), 5.19 – 5.02 (m, 4H), 4.85 (t, J = 7.2 Hz, 1H), 2.65 (d, J = 7.2 Hz, 2H), 2.33 (d, J = 7.5 Hz, 2H), 2.20 (dd, J = 8.4, 5.8 Hz, 2H), 1.89 (t, J = 7.1 Hz, 4H), 1.23 (s, 16H), 0.88 (td, J = 6.7, 2.1 Hz, 6H). **^{13}C NMR** (75 MHz, $CDCl_3$) δ 200.70, 170.82, 144.69, 135.30, 128.54, 128.36, 128.25, 116.46, 67.13, 56.89, 39.11, 37.02, 31.77, 31.47, 30.21, 29.49, 29.10, 28.31, 28.14, 24.86, 22.69, 22.66, 14.14. **HRMS** (ESI-TOF) [M+H] calculated for $[C_{35}H_{49}O_5]^+$ 549.3580, observed 549.3587.

(6) **dibenzyl 2-(2-cyclopentylideneethyl)-2-(3-oxopropyl)malonate 1f** (SM06)

The title compound was prepared according to method B. **^{1}H NMR** (400 MHz, $CDCl_3$) δ 9.76 (s, 1H), 7.69 – 7.66 (m, 2H), 7.30 (d, J = 7.9 Hz, 2H), 5.08 (dtd, J = 6.8, 4.6, 2.2 Hz, 1H), 3.75 (d, J = 7.0 Hz, 2H), 3.38 (t, J = 7.1 Hz, 2H), 2.80 (td, J = 7.1, 1.1 Hz, 2H), 2.42 (d, J = 3.0 Hz, 3H), 2.17 (dd, J = 5.1, 3.3 Hz, 4H), 1.71 – 1.53 (m, 4H). **^{13}C NMR** (101 MHz, $CDCl_3$) δ 200.58, 149.25, 143.37, 136.40, 129.69, 127.25, 114.11, 48.03, 44.03, 41.08, 33.77, 28.80, 26.22, 25.94, 21.51. **HRMS** (ESI-TOF) [M+Na] calculated for $[C_{17}H_{23}NNaO_3S]^+$ 344.1296, observed 344.1310.

(7) ***N*-(2-cyclohexylideneethyl)-4-methyl-*N*-(3-oxopropyl)benzenesulfonamide 1g** (SM07)

The title compound was prepared according to method B. **^{1}H NMR** (400 MHz, $CDCl_3$) δ 9.76 (t, J = 1.1 Hz, 1H), 7.71 – 7.67 (m, 2H), 7.31 (d, J = 8.0 Hz, 2H), 4.94 (t, J = 7.3 Hz, 1H), 3.78 (d, J = 7.3 Hz, 2H), 3.37 (t, J = 7.1 Hz, 2H), 2.84 – 2.76 (m, 2H), 2.43 (s, 3H), 2.13 – 2.06 (m, 2H), 2.02 (d, J = 4.9 Hz, 2H), 1.56 – 1.44 (m, 6H). **^{13}C NMR** (101 MHz, $CDCl_3$) δ 200.52, 145.71, 143.39, 136.36, 129.72, 127.26, 115.30, 45.58, 44.11, 40.81, 37.08, 28.63, 28.30, 27.62, 26.54, 21.52. **HRMS** (ESI-TOF) [M+Na] calculated for $[C_{18}H_{25}NNaO_3S]^+$ 358.1453, observed 358.1449.

(8) ***N*-(2-cycloheptylideneethyl)-4-methyl-*N*-(3-oxopropyl)benzenesulfonamide 1h** (SM08)

The title compound was prepared according to method B. **^{1}H NMR** (400 MHz, $CDCl_3$) δ 9.76 (d, J = 1.0 Hz, 1H), 7.68 (d, J = 8.3 Hz, 2H), 7.30 (d, J = 8.0 Hz, 2H), 4.98 (t, J = 6.8 Hz, 1H), 3.78 (d, J = 6.9 Hz, 2H), 3.38 (t, J = 7.1 Hz, 2H), 2.87 – 2.76 (m, 2H), 2.43 (s, 3H), 2.24 – 2.10 (m, 4H), 1.48 (s, 8H). **^{13}C NMR** (101 MHz, $CDCl_3$) δ 200.51, 147.00, 143.39, 136.35, 129.70, 127.28, 118.87, 46.16, 44.08, 41.10, 37.68, 29.97, 29.62, 29.03, 28.86, 26.86, 21.52. **HRMS** (ESI-TOF) [M+Na] calculated for $[C_{19}H_{27}NNaO_3S]^+$ 372.1609, observed 372.1606.

(9) **3-(2,2-dimethyl-5-(3-methylbut-2-en-1-yl)-1,3-dioxan-5-yl)propanal 1i** (SM09)

The title compound was prepared according to method C. **^{1}H NMR** (400 MHz, $CDCl_3$) δ 9.78 (s, 1H), 5.13 – 4.98 (m, 1H), 3.57 (dd, J = 29.1, 11.7 Hz, 4H), 2.48 – 2.39 (m, 2H), 2.03 (d, J = 7.7 Hz, 2H), 1.74 – 1.65 (m, 5H), 1.63 (s, 3H), 1.40 (d, J = 6.0 Hz,

6H). **^{13}C NMR** (101 MHz, $CDCl_3$) δ 202.31, 135.27, 117.94, 98.21, 67.61, 38.32, 35.41, 30.73, 26.17, 24.65, 24.35, 23.43, 18.01. **HRMS** (ESI-TOF) [M+H] calculated for $[C_{14}H_{25}O_3]^+$ 241.1804, observed 241.1821.

(10) **3-(3-(3-methylbut-2-en-1-yl)-1-tosylazetidin-3-yl)propanal 1j** (SM10)

The title compound was prepared according to method D. **^{1}H NMR** (400 MHz, $CDCl_3$) δ 9.71 (t, J = 1.3 Hz, 1H), 7.72 (d, J = 8.2 Hz, 2H), 7.38 (d, J = 7.9 Hz, 2H), 4.86 – 4.74 (m, 1H), 3.46 (d, J = 7.9 Hz, 2H), 3.40 (d, J = 7.9 Hz, 2H), 2.46 (s, 3H), 2.37 – 2.26 (m, 2H), 2.02 (d, J = 7.2 Hz, 2H), 1.69 (dd, J = 8.7, 7.1 Hz, 2H), 1.61 (d, J = 0.9 Hz, 3H), 1.50 (s, 3H). **^{13}C NMR** (101 MHz, $CDCl_3$) δ 201.00, 144.08, 135.54, 131.49, 129.75, 128.37, 117.70, 58.77, 38.77, 36.24, 34.71, 28.21, 25.92, 21.60, 18.00. **HRMS** (ESI-TOF) [M+Na] calculated for $[C_{18}H_{25}NNaO_3S]^+$ 358.1453, observed 358.1460.

(11) ***N*-allyl-4-methyl-*N*-(3-oxopropyl)benzenesulfonamide 1k** (SM11)

The title compound was prepared according to method B. **^{1}H NMR** (400 MHz, $CDCl_3$) δ 9.73 (d, J = 0.9 Hz, 1H), 7.71 – 7.64 (m, 2H), 7.30 (d, J = 8.0 Hz, 2H), 5.62 (ddt, J = 16.6, 10.1, 6.4 Hz, 1H), 5.16 (ddd, J = 10.8, 8.6, 1.3 Hz, 2H), 3.77 (d, J = 6.4 Hz, 2H), 3.40 (t, J = 7.1 Hz, 2H), 2.84 – 2.75 (m, 2H), 2.42 (s, 3H). **^{13}C NMR** (101 MHz, $CDCl_3$) δ 200.30, 143.61, 136.20, 132.88, 129.83, 127.20, 119.42, 51.71, 43.75, 41.01, 21.52. **HRMS** (ESI-TOF) [M+H] calculated for $[C_{13}H_{18}NO_3S]^+$ 268.1007, observed 268.0999.

(12) **4-methyl-*N*-(2-methylallyl)-*N*-(3-oxopropyl)benzenesulfonamide 1l** (SM12)

The title compound was prepared according to method B. **^{1}H NMR** (400 MHz, $CDCl_3$) δ 9.71 (s, 1H), 7.68 (d, J = 8.2 Hz, 2H), 7.31 (d, J = 8.3 Hz, 2H), 4.89 (d, J = 15.9 Hz, 2H), 3.65 (s, 2H), 3.36 (t, J = 7.3 Hz, 2H), 2.77 (t, J = 7.3 Hz, 2H), 2.43 (s, 3H), 1.72 (s, 3H). **^{13}C NMR** (101 MHz, $CDCl_3$) δ 200.34, 143.69, 140.65, 136.12, 129.90, 127.33, 115.13, 55.79, 43.65, 41.73, 21.63, 19.88. **HRMS** (ESI-TOF) [M+Na] calculated for $[C_{14}H_{19}NNaO_3S]^+$ 304.0983, observed 304.0988.

(13) ***N*-(2,3-dimethylbut-2-en-1-yl)-4-methyl-*N*-(3-oxopropyl)benzenesulfonamide 1m** (SM13)

The title compound was prepared according to method B. **^{1}H NMR** (400 MHz, $CDCl_3$) δ 9.71 (s, 1H), 7.69 (d, J = 8.3 Hz, 2H), 7.32 (d, J = 8.0 Hz, 2H), 3.73 (s, 2H), 3.33 – 3.24 (m, 2H), 2.79 – 2.70 (m, 2H), 2.44 (s, 3H), 1.66 (d, J = 4.8 Hz, 6H), 1.63 (d, J = 0.9 Hz, 3H). **^{13}C NMR** (101 MHz, $CDCl_3$) δ 200.47, 143.39, 135.99, 131.22, 129.73, 127.26, 122.57, 51.46, 44.00, 41.36, 21.51, 21.16, 20.11, 16.34. **HRMS** (ESI-TOF) [M+H] calculated for $[C_{16}H_{24}NO_3S]^+$ 310.1477, observed 310.1469.

(14) **(*Z,E*)-*N*-(but-2-en-1-yl)-4-methyl-*N*-(3-oxopropyl)benzenesulfonamide 1n** (SM14)

The title compound was prepared according to method B. **^{1}H NMR** (400 MHz, $CDCl_3$) δ 9.75 (dd), 7.74 – 7.64 (m), 7.36 – 7.27 (m), 5.71 – 5.51 (m), 5.34 – 5.16 (m), 3.84 (d), 3.71 (d), 3.38 (td), 2.80 (ddd), 2.43 (s), 1.68 – 1.54 (m). **^{13}C NMR** (101 MHz, $CDCl_3$) δ 200.50, 200.44, 143.51, 143.47, 136.34, 131.02, 129.78, 129.75, 129.21, 127.22, 125.41, 124.55, 50.96, 45.22, 43.95, 43.83, 41.07, 40.67, 21.51, 17.65, 12.86. **HRMS** (ESI-TOF) [M+H] calculated for $[C_{14}H_{19}NO_3S]^+$ 281.1086, observed 281.1094.

(15) **(*E*)-4-methyl-*N*-(3-oxopropyl)-*N*-(3-phenylbut-2-en-1-yl)benzenesulfonamide 1o** (SM15)

The title compound was prepared according to method B. **^{1}H NMR** (500 MHz, $CDCl_3$) δ 9.76 (s, 1H), 7.72 (d, J = 8.3 Hz, 2H), 7.34 – 7.27 (m, 4H), 7.26 – 7.15 (m, 3H), 5.51 (td, J = 6.8, 1.3 Hz, 1H), 4.02 (d, J = 6.8 Hz, 2H), 3.46 (t, J = 7.0 Hz, 2H), 2.89 – 2.82 (m, 2H), 2.43 (s, 3H), 2.03 (s, 3H). **^{13}C NMR** (126 MHz, $CDCl_3$) δ 200.15, 143.55, 142.46, 139.46, 136.55, 129.80, 128.28, 127.47, 127.30, 125.66, 121.71, 47.09, 44.05, 41.39, 21.46, 15.95. **HRMS** (ESI-TOF) [M+H] calculated for $[C_{20}H_{24}NO_3S]^+$ 358.1477, observed 358.1472.

Analytical data of the products:

(16) **(1R,5S)-dibenzyl 1-formyl-6,6-dimethylbicyclo[3.1.0]hexane-3,3-dicarboxylate 2a** (CP001)

Colorless liquid, yield: 73%; er = 93:7, determined by chiral HPLC. **^{1}H NMR** (400 MHz, $CDCl_3$) δ 9.28 (s, 1H), 7.32-7.20 (m, 10H), 5.14 – 5.04 (m, 4H), 3.26 (dd, J = 15.0, 1.4 Hz, 1H), 2.70 (ddd, J = 14.4, 7.2, 1.4 Hz, 1H), 2.33 (dd, J = 7.2, 3.2 Hz, 1H), 2.16 (d, J = 15.0 Hz, 1H), 2.04 (dd, J = 14.5, 3.2 Hz, 1H), 1.38 (s, 3H), 1.18 (s, 3H). **^{13}C NMR** (101 MHz, $CDCl_3$) δ 200.09, 171.63, 169.96, 135.28, 128.53, 128.31, 128.19, 127.95, 68.31, 67.56, 67.17, 52.27, 43.00, 38.25, 33.24, 31.46, 22.54, 17.42. **HRMS** (ESI-TOF) [M+Na] calculated for $[C_{25}H_{26}NaO_5]^+$ 429.1678, observed 429.1685. **HPLC** (Chiralpak AD-H column, 90:10 hexane/ethanol, flow rate: 1.0 mL/min): t_{major} = 14.808min; t_{minor} = 12.145 min.

(17) **(1R,5S)-di-tert-butyl 1-formyl-6,6-dimethylbicyclo[3.1.0]hexane-3,3-dicarboxylate 2b** (CP002)

Yellow liquid (transparent solid, when stored in refrigerator), yield: 70%; er = 93:7, determined by chiral HPLC. **^{1}H NMR** (400 MHz, $CDCl_3$) δ 9.28 (s, 1H), 3.09 (dd, J = 15.0, 1.6 Hz, 1H), 2.57 (ddd, J = 14.3, 7.2, 1.6 Hz, 1H), 2.31 (dd, J = 7.3, 3.2 Hz, 1H), 2.06 (d, J = 15.0 Hz, 1H), 1.87 (dd, J = 14.3, 3.2 Hz, 1H), 1.43 (d, J = 2.9 Hz, 18H), 1.38 (s, 3H), 1.18 (s, 3H). **^{13}C NMR** (101 MHz, $CDCl_3$) δ 200.34, 171.24, 169.58, 81.51, 81.33, 69.72, 52.24, 43.26, 38.02, 32.80, 30.99, 27.84, 27.75, 22.62, 17.40. **HRMS** (ESI-TOF) [M+Na] calculated for $[C_{19}H_{30}NaO_5]^+$ 361.1991, observed 361.1983. **HPLC** (Chiralpak AD-H column, 98:2 hexane/ethanol, flow rate: 1.0 mL/min): t_{major} = 5.146 min; t_{minor} = 4.246 min.

(18) **(1R,5S)-6,6-dimethyl-3-tosyl-3-azabicyclo[3.1.0]hexane-1-carbaldehyde 2C** (CP003)

Yellow liquid, yield: 82%; er = 93:7, determined by chiral HPLC. **^{1}H NMR** (400 MHz, $CDCl_3$) δ 9.23 (s, 1H), 7.69 (d, J = 8.2 Hz, 2H), 7.32 (d, J = 8.0 Hz, 2H), 3.83 (d, J = 10.4 Hz, 1H), 3.53 (d, J = 10.0 Hz, 1H), 3.42 – 3.31 (m, 2H), 2.42 (s, 3H), 2.22 (d, J = 5.3 Hz, 1H), 1.38 (s, 3H), 1.24 (s, 3H). **^{13}C NMR** (101 MHz, $CDCl_3$) δ 198.41, 143.76, 133.38, 129.80, 127.43, 48.03, 47.32, 45.71, 38.39, 32.87, 22.35, 21.53, 15.23. **HRMS** (ESI-TOF) [M+Na] calculated for $[C_{15}H_{19}NNaO_3S]^+$ 316.0983, observed 316.0987. **HPLC** (Chiralpak AD-H column, 20:80 hexane/ethanol, flow rate: 1.0 mL/min): t_{major} = 9.724 min; t_{minor} = 7.127 min.

(19) **(1R,5S)-6,6-diethyl-3-tosyl-3-azabicyclo[3.1.0]hexane-1-carbaldehyde 2d** (CP004)

Colorless liquid, yield: 82%; er = 97:3, determined by chiral HPLC. **^{1}H NMR** (400 MHz, $CDCl_3$) δ 9.21 (s, 1H), 7.70 (d, J = 8.2 Hz, 2H), 7.32 (d, J = 8.1 Hz, 2H), 3.91 (d, J = 10.6 Hz, 1H), 3.48 (d, J = 9.9 Hz, 1H), 3.39 (dd, J = 10.2, 5.7 Hz, 1H), 3.30 (d, J = 10.6 Hz, 1H), 2.42 (s, 3H), 2.25 (d, J = 5.1 Hz, 1H), 1.91 – 1.82 (m, 1H), 1.50 (dt, J = 9.3, 6.2 Hz, 3H), 0.90 (td, J = 7.4, 4.0 Hz, 6H). **^{13}C NMR** (101 MHz, CDCl3) δ 198.48, 143.75, 133.36, 129.79, 127.43, 48.61, 47.49, 45.81, 43.42, 38.70, 23.97, 21.54, 16.76, 10.86, 10.44. **HRMS** (ESI-TOF) [M+Na] calculated for $[C_{17}H_{23}NNaO_3S]^+$ 344.1296, observed 344.1290. **HPLC** (Chiralcel OJ-H column, 60:40 hexane/ethanol, flow rate: 1.0 mL/min): t_{major} = 10.363 min; t_{minor} = 14.574 min.

(20) **(1R,5S)-dibenzyl 1-formyl-6,6-dihexylbicyclo[3.1.0]hexane-3,3-dicarboxylate 2e** (CP005)

Colorless liquid, yield: 77%; er = 97:3, determined by chiral HPLC. **^{1}H NMR** (400 MHz, $CDCl_3$) δ 9.25 (s, 1H), 7.39 – 7.17 (m, 12H), 5.13 – 5.05 (m, 5H), 3.27 (dd, J = 15.1, 1.3 Hz, 1H), 2.67 (ddd, J = 14.3, 7.2, 1.2 Hz, 1H), 2.33 (dd, J = 7.3, 3.2 Hz, 1H), 2.09 (d, J = 15.1 Hz, 1H), 1.98 (dd, J = 14.5, 3.2 Hz, 1H), 1.81 – 1.68 (m, 1H), 1.33 – 1.18 (m, 18H), 0.88 (q, J = 6.9 Hz, 7H). **^{13}C NMR** (101 MHz, $CDCl_3$) δ 200.18, 171.70, 169.93, 135.33, 135.31, 128.57, 128.52, 128.30, 128.18, 127.97, 68.56, 67.53, 67.13, 52.48, 46.88, 43.09, 33.04, 31.92, 31.78, 31.70, 31.38, 29.43, 29.37, 26.76, 26.02, 25.74, 22.60, 14.10, 14.05. **HRMS** (ESI-TOF) [M+Na] calculated for $[C_{35}H_{46}NaO_5]^+$ 569.3243, observed 569.3238. **HPLC** (Chiralpak AD-H column, 90:10 hexane/ethanol, flow rate: 1.0 mL/min): t_{major} = 9.135 min; t_{minor} = 5.753 min.

(21) **(1R,5S)-3-tosyl-3-azaspiro[bicyclo[3.1.0]hexane-6,1'-cyclopentane]-1-carbaldehyde 2f** (CP006)

Colorless liquid, yield: 64%. er: 87:13, determined by chiral HPLC. **^{1}H NMR** (400 MHz, $CDCl_3$) δ 9.12 (s, 1H), 7.69 (d, J = 8.2 Hz, 2H), 7.32 (d, J = 8.2 Hz, 2H), 3.62 (d, J = 10.1 Hz, 1H), 3.55 (d, J = 9.9 Hz, 1H), 3.45 (d, J = 10.1 Hz, 1H), 3.21 (dd, J = 9.9, 4.8 Hz, 1H), 2.43 (s, 3H), 2.23 (d, J = 4.7 Hz, 1H), 1.85 – 1.60 (m, 8H). **^{13}C NMR** (101 MHz, $CDCl_3$) δ 198.22, 143.84, 133.30, 129.87, 127.59, 47.29, 46.63, 45.85, 41.77, 37.59, 33.50, 26.90, 25.70, 25.60, 21.63. **HRMS** (ESI-TOF) [M+H] calculated for $[C_{17}H_{22}NO_3S]^+$ 320.1320, observed 320.1329. [M+Na] calculated for $[C_{17}H_{21}NNaO_3S]^+$ 342.1140, observed 342.1137. **HPLC** (Chiralpak-AS column, 80: 20 hexane/ethanol, flow rate: 1.0 mL/min): t_{major} = 12.709 min; t_{minor} = 14.795 min.

(22) **(1R,5S)-3-tosyl-3-azaspiro[bicyclo[3.1.0]hexane-6,1'-cyclohexane]-1-carbaldehyde 2g** (CP007)

Colorless liquid, yield: 60%; er = 89.5:10.5, determined by chiral HPLC. **^{1}H NMR** (400 MHz, $CDCl_3$) δ 9.21 (s, 1H), 7.68 (d, J = 8.2 Hz, 2H), 7.31 (d, J = 8.0 Hz, 2H), 3.88 (d, J = 10.5 Hz, 1H), 3.48 (d, J = 9.9 Hz, 1H), 3.38 (dd, J = 10.0, 5.6 Hz, 1H), 3.30 (d, J =

10.5 Hz, 1H), 2.41 (s, 3H), 2.25 (d, J = 5.2 Hz, 1H), 1.83 – 1.16 (m, 10H). ^{13}**C NMR** (101 MHz, $CDCl_3$) δ 198.50, 143.75, 133.32, 129.78, 127.41, 48.07, 47.25, 45.62, 40.80, 38.01, 31.87, 25.94, 25.79, 25.62, 25.46, 21.52. **HRMS** (ESI-TOF) [M+H] calculated for $[C_{18}H_{23}NO_3S]^+$ 334.1477, observed 334.1473. **HPLC** (Chiralpak AD-H column, 20:80 hexane/ethanol, flow rate: 1.0 mL/min): t_{major} = 8.875 min; t_{minor} = 6.571 min.

(23) **(1R,5S)-3-tosyl-3-azaspiro[bicyclo[3.1.0]hexane-6,1'-cycloheptane]-1-carbaldehyde 2h** (CP008)

Colorless liquid, yield: 67%; er = 93:7, determined by chiral HPLC. ^{1}**H NMR** (400 MHz, $CDCl_3$) δ 9.19 (s, 1H), 7.69 (d, J = 8.2 Hz, 2H), 7.32 (d, J = 8.0 Hz, 2H), 3.80 (d, J = 10.5 Hz, 1H), 3.52 (d, J = 10.1 Hz, 1H), 3.41 (d, J = 10.5 Hz, 1H), 3.32 (dd, J = 10.1, 5.4 Hz, 1H), 2.42 (s, 3H), 2.23 (d, J = 5.2 Hz, 1H), 2.02 – 1.91 (m, 1H), 1.79 – 1.42 (m, 11H). ^{13}**C NMR** (101 MHz, $CDCl_3$) δ 198.79, 143.73, 133.27, 129.77, 127.44, 48.76, 47.26, 45.88, 41.27, 39.16, 35.01, 27.84, 27.81, 27.27, 27.04, 25.43, 21.53. **HRMS** (ESI-TOF) [M+Na] calculated for $[C_{19}H_{25}NNaO_3S]^+$ 370.1453, observed 370.1440. **HPLC** (Chiralcel OJ-H column, 60:40 hexane/ethanol, flow rate: 1.0 mL/min): t_{major} = 12.075 min; t_{minor} = 14.881 min.

(24) **(1R,5S)-2',2',6,6-tetramethylspiro[bicyclo[3.1.0]hexane-3,5'-[1,3]dioxane]-1-carbaldehyde 2i** (CP009)

Colorless liquid, yield: 75%; er = 96:4, determined by chiral HPLC. ^{1}**H NMR** (400 MHz, $CDCl_3$) δ 9.30 (s, 1H), 3.68 (d, J = 11.4 Hz, 1H), 3.60 – 3.47 (m, 2H), 3.38 (dd, J = 11.4, 1.3 Hz, 1H), 2.43 (d, J = 14.5 Hz, 1H), 2.23 (dd, J = 7.4, 2.7 Hz, 1H), 2.16 (dd, J = 13.9, 7.4 Hz, 1H), 1.64 (s, 1H), 1.40 (d, J = 2.0 Hz, 6H), 1.37 (s, 3H), 1.35 (s, 1H), 1.14 (d, J = 3.6 Hz, 3H). ^{13}**C NMR** (101 MHz, $CDCl_3$) δ 201.02, 97.93, 68.33, 67.36, 52.08, 50.92, 42.76, 37.13, 32.05, 28.86, 25.74, 22.73, 21.74, 17.48. **HRMS** (ESI-TOF) [M+H] calculated for $[C_{14}H_{23}O_3S]^+$ 239.1647, observed 239.1616. **HPLC** (Chiralcel OJ-H column, 80:20 hexane/ethanol, flow rate: 1.0 mL/min): t_{major} = 5.439 min; t_{minor} = 4.596 min

(25) **(1'R,5'S)-6',6'-dimethyl-1-tosylspiro[azetidine-3,3'-bicyclo[3.1.0]hexane]-1'-carbaldehyde 2j** (CP010)

Colorless liquid, yield: 80%; er = 93:7, determined by chiral HPLC. ^{1}**H NMR** (400 MHz, $CDCl_3$) δ 9.18 (s, 1H), 7.72 – 7.64 (m, 2H), 7.36 (d, J = 7.9 Hz, 2H), 3.64 (d, J = 7.8 Hz, 1H), 3.55 (dd, J = 13.0, 7.4 Hz, 3H), 2.59 (d, J = 14.9 Hz, 1H), 2.46 (s, 3H), 2.19 – 2.07 (m, 2H), 1.73 (d, J = 11.9 Hz, 1H), 1.44 (d, J = 14.9 Hz, 1H), 1.35 (s, 3H), 0.97 (s, 3H). ^{13}**C NMR** (101 MHz, $CDCl_3$) δ 200.43, 144.17, 131.10, 129.75, 128.39, 65.83, 60.64, 52.54, 46.56, 42.89, 38.27, 35.18, 35.16, 22.70, 21.64, 17.16. **HRMS** (ESI-TOF) [M+Na] calculated for $[C_{18}H_{23}NaNO_3S]^+$ 356.1296, observed 356.1325. **HPLC** (Chiralcel OD-H column, 80:20 hexane/ethanol, flow rate: 1.0 mL/min): t_{major} = 10.459 min; t_{minor} = 8.970 min.

(26) **(1S,5S)-3-tosyl-3-azabicyclo[3.1.0]hexane-1-carbaldehyde 2k** (CP011)

Colorless liquid, yield: 39%; er = 58:42, determined by chiral HPLC. ^{1}**H NMR** (400 MHz, $CDCl_3$) δ 8.86 (s, 1H), 7.72 – 7.63 (m, 2H), 7.34 (d, J = 7.9 Hz, 2H), 3.62 (dd, J = 22.1, 9.7 Hz, 2H), 3.41 (d, J = 9.7 Hz, 1H), 3.06 (dd, J = 9.6, 4.0 Hz, 1H), 2.44 (s, 3H), 2.15 – 2.04 (m, 1H), 1.52 (dd, J = 8.4, 5.7 Hz, 1H), 1.43 (t, J = 5.5 Hz, 1H). ^{13}**C NMR** (101 MHz, $CDCl_3$) δ 196.67, 143.99, 132.57, 129.84, 127.66, 48.69, 47.20, 39.82, 29.69, 25.63, 21.54, 15.79. **HRMS** (ESI-TOF) [M+H] calculated for $[C_{13}H_{16}NO_3S]^+$ 266.0851, observed 266.0839. **HPLC** (Chiralpak AD-H column, 60:40 hexane/ethanol, flow rate: 1.0 mL/min): t_{major} = 19.632 min; t_{minor} = 13.894 min.

(27) **(1S,5S)-5-methyl-3-tosyl-3-azabicyclo[3.1.0]hexane-1-carbaldehyde 2l** (CP012)

Colorless liquid, yield: 40%; er = 57:43, determined by chiral HPLC. ^{1}**H NMR** (400 MHz, $CDCl_3$) δ 9.12 (s, 1H), 7.68 (d, J = 8.3 Hz, 2H), 7.34 (d, J = 8.0 Hz, 2H), 3.61 (dd, J = 9.5, 6.0 Hz, 2H), 3.42 (d, J = 9.6 Hz, 1H), 2.85 (d, J = 9.4 Hz, 1H), 2.44 (s, 3H), 1.52 (dd, J = 18.6, 5.4 Hz, 2H), 1.32 (s, 3H). ^{13}**C NMR** (101 MHz, $CDCl_3$) δ 198.06, 143.98, 132.63, 129.85, 127.66, 54.33, 48.58, 41.82, 36.18, 22.64, 21.57, 15.39. **HRMS** (ESI-TOF) [M+Na] calculated for $[C_{14}H_{17}NNaO_3S]^+$ 302.0827, observed 302.0760. **HPLC** (Chiralpak AD-H column, 60:40 hexane/ethanol, flow rate: 1.0 mL/min): t_{major} = 13.271 min; t_{minor} = 10.571 min.

(28) **(1S,5S)-5,6,6-trimethyl-3-tosyl-3-azabicyclo[3.1.0]hexane-1-carbaldehyde 2m** (CP013)

Colorless liquid, yield: 71%; er = 91:9, determined by chiral HPLC. ^{1}**H NMR** (400 MHz, $CDCl_3$) δ 9.33 (s, 1H), 7.70 (d, J = 8.2 Hz, 2H), 7.33 (d, J = 8.1 Hz, 2H), 3.86 (d, J = 10.3 Hz, 1H), 3.59 (d, J = 9.8 Hz, 1H), 3.43 (d, J = 10.3 Hz, 1H), 3.21 (d, J = 9.8 Hz, 1H), 2.43 (s, 3H), 1.43 (s, 3H), 1.39 (s, 3H), 1.25 (s, 3H). ^{13}**C NMR** (101 MHz, $CDCl_3$) δ 199.42, 143.71, 133.62, 129.79, 127.40, 54.53, 47.12, 47.03, 43.55, 36.18, 21.55, 18.24, 16.70, 13.52. **HRMS** (ESI-TOF) [M+H] calculated for $[C_{16}H_{22}NO_3S]^+$ 308.1320, observed 308.1314. **HPLC** (Chiralpak AD-H column, 30:70 hexane/ethanol, flow rate: 1.0 mL/min): t_{major} = 5.607 min; t_{minor} = 6.428 min.

(29) **(1S,5S,6R)-6-methyl-3-tosyl-3-azabicyclo[3.1.0]hexane-1-carbaldehyde 2n** (CP014)

Colorless liquid. ^{1}**H NMR** (500 MHz, $CDCl_3$) δ 8.81 (s, 1H), 7.71 (d, J = 8.2 Hz, 2H), 7.34 (d, J = 8.1 Hz, 2H), 3.77 (d, J = 10.2 Hz, 1H), 3.54 (d, J = 9.9 Hz, 1H), 3.45 (d, J = 10.2 Hz, 1H), 3.35 (dd, J = 9.9, 5.2 Hz, 1H), 2.43 (s, 3H), 2.19 – 2.10 (m, 1H), 1.86 (dd, J = 8.8, 6.6 Hz, 1H), 1.22 (d, J = 6.5 Hz, 3H). ^{13}**C NMR** (126 MHz, $CDCl_3$) δ 197.18, 143.82, 133.30, 129.81, 127.53, 46.29, 44.69,

44.02, 30.13, 24.45, 21.50, 6.11. **HRMS** (ESI-TOF) [M+H] calculated for $[C_{14}H_{18}NO_3S]^+$ 280.1007, observed 280.0998. **HPLC** (Chiralpak AD-H column, 20:80 hexane/ethanol, flow rate: 1.0 mL/min): t_{major} = 11.200 min; t_{minor} = 10.053min.

(30) **(1S,5S,6S)-6-methyl-3-tosyl-3-azabicyclo[3.1.0]hexane-1-carbaldehyde 2n'** (CP015)

Colorless liquid. **^{1}H NMR** (400 MHz, $CDCl_3$) δ 9.24 (s, 1H), 7.67 (d, J = 8.3 Hz, 2H), 7.33 (d, J = 8.0 Hz, 2H), 3.66 (d, J = 9.5 Hz, 1H), 3.52 (d, J = 9.7 Hz, 1H), 3.36 (d, J = 9.7 Hz, 1H), 2.99 (dd, J = 9.5, 4.0 Hz, 1H), 2.43 (s, 3H), 2.14 – 2.07 (m, 1H), 1.92 – 1.78 (m, 1H), 1.37 (d, J = 6.6 Hz, 3H). **^{13}C NMR** (101 MHz, $CDCl_3$) δ 197.74, 143.90, 132.58, 129.79, 127.65, 49.03, 47.92, 43.22, 34.28, 25.80, 21.54, 12.23. **HRMS** (ESI-TOF) [M+H] calculated for $[C_{14}H_{18}NO_3S]^+$ 280.1007, observed 280.1011. **HPLC** (Chiralpak AD-H column, 20:80 hexane/ethanol, flow rate: 1.0 mL/min): t_{major} = 17.607 min; t_{minor} = 9.589 min.

(31) **(1R,5S,6R)-6-methyl-6-phenyl-3-tosyl-3-azabicyclo[3.1.0]hexane-1-carbaldehyde 2o** (CP016)

Colorless liquid, er = 72:28, determined by chiral HPLC. **^{1}H NMR** (400 MHz, $CDCl_3$) δ 8.43 (s, 1H), 7.74 (d, J = 8.3 Hz, 2H), 7.32 (dd, J = 14.2, 7.5 Hz, 5H), 7.24 (dd, J = 5.2, 3.0 Hz, 2H), 3.96 (d, J = 10.5 Hz, 1H), 3.72 (d, J = 10.1 Hz, 1H), 3.62 – 3.52 (m, 2H), 2.83 (d, J = 5.0 Hz, 1H), 2.43 (s, 3H), 1.52 (s, 3H). **^{13}C NMR** (126 MHz, $CDCl_3$) δ 197.62, 143.80, 140.93, 133.71 129.83, 129.11, 128.71, 127.56, 127.46, 48.31, 47.20, 45.31, 41.28, 34.19, 21.50, 17.29.. **HRMS** (ESI-TOF) [M+H] calculated for $[C_{20}H_{22}NO_3S]^+$ 356.1320, observed 356.1318. **HPLC** (Chiralpak AD-H column, 60:40 hexane/ethanol, flow rate: 1.2 mL/min): t_{major} = 13.939 min; t_{minor} = 13.228 min.

(32) **(1R,5S,6S)-6-methyl-6-phenyl-3-tosyl-3-azabicyclo[3.1.0]hexane-1-carbaldehyde 2o'** (CP017)

Colorless liquid, er = 57:43, determined by chiral HPLC. **^{1}H NMR** (400 MHz, $CDCl_3$) δ 9.52 (s, 1H), 7.41 – 7.26 (m, 5H), 7.20 – 7.09 (m, 4H), 3.66 (dd, J = 24.1, 10.0 Hz, 2H), 3.38 – 3.25 (m, 2H), 2.58 (d, J = 4.6 Hz, 1H), 2.38 (s, 3H), 1.62 (s, 3H). **^{13}C NMR** (101 MHz, $CDCl_3$) δ 198.08, 143.36, 138.00, 133.71, 129.54, 129.11, 127.34, 127.32, 127.00, 48.09, 46.87, 45.71, 39.94, 38.55, 24.85, 21.50. **HRMS** (ESI-TOF) [M+H] calculated for $[C_{20}H_{22}NO_3S]^+$ 356.1320, observed 356.1316. **HPLC** (Chiralpak AD-H column, 20:80 hexane/ethanol, flow rate: 1.0 mL/min): t_{major} = 16.780 min; t_{minor} = 6.997 min.

(33) **dibenzyl 2-(2-iodo-3-oxopropyl)-2-(3-methylbut-2-en-1-yl)malonate 4a** (CP018)

Yellow liquid. **^{1}H NMR** (400 MHz, $CDCl_3$) δ 9.00 (d, J = 2.5 Hz, 1H), 7.40 – 7.17 (m, 10H), 5.18 – 4.96 (m, 4H), 4.85 (ddd, J = 7.5, 6.1, 1.3 Hz, 1H), 4.75 (dt, J = 9.7, 3.0 Hz, 1H), 2.92 (dd, J = 15.1, 9.7 Hz, 1H), 2.66 (t, J = 7.1 Hz, 2H), 2.55 (dd, J = 15.1, 3.2 Hz, 1H), 1.65 (d, J = 0.7 Hz, 3H), 1.56 (s, 3H). **^{13}C NMR** (101 MHz, $CDCl_3$) δ 189.89, 170.22, 170.02, 137.01, 134.99, 134.87, 129.33, 128.58, 128.47, 128.38, 120.98, 117.81, 116.59, 67.58, 67.54, 58.27, 36.16, 32.97, 29.81, 26.00, 18.02. **HRMS** (ESI-TOF) [M+H] calculated for $[C_{25}H_{28}IO_5]^+$ 535.0981, observed 535. 0973.

(34) **N-(2-iodo-3-oxopropyl)-4-methyl-N-(3-methylbut-2-en-1-yl)benzenesulfonamide 4c** (CP019)

Colorless liquid. **^{1}H NMR** (400 MHz, $CDCl_3$) δ 9.27 (d, J = 3.1 Hz, 1H), 7.69 (d, J = 8.3 Hz, 2H), 7.33 (d, J = 8.0 Hz, 2H), 4.87 (ddd, J = 9.2, 5.3, 3.1 Hz, 2H), 3.83 (qd, J = 15.4, 7.2 Hz, 2H), 3.70 (dd, J = 15.2, 9.3 Hz, 1H), 3.40 (dd, J = 15.2, 5.3 Hz, 1H), 2.45 (s, 3H), 1.65 (d, J = 3.7 Hz, 6H). **^{13}C NMR** (101 MHz, $CDCl_3$) δ 191.17, 143.88, 138.67, 135.82, 129.83, 127.46, 118.03, 48.88, 47.36, 32.27, 25.81, 21.57, 17.84. **HRMS** (ESI-TOF) [M+H] calculated for $[C_{15}H_{21}INO_3S]^+$ 422.0287, observed 422.0296.

(35) **(1R,5S)-di-tert-butyl 1-(hydroxymethyl)-6,6-dimethylbicyclo[3.1.0]hexane-3,3-dicarboxylate 5b** (CP020)

Colorless liquid. **^{1}H NMR** (400 MHz, $CDCl_3$) δ 3.80 (d, J = 11.5 Hz, 1H), 3.55 (d, J = 11.6 Hz, 1H), 2.60 (s, 1H), 2.48 (dd, J = 14.9, 1.0 Hz, 1H), 2.31 (ddd, J = 14.8, 7.1, 1.1 Hz, 1H), 2.14 – 2.01 (m, 2H), 1.43 (d, J = 15.7 Hz, 18H), 1.20 (dd, J = 7.2, 2.5 Hz, 1H), 1.10 (s, 3H), 1.02 (s, 3H). **^{13}C NMR** (101 MHz, $CDCl_3$) δ 173.81, 169.97, 81.80, 80.93, 69.00, 65.15, 41.71, 37.19, 34.57, 34.13, 28.01, 27.84, 27.79, 22.88, 16.64. **HRMS** (ESI-TOF) [M+H] calculated for $[C_{19}H_{33}O_5]^+$ 341.2328, observed 341.2342.

(36) **((1R,5S)-6,6-dimethyl-3-tosyl-3-azabicyclo[3.1.0]hexan-1-yl)methanol 5c** (CP021)

Colorless liquid. **^{1}H NMR** (400 MHz, $CDCl_3$) δ 7.71 (d, J = 8.2 Hz, 2H), 7.31 (d, J = 8.0 Hz, 2H), 3.69 (s, 2H), 3.48 (q, J = 9.8 Hz, 2H), 3.42 – 3.36 (m, 2H), 2.43 (s, 3H), 1.18 – 1.13 (m, 1H), 1.12 (s, 3H), 1.02 (s, 3H). **^{13}C NMR** (101 MHz, $CDCl_3$) δ 143.36, 134.17, 129.62, 127.37, 63.94, 51.40, 48.12, 38.12, 31.58, 24.18, 22.49, 21.54, 14.50. **HRMS** (ESI-TOF) [M+Na] calculated for $[C_{15}H_{21}NNaO_3S]^+$ 318.1140, observed 318.1135.

(37) **(1R,5S)-di-tert-butyl 6,6-dimethyl-1-(morpholinomethyl)bicyclo[3.1.0]hexane-3,3-dicarboxylate 6b** (CP022)

Colorless liquid. **^{1}H NMR** (400 MHz, $CDCl_3$) δ 3.74 – 3.52 (m, 4H), 2.75 (d, J = 12.4 Hz, 1H), 2.57 (d, J = 15.0 Hz, 1H), 2.45 (ddd, J = 14.6, 7.1, 1.0 Hz, 1H), 2.41 – 2.20 (m, 4H), 2.16 (d, J = 12.4 Hz, 1H), 2.06 (d, J = 15.0 Hz, 1H), 1.91 (dd, J = 14.6, 2.3 Hz, 1H), 1.42 (d, J = 15.7 Hz, 18H), 1.00 (d, J = 14.8 Hz, 6H), 0.92 (dd, J = 7.1, 2.3 Hz, 1H). **^{13}C NMR** (101 MHz, $CDCl_3$) δ 172.57,

170.44, 80.79, 80.77, 68.93, 67.17, 61.40, 53.42, 37.71, 37.30, 34.00, 33.67, 27.85, 27.41, 23.42, 16.54. **HRMS** (ESI-TOF) [M+H] calculated for $[C_{23}H_{40}NO_5]^+$ 410.2906, observed 410.2893.

(38) **(E)-methyl 3-((1S,5S)-6,6-dimethyl-3-tosyl-3-azabicyclo[3.1.0]hexan-1-yl)acrylate 7c** (CP023)

Colorless liquid. **^{1}H NMR** (400 MHz, $CDCl_3$) δ 7.69 (d, J = 8.2 Hz, 2H), 7.32 (d, J = 8.0 Hz, 2H), 6.86 (d, J = 15.8 Hz, 1H), 5.69 (d, J = 15.8 Hz, 1H), 3.70 (s, 3H), 3.53 – 3.39 (m, 3H), 3.32 (dd, J = 9.7, 5.2 Hz, 1H), 2.43 (s, 3H), 1.64 (d, J = 5.1 Hz, 1H), 1.17 (d, J = 8.5 Hz, 6H). **^{13}C NMR** (101 MHz, $CDCl_3$) δ 166.58, 148.62, 143.66, 133.50, 129.76, 127.39, 119.87, 51.53, 48.83, 47.43, 37.89, 37.65, 29.95, 23.69, 21.53, 14.73. **HRMS** (ESI-TOF) [M+H] calculated for $[C_{18}H_{24}NO_4S]^+$ 350.1426, observed 350.1431.

(39) **(1R,5S)-1-ethynyl-6,6-dimethyl-3-tosyl-3-azabicyclo[3.1.0]hexane 8c** (CP024)

Colorless solid. **^{1}H NMR** (400 MHz, $CDCl_3$) δ 7.68 (d, J = 8.2 Hz, 2H), 7.32 (d, J = 8.1 Hz, 2H), 3.55 (d, J = 9.7 Hz, 1H), 3.47 – 3.21 (m, 3H), 2.43 (s, 3H), 2.02 (s, 1H), 1.48 (d, J = 4.8 Hz, 1H), 1.18 (s, 3H), 1.11 (s, 3H). **^{13}C NMR** (101 MHz, $CDCl_3$) δ 143.64, 133.56, 129.74, 127.42, 82.92, 68.97, 51.37, 47.49, 36.25, 27.05, 26.43, 24.60, 21.53, 13.31. **HRMS** (ESI-TOF) [M+H] calculated for $[C_{16}H_{20}NO_2S]^+$ 290.1215, observed 290.1219.

(40) **(S)-di-tert-butyl 3-methylene-4-(prop-1-en-2-yl)cyclopentane-1,1-dicarboxylate 13b** (CP025)

Colorless liquid. **^{1}H NMR** (400 MHz, $CDCl_3$) δ 4.99 (d, J = 1.7 Hz, 1H), 4.83 (s, 2H), 4.77 (d, J = 2.3 Hz, 1H), 3.38 – 3.19 (m, 1H), 2.94 (dd, J = 16.8, 1.3 Hz, 1H), 2.81 (dd, J = 16.8, 2.5 Hz, 1H), 2.40 (ddd, J = 12.9, 7.9, 1.4 Hz, 1H), 2.04 (dd, J = 12.9, 11.4 Hz, 1H), 1.65 (s, 3H), 1.44 (dd, J = 5.5, 1.9 Hz, 18H). **^{13}C NMR** (101 MHz, $CDCl_3$) δ 170.99, 170.78, 150.00, 145.10, 113.09, 107.51, 81.29, 81.17, 59.95, 51.06, 40.62, 38.34, 27.86, 27.84, 18.16. **HRMS** (ESI-TOF) [M+Na] calculated for $[C_{19}H_{30}NaO_4]^+$ 345.2042, observed 345.2040.

(41) **(1R,5S)-6,6-dimethyl-1-((S)-oxiran-2-yl)-3-tosyl-3-azabicyclo[3.1.0]hexane 9c** (CP026)

Colorless liquid. **^{1}H NMR** (400 MHz, $CDCl_3$) δ 7.68 (d, J = 8.2 Hz, 2H), 7.32 (d, J = 8.1 Hz, 2H), 3.35 (dd, J = 10.8, 6.9 Hz, 2H), 3.25 (q, J = 9.6 Hz, 2H), 2.96 – 2.89 (m, 1H), 2.75 – 2.68 (m, 1H), 2.43 (s, 3H), 2.25 (dd, J = 4.9, 2.7 Hz, 1H), 1.29 (d, J = 4.2 Hz, 1H), 1.16 (s, 3H), 1.04 (s, 3H). **^{13}C NMR** (101 MHz, $CDCl_3$) δ 143.46, 133.97, 129.64, 127.33, 50.12, 48.53, 47.92, 45.67, 35.72, 30.27, 24.25, 21.97, 21.53, 14.34. **HRMS** (ESI-TOF) [M+Na] calculated for $[C_{16}H_{21}NNaO_3S]^+$ 330.1140, observed 330.1127.

(42) **(1S,5S)-1,6,6-trimethyl-3-tosyl-3-azabicyclo[3.1.0]hexane 10c** (CP027)

Colorless liquid. **^{1}H NMR** (400 MHz, $CDCl_3$) δ 7.69 (d, J = 8.2 Hz, 2H), 7.31 (d, J = 8.2 Hz, 2H), 3.41 – 3.33 (m, 3H), 3.10 (d, J = 9.5 Hz, 1H), 2.42 (s, 3H), 1.12 (s, 3H), 0.98 (d, J = 16.2 Hz, 6H), 0.86 (d, J = 1.7 Hz, 1H). **^{13}C NMR** (101 MHz, $CDCl_3$) δ 143.19, 134.27, 129.55, 127.35, 54.06, 48.34, 32.67, 30.86, 23.19, 22.67, 21.51, 15.69, 14.06. **HRMS** (ESI-TOF) [M+H] calculated for $[C_{15}H_{22}NO_2S]^+$ 280.1371, observed 280.1362.

(43) **(R)-3-(prop-1-en-2-yl)-1-tosylpyrrolidine 11c** (CP028)

Colorless liquid. **^{1}H NMR** (400 MHz, $CDCl_3$) δ 7.72 (d, J = 8.2 Hz, 2H), 7.33 (d, J = 8.2 Hz, 2H), 4.73 (s, 1H), 4.63 (s, 1H), 3.49 (dd, J = 9.6, 7.7 Hz, 1H), 3.46–3.39 (m, 1H), 3.24 (td, J = 9.5, 6.9 Hz, 1H), 3.01 (t, J = 9.4 Hz, 1H), 2.67–2.54 (m, 1H), 2.44 (s, 3H), 1.93 (dtd, J = 9.7, 6.7, 3.0 Hz, 1H), 1.66 (d, J = 5.6 Hz, 4H). **^{13}C NMR** δ 143.71, 143.35, 134.04, 129.65, 127.50, 110.83, 51.53, 47.68, 45.12, 29.97, 21.52, 21.01. **HRMS** (ESI-TOF) [M+H] calculated for $[C_{14}H_{20}NO_2S]^+$ 266.1215, observed 266.1256.

(44) **(3S,4R)-4-(prop-1-en-2-yl)-1-tosylpyrrolidine-3-carbaldehyde 12c** (CP029)

Colorless liquid. **^{1}H NMR** (400 MHz, $CDCl_3$) δ 9.50 (d, J = 1.9 Hz, 1H), 7.70 (d, J = 8.2Hz, 2H), 7.33 (d, J = 8.2 Hz, 2H), 4.84 (s, 1H), 4.76 (s, 1H), 3.58 – 3.46 (m, 3H), 3.10 (dd, J = 9.9, 7.8 Hz, 1H), 2.96 - 2.85 (m, 2H), 2.45 (s, 3H), 1.69 (s, 3H). **^{13}C NMR** (101 MHz, $CDCl_3$) δ 198.96, 143.95, 141.49, 133.15, 129.83, 127.64, 113.29, 53.38, 51.55, 47.00, 46.29, 21.55, 20.48. **HRMS** (ESI-TOF) [M+H] calculated for $[C_{15}H_{20}NO_3S]^+$ 294.1164, observed 294.1149.
